# What a Near-Perfect AUROC Does Not Show: Random-Panel and Label-Permutation Controls for a Sepsis Blood-Transcriptome Signature

**DOI:** 10.3390/ijms27209273

**Published:** 2026-10-19

**Authors:** Mikhail Kolev, Irina Naskinova, Meglena Lazarova, Velin Klisurov, Hristo Kalinov

**Affiliations:** 1 Department of Applied Computer Science and Mathematical Modelling, Faculty of Mathematics and Computer Science, University of Warmia and Mazury in Olsztyn, Słoneczna 54, 10-710 Olsztyn, Poland; mikhail.kolev@uwm.edu.pl; 2 Department of Mathematics, University of Architecture, Civil Engineering and Geodesy (UACEG), 1 Hristo Smirnenski Blvd., 1164 Sofia, Bulgaria; inaskinova_fte@uacg.bg; 3 Department of Mathematical Modelling and Numerical Methods, Faculty of Applied Mathematics and Informatics, Technical University of Sofia, 8 “Kliment Ohridski” Blvd., 1000 Sofia, Bulgaria; 4 Faculty of Mathematics and Informatics, Sofia University “St. Kliment Ohridski”, 5 James Bourchier Blvd., 1164 Sofia, Bulgaria; klisurovi4@gmail.com (V.K.); hkalinov@uni-sofia.bg (H.K.)

**Keywords:** diagnostic specificity, host response, microarray, benchmark saturation, cross-platform validation, feature transportability, nested cross-validation, selection stability

## Abstract

Sepsis blood-transcriptome signatures are usually judged against healthy controls. Two questions are rarely separated: whether the classifier exceeds chance, and whether its genes outperform an arbitrary list of equal size. We tested both on public microarray data (discovery GSE65682, *n* = 359; validation GSE57065, *n* = 53), using the 9983 of 11,222 genes measurable on both platforms. A 10-gene signature was selected by nested cross-validation and applied to validation without refitting. It separated sepsis from health perfectly (area under the receiver operating characteristic curve, AUROC, 1.000). The pipeline exceeded chance: no label permutation in 1000 matched it (*p* = 0.001). The genes did not outperform random lists: 12.5% of 2000 random 10-gene panels matched or exceeded the signature, as did 67.6% of panels drawn from the same 300 preselected candidates. Against non-infected intensive care patients, cross-fitted AUROC fell to 0.730 (95% confidence interval 0.642–0.818), unchanged after age and sex matching. At a threshold of 0.5, the cross-fitted model called 70 of these 75 patients septic. Selection was unstable: 200 bootstrap resamples gave a median of 6 genes, from 109 distinct genes. A near-perfect AUROC against healthy controls reflects an easy benchmark, not a specific gene list.

## 1. Introduction

Sepsis is life-threatening organ dysfunction caused by a dysregulated host response to infection [1], and it remains one of the largest contributors to global mortality [2]. Treatment consists of source control, timely antimicrobials, and organ support, all of which are time-critical and largely supportive [3]. The practical bottleneck is therefore rarely the choice of therapy but rather recognition. The clinician must decide, in a patient who is already critically ill, whether infection lies behind the deterioration. That decision is hard because the syndrome is non-specific. Fever, leukocytosis, tachycardia, and organ dysfunction accompany trauma, pancreatitis, major surgery, and sterile inflammation as readily as they accompany infection. The Sepsis-3 definition makes clear that sepsis is a problem of differential diagnosis among patients who are already ill, not a screen that separates the sick from the well [1].

Whole-blood host-response transcriptomics has been studied for two decades as a way past this bottleneck. The rationale is that the leukocyte transcriptome integrates the response to a pathogen more directly than any single circulating analyte [4]. The field has produced important results. Multicohort analyses have derived diagnostic gene sets that separate infectious from sterile inflammation [5], and published diagnostics have been benchmarked against one another on pooled public data [6]. Unsupervised analyses have identified reproducible molecular subtypes of bacterial sepsis [7]. Single-cell work has resolved a monocyte state that is expanded in bacterial sepsis [8]. Independent groups have described blood genomic endotypes and sepsis response signatures that stratify patients by outcome and, in one randomized setting, by treatment response [9,10,11]. More recent work has combined host expression into composite scores of immune dysfunction [12], mechanistic endotypes assigned at first presentation [13], and integrated host–microbe assays [14]. Two host-expression assays have progressed far enough to be compared head-to-head in acute infection [15], and one has been evaluated beyond bacterial sepsis [16]. Reviews appear regularly [17,18], as do new candidate signatures assembled from public cohorts [19,20].

Against this background, one reporting convention has received little scrutiny. Many blood-transcriptomic sepsis signatures are reported by their discrimination against healthy control subjects, and an area under the receiver operating characteristic curve (AUROC) [21] close to 1.0 on that comparison is taken as evidence that the selected genes are diagnostic. However, discriminating sepsis from health and discriminating infection from generic critical illness are different claims, and only the second addresses the clinical question. When healthy volunteers are the only available comparator, the two claims cannot be separated, and apparent performance is inflated by an amount that the sepsis-versus-healthy contrast cannot itself estimate. Work on sepsis-induced immune dysfunction explains why. Much of what can be measured in blood reflects the magnitude and phase of a generic dysregulated response, including immunoparalysis, monocyte deactivation, and lymphocyte loss, rather than the presence of a pathogen [22,23,24,25,26]. Strategies that stratify patients by such states are valuable in their own right [27], but they do not detect infection.

Two negative controls address two different null hypotheses. The first asks whether the classifier performs better than chance. It is tested by permuting the class labels and re-running the selection procedure [28]. The second asks whether the particular genes chosen perform better than an arbitrary panel of the same size drawn from the same pool. Oncology has already confronted the second question. Michiels et al. showed that published breast-cancer outcome signatures were unstable under repeated random validation [29]. Ein-Dor et al. showed that thousands of samples would be needed before such gene lists stabilized [30]. Venet et al. showed that most random gene-expression signatures are significantly associated with breast-cancer outcome [31], and this positive bias persists in recent analyses [32]. A signature can pass the first test and fail the second, yet the two are often reported as if one implied the other. Neither test is routine in the sepsis-signature literature.

Cross-platform feature availability is also rarely checked before selection. A signature can be validated externally only on genes that the target platform measures. If candidate selection uses the full discovery feature space, some selected genes may be absent from the validation array. The “validated” signature then becomes a different and smaller model than the one selected. Platform and batch effects in expression data are well characterized [33]. Correction methods exist [34], as do cross-platform normalization methods that allow joint training [35]. However, correcting across the discovery/validation boundary risks passing information across the very split that the validation is meant to protect. A more conservative option is to audit feature availability first and to restrict the candidate pool accordingly, which is the approach taken here.

We address these issues using two public microarray cohorts. Both negative controls are run as primary analyses: a random-gene-panel benchmark, in which selected and random panels are fitted with the same modeling recipe, and a class-label permutation test, in which the candidate filter is re-derived within every permutation. Specificity is estimated by cross-fitting against non-infected intensive care unit (ICU) controls, so that no patient contributes to the model that scores them. The external validation crosses cohorts and platforms, with different patients, a different hospital, and a different array. A feature-availability audit precedes it, and the model is locked before it sees the validation data. A full-pipeline bootstrap repeats feature selection and hyperparameter tuning in every resample, which gives both a realistic interval and a direct measure of how reproducible the gene list is. Finally, the validation result provides a worked example of when an AUROC confidence interval should not be reported.

The analysis follows established safeguards against the optimism that has repeatedly affected omics classifiers. Feature selection is repeated within every outer cross-validation fold rather than performed once on the full data, which avoids the error that most often inflates reported accuracy [36,37]. Reporting follows general recommendations for developing and validating prediction models [38,39,40].

The primary objective of this study was to determine whether a discovery-derived blood-transcriptomic sepsis signature (a) classifies better than chance and (b) outperforms random gene panels of the same size drawn from the same candidate pool. The secondary objective was to quantify how much of the discrimination is specific to infection, by cross-fitting the locked selection procedure against non-infected ICU controls, and to test whether the remaining signal transfers across microarray platforms.

## 2. Results

**Figure 1:**
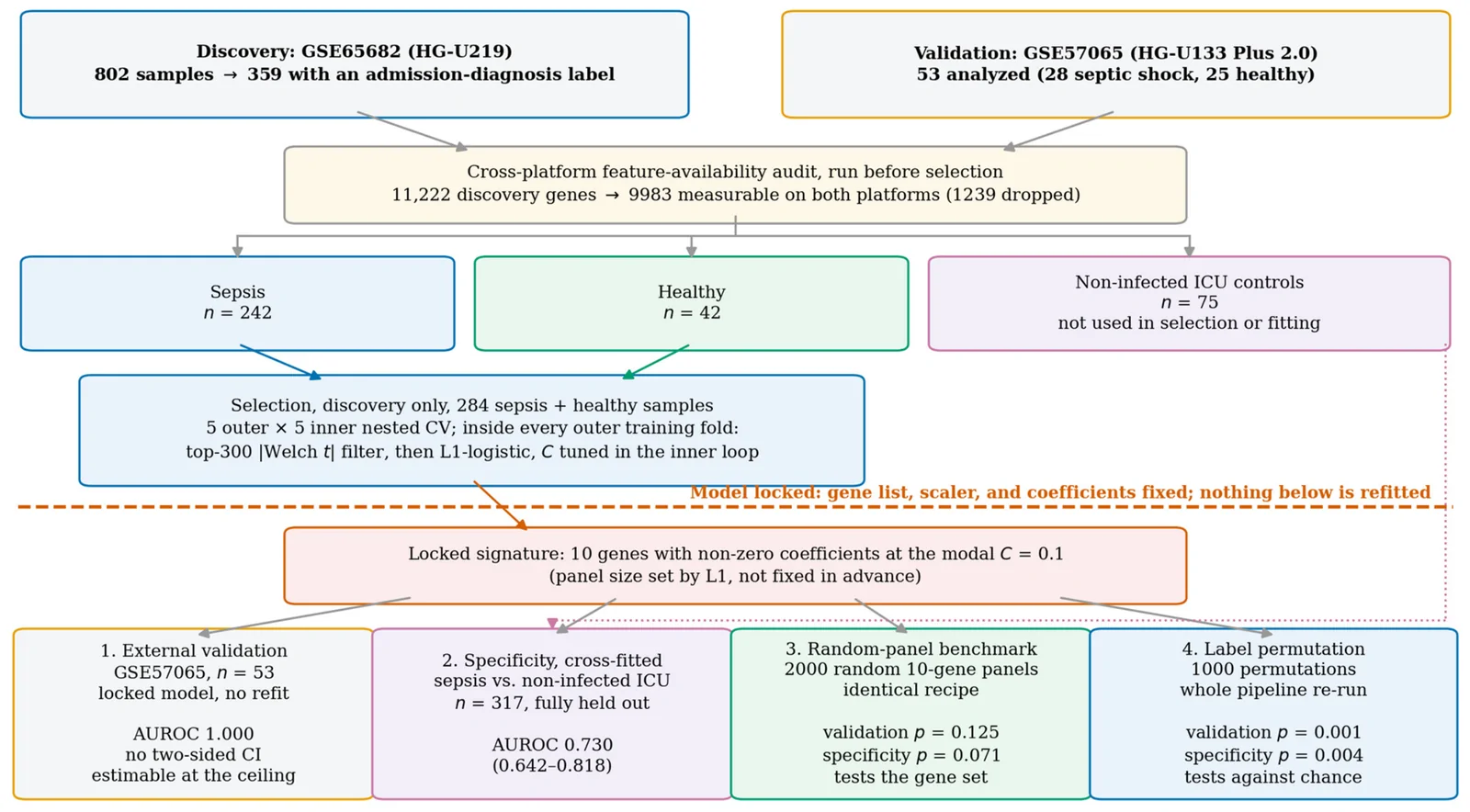
Sample flow, the point at which the model is locked, and what each evaluation tests. Boxes give the cohort or analysis step with its sample or gene count; arrows give the order of operations. The dashed horizontal line marks the locking point, after which the gene list, scaler, coefficients, and intercept are fixed and nothing is refitted. The 75 non-infected ICU patients (dotted path) never enter selection or fitting and are used only in the cross-fitted specificity evaluation. The four boxes on the bottom row are the four evaluations, each annotated with what it tests and what it found.

The analysis rests on one signature, selected once on the discovery cohort and then scored without refitting in four evaluations, each of which answers a different question. The sample flow through both series, the point at which the model was locked, and the question addressed by each evaluation are shown in Figure 1. The cohorts themselves, and the between-group comparisons on which the interpretation depends, are described in Table 1.

### 2.1. The Pipeline Outperforms Chance, but the Gene List Does Not Outperform Random Panels

The label-permutation test (Section 4.9) asks whether the pipeline performs better than chance, and the random-gene-panel benchmark (Section 4.8) asks whether the selected gene list outperforms arbitrary lists of the same size fitted with the same recipe. The two questions had different answers.

**Figure 2:**
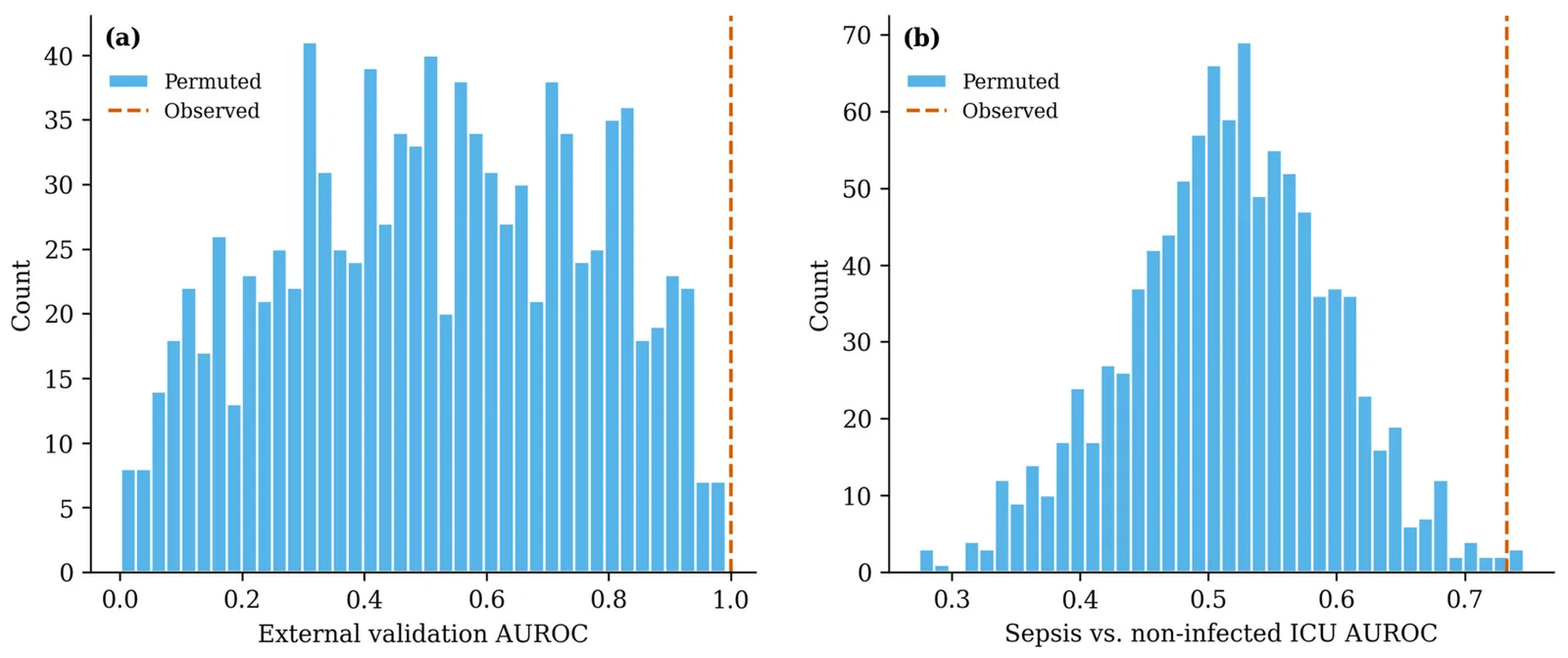
Permuting the class labels and re-running the whole pipeline shows that the classifier performs far above chance. (**a**) Histogram of external-validation AUROC across 1000 permutations of the discovery class labels, with the top-300 candidate filter re-derived inside every permutation (bars, Permuted); the dashed vertical reference line marks the unpermuted result (Observed), an AUROC of 1.000. No permutation reached it (*p* = 0.001). (**b**) The same permutation test scored on the sepsis versus non-infected ICU task (bars, Permuted; dashed vertical reference line, Observed), where the observed AUROC is 0.733; 3/1000 permutations matched or exceeded it (*p* = 0.004). AUROC, area under the receiver operating characteristic curve; ICU, intensive care unit.

The label-permutation test is shown in Figure 2. Permuting the discovery class labels and re-running the entire selection-and-fit pipeline 1000 times produced null distributions centered near chance on both tasks. On external validation, no permutation matched or exceeded the observed AUROC of 1.000 (0 of 1000; null mean 0.514, standard deviation [SD] 0.248; empirical *p* = 0.001). On the specificity task, 3 of 1000 permutations matched or exceeded the observed 0.733 (null mean 0.515, SD 0.080; *p* = 0.004). The pipeline therefore extracted real label information on both tasks.

**Figure 3:**
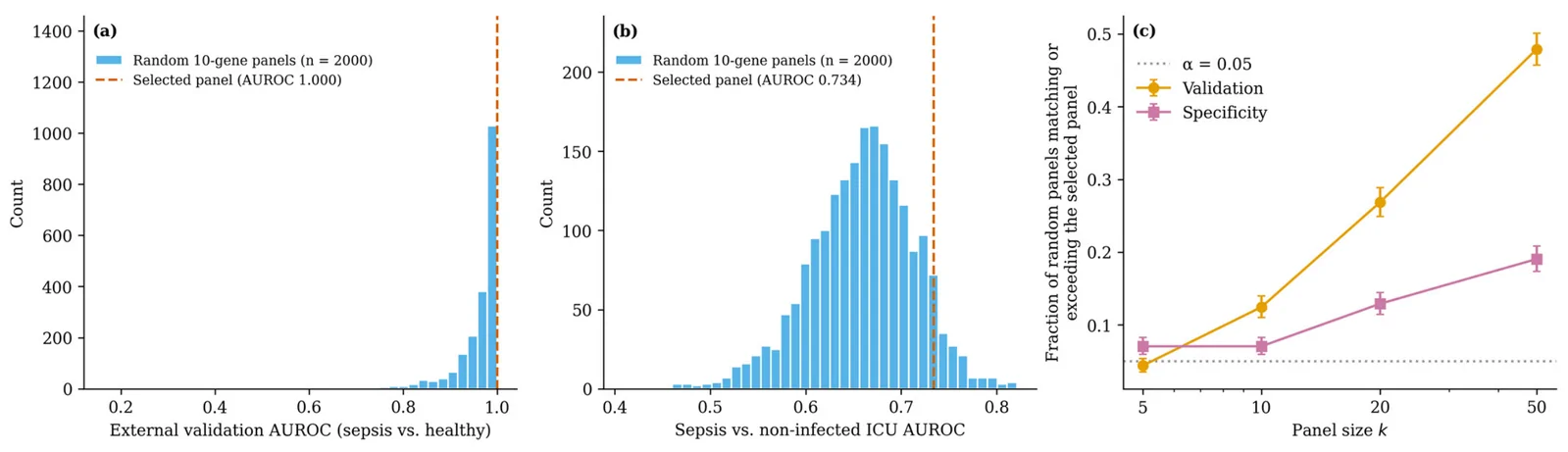
The selected gene panel does not significantly outperform random panels of the same size, and the gap narrows as panels grow. (**a**) Histogram of external-validation AUROC across 2000 random 10-gene panels drawn from the 9983-gene transportable pool and fitted by the signature’s own L1 recipe (bars, Random 10-gene panels (*n* = 2000)); the dashed vertical reference line marks the selected panel, Selected panel (AUROC 1.000). In total, 249/2000 (12.5%) matched or exceeded it (*p* = 0.125). (**b**) The same benchmark on the sepsis versus non-infected ICU task (bars, Random 10-gene panels (*n* = 2000); dashed vertical reference line, Selected panel (AUROC 0.734)); 141/2000 (7.1%) matched or exceeded it (*p* = 0.071). (**c**) Panel-size sweep, with the selected panel resized by the same selection procedure at every *k*, plotted as connected markers with Clopper–Pearson error bars for Validation (circles) and Specificity (squares) per the legend, against the dotted horizontal reference line at *α* = 0.05. The *x*-axis is logarithmic. AUROC, area under the receiver operating characteristic curve; ICU, intensive care unit.

The comparison with random panels of the same size is shown in Figure 3. Under the primary specification, panels were drawn from the 9983-gene transportable pool at the realized size *k* = 10 and fitted with the signature’s own L1 recipe. On external validation, 249 of 2000 random panels (12.5%, 95% confidence interval [CI] 11.0–14.0) matched or exceeded the selected panel (selected 1.000; null mean 0.960, SD 0.063; empirical *p* = 0.125). On the specificity task, 141 of 2000 did so (7.1%, 95% CI 6.0–8.3; selected 0.734; null mean 0.659, SD 0.055; *p* = 0.071). Neither result reached *p* < 0.05. The selected panel lay above the null mean on both tasks, but at these sample sizes, its margin could not be distinguished from chance selection.

The result did not depend on the choice of ten genes. When the panel size was varied and the selected panel was resized by the same procedure at each *k*, the proportions of random panels that matched or exceeded it were 4.4%, 12.5%, 26.9%, and 47.9% on validation, and 7.1%, 7.1%, 12.9%, and 19.1% on the specificity task, at *k* = 5, 10, 20, and 50 (Figure 3c). The advantage of the selected panel shrank as panels grew, as benchmark saturation predicts: the larger an arbitrary panel, the more likely it is to contain genes that separate these groups. The nominally significant validation cell at *k* = 5 (*p* = 0.044) is one of eight cells and is not corrected for multiple comparisons.

Two specification checks gave consistent results. Under the unpenalized recipe, the proportions were 7.9% and 10.5%. When the null panels were drawn from the selection-matched 300-gene candidate pool instead of the whole transportable set, the proportions were 67.6% on validation and 15.8% on the specificity task. Once the |*t*| filter had been applied, the L1 step therefore chose among the remaining genes no better than a random selection of ten. No duplicate panel was drawn in any cell. Table 2 collects what each evaluation tests and what it found.

### 2.2. Sepsis Versus Non-Infected ICU Controls, Cross-Fitted

**Figure 4:**
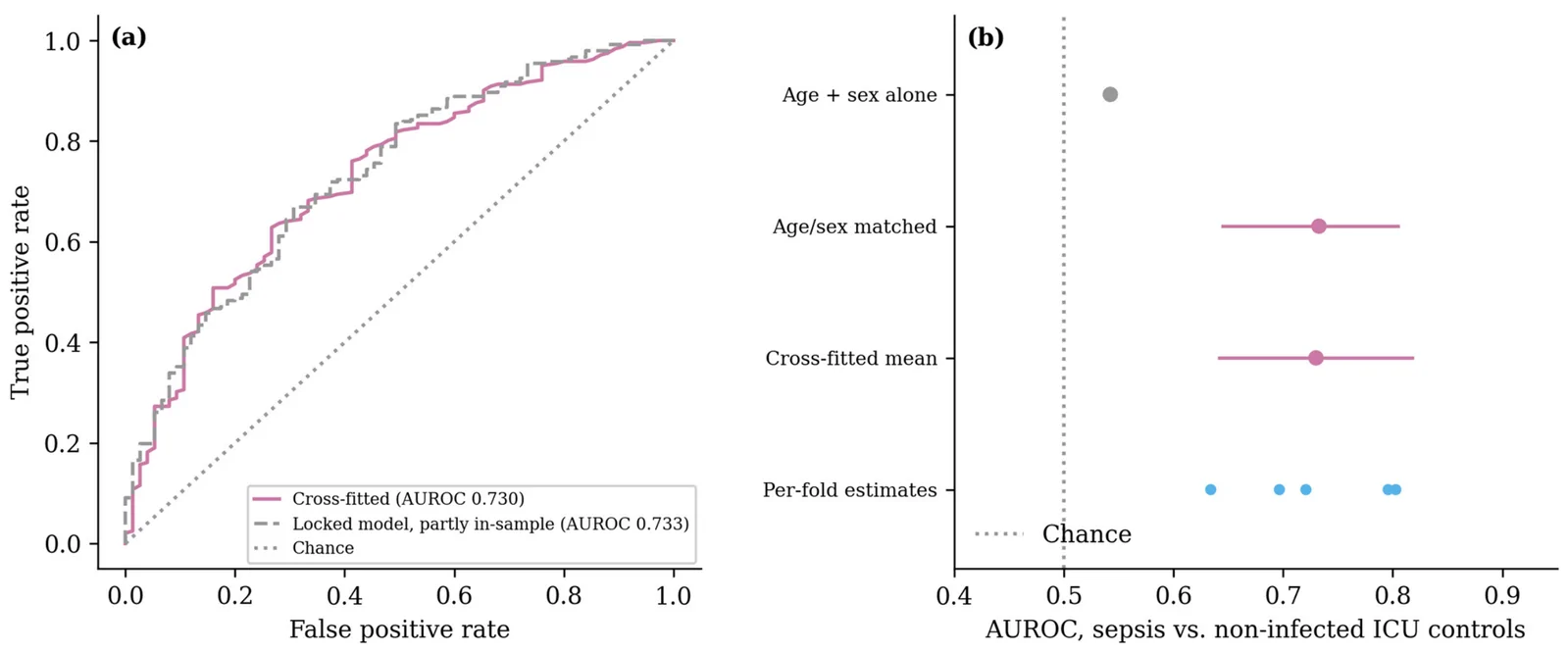
Cross-fitting the specificity analysis leaves the estimate essentially unchanged, and demography does not explain it. (**a**) Receiver-operating-characteristic curves for sepsis versus non-infected ICU controls: the cross-fitted predictions, in which no sample contributes to the model that scores it (solid pink line, Cross-fitted (AUROC 0.730)), and the partly in-sample scores of the final locked model, which saw every sepsis case in training (dashed gray line, Locked model, partly in-sample (AUROC 0.733)), against the dotted gray diagonal reference line, Chance. (**b**) The same quantity estimated four ways, plotted as markers with horizontal 95% confidence-interval bars where an interval applies: the five individual fold estimates (blue dots, no interval), the cross-fitted mean and the age- and sex-matched estimate (pink dots with pink horizontal bars), and the AUROC obtained from age and sex alone (gray dot, no interval), against the dotted gray vertical reference line at Chance. AUROC, area under the receiver operating characteristic curve.

Against the 75 non-infected ICU controls, the cross-fitted selection procedure (Section 4.6), in which no sepsis case contributed to the model that scored it, reached AUROC 0.730 (95% CI 0.642–0.818). This is the mean of fivefold estimates, which ranged from 0.634 to 0.803 (Figure 4). Pooling the out-of-fold predictions after within-fold rank normalization gave 0.730 (0.660–0.789). Pooling the raw probabilities gave a lower value, 0.632 (0.554–0.703). Each fold model is calibrated on its own training partition, and the offsets between folds degrade the pooled ranking, so the lower value reflects pooling rather than a difference in discrimination.

The pooled estimate mixes two sub-studies. The 317 patients come from the pneumonia sub-study of the Molecular Diagnosis and Risk Stratification of Sepsis (MARS) consortium (192 sepsis, 33 non-infected) and the abdominal sub-study (50 sepsis, 42 non-infected). Sepsis prevalence differs between them (85.3% and 54.3%), and among sepsis patients, whole-blood expression identifies the sub-study with a cross-validated AUROC of 0.886. A pooled AUROC over such a mixture can be biased in either direction, so we also estimated it within each sub-study (Table S14b). The cross-fitted AUROC was 0.737 in the pneumonia sub-study and 0.882 in the abdominal sub-study, with a stratified average of 0.773 against 0.730 pooled. The pooled figure is therefore the more conservative estimate and was retained as the main one. The two sub-studies address different clinical questions, so neither within-sub-study value replaces it.

Scoring the final locked model against the same controls gave 0.733 (0.664–0.792). This estimate is partly in-sample because the model was trained on every sepsis case, and cross-fitting removed an optimism of only 0.003.

Demography did not explain the result. Age and sex alone discriminated sepsis from non-infected ICU controls with AUROC 0.542. Adding the signature score to a model containing both improved the fit substantially (likelihood-ratio *χ*^2^_1_ = 37.7, *p* = 8 × 10^−10^; adjusted odds ratio 23.7 per unit of the rank score, 95% CI 7.8–71.4). We matched each of the 75 controls to a sepsis case exactly on sex and within a 5-year age caliper. The 75 pairs had identical mean age (59.1 versus 59.1 years, *p* = 0.987) and gave AUROC 0.733 (0.646–0.805), essentially the same as the unmatched estimate. Discrimination was almost identical in women (0.733) and men (0.732) and decreased with age across tertiles (0.790, 0.733, 0.618).

The healthy-control benchmark, in contrast, is confounded by demography. In the validation cohort, age and sex alone discriminated sepsis from health with AUROC 0.884. Demography alone therefore accounts for most of the separation on the contrast that yields the near-perfect AUROC, and for almost none on the contrast that yields 0.730.

Discrimination is not the same as clinical usefulness (Table 3). At its nominal threshold of 0.5, the cross-fitted model correctly classified 98.8% of sepsis cases but only 6.7% of non-infected ICU controls. It labeled 70 of the 75 non-infected ICU patients as septic, and its balanced accuracy of 0.527 was barely above the 0.5 expected by chance. At the Youden-optimal threshold, which was chosen on these data and is therefore optimistic, sensitivity was 0.872 and specificity 0.360. Calibration was poor, with an intercept of −0.689 and a slope of 0.240 against ideal values of 0 and 1, and the Brier score was 0.182. A model trained to separate sepsis from health thus ranked these patients above chance without providing a usable decision rule for the bedside question.

### 2.3. The Gene List Is Not Reproducible Under Resampling

Repeating the entire selection procedure in 200 bootstrap resamples of the discovery cohort gave an out-of-bag discovery AUROC of 0.999 (95% CI 0.991–1.000). The external validation AUROC was 1.000 in 199 of 200 replicates (lowest 0.9986), and the specificity AUROC was 0.715 (0.662–0.771). Discrimination was therefore stable under resampling.

The gene list, in contrast, was not stable. Across the 200 replicates, the signature contained a median of 6 genes (range 3 to 19), and 109 distinct genes were selected at least once. Among the ten genes of the locked signature, re-selection frequency ranged from 76.5% (*P2RY10*) to 8.0% (*KLRB1*). The lead gene *S100A8*, which has the largest coefficient, was re-selected in only 41.5% of resamples. The tuning parameter also varied: the most frequently chosen *C* was 0.03 (130 replicates), not the 0.1 of the locked fit.

This instability mirrors the random-panel result: when a benchmark is saturated enough that most 10-gene panels succeed, the data barely determine which ten genes are chosen.

### 2.4. Discovery and Validation on the Conventional Task

On the conventional sepsis-versus-healthy task, discrimination reached the ceiling in both cohorts, and the evaluations differ mainly in what their intervals include. The estimates and the interval method used for each are collected in Table 4.

**Figure 5:**
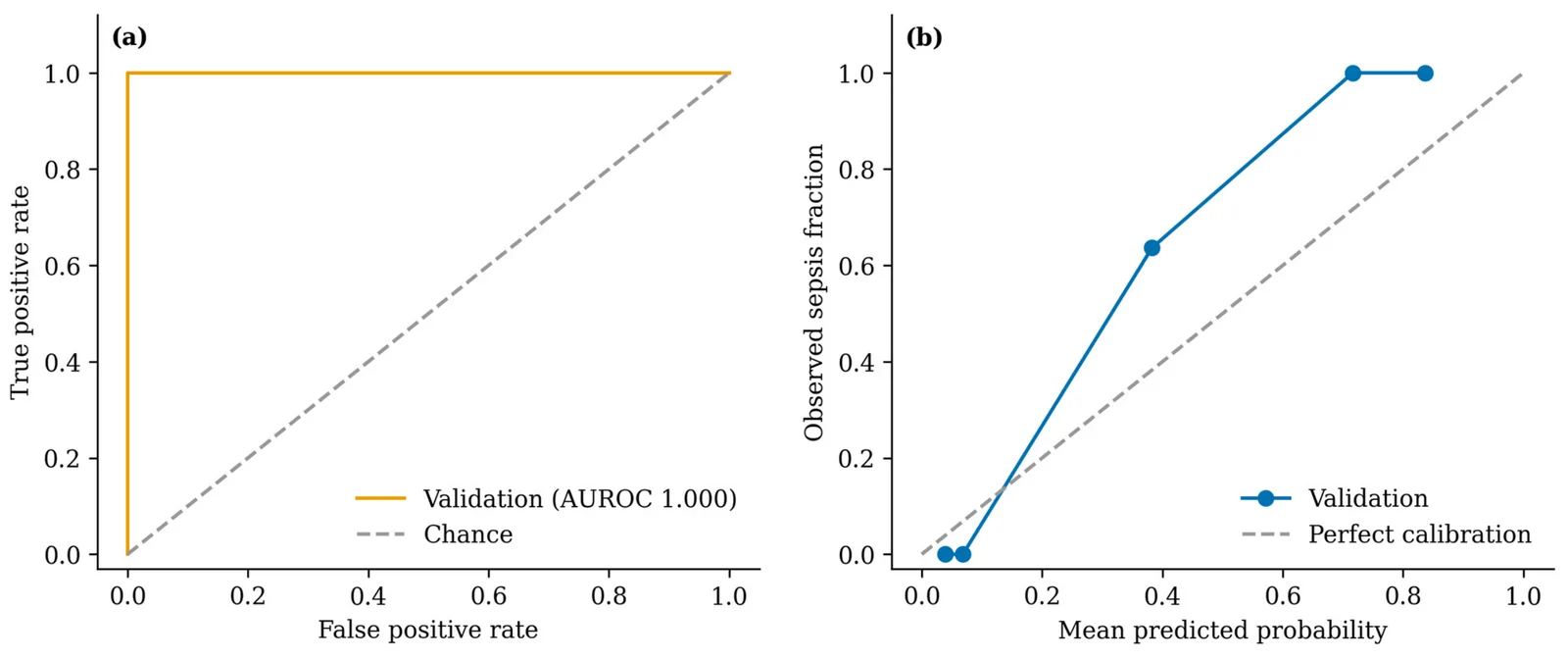
External validation shows ceiling discrimination on the conventional task. (**a**) Receiver-operating-characteristic curve for the locked 10-gene model scored on the 53-sample validation cohort (solid line, Validation (AUROC 1.000)); the dashed diagonal reference line is Chance. (**b**) Reliability curve for the same predictions (5 quantile bins; solid line with circular markers, Validation), plotting observed sepsis fraction against mean predicted probability in each bin, against the dashed diagonal reference line, Perfect calibration; Brier score 0.054. AUROC, area under the receiver operating characteristic curve.

The locked model scored on GSE57065 reached AUROC 1.000 with a Brier score of 0.054 (Figure 5). The separation was genuine rather than a numerical artifact: healthy scores ranged from 0.027 to 0.142 and sepsis scores from 0.419 to 0.881, a margin of 0.276 with no ties and no saturated values. Discovery and validation AUROCs differed by −0.001, so performance on this task did not degrade on transfer, consistent with Section 2.1. At the 0.5 threshold, the locked model gave a sensitivity of 0.929, a specificity of 1.000, and a balanced accuracy of 0.964 on validation. These values describe a saturated benchmark and are reported for completeness.

### 2.5. Dataset, Cohort, and Platform Structure

**Figure 6:**
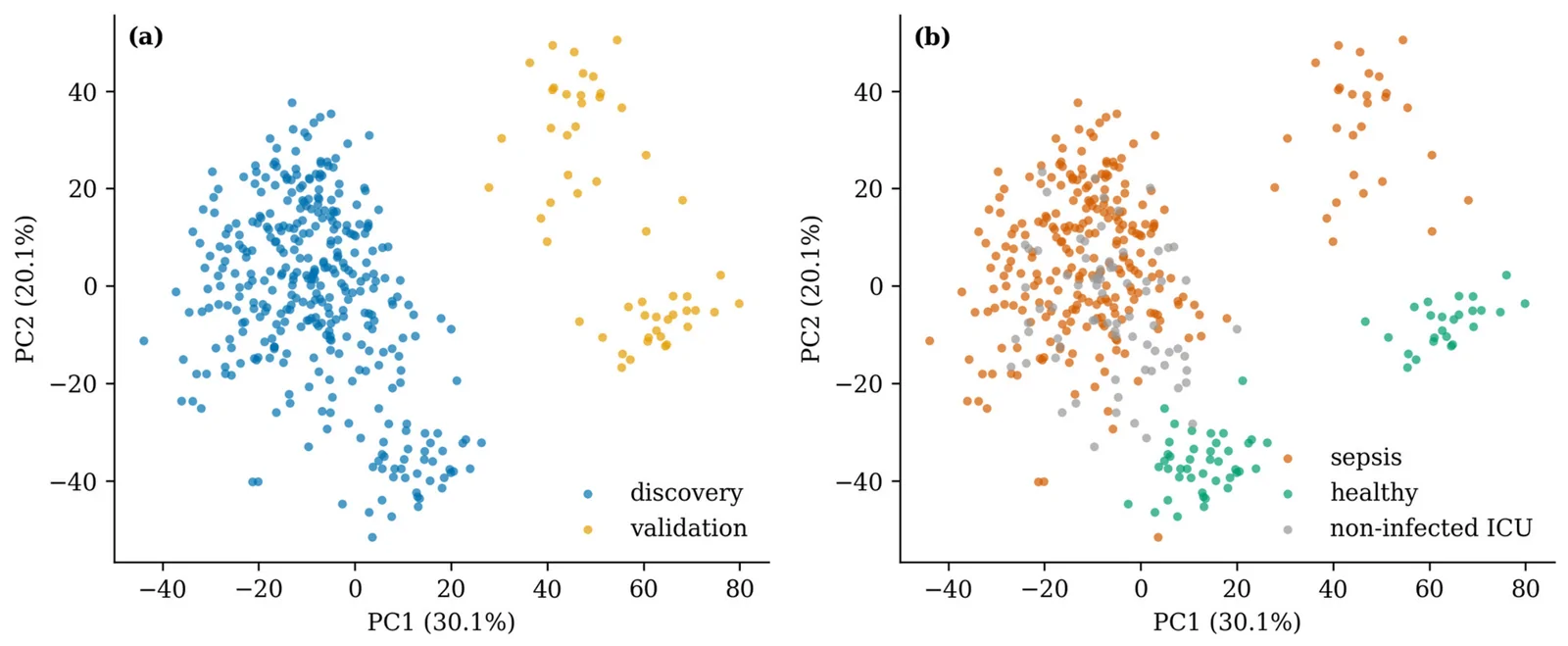
Discovery and validation occupy overlapping but distinguishable regions of transcriptome space. (**a**) Principal-component ordination of all discovery and validation samples pooled (top 2000 highest-variance transportable genes, one standardization fitted on the combined matrix, 10 components retained); each point is one sample, plotted as a scatter marker with no connecting line, grouped by dataset membership (discovery; validation) per the legend. The first two principal components (PC1 and PC2) explain 50.2% of pooled variance, and permutational multivariate analysis of variance (PERMANOVA) on the first 10 components attributes *R*^2^ = 0.311 to dataset membership (pseudo-*F* = 184.9, *p* = 0.001). (**b**) The identical ordination with the same points grouped instead by diagnosis (sepsis; healthy; non-infected ICU), for which the same test gives *R*^2^ = 0.125 (pseudo-*F* = 58.5, *p* = 0.001). The deconfounded estimates are in Section 2.5. ICU, intensive care unit.

In the pooled ordination (Figure 6), the first two principal components (PC1 and PC2) explained 50.2% of the variance. Permutational multivariate analysis of variance (PERMANOVA) attributed *R*^2^ = 0.311 to dataset membership (pseudo-*F* = 184.9, *p* = 0.001) and *R*^2^ = 0.125 to sepsis status (pseudo-*F* = 58.5, *p* = 0.001). These two quantities are confounded, and neither is a platform effect on its own.

The deconfounded estimates are more informative. Among healthy donors only, who appear in both series and therefore share the same diagnosis, dataset membership accounted for *R*^2^ = 0.811 (pseudo-*F* = 279.6, *p* = 0.001, *n* = 67). Within discovery only, where platform, cohort, and hospital are constant, sepsis status accounted for *R*^2^ = 0.262 (pseudo-*F* = 100.3, *p* = 0.001, *n* = 284). In a two-factor sequential decomposition of the pooled sepsis and healthy samples, the dataset entering first explained *R*^2^ = 0.336 and diagnosis given dataset 0.178. With diagnosis entering first, the values were 0.239 for diagnosis and 0.266 for dataset given diagnosis. The two orders disagree, as expected in a confounded design, so no single value is given as the platform effect.

### 2.6. Over-Representation of the Differential-Expression Set

**Figure 7:**
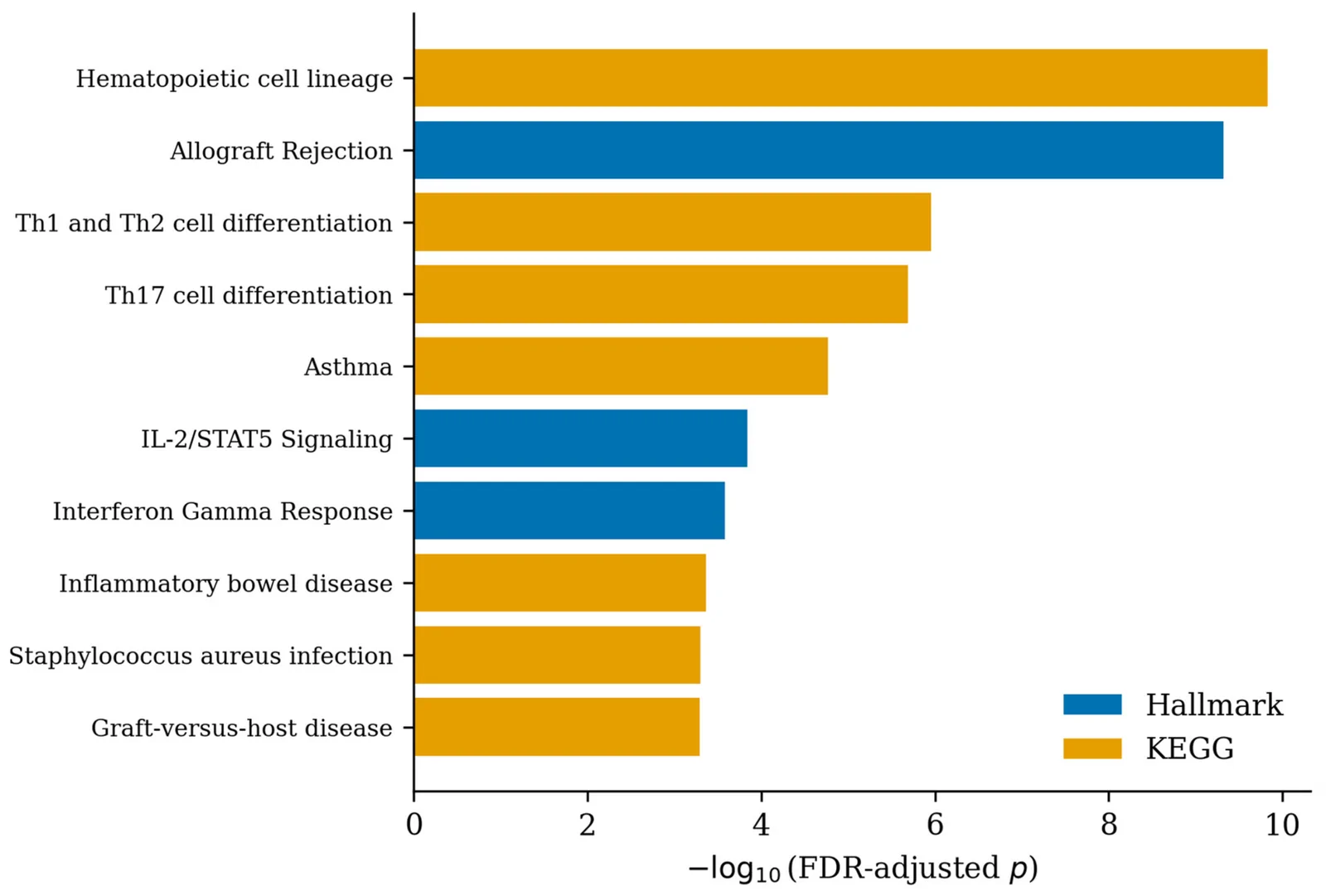
The top 10 Hallmark and KEGG terms by Benjamini–Hochberg-adjusted *p*-value for the 1508-gene discovery differential-expression set (hypergeometric over-representation test, background = the 9983-gene transportable universe, correction family = all 5742 testable terms across the three libraries), ranked by bar length as −log_10_(adjusted *p*); bars are grouped by source library (Hallmark; KEGG) per the legend. KEGG, Kyoto Encyclopedia of Genes and Genomes.

The gene-set libraries were the Hallmark collection, the Kyoto Encyclopedia of Genes and Genomes (KEGG), and the Gene Ontology (GO) Biological Process terms. With the multiple-testing family defined as all 5742 testable terms across the three libraries, 56 terms reached a false discovery rate (FDR) *p* < 0.05 for the 1508-gene discovery set (Figure 7).

Leading Hallmark terms were Allograft Rejection (61/156 genes, *p*adj = 4.8 × 10^−10^), IL-2/STAT5 Signaling (50/162, 1.4 × 10^−4^), Interferon Gamma Response (52/175, 2.6 × 10^−4^), Myc Targets V1 (46/175, 1.2 × 10^−2^), and Complement (39/148, 2.9 × 10^−2^). Leading KEGG terms were Hematopoietic Cell Lineage (38/70, 1.5 × 10^−10^), Th1 and Th2 Cell Differentiation (33/73, 1.1 × 10^−6^), Th17 Cell Differentiation (36/86, 2.1 × 10^−6^), and Staphylococcus aureus Infection (19/40, 5.0 × 10^−4^). The leading GO Biological Process term was Positive Regulation of T Cell Activation (36/79, 1.9 × 10^−7^).

**Figure 8:**
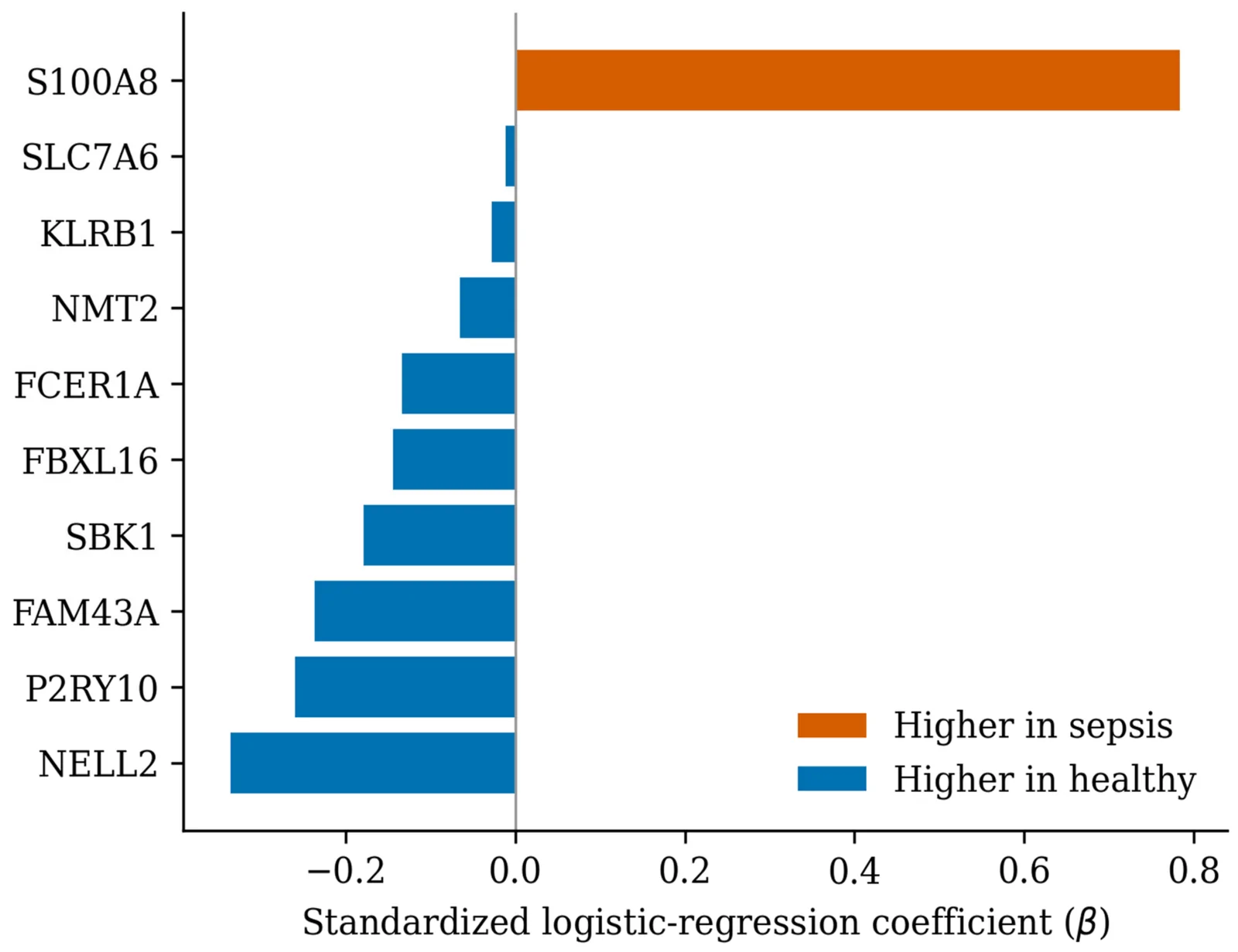
Standardized L1-logistic-regression coefficients (*β*) for the 10 genes of the locked signature, one bar per gene, ordered by coefficient value; bars extending in the positive direction indicate higher expression in sepsis and bars extending in the negative direction indicate higher expression in healthy controls, per the legend. Section 2.3 reports how often each of these genes is re-selected when the whole procedure is repeated on resampled data, which ranges from 76.5% to 8.0%.

The 10-gene locked signature (Figure 8) returned no FDR-significant term in any library (Table S5). This is expected for a set of that size, and the result is an underpowered null rather than evidence against the result for the differential-expression set. The test is non-directional, so none of these terms is interpreted as an activated pathway.

## 3. Discussion

### 3.1. Two Negative Controls, Two Different Answers

A 10-gene signature selected within nested cross-validation reached AUROC 1.000 when scored, without refitting, on an independent septic-shock cohort profiled on a different platform. By the conventions of this literature, that is a strong external validation. Read against its negative controls, however, it contains two separate findings.

The classifier captures real signals. Permuting the class labels and re-running the whole selection procedure 1000 times never reproduced the validation result and reproduced the specificity result only three times (*p* = 0.001 and *p* = 0.004).

The particular gene list, however, is not special. Against 2000 random 10-gene panels drawn from the same pool and fitted identically, 12.5% matched or exceeded it on validation and 7.1% on the specificity task. Against panels drawn from the 300 genes that the filter had already selected, 67.6% did. Either analysis alone would leave the other question open. Together, they show that the pipeline works but that the specific genes it names are close to arbitrary among the many that would work as well.

The differential-expression step shows the mechanism. Of the 9983 genes measurable on both platforms, 8319 (83.3%) reached Benjamini–Hochberg significance for sepsis versus healthy [41]. When five-sixths of the assayed transcriptome separates two groups, almost any compact panel drawn from it will separate them too, and the discrimination metric then says little about the genes chosen. Venet et al. demonstrated this phenomenon for breast-cancer outcome signatures [31]. Earlier work had shown that published signatures were unstable under repeated random validation and would need far larger samples to stabilize [29,30], and the problem is not confined to one disease area [32]. Widespread differential expression does not imply that no useful biomarker exists in sepsis, only that this comparison cannot show that one has been found.

### 3.2. Selection Instability Reflects the Same Saturation

Repeating the entire procedure in 200 bootstrap resamples of the same cohort returned a median of 6 genes rather than 10 (range 3 to 19), drawn from a pool of 109 distinct genes. *S100A8* was re-selected in 41.5% of replicates and *KLRB1* in 8.0%. Discrimination, meanwhile, was almost perfectly stable: the out-of-bag AUROC was 0.999, and the validation AUROC was 1.000 in 199 of 200 replicates.

A signature that predicts equally well while naming a different set of genes each time cannot be interpreted as a biological finding, whatever its AUROC. Stability selection was designed to expose this behavior [42], and although the analysis is inexpensive, signature papers rarely report it. The instability also explains the random-panel result: the data barely determine which ten genes to choose, so ten genes chosen at random perform almost as well.

### 3.3. The Specificity Estimate Is Stable Under Cross-Fitting and Matching

Scoring the final locked model against the non-infected controls reuses all 242 sepsis cases that trained it. Cross-fitting, in which no sample is scored by a model that saw it, gives 0.730, against 0.733 for the partly in-sample score, an optimism of 0.003. Exact matching on sex and within a 5-year age caliper gave 0.733. Age and sex alone reach only 0.542 on this contrast, and adding the score to a demographic model improves it substantially (*p* = 8 × 10^−10^).

This estimate therefore holds under cross-fitting and matching, and it is the clinically relevant one. It reflects real signals, well above chance, on the comparison that resembles the bedside decision. The gap between it and the near-ceiling healthy-control result is not, however, a direct estimate of how much of the benchmark reflects generic critical illness. The two AUROCs come from different comparator populations, and a difference between AUROCs computed against different negative classes does not decompose into infection-specific and illness-generic components. The narrower statement is that the same selection procedure yields 1.000 against healthy volunteers and 0.730 against non-infected ICU patients. These are not two scores of one fixed model. The 1.000 comes from the locked model on the external cohort, whereas the 0.730 is a cross-fitted estimate from five models refitted within the folds, which differ in their genes and tuning (Table S9a). Only the second comparison resembles the clinical question.

Demography sharpens the contrast. In the validation cohort, age and sex alone reach AUROC 0.884, so most of the separation rewarded by the conventional benchmark is available without measuring any gene. On the specificity contrast, they reach 0.542, so the benchmark that produces the high AUROC is also the confounded one.

Discrimination does not imply clinical usefulness. At its nominal threshold, the cross-fitted model labeled 70 of the 75 non-infected ICU patients as septic, with a balanced accuracy of 0.527 and a calibration slope of 0.240. When a model developed against healthy controls is applied to the clinical comparator, it loses more than accuracy. Its operating point loses its meaning because the score distribution on which it was calibrated is no longer present. This limitation is not apparent from the AUROC and becomes visible only when metrics beyond AUROC are reported.

### 3.4. When a Confidence Interval Should Not Be Reported

The discovery interval, 0.996–1.000, reaches the AUROC ceiling, and its narrowness does not indicate precision. The interval is narrow because separation is almost complete, so nearly every resample reproduces it, and its width reflects a sampling distribution pressed against a boundary.

The validation interval raises a more serious issue. At an AUROC of exactly 1.000, the DeLong variance [43] is zero and the subject-level bootstrap is degenerate, so no two-sided interval can be estimated. A possible workaround is to treat the AUROC as a proportion of concordant case–control pairs and apply a Wilson interval to the 700 pairs [44], which gives 0.995–1.000. That interval is invalid because the 700 pairs share 53 subjects and are not independent observations. The effective sample size is the number of subjects, so the interval is far too narrow. No two-sided interval is therefore reported for this row. Instead, we give a conservative one-sided lower bound of 0.887 from the rule of three [45], based on the 25 case–control comparisons that can be formed without reusing a subject.

The point applies beyond this study. A metric at its boundary produces no interval, and an empty cell in a results table can look like an omission, which makes any formula that returns two numbers tempting. A narrow interval obtained in this way is an artifact of treating dependent comparisons as independent, not a precise estimate. The appropriate report is the point estimate, a statement of why no interval exists, a conservative bound, and the separation margin showing that the estimate is real rather than numerical. The case illustrates a broader problem, in which conventionally reported statistics do not support the inferences drawn from them [46].

### 3.5. Dataset Structure and the Absence of Cross-Cohort Correction

The pooled ordination attributes *R*^2^ = 0.311 to dataset membership and 0.125 to sepsis status. These quantities are confounded because dataset, platform, hospital, cohort, and sample composition all change together across this boundary. The design cannot isolate a platform effect, so deconfounded estimates are reported instead. Among healthy donors alone, where diagnosis is constant, dataset membership accounts for *R*^2^ = 0.811. Within discovery alone, where platform is constant, sepsis status accounts for *R*^2^ = 0.262.

These estimates support the decision not to correct across cohorts. Batch correction across the boundary [33,34], or cross-platform normalization fitted to both cohorts jointly [35], would have improved apparent transfer. It would also have let information cross the split that the external validation is meant to protect. The more conservative arrangement is to restrict candidates to the 89.0% of discovery genes measurable on the validation platform, to standardize with discovery statistics only, and to lock the model before validation. The feature-availability audit is useful independently of the negative finding, since a signature that contains genes the target platform does not measure cannot be validated externally, whatever its discovery performance. The main lesson of the second phase of the MicroArray Quality Control project (MAQC-II) was that reproducibility across platforms depends on this kind of procedural discipline [47].

Scan batch within the discovery series is a separate question. Each GSE65682 sample title records its hybridization date. Healthy volunteers were scanned on only 7 of the 14 dates, so group and scan date are strongly associated (*χ*^2^ = 77.6, *p* < 0.001). The submitters’ ComBat adjustment appears to have removed the date signal itself. Among sepsis patients, where diagnosis is constant, expression predicted scan date no better than chance (mean one-versus-rest cross-validated AUROC 0.378 for the 2000 most variable genes and 0.440 for the 10 signature genes). Restricted to the seven dates on which healthy volunteers were scanned, the locked signature still separated 103 sepsis patients from 42 healthy volunteers completely (Table S14b). Scan batch therefore does not explain the sepsis-versus-healthy result, which was also reproduced in an independently processed series. One caveat cannot be resolved with the deposited data. When batch adjustment protects a group covariate in a design where groups are unevenly spread across batches, it can exaggerate group differences. We cannot tell whether the submitters protected diagnosis. If they did, within-discovery estimates, including the 0.730, could be modestly inflated. The external validation is unaffected by this adjustment.

### 3.6. Over-Representation Results

The 1508-gene differential-expression set is over-represented for allograft rejection, IL-2/STAT5 signaling, interferon-gamma response, complement, hematopoietic cell lineage, and Th1/Th2/Th17 differentiation terms [48,49,50,51,52]. This is consistent with the T-cell activation, interferon signaling, and lineage-shift processes documented in sepsis immunobiology [22,24]. The association is hypothesis-generating and describes the discovery cohort only. The hypergeometric test is non-directional, so on this evidence, none of these terms is an activated pathway. Over-representation analysis without adjustment for cell composition also cannot support inference about specific cell states. The analysis cannot show that any pathway drives, causes, or mechanistically explains classifier performance, and no wet-lab validation was performed.

### 3.7. Limitations

The most important limitation is that the specificity result is internal to the discovery cohort. GSE57065 has no non-infected ICU arm, so the most clinically important estimate in this study has not been validated externally. Cross-fitting removes the in-sample optimism but cannot replace a second cohort. The next step is a public blood-transcriptome series with sepsis, non-infected ICU, and healthy arms on a third platform, with the pre-specified question of whether 0.730 replicates. Until then, the specificity estimate is a within-cohort finding, and any clinical claim based on it is provisional.

Severity is not matched, and severity data are not available. The deposited characteristics do not record ventilation or vasopressor status. It is therefore unknown whether the sepsis and non-infected groups were similarly ill, beyond the fact that both were ICU admissions under the same protocol. If the groups differ systematically in severity, part of the 0.730 reflects that difference rather than infection, and the public data cannot resolve this.

Age and sex confound the healthy-control contrasts. Demography alone reaches AUROC 0.884 on the validation contrast. This is a further reason why the healthy-control benchmark overstates infection-specific performance, and it means that the near-ceiling values should not be read as gene-specific. The specificity contrast is demographically balanced and is unchanged by matching, which makes it the stronger of the two results.

The validation cohort is small. Twenty-eight sepsis patients and 25 healthy subjects provide a limited basis for any claim, and the small size is one reason why no two-sided interval can be estimated at AUROC 1.000. Conclusions drawn from this cohort are correspondingly weak, all the more so given its marked demographic imbalance.

Both cohorts were profiled on microarrays, not by RNA sequencing (RNA-seq), and both are adult ICU populations. Transfer to RNA-seq platforms, to emergency-department presentations, and to pediatric populations [53] is therefore untested.

Several further constraints apply. The sepsis group is defined by admission diagnosis rather than by Sepsis-3 criteria applied uniformly, which is unavoidable in secondary use of archived series [54]. Preprocessing follows the submitters’ robust multi-array average (RMA) pipelines [55] rather than uniform reprocessing from raw intensities. The L1-logistic model class [56] was fixed in advance, and no architecture search was performed. A different learner might select a different panel, although the benchmark concerns the comparison rather than the learner, and a more flexible model is not expected to escape a saturated benchmark. The full-pipeline bootstrap uses 200 replicates, so its percentiles carry more Monte Carlo error than the 2000-draw benchmarks, and its interval is an approximation. Finally, this is a computational reanalysis of public data, without wet-lab or protein-level confirmation.

The 10-gene model is a fixed diagnostic model: its coefficients are locked, and it can be scored on new data without change. It is not a validated tool for clinical use, and Section 2.3 shows that it is not a stable list of biologically important genes.

## 4. Materials and Methods

The design described in this section is shown in overview in Figure 1, which gives the sample flow, the point at which the model was locked, and the question addressed by each of the four evaluations.

### 4.1. Data Sources and Sample Selection

The discovery cohort was GSE65682, the whole-blood leukocyte transcriptome series of the MARS consortium described by Scicluna et al. [57]. It comprises ICU admissions and healthy volunteers profiled on the Affymetrix HG-U219 array (Affymetrix, Santa Clara, CA, USA; platform GPL13667), and its public series matrix was obtained from the Gene Expression Omnibus (GEO) of the National Center for Biotechnology Information (NCBI) [54]. Before deposition, the submitters had applied RMA background correction [55] and quantile normalization, pre-filtered the matrix to 24,646 variance-passing probes, and removed non-experimental chip effects with surrogate variable analysis and empirical-Bayes ComBat adjustment [34]. The public matrix is therefore already batch-adjusted within this series, and Section 3.5 examines what that adjustment left behind.

The series contains 802 samples, all with distinct patient codes in their sample titles. Of these, 359 belonged to two MARS sub-studies, on pneumonia and on abdominal sepsis, that defined both cases and non-infected comparators. These samples came from 359 distinct patients and were assigned to three groups: 242 patients with sepsis (an admission diagnosis of community-acquired pneumonia, hospital-acquired pneumonia, or abdominal sepsis), 75 non-infected ICU controls (a non-infectious ICU admission or a gastrointestinal-surgery control), and 42 healthy volunteers. All patient samples in the series were drawn at or after ICU admission, according to the GEO series description. The remaining 443 samples belonged to neither sub-study and were excluded, so that every case and every non-infected comparator came from a sub-study that defined both. They comprised 248 sepsis patients from the MARS endotype cohorts, 3 draws taken at an ICU-acquired infection, and 192 samples without a diagnostic label (Table S14a). The non-infected ICU controls were used only in the cross-fitted specificity analysis (Section 4.6) and never in feature selection or model fitting. The validation series has no equivalent arm.

The deposited characteristics of GSE65682 record admission diagnosis, age, sex, and 28-day mortality, but not organ-support status at the time of sampling. The fraction of the 242 sepsis patients who were mechanically ventilated or receiving vasopressors is therefore unknown, and the sepsis and non-infected groups could not be matched on illness severity. Both groups were ICU admissions under the same consortium protocol, drawn from the same units over the same period. The two sub-studies differ in their case and comparator definitions, and Section 2.2 reports the estimate within each. Formal severity matching would require clinical linkage that is not public (Section 3.7).

The validation cohort was GSE57065 [58], an independent septic-shock cohort profiled on the Affymetrix HG-U133 Plus 2.0 array (Affymetrix, Santa Clara, CA, USA; platform GPL570). Its series matrix, also obtained from GEO, is RMA-normalized and covers the full 54,675-probe array without submitter pre-filtering. The series contains 107 samples from 28 septic-shock patients, each sampled up to three times (at shock onset and 24 and 48 h later), and 25 healthy volunteers sampled once. The analytic cohort of 53 subjects comprised the first draw of each patient, taken within 30 min of shock onset, and all 25 healthy volunteers, so that each subject contributed exactly one sample.

Cohort characteristics and between-group comparisons are given in Table 1. Both series are public and de-identified, and the analysis used the deposited series matrices rather than raw CEL or FASTQ files.

### 4.2. Probe-to-Gene Mapping

Platform annotation tables for GPL13667 and GPL570 were obtained from GEO. They map probe identifiers to gene symbols in 47,320 rows for GPL13667 (19,040 unique genes) and 42,986 rows for GPL570 (21,655 unique genes). Probes annotated to more than one gene and probes without a resolvable symbol were dropped. Where several probes mapped to the same gene, the probe with the highest mean expression was retained, a common collapse rule for Affymetrix arrays. The resulting discovery and validation gene-level matrices contained 11,222 and 21,655 genes.

### 4.3. Cross-Platform Feature-Availability Audit

Before any differential expression or signature selection, each of the 11,222 discovery genes was checked for a matching gene in the validation gene-level matrix. Of the 11,222 genes, 9983 (89.0%) were transportable, that is, measurable on both platforms. The remaining 1239 (11.0%) were absent from the validation platform’s usable gene set and were excluded from all downstream candidate pools, including those from which the random panels of Section 4.8 were drawn. Selected and random panels therefore came from the same gene universe.

### 4.4. Testing Dataset, Cohort, and Platform Structure

The discovery and validation samples, restricted to the transportable genes and further to the 2000 genes with the highest pooled variance, were jointly decomposed by principal-component analysis with 10 components retained. Group structure in this ordination was tested by PERMANOVA on Euclidean distances [59], with 999 label permutations giving an *R*^2^ and an empirical *p*-value.

Dataset, cohort, hospital, platform, and sample composition are mutually confounded across the discovery/validation boundary. Three contrasts were therefore estimated: the pooled contrast between the two series, which captures all of these factors together; the dataset contrast among healthy samples only, where diagnosis is constant because healthy donors appear in both series; and the diagnosis contrast within the discovery cohort only, where platform, cohort, and hospital are constant. A two-factor sequential decomposition of the pooled sepsis and healthy samples was also computed in both entry orders, and the difference between the two orders indicates the degree of confounding.

Discovery and validation were not batch-corrected against each other, and neither ComBat adjustment [34] nor joint cross-platform normalization [35] was applied. Two standardization procedures were used. For modeling, gene-wise means and scales were estimated on the discovery training data alone and applied unchanged to the validation and specificity cohorts at scoring time. For the descriptive ordination of Section 2.5, which uses no labels, a single standardization was fitted on the combined matrix.

### 4.5. Differential Expression and Nested Cross-Validation Signature Selection

For each of the 9983 transportable genes, the sepsis (*n* = 242) and healthy (*n* = 42) samples of the discovery cohort were compared with Welch’s two-sample *t*-test, and *p*-values were adjusted with the Benjamini–Hochberg false discovery rate (FDR) procedure [41]. In total, 8319 genes (83.3%) had an FDR-adjusted *p*-value below 0.05. The 1508 of these with an absolute log_2_ fold change of at least 1 formed the query set for over-representation analysis (Section 4.12).

Signatures were selected by nested cross-validation with 5 outer and 5 inner folds. The candidate-gene filter was re-derived inside every outer training fold, so that no feature-selection step saw its own test fold [36,37]. Within each outer training fold, a candidate pool of the 300 genes with the largest absolute Welch *t* statistic was first derived from the training partition alone. An inner 5-fold grid search over the regularization parameter *C* ∈ {0.003, 0.01, 0.03, 0.1, 0.3, 1.0} of L1-penalized logistic regression [56] then selected the value that maximized mean inner AUROC. The model was finally refitted on the training partition and scored on the held-out test fold. Outer-fold AUROCs were 1.000, 1.000, 1.000, 0.984, and 0.997, and the pooled out-of-fold AUROC was 0.999. The candidate pool was reasonably stable across outer folds (mean pairwise Jaccard overlap 0.76).

The final model, referred to as the locked model and used in all later evaluations, was produced by a single fit of the same two-stage procedure (the top-300 |*t*| filter followed by L1-penalized logistic regression at the modal outer-fold *C* = 0.1) on all 284 discovery sepsis and healthy samples. This fit fixed the gene list, the feature order, the scaler mean and scale, the coefficients, and the intercept, and none of these was refitted, re-tuned, or re-thresholded on any later cohort. It retained 10 genes with non-zero coefficients: *S100A8* (*β* = +0.783), *NELL2* (−0.335), *P2RY10* (−0.259), *FAM43A* (−0.237), *SBK1* (−0.179), *FBXL16* (−0.144), *FCER1A* (−0.133), *NMT2* (−0.065), *KLRB1* (−0.028), and *SLC7A6* (−0.011). The panel size was not fixed in advance, since the ten genes are those whose L1 coefficients remained non-zero at the modal *C*, and Section 2.3 shows how much this number varies under resampling.

### 4.6. Cross-Fitted Specificity Analysis Against Non-Infected ICU Controls

Critical illness alone reprograms the leukocyte transcriptome, largely through a shift in circulating cell composition [60,61], so sepsis is readily separated from healthy outpatient volunteers regardless of infection status. The specificity analysis therefore compared sepsis cases with non-infected ICU controls, to assess whether the signature detects infection rather than illness.

Scoring the locked model against the 75 non-infected ICU controls would hold out the entire negative class but reuse all 242 sepsis cases, each of which contributed to fitting the model, so that AUROC is partly in-sample. The primary estimate was therefore cross-fitted. The 242 sepsis cases and the 75 non-infected ICU controls were partitioned into the same five stratified folds. For each fold, the full selection-and-fit procedure of Section 4.5 was re-run from scratch on the training sepsis cases plus all 42 healthy controls, and the held-out sepsis cases and non-infected controls of that fold were scored by that model alone. Each of the 317 samples was thus scored exactly once, by a model that had not seen it. The healthy controls remained in training in every fold because they define the task for which the signature was selected, and splitting them would change the estimand.

Three summaries of the out-of-fold predictions were reported. The primary estimate was the mean of the per-fold AUROCs, with a *t* interval across folds. The second was the pooled AUROC after each fold’s scores had been converted to within-fold percentile ranks, which measures ranking alone. The third was the pooled AUROC of the raw probabilities, which is lower because each fold model is calibrated on its own training partition and between-fold offsets degrade the pooled ranking. The partly in-sample AUROC of the locked model was reported alongside, to quantify its optimism.

### 4.7. Uncertainty: Subject-Level Intervals and a Full-Pipeline Bootstrap

AUROC intervals were computed with the non-parametric subject-level variance of DeLong, DeLong, and Clarke-Pearson [43], using the midrank algorithm of Sun and Xu [62], and were cross-checked against a stratified subject-level percentile bootstrap. A Wilson score interval over the *n*_1_ × *n*_0_ = 700 case–control comparisons in the validation cohort, which treats them as independent Bernoulli trials [44], was not used. Each of the 53 subjects appears in many pairs, so the comparisons are not independent, the effective sample size is the number of subjects, and such an interval is too narrow.

At an AUROC of exactly 1.000, the DeLong variance is zero and the subject-level bootstrap is degenerate because resampling within a class cannot invert a between-class rank. No two-sided interval is then estimable, and a conservative one-sided lower bound was reported instead. With zero discordant pairs observed, the binomial rule of three [45] bounds the unobserved discordance rate at approximately 3/*m*, where *m* is the number of case–control comparisons that can be formed without reusing a subject, that is min (*n*_1_, *n*_0_) = 25. The raw score margin was reported alongside, to distinguish genuine separation from a numerical artifact. The discovery interval abuts the ceiling for a related reason: sepsis-versus-healthy separation is near-complete, so almost every resample reproduces it, and the width of the interval reflects a sampling distribution pressed against a boundary rather than the standard error of a well-estimated effect.

A percentile bootstrap of the frozen out-of-fold predictions (Table 4, first row) holds feature selection and hyperparameter tuning fixed across replicates and therefore excludes selection uncertainty. A second, full-pipeline bootstrap repeated the entire procedure. In each of 200 replicates, a stratified bootstrap sample of the 284 discovery subjects was drawn with replacement, the top-300 filter was re-derived, *C* was re-tuned by inner cross-validation, and the L1 model was refitted. Each replicate was evaluated on its out-of-bag subjects, roughly 36.8% of subjects per replicate, and scored on both external cohorts. Out-of-bag evaluation was used instead of nested cross-validation inside the bootstrap sample because sampling with replacement can place the same subject in both a training and a test fold and reinflate the optimism that the interval is meant to capture [40]. The re-selection frequency of each locked-signature gene across replicates was recorded as a direct measure of selection stability [42].

### 4.8. Random-Gene-Panel Benchmark

The null hypothesis of the benchmark was that a panel of *k* genes drawn uniformly at random, without replacement, from a stated candidate pool, and fitted by a stated modeling recipe on the 284 discovery sepsis and healthy samples, achieves a test-cohort AUROC no worse than the panel selected by the study’s procedure and fitted by the identical recipe. The benchmark thus compares gene sets with the modeling recipe held fixed. No label is permuted, so it does not test classification against chance, which is the role of the label-permutation test (Section 4.9).

Four design choices defined the benchmark. First, the modeling recipe was specified explicitly rather than taken from a software default. Two recipes were run, identically for selected and random panels: the primary specification, L1-penalized at the locked modal *C* = 0.1, which is the signature’s own recipe, and an unpenalized recipe reported as a specification check. Second, random panels have no locked coefficients, so they were compared with the selected panel refitted under each recipe, and the AUROC of the locked model was reported alongside. The difference between the two was 0.001 on the specificity task and zero on validation. Third, two candidate pools were used. The 9983-gene transportable pool tests whether the gene list outperforms an arbitrary measurable one, and the 300-gene top-|*t*| candidate pool tests the selection-matched question of whether, given the filter, the L1 step selected better than chance. Fourth, panel size was varied over *k* ∈ {5, 10, 20, 50}, and the selected panel was resized at every *k* by the same procedure, so that selected and random panels were always compared at equal size. For resizing, *C* was relaxed along a fine grid until at least *k* coefficients remained non-zero, and the *k* largest were taken.

For each cell, 2000 panels were drawn, and duplicate draws were counted (none occurred in any cell). The full panel list for the primary cell is given in the data workbook (File S3), with its SHA-256 checksum in Table S8, so that the Monte Carlo run can be verified independently. The empirical *p*-value is (1 + *r*)/(1 + 2000), where *r* counts the random panels whose AUROC was at least that of the selected panel. The 2000 draws are mutually independent, so each proportion also carries an exact Clopper–Pearson interval, which is valid here, unlike the pairwise-concordance interval discussed in Section 4.7. The width of these intervals is small relative to the distance between each reported proportion and 0.05, so 2000 draws are sufficient.

### 4.9. Label-Permutation Test

The label-permutation test assessed the null hypothesis that the discovery sepsis/healthy label vector is exchangeable with respect to the expression matrix. In each of 1000 permutations, the class labels of the 284 discovery samples were shuffled and the entire selection-and-fit procedure was re-run on the permuted labels: the top-300 |Welch *t*| filter was re-derived from the shuffled labels, and an L1-logistic model was fitted at the modal *C*. The resulting model was scored on the validation cohort and on the specificity cohort, both with their true labels. Re-deriving the filter inside each permutation makes this a test of the pipeline rather than of a fixed feature set, since holding the selected genes fixed would leak the original selection into the null. The empirical *p*-value followed the standard construction for classifier permutation testing [28].

A small *p*-value indicates that the pipeline extracts real label information. It does not indicate whether the particular genes chosen outperform an arbitrary panel, which is the question addressed in Section 4.8.

### 4.10. Performance Metrics Beyond AUROC

For every evaluation cohort, sensitivity, specificity, positive and negative predictive value, accuracy, balanced accuracy, F1, the area under the precision–recall curve (PR-AUC), Brier score, calibration intercept and slope, and the 2 × 2 confusion matrix were reported. They were computed at two thresholds: the model’s nominal 0.5, fixed a priori, and the Youden-optimal threshold, which is chosen on the evaluation data and is therefore optimistic and labeled as such. Predictive values depend on prevalence, and the prevalence in these cohorts reflects how the series were assembled rather than any clinical population, so they were reported both at the cohort prevalence and at a reference prevalence of 10%.

### 4.11. Demographic Sensitivity Analyses

Age and sex both shift the leukocyte transcriptome, and Table 1 shows that both are imbalanced in the healthy-control contrasts. Three analyses were therefore run on each contrast. The first measured incremental discrimination, using logistic models of the outcome on age and sex alone, on the signature score alone, and on all three, with a likelihood-ratio test for the score given demography. The second recomputed AUROC within sex strata and within age tertiles. The third used greedy 1:1 matching of controls to cases, exact on sex and within a 5-year age caliper, and recomputed AUROC in the matched subset. The specificity contrast used the cross-fitted scores of Section 4.6, not the partly in-sample ones.

### 4.12. Pathway and Gene Ontology Over-Representation Analysis

Gene sets from Molecular Signatures Database (MSigDB) Hallmark 2020 (50 terms) [49], KEGG 2021 Human (320 terms) [50], and GO Biological Process 2023 (5407 terms) [51] were downloaded once in text format from Enrichr [52], and no network access was used during the analysis.

The test was hypergeometric over-representation analysis (ORA) [48], with the 9983-gene transportable universe as the background. ORA differs from gene set enrichment analysis of ranked lists [63], which addresses a different question with a different input. ORA is also non-directional: a term can be over-represented because its genes are up-regulated, down-regulated, or a mixture, so these results do not support claims of pathway activation.

The multiple-testing family was defined in advance, independently of the data. Every term with at least one gene in the universe was tested and entered the correction, since dropping terms with low observed overlap before correction would make the family depend on the observed test statistic. For each query set, the family was the union of all testable terms across the three libraries, corrected once (5742 terms, with 35 untestable because no member gene lay in the universe). Per-library correction was retained as a secondary column. The overlap filter was applied afterwards, for display only, and affects no *p*-value. Enrichment results are hypothesis-generating associations and do not show that any pathway drives, causes, or mechanistically explains classifier performance.

### 4.13. Reproducibility and Software

A fixed random seed (42) was used throughout for data splitting, bootstrap resampling, random panel draws, and label permutations. The software was Python 3.13.12, pandas 2.2.3, numpy 2.4.2, scikit-learn 1.6.1, scipy 1.16.3, and statsmodels 0.14.6. Expression matrices were held in single precision (float32).

Given the seed and these versions, the following are expected to reproduce exactly: the cohort construction and all sample counts, the probe-to-gene collapse, the transportability audit, the differential-expression table, the nested cross-validation fold assignment, the locked gene list and its coefficients, and the sequence of random panels drawn (verifiable against the recorded SHA-256 checksum). The following may differ in the last reported digit across implementations of the basic linear algebra subprograms (BLAS) or across library versions because they aggregate many floating-point operations whose order is not guaranteed: AUROC values at the fourth decimal, bootstrap and permutation interval endpoints, and PERMANOVA pseudo-*F*. The empirical *p*-values are counts out of a fixed number of draws and are reproducible given the same draw sequence. All reported numbers were taken directly from the result files of the analysis code.

## 5. Conclusions

A label-permutation test and a random-panel benchmark answer different questions about a transcriptomic signature. The 10-gene sepsis signature examined here passed the first (*p* = 0.001) but not the second (*p* = 0.125), and its genes were re-selected in 76.5% to 8.0% of bootstrap resamples. Its discrimination is therefore real, but the particular genes it names are close to arbitrary. Both controls are inexpensive, requiring a few thousand refits of a small logistic model, and reporting them together with re-selection frequencies would make signature studies easier to interpret. Whether such controls should be required for all transcriptomic classifiers would need evaluation across further diseases, cohorts, and model classes.

The comparator determines what a sepsis signature measures. Against healthy volunteers, the signature achieved 0.999 to 1.000, on a contrast where age and sex alone reach 0.884. Against non-infected ICU patients, the same selection procedure achieved a cross-fitted 0.730, on a contrast where age and sex reach 0.542 and where matching changed nothing, and at its nominal operating point, it called 70 of the 75 non-infected ICU patients septic. Until the specificity estimate is externally confirmed, a signature of this kind is better described as detecting the magnitude of a generic critical-illness response than as detecting infection, and it can at most complement clinical Sepsis-3 assessment [1]. Clinical translation requires a third, independent cohort with a non-infected critical-illness arm, matched or adjusted for age and sex, with recorded severity, on a platform different from both used here, followed by the analytical and clinical validation required for in vitro diagnostics. More sepsis-versus-healthy samples would narrow the intervals on a saturated benchmark without making the comparison more informative.

## Figures and Tables

**Table 1:** Cohort characteristics for the discovery and validation series, with between-group comparisons.

Cohort	Group	*n*	Age, y (Mean ± SD)	Female, %	Comparison with the Sepsis Group of the Same Cohort
Discovery (GSE65682)	Sepsis	242	61.3 ± 15.2	40.5	reference
Discovery (GSE65682)	Non-infected ICU	75	59.1 ± 15.5	34.7	age *t* = 1.085, *p* = 0.280; sex *χ*^2^ = 0.59, *p* = 0.442
Discovery (GSE65682)	Healthy	42	46.2 ± 19.6	42.9	age *t* = 4.756, *p* < 0.001; sex *χ*^2^ = 0.01, *p* = 0.907
Validation (GSE57065)	Sepsis	28	62.7 ± 15.8	32.1	reference
Validation (GSE57065)	Healthy	25	44.5 ± 11.2	80.0	age *t* = 4.893, *p* < 0.001; sex *χ*^2^ = 10.35, *p* = 0.001

Age is compared by Welch’s two-sample *t*-test and sex by the chi-square test. Sepsis patients are markedly older than healthy volunteers in both cohorts, and the validation cohort is also imbalanced by sex. The conventional benchmark is therefore demographically confounded. The clinically relevant contrast, sepsis versus non-infected ICU controls, is not imbalanced on either variable. Section 2.2 quantifies the consequences. SD, standard deviation.

**Table 2:** What each evaluation tests, and what it found. Every row uses the same locked selection procedure.

Evaluation	What the Null Says	Statistic	Result
Label permutation, validation	labels carry no information	AUROC vs. 1000 permuted-label refits	1.000 vs. null mean 0.514; *p* = 0.001
Label permutation, specificity	labels carry no information	AUROC vs. 1000 permuted-label refits	0.733 vs. null mean 0.515; *p* = 0.004
Random panels, validation	an arbitrary 10-gene panel does as well	AUROC vs. 2000 random panels	1.000 vs. null mean 0.960; *p* = 0.125
Random panels, specificity	an arbitrary 10-gene panel does as well	AUROC vs. 2000 random panels	0.734 vs. null mean 0.659; *p* = 0.071
Random panels, selection-matched pool	the L1 step chose no better than chance among the 300 filtered candidates	AUROC vs. 2000 random panels	1.000 vs. null mean 0.999; *p* = 0.676

Full results are given in Tables S7 and S13 of File S2. The empirical *p*-value is (1 + *r*)/(1 + *B*), where *r* is the number of null values at least as large as the observed one and *B* is the number of draws. The first two rows and the last three address different questions. AUROC, area under the receiver operating characteristic curve.

**Table 3:** Performance beyond AUROC at the nominal threshold of 0.5, for the external validation and the cross-fitted specificity evaluations.

Metric	Validation (Locked), Threshold 0.5	Specificity (Cross-Fitted), Threshold 0.5
Sensitivity	0.929	0.988
Specificity	1.000	0.067
Balanced accuracy	0.964	0.527
F1	0.963	0.868
PPV at cohort prevalence	1.000	0.774
NPV at cohort prevalence	0.926	0.625
PR-AUC (baseline = prevalence)	1.000 (0.528)	0.838 (0.763)
Brier score	0.054	0.182
Calibration intercept, slope	not estimable under complete separation	−0.689, 0.240

Full results are given in Table S11a,b. Predictive values are also reported there at a 10% reference prevalence because cohort prevalence here is an artifact of series assembly. Youden-optimal thresholds are given in the same tables and are optimistic, having been chosen on the evaluation data. AUROC, area under the receiver operating characteristic curve; PPV, positive predictive value; NPV, negative predictive value; PR-AUC, area under the precision–recall curve.

**Table 4:** Discrimination across every evaluation, with the interval method stated for each.

Evaluation	*n*	AUROC	95% CI	Interval Method
Discovery, nested CV, pooled out-of-fold	284	0.999	0.996–1.000	percentile bootstrap on frozen predictions; excludes selection uncertainty
Discovery, full-pipeline bootstrap, out-of-bag	284	0.999	0.991–1.000	200 replicates, selection and tuning repeated in each
Validation, external, locked model	53	1.000	not estimable; ≥0.887 one-sided	DeLong variance is zero at complete separation; rule-of-three lower bound
Specificity, cross-fitted (primary)	317	0.730	0.642–0.818	*t* interval across 5 fold estimates
Specificity, cross-fitted, rank-pooled	317	0.730	0.660–0.789	DeLong, subject-level
Specificity, age- and sex-matched	150	0.733	0.646–0.805	DeLong, subject-level
Specificity, locked model, partly in-sample	317	0.733	0.664–0.792	DeLong; retained only to quantify optimism

No two-sided interval is estimable for the validation row (Section 4.7). A Wilson interval over the 700 case–control pairs is not reported because the pairs share subjects and are not independent. AUROC, area under the receiver operating characteristic curve; CI, confidence interval; CV, cross-validation.

## Data Availability

All data analyzed in this study are publicly available and were not generated by the authors. Gene-expression series matrices were obtained from the NCBI Gene Expression Omnibus (GEO): GSE65682 (https://ftp.ncbi.nlm.nih.gov/geo/series/GSE65nnn/GSE65682/matrix/, accessed on 1 August 2026) and GSE57065 (https://ftp.ncbi.nlm.nih.gov/geo/series/GSE57nnn/GSE57065/matrix/, accessed on 1 August 2026). Platform annotation tables for GPL13667 (Affymetrix HG-U219) and GPL570 (Affymetrix HG-U133 Plus 2.0) were obtained from https://www.ncbi.nlm.nih.gov/geo/query/acc.cgi (accessed on 1 August 2026). The MSigDB Hallmark 2020, KEGG 2021 Human, and GO Biological Process 2023 gene-set libraries were obtained from Enrichr (https://maayanlab.cloud/Enrichr/#libraries, accessed on 1 August 2026). GEO data are in the public domain, MSigDB and the Gene Ontology are distributed under the Creative Commons Attribution 4.0 license, and KEGG is distributed under its academic-use terms. The analysis code and the list of 2000 random gene panels underlying the primary benchmark, with its SHA-256 checksum, are provided in the Supplementary Materials (Files S1 and S3).

## Supplementary Materials

The following supporting information can be downloaded at: https://www.mdpi.com/article/10.3390/ijms27209273/s1.

## Abbreviations

The following abbreviations are used in this manuscript:

AUROC	area under the receiver operating characteristic curve
BLAS	basic linear algebra subprograms
CI	confidence interval
FDR	false discovery rate
GEO	Gene Expression Omnibus
GO	Gene Ontology
ICU	intensive care unit
KEGG	Kyoto Encyclopedia of Genes and Genomes
MAQC-II	second phase of the MicroArray Quality Control project
MARS	Molecular Diagnosis and Risk Stratification of Sepsis
MSigDB	Molecular Signatures Database
NCBI	National Center for Biotechnology Information
NPV	negative predictive value
ORA	over-representation analysis
PC	principal component
PERMANOVA	permutational multivariate analysis of variance
PPV	positive predictive value
PR-AUC	area under the precision–recall curve
RMA	robust multi-array average
RNA-seq	RNA sequencing
SD	standard deviation
