# Supplementary material for: What a Near-Perfect AUROC Does Not Show: Random-Panel and Label-Permutation Controls for a Sepsis Blood-Transcriptome Signature
Source: Int J Mol Sci. 2026 Oct 19;27(20):9273. doi: 10.3390/ijms27209273 (PMC13704978; doi:10.3390/ijms27209273)
Supplement: Supplementary file 1 [file ijms-27-09273-s001.zip › Supplement_S1_Reproducible_Analysis_Code_CEL_R2.pdf]

What a Near-Perfect AUROC Does Not Show: Random-Panel and Label-Permutation Controls for a Sepsis Blood-Transcriptome Signature

Supplementary File S1: Reproducible Analysis Code

This document contains the complete, formatted source code accompanying the article above, organized by file. Each listing is reproduced verbatim from the reproducible analysis pipeline; line numbers are added for reference only.

Contents

1 `src/config.py` 2

2 `src/formatting.py` 3

3 `src/plotting.py` 3

4 `src/scoring.py` 5

5 `src/auroc_inference.py` 6

6 `src/parse_platforms.py` 8

7 `src/build_discovery.py` 9

8 `src/build_validation.py` 12

9 `src/validation_metadata_extra.py` 14

10 `src/platform_audit.py` 15

11 `src/transport_audit.py` 16

12 `src/quantify_batch_platform.py` 17

13 `src/differential_expression.py` 22

14 `src/nested_cv_signature.py` 23

15 `src/specificity_check.py` 28

16 `src/specificity_crossfit.py` 29

17 `src/batch_source_structure.py` 34

18 `src/external_validation.py` 39

19 `src/random_panel_benchmark.py` 43

20 `src/label_permutation_test.py` 52

21 `src/pipeline_bootstrap.py` 55

22 `src/clinical_metrics.py` 60

23 `src/demographic_sensitivity.py` 64

24 `src/enrichment_analysis.py` 69

25 `src/table1_cohorts.py` 72

26 `src/make_summary_figures.py` **75**

27 `src/make_figures_3_4.py` **76**

28 `src/fig_design_schematic.py` **80**

29 `scripts/run_all.py` **85**

1 src/config.py

```python
from __future__ import annotations

import logging
from pathlib import Path

logger = logging.getLogger(__name__)

PROJECT_ROOT = Path(__file__).resolve().parents[1]
RAW_DATA_DIR = PROJECT_ROOT / "data" / "raw"
INTERIM_DATA_DIR = PROJECT_ROOT / "data" / "interim"
PROCESSED_DATA_DIR = PROJECT_ROOT / "data" / "processed"
RESULTS_DIR = PROJECT_ROOT / "results"
FIGURES_DIR = RESULTS_DIR / "figures"
TABLES_DIR = RESULTS_DIR / "tables"
METRICS_DIR = RESULTS_DIR / "metrics"
MODELS_DIR = RESULTS_DIR / "models"

for _directory in (
    INTERIM_DATA_DIR,
    PROCESSED_DATA_DIR,
    FIGURES_DIR,
    TABLES_DIR,
    METRICS_DIR,
    MODELS_DIR,
):
    _directory.mkdir(parents=True, exist_ok=True)

RANDOM_SEED = 42

DISCOVERY_ACCESSION = "GSE65682"
DISCOVERY_PLATFORM = "GPL13667"
DISCOVERY_MATRIX = RAW_DATA_DIR / "GSE65682_series_matrix.txt.gz"
DISCOVERY_PLATFORM_ANNOTATION = RAW_DATA_DIR / "GPL13667_platform.txt"

DISCOVERY_INFECTED_LABELS = {
    "pneumonia diagnoses": {"cap", "hap"},
    "abdominal_sepsis_and_controls": {"abdo_s"},
}
DISCOVERY_NONINFECTED_LABELS = {
    "pneumonia diagnoses": {"no-cap"},
    "abdominal_sepsis_and_controls": {"ctrl_GI"},
}

VALIDATION_ACCESSION = "GSE57065"
VALIDATION_PLATFORM = "GPL570"
VALIDATION_MATRIX = RAW_DATA_DIR / "GSE57065_series_matrix.txt.gz"
VALIDATION_PLATFORM_ANNOTATION = RAW_DATA_DIR / "GPL570_platform.txt"
VALIDATION_TIMEPOINT = "H00"

HALLMARK_GENE_SETS = RAW_DATA_DIR / "MSigDB_Hallmark_2020.gmt"
KEGG_GENE_SETS = RAW_DATA_DIR / "KEGG_2021_Human.gmt"
GO_BIOLOGICAL_PROCESS_GENE_SETS = (
    RAW_DATA_DIR / "GO_Biological_Process_2023.gmt"
)

OUTER_FOLDS = 5
INNER_FOLDS = 5
BOOTSTRAP_REPLICATES = 1000
RANDOM_PANEL_DRAWS = 2000
LABEL_PERMUTATIONS = 1000
PIPELINE_BOOTSTRAP_REPLICATES = 200

PANEL_SIZES = (5, 10, 20, 50)


def configure_logging() -> None:
    logging.basicConfig(level=logging.INFO, format="%(message)s")
```

2 src/formatting.py

```
from __future__ import annotations

import math

MINUS = "-"

DECIMALS: dict[str, int] = {
    "auroc": 3,
    "auprc": 3,
    "c_index": 3,
    "r2": 3,
    "auc_diff": 4,
    "percent": 1,
    "sensitivity": 1,
    "specificity": 1,
    "accuracy": 1,
    "coef": 3,
    "beta": 3,
    "log_hr": 3,
    "shap": 3,
    "hr": 2,
    "or": 2,
    "rr": 2,
    "pvalue": 3,
    "brier": 3,
    "calibration_slope": 3,
    "clinical": 1,
}


def format_count(count: float | int) -> str:
    count = int(round(count))
    return f"{count:,}" if abs(count) >= 10000 else str(count)


def format_number(
    value: float, kind: str = "coef", *, signed: bool = False
) -> str:
    if value is None or (isinstance(value, float) and math.isnan(value)):
        return "NA"
    decimals = DECIMALS.get(kind, 3)
    text = f"{value:+.{decimals}f}" if signed else f"{value:.{decimals}f}"
    return text.replace("-", MINUS)


def format_percent(
    value: float, *, already_fraction: bool = False, signed: bool = False
) -> str:
    percent = value * 100 if already_fraction else value
    return format_number(percent, "percent", signed=signed) + "%"


def format_p_value(p_value: float) -> str:
    if p_value is None or (isinstance(p_value, float) and math.isnan(p_value)):
        return "NA"
    if p_value < 0.001:
        return "p < 0.001"
    return f"p = {p_value:.3f}"
```

3 src/plotting.py

```
from __future__ import annotations

import json
from pathlib import Path

import matplotlib as mpl
import numpy as np

PALETTE: dict[str, str] = {
```

```python
    "blue": "#0072B2",
    "orange": "#E69F00",
    "green": "#009E73",
    "pink": "#CC79A7",
    "red": "#D55E00",
    "sky": "#56B4E9",
    "yellow": "#F0E442",
    "gray": "#999999",
}

FULL_WIDTH_INCHES = 5.5


def apply_style() -> None:
    mpl.rcParams.update(
        {
            "axes.unicode_minus": True,
            "font.family": "serif",
            "font.size": 10,
            "figure.dpi": 300,
            "savefig.dpi": 300,
            "mathtext.default": "it",
            "axes.grid": False,
            "axes.spines.top": False,
            "axes.spines.right": False,
            "legend.frameon": False,
            "axes.titlesize": 10,
        }
    )


def label_panel(ax, letter: str) -> None:
    ax.text(
        0.02,
        0.98,
        f"({letter.lower()})",
        transform=ax.transAxes,
        fontweight="bold",
        va="top",
        ha="left",
    )


def collect_handles(fig) -> dict:
    handles: list[dict] = []
    for ax in fig.get_axes():
        for line in ax.get_lines():
            lbl = line.get_label()
            if lbl and not lbl.startswith("_"):
                handles.append(
                    {
                        "type": "line",
                        "label": lbl,
                        "color": mpl.colors.to_hex(line.get_color()),
                        "linestyle": str(line.get_linestyle()),
                    }
                )
        for patch in ax.patches:
            lbl = patch.get_label()
            if lbl and not lbl.startswith("_"):
                handles.append(
                    {
                        "type": "patch",
                        "label": lbl,
                        "color": mpl.colors.to_hex(patch.get_facecolor()),
                    }
                )
        for coll in ax.collections:
            lbl = coll.get_label()
            if lbl and not lbl.startswith("_"):
                handles.append({"type": "collection", "label": lbl})
        seen = {h["label"] for h in handles}
        for container in getattr(ax, "containers", []):
            lbl = container.get_label()
            if not lbl or lbl.startswith("_") or lbl in seen:
                continue
```

```python
            color = None
            for child in container.get_children():
                if hasattr(child, "get_facecolor"):
                    face = np.asarray(child.get_facecolor())
                    if face.ndim == 2:
                        if len(face) == 0:
                            continue
                        face = face[0]
                    color = mpl.colors.to_hex(face)
                    break
            entry = {"type": "container", "label": lbl}
            if color:
                entry["color"] = color
            handles.append(entry)
            seen.add(lbl)
    return {"handles": handles, "n_axes": len(fig.get_axes())}


def save_figure(
    fig, outdir: Path | str, name: str, *, tight: bool = True
) -> dict[str, Path]:
    outdir = Path(outdir)
    outdir.mkdir(parents=True, exist_ok=True)
    kw = {"bbox_inches": "tight"} if tight else {}
    png = outdir / f"{name}.png"
    pdf = outdir / f"{name}.pdf"
    fig.savefig(png, dpi=300, **kw)
    fig.savefig(pdf, **kw)
    sidecar = outdir / f"{name}.colors.json"
    sidecar.write_text(
        json.dumps(collect_handles(fig), indent=2), encoding="utf-8"
    )
    return {"png": png, "pdf": pdf, "sidecar": sidecar}
```

4 src/scoring.py

```python
from __future__ import annotations

import json

import numpy as np
import pandas as pd

from config import PROCESSED_DATA_DIR

LOCKED_MODEL_FILE = PROCESSED_DATA_DIR / "locked_model.json"


def load_locked_model() -> dict:
    with open(LOCKED_MODEL_FILE, encoding="utf-8") as model_file:
        return json.load(model_file)


def predict_probability(
    expression: pd.DataFrame, locked_model: dict
) -> np.ndarray:
    gene_order = locked_model["gene_names_order"]
    missing_genes = [
        gene for gene in gene_order if gene not in expression.columns
    ]
    if missing_genes:
        raise ValueError(
            f"{len(missing_genes)} genes missing from the scoring frame, "
            f"e.g. {missing_genes[:5]}"
        )
    features = expression[gene_order].values.astype(np.float64)
    selected = features[:, np.array(locked_model["candidate_idx_final"])]
    standardized = (selected - np.array(locked_model["scaler_mean"])) / (
        np.array(locked_model["scaler_scale"])
    )
    logits = (
        standardized @ np.array(locked_model["model_coef"])
        + locked_model["model_intercept"]
```

```python
    )
    return 1.0 / (1.0 + np.exp(-logits))
```

5 src/auroc_inference.py

```python
from __future__ import annotations

import numpy as np
from scipy import stats
from sklearn.metrics import roc_auc_score


def auroc_report(
    labels: np.ndarray,
    scores: np.ndarray,
    bootstrap_replicates: int = 2000,
    seed: int = 42,
    confidence: float = 0.95,
) -> dict:
    labels = np.asarray(labels).astype(int)
    scores = np.asarray(scores, dtype=float)
    delong = delong_interval(labels, scores, confidence=confidence)
    bootstrap = bootstrap_interval(
        labels,
        scores,
        replicates=bootstrap_replicates,
        seed=seed,
        confidence=confidence,
    )
    n_cases, n_controls = int((labels == 1).sum()), int((labels == 0).sum())
    ceiling_bound = rule_of_three_lower_bound(
        n_cases, n_controls, confidence=confidence
    )
    highest_control = float(scores[labels == 0].max())
    lowest_case = float(scores[labels == 1].min())
    report = {
        "auroc": float(roc_auc_score(labels, scores)),
        "n_case": n_cases,
        "n_control": n_controls,
        "delong": delong,
        "bootstrap": bootstrap,
        "rule_of_three": ceiling_bound,
        "score_margin": float(lowest_case - highest_control),
        "max_control_score": highest_control,
        "min_case_score": lowest_case,
    }
    if delong["degenerate"]:
        report["reported_ci_method"] = (
            "one-sided conservative lower bound (rule of three)"
        )
        report["reported_ci_lo"] = ceiling_bound["auroc_lower_bound"]
        report["reported_ci_hi"] = None
    else:
        report["reported_ci_method"] = "DeLong (subject-level)"
        report["reported_ci_lo"] = delong["ci_lo"]
        report["reported_ci_hi"] = delong["ci_hi"]
    return report


def delong_interval(
    labels: np.ndarray, scores: np.ndarray, confidence: float = 0.95
) -> dict:
    auroc, variance = delong_auroc_variance(labels, scores)
    standard_error = float(np.sqrt(variance))
    z_value = float(stats.norm.ppf(0.5 + confidence / 2))
    result = {
        "auroc": auroc,
        "se": standard_error,
        "method": "DeLong (subject-level)",
        "n_case": int(np.sum(np.asarray(labels) == 1)),
        "n_control": int(np.sum(np.asarray(labels) == 0)),
    }
    if standard_error == 0.0 or auroc >= 1.0 or auroc <= 0.0:
```

```python
        result.update(
            {
                "degenerate": True,
                "ci_lo": None,
                "ci_hi": None,
                "note": "variance is exactly zero at complete separation; "
                "no two-sided DeLong interval is estimable",
            }
        )
        return result
    logit = np.log(auroc / (1 - auroc))
    logit_error = standard_error / (auroc * (1 - auroc))
    lower = 1 / (1 + np.exp(-(logit - z_value * logit_error)))
    upper = 1 / (1 + np.exp(-(logit + z_value * logit_error)))
    result.update(
        {"degenerate": False, "ci_lo": float(lower), "ci_hi": float(upper)}
    )
    return result


def delong_auroc_variance(
    labels: np.ndarray, scores: np.ndarray
) -> tuple[float, float]:
    labels = np.asarray(labels).astype(int)
    scores = np.asarray(scores, dtype=float)
    case_scores = scores[labels == 1]
    control_scores = scores[labels == 0]
    n_cases, n_controls = len(case_scores), len(control_scores)
    if n_cases == 0 or n_controls == 0:
        raise ValueError("DeLong needs at least one case and one control")

    case_ranks = _midranks(case_scores)
    control_ranks = _midranks(control_scores)
    pooled_ranks = _midranks(np.concatenate([case_scores, control_scores]))
    pooled_case_ranks = pooled_ranks[:n_cases]
    pooled_control_ranks = pooled_ranks[n_cases:]

    auroc = (
        pooled_case_ranks.sum() / n_cases - (n_cases + 1) / 2.0
    ) / n_controls
    case_placements = (pooled_case_ranks - case_ranks) / n_controls
    control_placements = 1.0 - (pooled_control_ranks - control_ranks) / n_cases
    case_variance = np.var(case_placements, ddof=1) if n_cases > 1 else 0.0
    control_variance = (
        np.var(control_placements, ddof=1) if n_controls > 1 else 0.0
    )
    variance = case_variance / n_cases + control_variance / n_controls
    return float(auroc), float(variance)


def _midranks(values: np.ndarray) -> np.ndarray:
    order = np.argsort(values)
    sorted_values = values[order]
    count = len(values)
    sorted_ranks = np.empty(count, dtype=float)
    start = 0
    while start < count:
        end = start
        while (
            end < count - 1 and sorted_values[end + 1] == sorted_values[start]
        ):
            end += 1
        sorted_ranks[start : end + 1] = 0.5 * (start + end) + 1
        start = end + 1
    ranks = np.empty(count, dtype=float)
    ranks[order] = sorted_ranks
    return ranks


def bootstrap_interval(
    labels: np.ndarray,
    scores: np.ndarray,
    replicates: int = 2000,
    seed: int = 42,
    confidence: float = 0.95,
) -> dict:
```

```
    labels = np.asarray(labels).astype(int)
    scores = np.asarray(scores, dtype=float)
    case_rows = np.where(labels == 1)[0]
    control_rows = np.where(labels == 0)[0]
    random_generator = np.random.default_rng(seed)
    resampled_aurocs = np.empty(replicates)
    for replicate in range(replicates):
        rows = np.concatenate(
            [
                random_generator.choice(
                    case_rows, size=len(case_rows), replace=True
                ),
                random_generator.choice(
                    control_rows, size=len(control_rows), replace=True
                ),
            ]
        )
        resampled_aurocs[replicate] = roc_auc_score(labels[rows], scores[rows])
    alpha = (1 - confidence) / 2
    lower, upper = np.percentile(
        resampled_aurocs, [100 * alpha, 100 * (1 - alpha)]
    )
    return {
        "auroc": float(roc_auc_score(labels, scores)),
        "ci_lo": float(lower),
        "ci_hi": float(upper),
        "n_boot": int(replicates),
        "method": "stratified subject-level bootstrap",
        "degenerate": bool(lower == upper),
    }


def rule_of_three_lower_bound(
    n_cases: int, n_controls: int, confidence: float = 0.95
) -> dict:
    independent_pairs = int(min(n_cases, n_controls))
    if independent_pairs < 1:
        raise ValueError("need at least one case and one control")
    max_discordance = 1 - (1 - confidence) ** (1 / independent_pairs)
    return {
        "m_independent_pairs": independent_pairs,
        "max_discordance_rate": float(max_discordance),
        "auroc_lower_bound": float(1 - max_discordance),
        "conf": confidence,
        "method": "rule of three on independent case-control comparisons",
    }
```

6 src/parse_platforms.py

```
from __future__ import annotations

import logging
from pathlib import Path

import pandas as pd

from config import (
    DISCOVERY_PLATFORM_ANNOTATION,
    INTERIM_DATA_DIR,
    VALIDATION_PLATFORM_ANNOTATION,
    configure_logging,
)

logger = logging.getLogger(__name__)

UNUSABLE_SYMBOLS = ["", "nan", "---", "NA"]


def main() -> None:
    configure_logging()
    parse_platform(
        DISCOVERY_PLATFORM_ANNOTATION,
        "ID",
```

```python
        "Gene Symbol",
        "GPL13667_probe2gene.csv",
    )
    parse_platform(
        VALIDATION_PLATFORM_ANNOTATION,
        "ID",
        "Gene Symbol",
        "GPL570_probe2gene.csv",
    )


def parse_platform(
    annotation_path: Path,
    probe_column: str,
    symbol_column: str,
    output_name: str,
) -> pd.DataFrame:
    probe_map = pd.read_csv(
        annotation_path,
        sep="\t",
        skiprows=_table_start_line(annotation_path),
        usecols=[probe_column, symbol_column],
        dtype=str,
        engine="python",
        on_bad_lines="skip",
    )
    probe_map = probe_map[
        probe_map[probe_column] != "!platform_table_end"
    ].copy()
    probe_map = probe_map.rename(
        columns={probe_column: "probe_id", symbol_column: "gene_symbol"}
    )
    probe_map["gene_symbol"] = probe_map["gene_symbol"].astype(str).str.strip()
    unusable = probe_map["gene_symbol"].isin(UNUSABLE_SYMBOLS) | probe_map[
        "gene_symbol"
    ].str.contains("///")
    probe_map = probe_map.loc[
        ~unusable, ["probe_id", "gene_symbol"]
    ].drop_duplicates()
    output_path = INTERIM_DATA_DIR / output_name
    probe_map.to_csv(output_path, index=False)
    logger.info(
        "%s: %d probe->gene rows, %d unique genes -> %s",
        output_name,
        len(probe_map),
        probe_map["gene_symbol"].nunique(),
        output_path,
    )
    return probe_map


def _table_start_line(annotation_path: Path) -> int:
    with open(annotation_path, encoding="utf-8", errors="replace") as lines:
        for line_number, line in enumerate(lines):
            if line.startswith("!platform_table_begin"):
                return line_number + 1
    raise ValueError(f"!platform_table_begin not found in {annotation_path}")


if __name__ == "__main__":
    main()
```

7 src/build_discovery.py

```python
from __future__ import annotations

import gzip
import logging
import re
from pathlib import Path

import numpy as np
import pandas as pd
```

```python

from config import (
    DISCOVERY_INFECTED_LABELS,
    DISCOVERY_MATRIX,
    DISCOVERY_NONINFECTED_LABELS,
    INTERIM_DATA_DIR,
    PROCESSED_DATA_DIR,
    configure_logging,
)

logger = logging.getLogger(__name__)


def main() -> None:
    configure_logging()
    metadata, probe_expression = read_series_matrix(DISCOVERY_MATRIX)
    logger.info(
        "parsed %s: %d samples, %d probes",
        DISCOVERY_MATRIX.name,
        metadata.shape[0],
        probe_expression.shape[0],
    )
    labeled = label_samples(metadata)
    labeled.to_csv(
        INTERIM_DATA_DIR / "discovery_sample_metadata.csv", index=False
    )
    gene_expression = collapse_to_genes(
        probe_expression, labeled["geo_accession"].tolist()
    )
    gene_expression.to_csv(
        PROCESSED_DATA_DIR / "discovery_expr_gene.csv.gz", compression="gzip"
    )
    labeled.set_index("geo_accession").to_csv(
        PROCESSED_DATA_DIR / "discovery_labels.csv"
    )


def read_series_matrix(
    matrix_path: Path,
) -> tuple[pd.DataFrame, pd.DataFrame]:
    sample_fields: dict[str, list[str]] = {}
    characteristic_rows: list[list[str]] = []
    expression_rows: list[list[str]] = []
    header = None
    in_table = False
    with gzip.open(
        matrix_path, "rt", encoding="utf-8", errors="replace"
    ) as matrix:
        for raw_line in matrix:
            line = raw_line.rstrip("\n")
            if line.startswith("!series_matrix_table_begin"):
                in_table = True
                continue
            if line.startswith("!series_matrix_table_end"):
                in_table = False
                continue
            if in_table:
                if header is None:
                    header = [cell.strip('"') for cell in line.split("\t")]
                else:
                    expression_rows.append(line.split("\t"))
                continue
            if line.startswith("!Sample_characteristics_ch1"):
                characteristic_rows.append(_unquoted_values(line))
            elif line.startswith("!Sample_"):
                sample_fields[line.split("\t")[0]] = _unquoted_values(line)

    metadata = _metadata_frame(sample_fields, characteristic_rows)
    return metadata, _expression_frame(expression_rows, header)


def _unquoted_values(line: str) -> list[str]:
    return [value.strip('"') for value in line.split("\t")[1:]]


def _metadata_frame(
```

```python
    sample_fields: dict[str, list[str]],
    characteristic_rows: list[list[str]],
) -> pd.DataFrame:
    sample_count = len(sample_fields["!Sample_geo_accession"])
    metadata = pd.DataFrame(
        {"geo_accession": sample_fields["!Sample_geo_accession"]}
    )
    metadata["title"] = sample_fields["!Sample_title"]
    for row in characteristic_rows:
        name = row[0].split(":")[0]
        values = [
            value.split(":", 1)[-1].strip() if ":" in value else value
            for value in row
        ]
        if len(values) == sample_count:
            metadata[name] = values
    return metadata


def _expression_frame(
    expression_rows: list[list[str]], header: list[str] | None
) -> pd.DataFrame:
    expression = pd.DataFrame(expression_rows, columns=header)
    expression = expression.rename(columns={"ID_REF": "probe_id"})
    expression["probe_id"] = expression["probe_id"].str.strip('"')
    for column in expression.columns:
        if column != "probe_id":
            expression[column] = pd.to_numeric(
                expression[column], errors="coerce"
            )
    return expression


def label_samples(metadata: pd.DataFrame) -> pd.DataFrame:
    is_healthy = metadata["title"].str.startswith("healthy subject")
    is_infected = _matches_any(metadata, DISCOVERY_INFECTED_LABELS)
    is_noninfected_icu = _matches_any(metadata, DISCOVERY_NONINFECTED_LABELS)
    metadata["group"] = np.select(
        [is_infected, is_healthy, is_noninfected_icu],
        ["sepsis", "healthy", "noninfected_icu"],
        default="unlabeled",
    )
    metadata["patient_code"] = metadata["title"].apply(_patient_code)

    unlabeled_count = (metadata["group"] == "unlabeled").sum()
    labeled = metadata[metadata["group"] != "unlabeled"].copy()
    duplicate_count = labeled["patient_code"].duplicated().sum()
    logger.info(
        "n_dataset_total=%d, n_unlabeled_excluded=%d, n_labeled=%d, "
        "duplicate patient codes among labeled=%d",
        len(metadata),
        unlabeled_count,
        len(labeled),
        duplicate_count,
    )
    logger.info("%s", labeled["group"].value_counts().to_dict())
    if duplicate_count:
        raise ValueError(
            "duplicate patient codes in the labeled discovery sample"
        )
    return labeled


def _matches_any(
    metadata: pd.DataFrame, labels: dict[str, set[str]]
) -> pd.Series:
    matched = pd.Series(False, index=metadata.index)
    for column, values in labels.items():
        if column in metadata.columns:
            matched = matched | metadata[column].isin(values)
    return matched


def _patient_code(title: str) -> str:
    bracketed = re.search(r"\[(.*)\]", title)
    if not bracketed:
```

```python
        return title
    return bracketed.group(1).split("_")[-1]


def collapse_to_genes(
    probe_expression: pd.DataFrame, sample_columns: list[str]
) -> pd.DataFrame:
    samples_only = probe_expression[["probe_id", *sample_columns]]
    probe_map = pd.read_csv(INTERIM_DATA_DIR / "GPL13667_probe2gene.csv")
    mapped = samples_only.merge(probe_map, on="probe_id", how="inner")
    logger.info(
        "probes mapped to a gene symbol: %d / %d",
        len(mapped),
        len(samples_only),
    )
    mapped["_mean_expr"] = mapped[sample_columns].mean(axis=1)
    best_probes = mapped.groupby("gene_symbol")["_mean_expr"].idxmax()
    gene_expression = (
        mapped.loc[best_probes].set_index("gene_symbol")[sample_columns].T
    )
    gene_expression.index.name = "geo_accession"
    logger.info(
        "discovery gene-level matrix: %d samples x %d genes",
        gene_expression.shape[0],
        gene_expression.shape[1],
    )
    return gene_expression


if __name__ == "__main__":
    main()
```

8 src/build_validation.py

```python
from __future__ import annotations

import gzip
import logging
from pathlib import Path

import pandas as pd

from config import (
    INTERIM_DATA_DIR,
    PROCESSED_DATA_DIR,
    VALIDATION_MATRIX,
    VALIDATION_TIMEPOINT,
    configure_logging,
)

logger = logging.getLogger(__name__)


def main() -> None:
    configure_logging()
    metadata, probe_expression = read_series_matrix(VALIDATION_MATRIX)
    logger.info(
        "parsed %s: %d samples, %d probes",
        VALIDATION_MATRIX.name,
        metadata.shape[0],
        probe_expression.shape[0],
    )
    analytic = select_analytic_samples(metadata)
    analytic.to_csv(
        INTERIM_DATA_DIR / "validation_sample_metadata.csv", index=False
    )
    gene_expression = collapse_to_genes(
        probe_expression, analytic["geo_accession"].tolist()
    )
    gene_expression.to_csv(
        PROCESSED_DATA_DIR / "validation_expr_gene.csv.gz",
        compression="gzip",
    )
```

```python
    analytic.set_index("geo_accession").to_csv(
        PROCESSED_DATA_DIR / "validation_labels.csv"
    )


def read_series_matrix(
    matrix_path: Path,
) -> tuple[pd.DataFrame, pd.DataFrame]:
    sample_fields: dict[str, list[str]] = {}
    header = None
    expression_rows: list[list[str]] = []
    in_table = False
    with gzip.open(
        matrix_path, "rt", encoding="utf-8", errors="replace"
    ) as matrix:
        for raw_line in matrix:
            line = raw_line.rstrip("\n")
            if line.startswith("!series_matrix_table_begin"):
                in_table = True
                continue
            if line.startswith("!series_matrix_table_end"):
                in_table = False
                continue
            if in_table:
                if header is None:
                    header = [cell.strip('"') for cell in line.split("\t")]
                else:
                    expression_rows.append(line.split("\t"))
                continue
            if line.startswith("!Sample_"):
                sample_fields[line.split("\t")[0]] = [
                    value.strip('"') for value in line.split("\t")[1:]
                ]

    metadata = pd.DataFrame(
        {
            "geo_accession": sample_fields["!Sample_geo_accession"],
            "title": sample_fields["!Sample_title"],
            "source": sample_fields["!Sample_source_name_ch1"],
        }
    )
    expression = pd.DataFrame(expression_rows, columns=header)
    expression = expression.rename(columns={"ID_REF": "probe_id"})
    expression["probe_id"] = expression["probe_id"].str.strip('"')
    for column in expression.columns:
        if column != "probe_id":
            expression[column] = pd.to_numeric(
                expression[column], errors="coerce"
            )
    return metadata, expression


def select_analytic_samples(metadata: pd.DataFrame) -> pd.DataFrame:
    is_healthy = metadata["title"].str.startswith("Blood_HV")
    is_baseline_shock = metadata["title"].str.contains(
        r"_P\d+_" + VALIDATION_TIMEPOINT + r"$", regex=True
    )
    metadata["group"] = "excluded"
    metadata.loc[is_healthy, "group"] = "healthy"
    metadata.loc[is_baseline_shock, "group"] = "sepsis"

    analytic = metadata[metadata["group"] != "excluded"].copy()
    logger.info(
        "n_dataset_total=%d, n_excluded_followup_draws=%d, n_analytic=%d",
        len(metadata),
        len(metadata) - len(analytic),
        len(analytic),
    )
    logger.info("%s", analytic["group"].value_counts().to_dict())
    return analytic


def collapse_to_genes(
    probe_expression: pd.DataFrame, sample_columns: list[str]
) -> pd.DataFrame:
    samples_only = probe_expression[["probe_id", *sample_columns]]
```

```python
    probe_map = pd.read_csv(INTERIM_DATA_DIR / "GPL570_probe2gene.csv")
    mapped = samples_only.merge(probe_map, on="probe_id", how="inner")
    logger.info(
        "probes mapped to a gene symbol: %d / %d",
        len(mapped),
        len(samples_only),
    )
    mapped["_mean_expr"] = mapped[sample_columns].mean(axis=1)
    best_probes = mapped.groupby("gene_symbol")["_mean_expr"].idxmax()
    gene_expression = (
        mapped.loc[best_probes].set_index("gene_symbol")[sample_columns].T
    )
    gene_expression.index.name = "geo_accession"
    logger.info(
        "validation gene-level matrix: %d samples x %d genes",
        gene_expression.shape[0],
        gene_expression.shape[1],
    )
    return gene_expression


if __name__ == "__main__":
    main()
```

9 src/validation_metadata_extra.py

```python
from __future__ import annotations

import gzip
import logging
from pathlib import Path

import pandas as pd

from config import INTERIM_DATA_DIR, VALIDATION_MATRIX, configure_logging

logger = logging.getLogger(__name__)

HEADER_FIELDS = (
    "!Sample_characteristics_ch1",
    "!Sample_title",
    "!Sample_geo_accession",
)


def main() -> None:
    configure_logging()
    header_fields = read_header_fields(VALIDATION_MATRIX)
    characteristics = header_fields["!Sample_characteristics_ch1"]
    age_row = next(row for row in characteristics if row[0].startswith("age"))
    gender_row = next(
        row for row in characteristics if row[0].startswith("gender")
    )
    age_gender = pd.DataFrame(
        {
            "geo_accession": header_fields["!Sample_geo_accession"][0],
            "title": header_fields["!Sample_title"][0],
            "age": _characteristic_values(age_row),
            "gender": _characteristic_values(gender_row),
        }
    )
    age_gender.to_csv(
        INTERIM_DATA_DIR / "validation_age_gender.csv", index=False
    )
    logger.info("wrote %d rows -> validation_age_gender.csv", len(age_gender))
    logger.info("\n%s", age_gender.head())


def read_header_fields(matrix_path: Path) -> dict[str, list[list[str]]]:
    header_fields: dict[str, list[list[str]]] = {}
    with gzip.open(
        matrix_path, "rt", encoding="utf-8", errors="replace"
    ) as matrix:
```

```python
        for line in matrix:
            if line.startswith("!series_matrix_table_begin"):
                break
            if line.startswith(HEADER_FIELDS):
                values = [
                    value.strip('"') for value in line.rstrip("\n").split("\t")
                ]
                header_fields.setdefault(values[0], []).append(values[1:])
    return header_fields


def _characteristic_values(row: list[str]) -> list[str]:
    return [value.split(":", 1)[-1].strip() for value in row]


if __name__ == "__main__":
    main()
```

10 src/platform_audit.py

```python
from __future__ import annotations

from dataclasses import dataclass

import pandas as pd


@dataclass
class FeatureAudit:

    feature: str
    source_coverage: float
    target_coverage: float
    present_in_target: bool
    verdict: str
    note: str = ""


@dataclass
class AuditReport:

    features: list[FeatureAudit]
    n_source: int
    n_target: int

    def to_frame(self) -> pd.DataFrame:
        return pd.DataFrame(
            [
                {
                    "feature": f.feature,
                    "source_coverage": f.source_coverage,
                    "target_coverage": f.target_coverage,
                    "present_in_target": f.present_in_target,
                    "verdict": f.verdict,
                    "note": f.note,
                }
                for f in self.features
            ]
        )

    def summary(self) -> str:
        n = len(self.features)
        ok = sum(f.verdict == "ok" for f in self.features)
        absent = sum(f.verdict == "absent" for f in self.features)
        sparse = sum(f.verdict == "sparse" for f in self.features)
        return (
            f"{ok}/{n} features transportable; "
            f"{absent} absent, {sparse} sparse. "
            f"source n={self.n_source}, target n={self.n_target}"
        )


def audit_feature_availability(
```

```
    source: pd.DataFrame,
    target: pd.DataFrame,
    features: list[str],
    *,
    sparse_threshold: float = 0.50,
) -> AuditReport:
    audits: list[FeatureAudit] = []
    for feat in features:
        src_cov = (
            float(source[feat].notna().mean())
            if feat in source.columns
            else 0.0
        )
        if feat in target.columns:
            tgt_cov = float(target[feat].notna().mean())
            present = True
        else:
            tgt_cov, present = 0.0, False

        if not present or tgt_cov == 0.0:
            verdict, note = "absent", "not supplied by the target cohort"
        elif tgt_cov < sparse_threshold:
            verdict, note = (
                "sparse",
                "coverage below threshold; imputation will dominate",
            )
        else:
            verdict, note = "ok", ""
        audits.append(
            FeatureAudit(feat, src_cov, tgt_cov, present, verdict, note)
        )

    return AuditReport(audits, len(source), len(target))
```

11 src/transport_audit.py

```
from __future__ import annotations

import logging

import pandas as pd

from config import (
    METRICS_DIR,
    PROCESSED_DATA_DIR,
    TABLES_DIR,
    configure_logging,
)
from platform_audit import audit_feature_availability

logger = logging.getLogger(__name__)


def main() -> None:
    configure_logging()
    discovery = pd.read_csv(
        PROCESSED_DATA_DIR / "discovery_expr_gene.csv.gz", index_col=0
    )
    validation = pd.read_csv(
        PROCESSED_DATA_DIR / "validation_expr_gene.csv.gz", index_col=0
    )
    discovery_genes = list(discovery.columns)
    report = audit_feature_availability(
        discovery, validation, discovery_genes, sparse_threshold=0.50
    )
    audit = report.to_frame()
    audit.to_csv(TABLES_DIR / "transport_audit.csv", index=False)
    logger.info("%s", report.summary())

    transportable_count = (audit["verdict"] == "ok").sum()
    absent_count = (audit["verdict"] == "absent").sum()
    logger.info("transportable (verdict=ok): %d", transportable_count)
    logger.info("absent from validation platform: %d", absent_count)
```

```python

    write_transportable_genes(audit)
    summary = {
        "n_discovery_genes": len(discovery_genes),
        "n_validation_genes": validation.shape[1],
        "n_transportable": int(transportable_count),
        "n_absent_from_validation": int(absent_count),
        "pct_transportable": round(
            100 * transportable_count / len(discovery_genes), 1
        ),
    }
    pd.Series(summary).to_json(METRICS_DIR / "transport_audit_summary.json")
    logger.info("%s", summary)


def write_transportable_genes(audit: pd.DataFrame) -> None:
    transportable = sorted(
        audit.loc[audit["verdict"] == "ok", "feature"].tolist()
    )
    pd.Series(transportable, name="gene_symbol").to_csv(
        PROCESSED_DATA_DIR / "transportable_genes.csv", index=False
    )
    logger.info(
        "wrote %d transportable genes -> transportable_genes.csv",
        len(transportable),
    )


if __name__ == "__main__":
    main()
```

12 src/quantify_batch_platform.py

```python
from __future__ import annotations

import json
import logging

import matplotlib.pyplot as plt
import numpy as np
import pandas as pd
from sklearn.decomposition import PCA
from sklearn.preprocessing import StandardScaler

import formatting
import plotting
from config import (
    FIGURES_DIR,
    METRICS_DIR,
    PROCESSED_DATA_DIR,
    RANDOM_SEED,
    configure_logging,
)

logger = logging.getLogger(__name__)

PERMANOVA_PERMUTATIONS = 999
TOP_VARIANCE_GENES = 2000
PRINCIPAL_COMPONENTS = 10


GROUP_LABELS = {"noninfected_icu": "non-infected ICU"}


def main() -> None:
    configure_logging()
    components, explained, dataset, diagnosis = build_ordination()
    structure = test_structure(components, dataset, diagnosis)
    plot_ordination(components, explained, dataset, diagnosis)
    write_summary(structure, explained)


def build_ordination() -> (
```

```python
    tuple[np.ndarray, np.ndarray, np.ndarray, np.ndarray]
):
    discovery, validation, labels = _load_transportable_expression()
    top_genes = _top_variance_genes(discovery, validation)
    combined = pd.concat([discovery[top_genes], validation[top_genes]], axis=0)
    dataset = np.array(
        ["discovery"] * len(discovery) + ["validation"] * len(validation)
    )
    diagnosis = np.concatenate(
        [
            labels["discovery"].loc[discovery.index, "group"].values,
            labels["validation"].loc[validation.index, "group"].values,
        ]
    )
    standardized = StandardScaler().fit_transform(
        combined.values.astype(np.float32)
    )
    pca = PCA(n_components=PRINCIPAL_COMPONENTS, random_state=RANDOM_SEED)
    components = pca.fit_transform(standardized)
    return components, pca.explained_variance_ratio_, dataset, diagnosis


def _load_transportable_expression() -> (
    tuple[pd.DataFrame, pd.DataFrame, dict[str, pd.DataFrame]]
):
    transportable = pd.read_csv(
        PROCESSED_DATA_DIR / "transportable_genes.csv"
    )["gene_symbol"].tolist()
    columns = ["geo_accession", *transportable]
    discovery = pd.read_csv(
        PROCESSED_DATA_DIR / "discovery_expr_gene.csv.gz",
        index_col=0,
        usecols=columns,
    ).astype(np.float32)
    validation = pd.read_csv(
        PROCESSED_DATA_DIR / "validation_expr_gene.csv.gz",
        index_col=0,
        usecols=columns,
    ).astype(np.float32)
    labels = {
        "discovery": pd.read_csv(
            PROCESSED_DATA_DIR / "discovery_labels.csv", index_col=0
        ),
        "validation": pd.read_csv(
            PROCESSED_DATA_DIR / "validation_labels.csv", index_col=0
        ),
    }
    shared = [
        gene
        for gene in transportable
        if gene in discovery.columns and gene in validation.columns
    ]
    return discovery[shared], validation[shared], labels


def _top_variance_genes(
    discovery: pd.DataFrame, validation: pd.DataFrame
) -> list[str]:
    variance_rank = discovery.var().rank(
        ascending=False
    ) + validation.var().rank(ascending=False)
    return variance_rank.sort_values().index[:TOP_VARIANCE_GENES].tolist()


def test_structure(
    components: np.ndarray, dataset: np.ndarray, diagnosis: np.ndarray
) -> dict:
    is_sepsis = (diagnosis == "sepsis").astype(int)
    pooled_dataset = permanova(components, dataset)
    pooled_diagnosis = permanova(components, is_sepsis)
    logger.info(
        "(a) pooled dataset/cohort/platform: R2=%s, F=%s, p=%s",
        pooled_dataset["r2"],
        pooled_dataset["pseudo_F"],
        pooled_dataset["p_value"],
    )
```

```
    logger.info(
        "(a) pooled sepsis-vs-not: R2=%s, F=%s, p=%s",
        pooled_diagnosis["r2"],
        pooled_diagnosis["pseudo_F"],
        pooled_diagnosis["p_value"],
    )

    healthy = diagnosis == "healthy"
    healthy_dataset = permanova(components[healthy], dataset[healthy])
    logger.info(
        "(b) dataset within healthy only (n=%s): R2=%s, p=%s",
        healthy.sum(),
        healthy_dataset["r2"],
        healthy_dataset["p_value"],
    )

    in_discovery = dataset == "discovery"
    discovery_sepsis_healthy = in_discovery & np.isin(
        diagnosis, ["sepsis", "healthy"]
    )
    discovery_diagnosis = permanova(
        components[discovery_sepsis_healthy],
        is_sepsis[discovery_sepsis_healthy],
    )
    discovery_three_groups = permanova(
        components[in_discovery], diagnosis[in_discovery]
    )
    logger.info(
        "(c) diagnosis within discovery (n=%s): R2=%s, p=%s",
        discovery_sepsis_healthy.sum(),
        discovery_diagnosis["r2"],
        discovery_diagnosis["p_value"],
    )

    sepsis_or_healthy = np.isin(diagnosis, ["sepsis", "healthy"])
    two_factor = permanova_two_factor(
        components[sepsis_or_healthy],
        dataset[sepsis_or_healthy],
        is_sepsis[sepsis_or_healthy],
        "dataset",
        "diagnosis",
    )
    logger.info("two-factor sequential:\n%s", json.dumps(two_factor, indent=2))
    return {
        "pooled_dataset": pooled_dataset,
        "pooled_diagnosis": pooled_diagnosis,
        "healthy_dataset": healthy_dataset,
        "discovery_diagnosis": discovery_diagnosis,
        "discovery_three_groups": discovery_three_groups,
        "two_factor": two_factor,
    }


def permanova(
    coordinates: np.ndarray,
    groups: np.ndarray,
    permutations: int = PERMANOVA_PERMUTATIONS,
    seed: int = RANDOM_SEED,
) -> dict:
    groups = np.asarray(groups)
    sample_count = len(groups)
    group_count = len(np.unique(groups))
    total_sum_of_squares = float(
        np.sum((coordinates - coordinates.mean(axis=0)) ** 2)
    )
    between = _between_group_sum_of_squares(coordinates, groups)
    within = total_sum_of_squares - between
    observed_f = (between / (group_count - 1)) / (
        within / (sample_count - group_count)
    )
    random_generator = np.random.default_rng(seed)
    null_f = np.empty(permutations)
    for permutation in range(permutations):
        permuted_between = _between_group_sum_of_squares(
            coordinates, random_generator.permutation(groups)
        )
```

```python
        null_f[permutation] = (permuted_between / (group_count - 1)) / (
            (total_sum_of_squares - permuted_between)
            / (sample_count - group_count)
        )
    p_value = (1 + int(np.sum(null_f >= observed_f))) / (1 + permutations)
    return {
        "r2": round(between / total_sum_of_squares, 4),
        "pseudo_F": round(float(observed_f), 3),
        "p_value": round(float(p_value), 5),
        "n_perm": permutations,
        "n": int(sample_count),
        "n_groups": int(group_count),
    }


def _between_group_sum_of_squares(
    coordinates: np.ndarray, groups: np.ndarray
) -> float:
    grand_centroid = coordinates.mean(axis=0)
    sum_of_squares = 0.0
    for group in np.unique(groups):
        members = groups == group
        sum_of_squares += members.sum() * np.sum(
            (coordinates[members].mean(axis=0) - grand_centroid) ** 2
        )
    return float(sum_of_squares)


def permanova_two_factor(
    coordinates: np.ndarray,
    first_factor: np.ndarray,
    second_factor: np.ndarray,
    first_name: str,
    second_name: str,
) -> dict:
    total_sum_of_squares = float(
        np.sum((coordinates - coordinates.mean(axis=0)) ** 2)
    )
    result = {}
    for entered, remaining, entered_name, remaining_name in (
        (first_factor, second_factor, first_name, second_name),
        (second_factor, first_factor, second_name, first_name),
    ):
        entered_sum_of_squares = _between_group_sum_of_squares(
            coordinates, entered
        )
        residual = _center_within_groups(coordinates, entered)
        remaining_sum_of_squares = _between_group_sum_of_squares(
            residual, remaining
        )
        result[f"{entered_name}_then_{remaining_name}"] = {
            f"r2_{entered_name}_first": round(
                entered_sum_of_squares / total_sum_of_squares, 4
            ),
            f"r2_{remaining_name}_given_{entered_name}": round(
                remaining_sum_of_squares / total_sum_of_squares, 4
            ),
            f"{remaining_name}_given_{entered_name}_permanova": permanova(
                residual, remaining
            ),
        }
    return result


def _center_within_groups(
    coordinates: np.ndarray, groups: np.ndarray
) -> np.ndarray:
    residual = coordinates.copy()
    for group in np.unique(groups):
        members = groups == group
        residual[members] = residual[members] - residual[members].mean(axis=0)
    return residual


def plot_ordination(
    components: np.ndarray,
```

```python
    explained: np.ndarray,
    dataset: np.ndarray,
    diagnosis: np.ndarray,
) -> None:
    plotting.apply_style()
    fig, axes = plt.subplots(
        1, 2, figsize=(plotting.FULL_WIDTH_INCHES * 2, 4.2)
    )
    dataset_colors = {
        "discovery": plotting.PALETTE["blue"],
        "validation": plotting.PALETTE["orange"],
    }
    diagnosis_colors = {
        "sepsis": plotting.PALETTE["red"],
        "healthy": plotting.PALETTE["green"],
        "noninfected_icu": plotting.PALETTE["gray"],
    }
    for ax, groups, colors, letter in (
        (axes[0], dataset, dataset_colors, "A"),
        (axes[1], diagnosis, diagnosis_colors, "B"),
    ):
        _scatter_groups(ax, components, groups, colors, explained)
        plotting.label_panel(ax, letter)
    plotting.save_figure(fig, FIGURES_DIR, "fig6_pca_dataset_structure")
    plt.close(fig)


def _scatter_groups(
    ax,
    components: np.ndarray,
    groups: np.ndarray,
    colors: dict[str, str],
    explained: np.ndarray,
) -> None:
    for name, color in colors.items():
        members = groups == name
        if members.sum() == 0:
            continue
        ax.scatter(
            components[members, 0],
            components[members, 1],
            s=14,
            alpha=0.7,
            color=color,
            label=GROUP_LABELS.get(name, name),
            edgecolors="none",
        )
    pc1 = formatting.format_percent(explained[0], already_fraction=True)
    pc2 = formatting.format_percent(explained[1], already_fraction=True)
    ax.set_xlabel(f"PC1 ({pc1})")
    ax.set_ylabel(f"PC2 ({pc2})")
    ax.legend()


def write_summary(structure: dict, explained: np.ndarray) -> None:
    summary = {
        "method": (
            "PERMANOVA (Anderson 2001) on Euclidean distances among the "
            f"first {PRINCIPAL_COMPONENTS} principal components of the "
            f"{TOP_VARIANCE_GENES} highest-pooled-variance transportable "
            f"genes; {PERMANOVA_PERMUTATIONS} label permutations; one "
            "StandardScaler fitted on the combined matrix for the "
            "ordination only"
        ),
        "n_top_variance_genes": TOP_VARIANCE_GENES,
        "pc1_2_explained_variance_pct": formatting.format_percent(
            float(explained[:2].sum()), already_fraction=True
        ),
        "pooled_confounded": {
            "caveat": (
                "dataset, cohort, hospital, platform and sample composition "
                "are mutually confounded in this comparison; report as "
                "dataset/cohort/platform structure, never as a platform "
                "effect"
            ),
            "dataset": structure["pooled_dataset"],
```

```python
            "diagnosis_sepsis_vs_not": structure["pooled_diagnosis"],
        },
        "deconfounded_dataset_within_healthy": {
            "rationale": (
                "healthy donors appear in both series, so diagnosis is "
                "constant"
            ),
            **structure["healthy_dataset"],
        },
        "deconfounded_diagnosis_within_discovery": {
            "rationale": (
                "platform, cohort and hospital are constant within discovery"
            ),
            "sepsis_vs_healthy": structure["discovery_diagnosis"],
            "all_three_groups": structure["discovery_three_groups"],
        },
        "two_factor_sequential_sepsis_healthy_only": structure["two_factor"],
    }
    with open(
        METRICS_DIR / "batch_platform_effect.json", "w", encoding="utf-8"
    ) as summary_file:
        json.dump(summary, summary_file, indent=2)
    logger.info("wrote batch_platform_effect.json")


if __name__ == "__main__":
    main()
```

13 src/differential_expression.py

```python
from __future__ import annotations

import logging

import numpy as np
import pandas as pd
from scipy import stats
from statsmodels.stats.multitest import multipletests

from config import (
    METRICS_DIR,
    PROCESSED_DATA_DIR,
    TABLES_DIR,
    configure_logging,
)

logger = logging.getLogger(__name__)

FDR_THRESHOLD = 0.05
FOLD_CHANGE_THRESHOLD = 1.0


def main() -> None:
    configure_logging()
    expression, is_sepsis = load_task_data()
    results = test_genes(expression, is_sepsis)
    results.to_csv(
        TABLES_DIR / "differential_expression_discovery.csv", index=False
    )

    significant = results["p_adj"] < FDR_THRESHOLD
    significant_count = significant.sum()
    large_effect_count = (
        significant & (results["log2fc"].abs() >= FOLD_CHANGE_THRESHOLD)
    ).sum()
    logger.info("genes with FDR p_adj < 0.05: %d", significant_count)
    logger.info(
        "genes with FDR p_adj < 0.05 and |log2FC| >= 1: %d",
        large_effect_count,
    )
    logger.info("\n%s", results.head(15).to_string(index=False))

    summary = {
```

```python
        "n_candidate_genes_tested": int(expression.shape[1]),
        "n_sig_fdr05": int(significant_count),
        "n_sig_fdr05_fc1": int(large_effect_count),
        "n_sepsis": int(is_sepsis.sum()),
        "n_healthy": int((1 - is_sepsis).sum()),
    }
    pd.Series(summary).to_json(METRICS_DIR / "de_summary.json")


def load_task_data() -> tuple[pd.DataFrame, np.ndarray]:
    transportable = pd.read_csv(
        PROCESSED_DATA_DIR / "transportable_genes.csv"
    )["gene_symbol"].tolist()
    expression = pd.read_csv(
        PROCESSED_DATA_DIR / "discovery_expr_gene.csv.gz",
        index_col=0,
        usecols=["geo_accession", *transportable],
    ).astype(np.float32)
    labels = pd.read_csv(
        PROCESSED_DATA_DIR / "discovery_labels.csv", index_col=0
    )

    task = labels[labels["group"].isin(["sepsis", "healthy"])]
    expression = expression.loc[task.index]
    is_sepsis = (task["group"] == "sepsis").astype(int).to_numpy()
    logger.info(
        "DE cohort: n=%d (%d sepsis, %d healthy), %d candidate genes",
        len(task),
        int(is_sepsis.sum()),
        int((1 - is_sepsis).sum()),
        expression.shape[1],
    )
    return expression, is_sepsis


def test_genes(
    expression: pd.DataFrame, is_sepsis: np.ndarray
) -> pd.DataFrame:
    sepsis = expression.values[is_sepsis == 1]
    healthy = expression.values[is_sepsis == 0]
    t_statistic, raw_p_value = stats.ttest_ind(
        sepsis, healthy, axis=0, equal_var=False
    )
    adjusted_p_value = multipletests(raw_p_value, method="fdr_bh")[1]
    return pd.DataFrame(
        {
            "gene_symbol": expression.columns,
            "log2fc": sepsis.mean(axis=0) - healthy.mean(axis=0),
            "t_stat": t_statistic,
            "p_raw": raw_p_value,
            "p_adj": adjusted_p_value,
            "mean_sepsis": sepsis.mean(axis=0),
            "mean_healthy": healthy.mean(axis=0),
        }
    ).sort_values("p_adj")


if __name__ == "__main__":
    main()
```

14 src/nested_cv_signature.py

```python
from __future__ import annotations

import itertools
import json
import logging
from collections import Counter

import numpy as np
import pandas as pd
from scipy import stats
from sklearn.linear_model import LogisticRegression
```

```python
from sklearn.metrics import roc_auc_score
from sklearn.model_selection import StratifiedKFold
from sklearn.preprocessing import StandardScaler

from config import (
    INNER_FOLDS,
    METRICS_DIR,
    OUTER_FOLDS,
    PROCESSED_DATA_DIR,
    RANDOM_SEED,
    TABLES_DIR,
    configure_logging,
)

logger = logging.getLogger(__name__)

CANDIDATE_POOL_SIZE = 300
REGULARIZATION_GRID = [0.003, 0.01, 0.03, 0.1, 0.3, 1.0]
OUT_OF_FOLD_BOOTSTRAP_REPLICATES = 1000

CONFIDENCE_INTERVAL_NOTE = (
    "TWO separate cautions apply to this interval. (1) It is narrow because it "
    "abuts the AUROC ceiling of 1.0, not because the estimate is precise: "
    "sepsis vs. healthy is a near-completely-separated task on whole-blood "
    "transcriptome, so almost any resample reproduces near-total separation. "
    "(2) It resamples a FROZEN vector of out-of-fold predictions, so it carries "
    "no uncertainty from feature selection or hyperparameter tuning -- the "
    "very steps this paper is about. The interval that repeats the whole "
    "pipeline inside the resampling is in pipeline_bootstrap_summary.json and "
    "is the one the manuscript reports. For whether the performance is "
    "distinguishable from chance, see label_permutation_summary.json; for "
    "whether the selected genes beat an arbitrary panel, see "
    "random_panel_benchmark_summary.json."
)


def main() -> None:
    configure_logging()
    features, labels, gene_names = load_discovery_task()
    logger.info(
        "nested CV cohort: n=%d (%d sepsis, %d healthy), %d candidate genes",
        len(labels),
        labels.sum(),
        (1 - labels).sum(),
        features.shape[1],
    )

    cross_validation = run_nested_cross_validation(
        features, labels, gene_names
    )
    fold_records = cross_validation["fold_records"]
    pd.DataFrame(fold_records).to_csv(
        TABLES_DIR / "nested_cv_outer_folds.csv", index=False
    )

    out_of_fold = cross_validation["out_of_fold_probabilities"]
    pooled_auroc = roc_auc_score(labels, out_of_fold)
    logger.info(
        "pooled out-of-fold AUROC (discovery, nested CV): %.4f", pooled_auroc
    )
    interval_lower, interval_upper, replicate_count = bootstrap_pooled_auroc(
        labels, out_of_fold
    )
    logger.info(
        "bootstrap 95%% CI: %.4f-%.4f (n_boot=%d)",
        interval_lower,
        interval_upper,
        replicate_count,
    )

    modal_regularization = Counter(
        cross_validation["chosen_regularization"]
    ).most_common(1)[0][0]
    locked_model, signature = fit_locked_model(
        features, labels, gene_names, modal_regularization
    )
```

```python
    signature.to_csv(TABLES_DIR / "locked_signature.csv", index=False)
    logger.info(
        "locked signature: %d genes (modal outer C=%s)",
        len(signature),
        modal_regularization,
    )
    logger.info("%s", signature.to_string(index=False))
    with open(
        PROCESSED_DATA_DIR / "locked_model.json", "w", encoding="utf-8"
    ) as model_file:
        json.dump(locked_model, model_file)

    summary = {
        "n_discovery": len(labels),
        "n_sepsis": int(labels.sum()),
        "n_healthy": int((1 - labels).sum()),
        "candidate_pool_size": CANDIDATE_POOL_SIZE,
        "outer_fold_aurocs": [
            round(record["auroc"], 4) for record in fold_records
        ],
        "pooled_oof_auroc": round(float(pooled_auroc), 4),
        "pooled_oof_auroc_ci_lo": round(float(interval_lower), 4),
        "pooled_oof_auroc_ci_hi": round(float(interval_upper), 4),
        "pooled_oof_auroc_ci_note": CONFIDENCE_INTERVAL_NOTE,
        "modal_C": modal_regularization,
        "n_signature_genes": len(signature),
        "mean_fold_pool_jaccard": round(
            float(
                mean_pairwise_jaccard(cross_validation["fold_candidate_sets"])
            ),
            3,
        ),
    }
    with open(
        METRICS_DIR / "discovery_nested_cv_summary.json", "w", encoding="utf-8"
    ) as summary_file:
        json.dump(summary, summary_file, indent=2)
    logger.info("%s", summary)


def load_discovery_task() -> tuple[np.ndarray, np.ndarray, np.ndarray]:
    transportable = pd.read_csv(
        PROCESSED_DATA_DIR / "transportable_genes.csv"
    )["gene_symbol"].tolist()
    expression = pd.read_csv(
        PROCESSED_DATA_DIR / "discovery_expr_gene.csv.gz",
        index_col=0,
        usecols=["geo_accession"] + transportable,
    ).astype(np.float32)
    groups = pd.read_csv(
        PROCESSED_DATA_DIR / "discovery_labels.csv", index_col=0
    )
    task = groups[groups["group"].isin(["sepsis", "healthy"])]
    expression = expression.loc[task.index]
    labels = (task["group"] == "sepsis").astype(int).to_numpy()
    return expression.to_numpy(), labels, np.array(expression.columns)


def run_nested_cross_validation(
    features: np.ndarray, labels: np.ndarray, gene_names: np.ndarray
) -> dict:
    outer = StratifiedKFold(
        n_splits=OUTER_FOLDS, shuffle=True, random_state=RANDOM_SEED
    )
    out_of_fold = np.full(len(labels), np.nan)
    fold_records = []
    chosen_regularization = []
    fold_candidate_sets = []

    for fold, (train, test) in enumerate(outer.split(features, labels)):
        best_regularization = select_regularization(
            features[train], labels[train], seed=RANDOM_SEED + fold
        )
        chosen_regularization.append(best_regularization)
        candidates, candidate_genes = top_t_genes(
            features[train], labels[train], gene_names, CANDIDATE_POOL_SIZE
```

```python
        )
        fold_candidate_sets.append(set(candidate_genes.tolist()))

        scaler = StandardScaler().fit(features[train][:, candidates])
        train_scaled = scaler.transform(features[train][:, candidates])
        test_scaled = scaler.transform(features[test][:, candidates])
        model = fit_l1_model(
            train_scaled,
            labels[train],
            best_regularization,
            RANDOM_SEED + fold,
        )
        test_probabilities = model.predict_proba(test_scaled)[:, 1]
        out_of_fold[test] = test_probabilities
        auroc = roc_auc_score(labels[test], test_probabilities)
        nonzero_genes = int(np.sum(model.coef_ != 0))
        fold_records.append(
            {
                "fold": fold,
                "n_train": len(train),
                "n_test": len(test),
                "best_C": best_regularization,
                "auroc": auroc,
                "n_nonzero_genes": nonzero_genes,
            }
        )
        logger.info(
            "outer fold %d: C=%s, AUROC=%.4f, n_nonzero=%d",
            fold,
            best_regularization,
            auroc,
            nonzero_genes,
        )

    return {
        "out_of_fold_probabilities": out_of_fold,
        "fold_records": fold_records,
        "chosen_regularization": chosen_regularization,
        "fold_candidate_sets": fold_candidate_sets,
    }


def select_regularization(
    features: np.ndarray, labels: np.ndarray, seed: int
) -> float:
    inner = StratifiedKFold(
        n_splits=INNER_FOLDS, shuffle=True, random_state=seed
    )
    mean_auroc = {}
    for regularization in REGULARIZATION_GRID:
        fold_aurocs = []
        for train, test in inner.split(features, labels):
            candidates, _ = top_t_genes(
                features[train],
                labels[train],
                np.arange(features.shape[1]),
                CANDIDATE_POOL_SIZE,
            )
            scaler = StandardScaler().fit(features[train][:, candidates])
            train_scaled = scaler.transform(features[train][:, candidates])
            test_scaled = scaler.transform(features[test][:, candidates])
            model = fit_l1_model(
                train_scaled, labels[train], regularization, seed
            )
            probabilities = model.predict_proba(test_scaled)[:, 1]
            fold_aurocs.append(roc_auc_score(labels[test], probabilities))
        mean_auroc[regularization] = float(np.mean(fold_aurocs))
    return max(mean_auroc, key=mean_auroc.__getitem__)


def top_t_genes(
    features: np.ndarray,
    labels: np.ndarray,
    gene_names: np.ndarray,
    pool_size: int,
) -> tuple[np.ndarray, np.ndarray]:
```

```python
    t_statistics, _ = stats.ttest_ind(
        features[labels == 1], features[labels == 0], axis=0, equal_var=False
    )
    order = np.argsort(-np.abs(t_statistics))[:pool_size]
    return order, np.asarray(gene_names)[order]


def fit_l1_model(
    features: np.ndarray,
    labels: np.ndarray,
    regularization: float,
    seed: int = RANDOM_SEED,
) -> LogisticRegression:
    return LogisticRegression(
        penalty="l1",
        solver="liblinear",
        C=regularization,
        max_iter=2000,
        random_state=seed,
    ).fit(features, labels)


def bootstrap_pooled_auroc(
    labels: np.ndarray, probabilities: np.ndarray
) -> tuple[float, float, int]:
    random_generator = np.random.default_rng(RANDOM_SEED)
    sample_count = len(labels)
    resampled_aurocs = []
    for _ in range(OUT_OF_FOLD_BOOTSTRAP_REPLICATES):
        rows = random_generator.integers(0, sample_count, sample_count)
        if len(np.unique(labels[rows])) < 2:
            continue
        resampled_aurocs.append(
            roc_auc_score(labels[rows], probabilities[rows])
        )
    lower, upper = np.percentile(resampled_aurocs, [2.5, 97.5])
    return lower, upper, len(resampled_aurocs)


def fit_locked_model(
    features: np.ndarray,
    labels: np.ndarray,
    gene_names: np.ndarray,
    regularization: float,
) -> tuple[dict, pd.DataFrame]:
    candidates, candidate_genes = top_t_genes(
        features, labels, gene_names, CANDIDATE_POOL_SIZE
    )
    scaler = StandardScaler().fit(features[:, candidates])
    scaled = scaler.transform(features[:, candidates])
    model = fit_l1_model(scaled, labels, regularization, RANDOM_SEED)
    coefficients = model.coef_.ravel()
    nonzero = coefficients != 0
    signature_genes = candidate_genes[nonzero]
    signature = pd.DataFrame(
        {"gene_symbol": signature_genes, "coef": coefficients[nonzero]}
    ).sort_values("coef", key=np.abs, ascending=False)
    locked_model = {
        "gene_names_order": gene_names.tolist(),
        "scaler_mean": scaler.mean_.tolist(),
        "scaler_scale": scaler.scale_.tolist(),
        "candidate_idx_final": candidates.tolist(),
        "modal_C": regularization,
        "model_coef": coefficients.tolist(),
        "model_intercept": float(model.intercept_[0]),
        "signature_genes": signature_genes.tolist(),
    }
    return locked_model, signature


def mean_pairwise_jaccard(gene_sets: list[set]) -> float:
    overlaps = [
        len(first & second) / len(first | second)
        for first, second in itertools.combinations(gene_sets, 2)
    ]
    return float(np.mean(overlaps))
```

```python


if __name__ == "__main__":
    main()
```

15 src/specificity_check.py

```python
from __future__ import annotations

import json
import logging

import numpy as np
import pandas as pd
from sklearn.metrics import roc_auc_score

from config import METRICS_DIR, PROCESSED_DATA_DIR, configure_logging
from scoring import load_locked_model, predict_probability

logger = logging.getLogger(__name__)

BOOTSTRAP_SEED = 42
BOOTSTRAP_REPLICATES = 1000

NOTE = (
    "locked sepsis-vs-healthy model scored, not refit, against a harder "
    "non-infected-critically-ill negative class; addresses whether the "
    "signature tracks infection specifically or generic illness severity."
)


def main() -> None:
    configure_logging()
    locked_model = load_locked_model()
    features, labels = load_specificity_cohort(
        locked_model["gene_names_order"]
    )
    logger.info(
        "specificity cohort: n=%d (%d sepsis, %d non-infected ICU)",
        len(labels),
        labels.sum(),
        (1 - labels).sum(),
    )

    probabilities = predict_probability(features, locked_model)
    auroc = roc_auc_score(labels, probabilities)
    lower, upper = bootstrap_interval(labels, probabilities)
    logger.info(
        "AUROC sepsis vs non-infected ICU (locked model, no refit): "
        "%.4f (95%% CI %.4f-%.4f)",
        auroc,
        lower,
        upper,
    )

    summary = {
        "n_sepsis": int(labels.sum()),
        "n_noninfected_icu": int((1 - labels).sum()),
        "auroc": round(float(auroc), 4),
        "auroc_ci_lo": round(float(lower), 4),
        "auroc_ci_hi": round(float(upper), 4),
        "note": NOTE,
    }
    with open(
        METRICS_DIR / "specificity_check_summary.json", "w", encoding="utf-8"
    ) as summary_file:
        json.dump(summary, summary_file, indent=2)
    logger.info("%s", summary)


def load_specificity_cohort(
    gene_order: list[str],
) -> tuple[pd.DataFrame, np.ndarray]:
```

```python
    expression = pd.read_csv(
        PROCESSED_DATA_DIR / "discovery_expr_gene.csv.gz",
        index_col=0,
        usecols=["geo_accession"] + gene_order,
    ).astype(np.float32)
    groups = pd.read_csv(
        PROCESSED_DATA_DIR / "discovery_labels.csv", index_col=0
    )
    cohort = groups[groups["group"].isin(["sepsis", "noninfected_icu"])]
    labels = (cohort["group"] == "sepsis").astype(int).to_numpy()
    return expression.loc[cohort.index], labels


def bootstrap_interval(
    labels: np.ndarray, probabilities: np.ndarray
) -> tuple[float, float]:
    random_generator = np.random.default_rng(BOOTSTRAP_SEED)
    sample_count = len(labels)
    resampled_aurocs = []
    for _ in range(BOOTSTRAP_REPLICATES):
        rows = random_generator.integers(0, sample_count, sample_count)
        if len(np.unique(labels[rows])) < 2:
            continue
        resampled_aurocs.append(
            roc_auc_score(labels[rows], probabilities[rows])
        )
    lower, upper = np.percentile(resampled_aurocs, [2.5, 97.5])
    return lower, upper


if __name__ == "__main__":
    main()
```

16 src/specificity_crossfit.py

```python
from __future__ import annotations

import json
import logging
import time

import numpy as np
import pandas as pd
from scipy import stats
from sklearn.linear_model import LogisticRegression
from sklearn.metrics import roc_auc_score
from sklearn.model_selection import StratifiedKFold
from sklearn.preprocessing import StandardScaler

from auroc_inference import auroc_report
from config import (
    INNER_FOLDS,
    METRICS_DIR,
    OUTER_FOLDS,
    PROCESSED_DATA_DIR,
    RANDOM_SEED,
    TABLES_DIR,
    configure_logging,
)
from scoring import load_locked_model, predict_probability

logger = logging.getLogger(__name__)

CANDIDATE_POOL_SIZE = 300
REGULARIZATION_GRID = [0.003, 0.01, 0.03, 0.1, 0.3, 1.0]

ESTIMAND = (
    "AUROC of the study's selection-and-fit pipeline discriminating "
    "sepsis from non-infected ICU controls, with no sample "
    "contributing to the model that scores it"
)
POOLING_NOTE = (
    "The raw-pooled value is lower than the fold mean because each fold "
```

```
    "model is calibrated on its own training partition, so pooling raw "
    "probabilities lets between-fold offsets degrade the global ranking. "
    "Converting each fold's scores to within-fold percentile ranks before "
    "pooling removes those offsets. All three numbers are reported; the fold "
    "mean is the primary estimate of the procedure's discrimination."
)
LOCKED_MODEL_NOTE = (
    "The locked-model value is retained only to quantify the optimism of "
    "the partly in-sample estimate. The cross-fitted value is the one the "
    "manuscript reports."
)


def main() -> None:
    configure_logging()
    start_time = time.time()
    locked_model = load_locked_model()
    expression, groups = load_discovery(locked_model["gene_names_order"])

    sepsis_ids = groups.index[groups["group"] == "sepsis"]
    healthy_ids = groups.index[groups["group"] == "healthy"]
    control_ids = groups.index[groups["group"] == "noninfected_icu"]
    logger.info(
        "sepsis %d, healthy %d, non-infected ICU %d",
        len(sepsis_ids),
        len(healthy_ids),
        len(control_ids),
    )

    specificity_ids = list(sepsis_ids) + list(control_ids)
    specificity_labels = np.array(
        [1] * len(sepsis_ids) + [0] * len(control_ids)
    )
    locked_probabilities = predict_probability(
        expression.loc[specificity_ids], locked_model
    )
    locked_report = auroc_report(specificity_labels, locked_probabilities)
    logger.info(
        "locked model, all 242 sepsis in-sample: AUROC %.4f (%s)",
        locked_report["auroc"],
        locked_report["reported_ci_method"],
    )

    cross_fit = cross_fit_predictions(
        expression, sepsis_ids, healthy_ids, control_ids, specificity_ids
    )
    folds = pd.DataFrame(cross_fit["fold_rows"])
    folds.to_csv(TABLES_DIR / "specificity_crossfit_folds.csv", index=False)

    fold_aurocs = folds["auroc"].values
    rank_scores = within_fold_ranks(
        cross_fit["probabilities"], cross_fit["fold_of_sample"]
    )
    raw_report = auroc_report(specificity_labels, cross_fit["probabilities"])
    rank_report = auroc_report(specificity_labels, rank_scores)
    fold_mean, fold_interval = fold_mean_interval(fold_aurocs)
    log_estimates(
        fold_mean, fold_aurocs, fold_interval, raw_report, rank_report
    )

    pd.DataFrame(
        {
            "geo_accession": specificity_ids,
            "y_true": specificity_labels,
            "fold": cross_fit["fold_of_sample"],
            "p_crossfit": cross_fit["probabilities"],
            "p_crossfit_within_fold_rank": rank_scores,
            "p_locked_in_sample": locked_probabilities,
        }
    ).to_csv(TABLES_DIR / "specificity_crossfit_predictions.csv", index=False)

    summary = {
        "estimand": ESTIMAND,
        "n_sepsis": len(sepsis_ids),
        "n_noninfected_icu": len(control_ids),
        "n_healthy_always_in_training": len(healthy_ids),
```

```python
        "n_folds": OUTER_FOLDS,
        "primary_estimate": "crossfit_fold_auroc_mean",
        "crossfit_fold_auroc_mean": round(fold_mean, 4),
        "crossfit_fold_auroc_ci_lo": round(fold_interval[0], 4),
        "crossfit_fold_auroc_ci_hi": round(fold_interval[1], 4),
        "crossfit_fold_auroc_ci_method": "t interval across 5 fold estimates",
        "crossfit_fold_aurocs": [float(auroc) for auroc in fold_aurocs],
        "crossfit_fold_auroc_min": round(float(fold_aurocs.min()), 4),
        "crossfit_fold_auroc_max": round(float(fold_aurocs.max()), 4),
        "crossfit_pooled_rank_auroc": round(rank_report["auroc"], 4),
        "crossfit_pooled_rank_ci_lo": round(rank_report["reported_ci_lo"], 4),
        "crossfit_pooled_rank_ci_hi": round(rank_report["reported_ci_hi"], 4),
        "crossfit_pooled_rank_ci_method": rank_report["reported_ci_method"],
        "crossfit_pooled_raw_auroc": round(raw_report["auroc"], 4),
        "crossfit_pooled_raw_ci_lo": round(raw_report["reported_ci_lo"], 4),
        "crossfit_pooled_raw_ci_hi": round(raw_report["reported_ci_hi"], 4),
        "pooling_note": POOLING_NOTE,
        "locked_in_sample_auroc": round(locked_report["auroc"], 4),
        "locked_in_sample_ci_method": locked_report["reported_ci_method"],
        "locked_in_sample_ci_lo": _rounded(locked_report["reported_ci_lo"]),
        "locked_in_sample_ci_hi": _rounded(locked_report["reported_ci_hi"]),
        "optimism_vs_fold_mean": round(locked_report["auroc"] - fold_mean, 4),
        "note": LOCKED_MODEL_NOTE,
        "runtime_s": round(time.time() - start_time, 1),
    }
    with open(
        METRICS_DIR / "specificity_crossfit_summary.json",
        "w",
        encoding="utf-8",
    ) as summary_file:
        json.dump(summary, summary_file, indent=2)
    logger.info("%s", json.dumps(summary, indent=2))


def load_discovery(gene_order: list[str]) -> tuple[pd.DataFrame, pd.DataFrame]:
    expression = pd.read_csv(
        PROCESSED_DATA_DIR / "discovery_expr_gene.csv.gz",
        index_col=0,
        usecols=["geo_accession"] + gene_order,
    ).astype(np.float32)
    groups = pd.read_csv(
        PROCESSED_DATA_DIR / "discovery_labels.csv", index_col=0
    )
    return expression[gene_order], groups


def cross_fit_predictions(
    expression: pd.DataFrame,
    sepsis_ids: pd.Index,
    healthy_ids: pd.Index,
    control_ids: pd.Index,
    specificity_ids: list[str],
) -> dict:
    splitter = StratifiedKFold(
        n_splits=OUTER_FOLDS, shuffle=True, random_state=RANDOM_SEED
    )
    case_folds = _fold_assignment(splitter, len(sepsis_ids))
    control_folds = _fold_assignment(splitter, len(control_ids))
    position = {sample: row for row, sample in enumerate(specificity_ids)}

    probabilities = np.full(len(specificity_ids), np.nan)
    fold_of_sample = np.full(len(specificity_ids), -1)
    fold_rows = []
    healthy_features = expression.loc[healthy_ids].values

    for fold in range(OUTER_FOLDS):
        train_cases = sepsis_ids[case_folds != fold]
        test_cases = sepsis_ids[case_folds == fold]
        test_controls = control_ids[control_folds == fold]

        train_features = np.vstack(
            [expression.loc[train_cases].values, healthy_features]
        )
        train_labels = np.r_[
            np.ones(len(train_cases), dtype=int),
            np.zeros(len(healthy_ids), dtype=int),
```

```
        ]
        best_regularization = select_regularization(
            train_features, train_labels, seed=RANDOM_SEED + fold
        )
        candidates = top_t_indices(
            train_features, train_labels, CANDIDATE_POOL_SIZE
        )
        scaler = StandardScaler().fit(train_features[:, candidates])
        model = fit_l1_model(
            scaler.transform(train_features[:, candidates]),
            train_labels,
            best_regularization,
            RANDOM_SEED + fold,
        )

        test_features = np.vstack(
            [
                expression.loc[test_cases].values,
                expression.loc[test_controls].values,
            ]
        )
        test_labels = np.r_[
            np.ones(len(test_cases), dtype=int),
            np.zeros(len(test_controls), dtype=int),
        ]
        test_probabilities = model.predict_proba(
            scaler.transform(test_features[:, candidates])
        )[:, 1]

        rows = [position[sample] for sample in test_cases] + [
            position[sample] for sample in test_controls
        ]
        probabilities[rows] = test_probabilities
        fold_of_sample[rows] = fold

        fold_auroc = roc_auc_score(test_labels, test_probabilities)
        fold_rows.append(
            {
                "fold": fold,
                "n_train_sepsis": len(train_cases),
                "n_train_healthy": len(healthy_ids),
                "n_test_sepsis": len(test_cases),
                "n_test_noninfected_icu": len(test_controls),
                "best_C": best_regularization,
                "n_nonzero_genes": int(np.sum(model.coef_ != 0)),
                "auroc": round(float(fold_auroc), 4),
            }
        )
        logger.info(
            "  fold %d: C=%s, test %d sepsis vs %d non-infected ICU, "
            "AUROC=%.4f",
            fold,
            best_regularization,
            len(test_cases),
            len(test_controls),
            fold_auroc,
        )

    if np.isnan(probabilities).any():
        raise ValueError("every specificity sample must be scored once")
    return {
        "probabilities": probabilities,
        "fold_of_sample": fold_of_sample,
        "fold_rows": fold_rows,
    }


def _fold_assignment(
    splitter: StratifiedKFold, sample_count: int
) -> np.ndarray:
    assignment = np.empty(sample_count, dtype=int)
    placeholder = np.zeros(sample_count)
    for fold, (_, test) in enumerate(splitter.split(placeholder, placeholder)):
        assignment[test] = fold
    return assignment
```

```python

def select_regularization(
    features: np.ndarray, labels: np.ndarray, seed: int
) -> float:
    inner = StratifiedKFold(
        n_splits=INNER_FOLDS, shuffle=True, random_state=seed
    )
    mean_auroc = {}
    for regularization in REGULARIZATION_GRID:
        fold_aurocs = []
        for train, test in inner.split(features, labels):
            candidates = top_t_indices(
                features[train], labels[train], CANDIDATE_POOL_SIZE
            )
            scaler = StandardScaler().fit(features[train][:, candidates])
            model = fit_l1_model(
                scaler.transform(features[train][:, candidates]),
                labels[train],
                regularization,
                seed,
            )
            probabilities = model.predict_proba(
                scaler.transform(features[test][:, candidates])
            )[:, 1]
            fold_aurocs.append(roc_auc_score(labels[test], probabilities))
        mean_auroc[regularization] = float(np.mean(fold_aurocs))
    return max(mean_auroc, key=mean_auroc.__getitem__)


def top_t_indices(
    features: np.ndarray, labels: np.ndarray, pool_size: int
) -> np.ndarray:
    t_statistics, _ = stats.ttest_ind(
        features[labels == 1], features[labels == 0], axis=0, equal_var=False
    )
    return np.argsort(-np.abs(np.nan_to_num(t_statistics, nan=0.0)))[
        :pool_size
    ]


def fit_l1_model(
    features: np.ndarray,
    labels: np.ndarray,
    regularization: float,
    seed: int,
) -> LogisticRegression:
    return LogisticRegression(
        penalty="l1",
        solver="liblinear",
        C=regularization,
        max_iter=2000,
        random_state=seed,
    ).fit(features, labels)


def within_fold_ranks(
    probabilities: np.ndarray, fold_of_sample: np.ndarray
) -> np.ndarray:
    ranks = np.empty_like(probabilities)
    for fold in range(OUTER_FOLDS):
        in_fold = fold_of_sample == fold
        ranks[in_fold] = stats.rankdata(probabilities[in_fold]) / (
            in_fold.sum() + 1
        )
    return ranks


def fold_mean_interval(
    fold_aurocs: np.ndarray,
) -> tuple[float, tuple[float, float]]:
    fold_mean = float(fold_aurocs.mean())
    standard_error = float(fold_aurocs.std(ddof=1) / np.sqrt(len(fold_aurocs)))
    t_critical = float(stats.t.ppf(0.975, len(fold_aurocs) - 1))
    return fold_mean, (
        fold_mean - t_critical * standard_error,
        fold_mean + t_critical * standard_error,
```

```python
    )


def log_estimates(
    fold_mean: float,
    fold_aurocs: np.ndarray,
    fold_interval: tuple[float, float],
    raw_report: dict,
    rank_report: dict,
) -> None:
    logger.info(
        "cross-fitted fold-mean AUROC: %.4f (per-fold %.3f-%.3f, "
        "t-based 95%% CI %.3f-%.3f)",
        fold_mean,
        fold_aurocs.min(),
        fold_aurocs.max(),
        fold_interval[0],
        fold_interval[1],
    )
    for name, report in (
        ("raw scores", raw_report),
        ("within-fold ranks", rank_report),
    ):
        logger.info(
            "cross-fitted pooled, %s: %.4f (%.3f-%.3f)",
            name,
            report["auroc"],
            report["reported_ci_lo"],
            report["reported_ci_hi"],
        )


def _rounded(value: float | None) -> float | None:
    return None if value is None else round(value, 4)


if __name__ == "__main__":
    main()
```

17 src/batch_source_structure.py

```python
from __future__ import annotations

import gzip
import logging

import numpy as np
import pandas as pd
from scipy import stats
from sklearn.linear_model import LogisticRegression
from sklearn.metrics import roc_auc_score
from sklearn.model_selection import StratifiedKFold, cross_val_predict
from sklearn.pipeline import make_pipeline
from sklearn.preprocessing import StandardScaler

from config import (
    DISCOVERY_MATRIX,
    PROCESSED_DATA_DIR,
    RANDOM_SEED,
    TABLES_DIR,
    configure_logging,
)
from scoring import load_locked_model, predict_probability

logger = logging.getLogger(__name__)

HIGH_VARIANCE_GENE_COUNT = 2000
MINIMUM_SAMPLES_PER_SCAN_DATE = 10
PNEUMONIA_LABELS = {"cap", "hap", "no-cap"}
ABDOMINAL_LABELS = {"abdo_s", "ctrl_GI"}
GENE_SET_NAMES = ("2000 highest-variance genes", "10 signature genes")


```

```python
def main() -> None:
    configure_logging()
    groups = load_groups()
    expression = pd.read_csv(
        PROCESSED_DATA_DIR / "discovery_expr_gene.csv.gz", index_col=0
    ).astype(np.float32)
    expression = expression.loc[groups.index]
    locked_model = load_locked_model()
    high_variance_genes = (
        expression.var()
        .sort_values(ascending=False)
        .index[:HIGH_VARIANCE_GENE_COUNT]
    )
    gene_sets = dict(
        zip(
            GENE_SET_NAMES,
            (high_variance_genes, list(locked_model["signature_genes"])),
            strict=True,
        )
    )

    inventory = sample_inventory(set(groups.index))
    inventory.to_csv(
        TABLES_DIR / "discovery_sample_inventory.csv", index=False
    )
    logger.info("%s", inventory.to_string())

    probabilities = pd.Series(
        predict_probability(expression, locked_model), index=expression.index
    )
    rows = scan_batch_rows(groups, expression, gene_sets, probabilities)
    rows += substudy_rows(groups, expression, gene_sets)
    rows += cross_fitted_substudy_rows(groups)

    checks = pd.DataFrame(rows).astype(
        {"n_cases": "Int64", "n_controls": "Int64"}
    )
    checks.to_csv(TABLES_DIR / "batch_source_structure.csv", index=False)
    logger.info("%s", checks.to_string())


def load_groups() -> pd.DataFrame:
    groups = pd.read_csv(
        PROCESSED_DATA_DIR / "discovery_labels.csv", index_col=0
    )
    groups["scan_date"] = groups["title"].str.extract(r"\[(\d\d_\d\d_\d{4})_")[
        0
    ]
    if groups["scan_date"].isna().any():
        raise ValueError("every GSE65682 title should carry a scan date")
    groups["substudy"] = np.select(
        [
            groups["pneumonia diagnoses"].isin(PNEUMONIA_LABELS),
            groups["abdominal_sepsis_and_controls"].isin(ABDOMINAL_LABELS),
        ],
        ["pneumonia", "abdominal"],
        default="healthy_volunteers",
    )
    return groups


def sample_inventory(labeled_ids: set[str]) -> pd.DataFrame:
    metadata = _read_series_metadata()
    metadata["code"] = metadata["title"].str.extract(r"\[(.*)\]")[0]
    labeled = metadata["geo_accession"].isin(labeled_ids)
    excluded = metadata[~labeled]
    endotype = excluded["endotype_cohort"].isin(["discovery", "validation"])
    infection_event = excluded["icu_acquired_infection_paired"].eq(
        "Event_ICUAI"
    )
    rows = [
        ("GSM samples in the series", len(metadata)),
        (
            "distinct sample-title codes in the series",
            metadata["code"].nunique(),
        ),
```

```python
        ("analyzed (labeled) samples", int(labeled.sum())),
        ("excluded samples", len(excluded)),
        (
            "excluded: sepsis patients in the MARS endotype cohorts",
            int(endotype.sum()),
        ),
        (
            "excluded: draws taken at an ICU-acquired infection event",
            int((infection_event & ~endotype).sum()),
        ),
        (
            "excluded: no diagnostic or cohort label",
            int((~endotype & ~infection_event).sum()),
        ),
    ]
    return pd.DataFrame(rows, columns=["item", "n"])


def _read_series_metadata() -> pd.DataFrame:
    characteristics: list[list[str]] = []
    accessions: list[str] = []
    titles: list[str] = []
    with gzip.open(
        DISCOVERY_MATRIX, "rt", encoding="utf-8", errors="replace"
    ) as matrix_file:
        for line in matrix_file:
            if line.startswith("!series_matrix_table_begin"):
                break
            fields = [
                field.strip('"') for field in line.rstrip("\n").split("\t")
            ]
            if fields[0] == "!Sample_geo_accession":
                accessions = fields[1:]
            elif fields[0] == "!Sample_title":
                titles = fields[1:]
            elif fields[0] == "!Sample_characteristics_ch1":
                characteristics.append(fields[1:])
    metadata = pd.DataFrame({"geo_accession": accessions, "title": titles})
    for characteristic_row in characteristics:
        for sample, entry in enumerate(characteristic_row):
            if ":" in entry:
                key, value = entry.split(":", 1)
                metadata.loc[sample, key.strip()] = value.strip()
    return metadata


def scan_batch_rows(
    groups: pd.DataFrame,
    expression: pd.DataFrame,
    gene_sets: dict,
    probabilities: pd.Series,
) -> list[dict]:
    rows = []
    date_by_group = pd.crosstab(groups["scan_date"], groups["group"])
    date_by_group.to_csv(TABLES_DIR / "scan_date_by_group.csv")
    healthy_dates = date_by_group.index[date_by_group["healthy"] > 0]
    sepsis_healthy = groups[groups["group"].isin(["sepsis", "healthy"])]
    chi_square, p_value = _chi_square(sepsis_healthy)
    rows.append(
        _row(
            "scan date vs group, sepsis/healthy: chi-square",
            len(sepsis_healthy),
            chi_square,
            f"p = {p_value:.1e}; healthy scanned on "
            f"{int((date_by_group['healthy'] > 0).sum())} "
            f"of {len(date_by_group)} dates",
        )
    )
    sepsis_noninfected = groups[
        groups["group"].isin(["sepsis", "noninfected_icu"])
    ]
    chi_square, p_value = _chi_square(sepsis_noninfected)
    rows.append(
        _row(
            "scan date vs group, sepsis/non-infected: chi-square",
            len(sepsis_noninfected),
```

```python
            chi_square,
            f"p = {p_value:.4f}",
        )
    )

    sepsis = groups[groups["group"] == "sepsis"]
    for name, genes in gene_sets.items():
        aurocs = [
            _cross_validated_auroc(
                expression.loc[sepsis.index, genes].values,
                (sepsis["scan_date"] == date).astype(int).values,
            )
            for date, count in sepsis["scan_date"].value_counts().items()
            if count >= MINIMUM_SAMPLES_PER_SCAN_DATE
        ]
        rows.append(
            _row(
                f"{name} predict scan date among sepsis patients: "
                "mean one-vs-rest CV AUROC",
                len(sepsis),
                np.mean(aurocs),
                f"range {min(aurocs):.3f} to {max(aurocs):.3f} "
                f"over {len(aurocs)} dates",
            )
        )

    labels = (sepsis_healthy["group"] == "sepsis").astype(int)
    rows.append(
        _row(
            "locked signature, sepsis vs healthy, all scan dates "
            "(in-sample AUROC)",
            len(sepsis_healthy),
            roc_auc_score(labels, probabilities[sepsis_healthy.index]),
        )
    )
    shared_dates = sepsis_healthy[
        sepsis_healthy["scan_date"].isin(healthy_dates)
    ]
    labels = (shared_dates["group"] == "sepsis").astype(int)
    rows.append(
        _row(
            "locked signature, sepsis vs healthy, healthy-scan dates only "
            "(in-sample AUROC)",
            len(shared_dates),
            roc_auc_score(labels, probabilities[shared_dates.index]),
            f"{len(healthy_dates)} scan dates",
            n_cases=int(labels.sum()),
            n_controls=int((1 - labels).sum()),
        )
    )
    return rows


def _chi_square(groups: pd.DataFrame) -> tuple[float, float]:
    chi_square, p_value, _, _ = stats.chi2_contingency(
        pd.crosstab(groups["scan_date"], groups["group"])
    )
    return chi_square, p_value


def substudy_rows(
    groups: pd.DataFrame, expression: pd.DataFrame, gene_sets: dict
) -> list[dict]:
    rows = []
    cohort = groups[groups["group"].isin(["sepsis", "noninfected_icu"])]
    for substudy in ("pneumonia", "abdominal"):
        members = cohort[cohort["substudy"] == substudy]
        rows.append(
            _row(
                f"sepsis prevalence, {substudy} sub-study",
                len(members),
                (members["group"] == "sepsis").mean(),
                n_cases=int((members["group"] == "sepsis").sum()),
                n_controls=int((members["group"] != "sepsis").sum()),
            )
        )
```

```
    is_pneumonia = (cohort["substudy"] == "pneumonia").astype(int).values
    for name, genes in gene_sets.items():
        for group, description in (
            ("sepsis", "sepsis patients"),
            ("noninfected_icu", "non-infected patients"),
        ):
            in_group = (cohort["group"] == group).values
            rows.append(
                _row(
                    f"{name} predict sub-study among {description}: CV AUROC",
                    in_group.sum(),
                    _cross_validated_auroc(
                        expression.loc[cohort.index[in_group], genes].values,
                        is_pneumonia[in_group],
                    ),
                )
            )
    rows.append(
        _row(
            "sub-study label alone as a predictor of sepsis: AUROC",
            len(cohort),
            roc_auc_score(
                (cohort["group"] == "sepsis").astype(int), is_pneumonia
            ),
            "0.5 would mean the sub-study carries no outcome information",
        )
    )
    return rows


def cross_fitted_substudy_rows(groups: pd.DataFrame) -> list[dict]:
    predictions = pd.read_csv(
        TABLES_DIR / "specificity_crossfit_predictions.csv", index_col=0
    ).join(groups[["substudy"]])
    score_column = "p_crossfit_within_fold_rank"
    rows = [
        _row(
            "cross-fitted AUROC, both sub-studies pooled (as reported)",
            len(predictions),
            roc_auc_score(predictions["y_true"], predictions[score_column]),
        )
    ]
    weighted_sum = total_weight = 0.0
    for substudy in ("pneumonia", "abdominal"):
        members = predictions[predictions["substudy"] == substudy]
        auroc = roc_auc_score(members["y_true"], members[score_column])
        pair_count = members["y_true"].sum() * (1 - members["y_true"]).sum()
        weighted_sum, total_weight = (
            weighted_sum + pair_count * auroc,
            total_weight + pair_count,
        )
        rows.append(
            _row(
                f"cross-fitted AUROC, {substudy} sub-study only",
                len(members),
                auroc,
                n_cases=int(members["y_true"].sum()),
                n_controls=int((1 - members["y_true"]).sum()),
            )
        )
    rows.append(
        _row(
            "cross-fitted AUROC, sub-study-stratified "
            "(weighted by case-control pairs)",
            len(predictions),
            weighted_sum / total_weight,
            "within-sub-study comparisons only",
        )
    )
    return rows


def _cross_validated_auroc(
    features: np.ndarray, labels: np.ndarray, seed: int = RANDOM_SEED
) -> float:
    model = make_pipeline(
```

```python
        StandardScaler(), LogisticRegression(C=0.1, max_iter=2000)
    )
    folds = StratifiedKFold(n_splits=5, shuffle=True, random_state=seed)
    probabilities = cross_val_predict(
        model, features, labels, cv=folds, method="predict_proba"
    )[:, 1]
    return float(roc_auc_score(labels, probabilities))


def _row(
    check: str,
    sample_count: int,
    value: float,
    note: str = "",
    n_cases: int | None = None,
    n_controls: int | None = None,
) -> dict:
    return {
        "check": check,
        "n": int(sample_count),
        "n_cases": n_cases,
        "n_controls": n_controls,
        "value": round(float(value), 4),
        "value_pct": (
            round(100 * float(value), 1) if float(value) <= 1 else None
        ),
        "note": note,
    }


if __name__ == "__main__":
    main()
```

18 src/external_validation.py

```python
from __future__ import annotations

import json
import logging

import matplotlib.pyplot as plt
import numpy as np
import pandas as pd
from sklearn.calibration import calibration_curve
from sklearn.metrics import brier_score_loss, roc_auc_score, roc_curve

import formatting
import plotting
from auroc_inference import auroc_report
from config import (
    FIGURES_DIR,
    METRICS_DIR,
    PROCESSED_DATA_DIR,
    TABLES_DIR,
    configure_logging,
)
from scoring import load_locked_model, predict_probability

logger = logging.getLogger(__name__)

CI_NOTE = (
    "The discovery interval abuts the AUROC ceiling of 1.0 because "
    "sepsis-vs-healthy separation is near-complete on whole-blood "
    "transcriptome, which is a generic critical-illness effect rather than "
    "evidence of a well-powered or gene-specific estimate; its width is "
    "uninformative about precision. The validation AUROC is exactly 1.000, "
    "at which point the DeLong variance is zero and the subject-level "
    "bootstrap is degenerate, so NO two-sided interval is estimable and none "
    "is reported; a conservative one-sided lower bound is given instead. For "
    "the informative test on this cohort see the random-gene-panel benchmark "
    "and the label-permutation test."
)

```

```python

def main() -> None:
    configure_logging()
    locked_model = load_locked_model()
    expression, labels = load_validation_cohort(locked_model)
    probabilities = predict_probability(expression, locked_model)
    auroc = roc_auc_score(labels, probabilities)
    brier = brier_score_loss(labels, probabilities)
    report = auroc_report(
        labels, probabilities, bootstrap_replicates=2000, seed=42
    )
    log_validation_report(report)

    with open(
        METRICS_DIR / "discovery_nested_cv_summary.json", encoding="utf-8"
    ) as summary_file:
        discovery_summary = json.load(summary_file)
    log_comparison(discovery_summary, auroc, brier, report)

    plot_validation_figure(labels, probabilities, auroc)
    write_performance_table(discovery_summary, labels, auroc, brier, report)
    write_summary(discovery_summary, labels, auroc, brier, report)
    pd.DataFrame(
        {
            "geo_accession": expression.index,
            "y_true": labels,
            "p_signature": probabilities,
        }
    ).to_csv(PROCESSED_DATA_DIR / "validation_predictions.csv", index=False)


def load_validation_cohort(
    locked_model: dict,
) -> tuple[pd.DataFrame, np.ndarray]:
    gene_order = locked_model["gene_names_order"]
    expression = pd.read_csv(
        PROCESSED_DATA_DIR / "validation_expr_gene.csv.gz",
        index_col=0,
        usecols=["geo_accession", *gene_order],
    ).astype(np.float32)
    cohort = pd.read_csv(
        PROCESSED_DATA_DIR / "validation_labels.csv", index_col=0
    )
    expression = expression.loc[cohort.index]
    labels = (cohort["group"] == "sepsis").astype(int).to_numpy()
    logger.info(
        "validation cohort: n=%d (%d sepsis, %d healthy)",
        len(labels),
        labels.sum(),
        (1 - labels).sum(),
    )
    return expression, labels


def log_validation_report(report: dict) -> None:
    degenerate = (
        " (degenerate: complete separation)"
        if report["delong"]["degenerate"]
        else ""
    )
    logger.info(
        "[validation] AUROC %.4f; DeLong SE %.4f%s",
        report["auroc"],
        report["delong"]["se"],
        degenerate,
    )
    logger.info(
        "[validation] stratified subject bootstrap: %.4f-%.4f%s",
        report["bootstrap"]["ci_lo"],
        report["bootstrap"]["ci_hi"],
        " (degenerate)" if report["bootstrap"]["degenerate"] else "",
    )
    logger.info(
        "[validation] reported: %s, lower bound %.4f",
        report["reported_ci_method"],
        report["reported_ci_lo"],
```

```python
    )
    logger.info(
        "[validation] score margin: max healthy %.4f, min sepsis %.4f, "
        "gap %.4f",
        report["max_control_score"],
        report["min_case_score"],
        report["score_margin"],
    )


def log_comparison(
    discovery_summary: dict, auroc: float, brier: float, report: dict
) -> None:
    logger.info(
        "discovery (nested CV, pooled OOF) AUROC: %.4f (%.4f-%.4f)",
        discovery_summary["pooled_oof_auroc"],
        discovery_summary["pooled_oof_auroc_ci_lo"],
        discovery_summary["pooled_oof_auroc_ci_hi"],
    )
    logger.info(
        "validation (external, locked model) AUROC: %.4f "
        "[%s, lower bound %.4f]",
        auroc,
        report["reported_ci_method"],
        report["reported_ci_lo"],
    )
    logger.info(
        "gap (discovery - validation): %.4f",
        discovery_summary["pooled_oof_auroc"] - auroc,
    )
    logger.info("validation Brier score: %.4f", brier)


def plot_validation_figure(
    labels: np.ndarray, probabilities: np.ndarray, auroc: float
) -> None:
    plotting.apply_style()
    fig, axes = plt.subplots(
        1, 2, figsize=(plotting.FULL_WIDTH_INCHES * 2, 4.2)
    )

    false_positive_rate, true_positive_rate, _ = roc_curve(
        labels, probabilities
    )
    axes[0].plot(
        false_positive_rate,
        true_positive_rate,
        color=plotting.PALETTE["orange"],
        linestyle="-",
        label=f"Validation (AUROC {formatting.format_number(auroc, 'auroc')})",
    )
    axes[0].plot(
        [0, 1],
        [0, 1],
        color=plotting.PALETTE["gray"],
        linestyle="--",
        label="Chance",
    )
    axes[0].set_xlabel("False positive rate")
    axes[0].set_ylabel("True positive rate")
    axes[0].set_ylim(-0.03, 1.12)
    axes[0].legend(loc="lower right")
    plotting.label_panel(axes[0], "A")

    observed_fraction, mean_predicted = calibration_curve(
        labels, probabilities, n_bins=5, strategy="quantile"
    )
    axes[1].plot(
        mean_predicted,
        observed_fraction,
        color=plotting.PALETTE["blue"],
        linestyle="-",
        marker="o",
        label="Validation",
    )
    axes[1].plot(
```

```python
        [0, 1],
        [0, 1],
        color=plotting.PALETTE["gray"],
        linestyle="--",
        label="Perfect calibration",
    )
    axes[1].set_xlabel("Mean predicted probability")
    axes[1].set_ylabel("Observed sepsis fraction")
    axes[1].set_ylim(-0.03, 1.12)
    axes[1].legend(loc="lower right")
    plotting.label_panel(axes[1], "B")

    plotting.save_figure(fig, FIGURES_DIR, "fig5_validation_roc_calibration")
    plt.close(fig)


def write_performance_table(
    discovery_summary: dict,
    labels: np.ndarray,
    auroc: float,
    brier: float,
    report: dict,
) -> None:
    rows = [
        {
            "cohort": "Discovery (nested CV, pooled OOF)",
            "n": discovery_summary["n_discovery"],
            "auroc": discovery_summary["pooled_oof_auroc"],
            "ci_method": "percentile bootstrap on frozen out-of-fold "
            "predictions (excludes selection uncertainty; see "
            "pipeline_bootstrap.py for the interval that includes it)",
            "ci_lo": discovery_summary["pooled_oof_auroc_ci_lo"],
            "ci_hi": discovery_summary["pooled_oof_auroc_ci_hi"],
            "brier": np.nan,
            "ci_note": CI_NOTE,
        },
        {
            "cohort": "Validation (external, GSE57065)",
            "n": len(labels),
            "auroc": round(float(auroc), 4),
            "ci_method": "no two-sided interval estimable at AUROC 1.000; "
            f"{report['reported_ci_method']}",
            "ci_lo": round(float(report["reported_ci_lo"]), 4),
            "ci_hi": np.nan,
            "brier": round(float(brier), 4),
            "ci_note": CI_NOTE,
        },
    ]
    pd.DataFrame(rows).to_csv(
        TABLES_DIR / "discovery_vs_validation_performance.csv", index=False
    )


def write_summary(
    discovery_summary: dict,
    labels: np.ndarray,
    auroc: float,
    brier: float,
    report: dict,
) -> None:
    discovery_auroc = discovery_summary["pooled_oof_auroc"]
    summary = {
        "auroc_discovery": discovery_auroc,
        "ci_discovery": [
            discovery_summary["pooled_oof_auroc_ci_lo"],
            discovery_summary["pooled_oof_auroc_ci_hi"],
        ],
        "ci_discovery_note": (
            "resamples frozen out-of-fold predictions; the interval that "
            "repeats the whole selection-and-fit pipeline is in "
            "pipeline_bootstrap_summary.json"
        ),
        "auroc_validation": round(float(auroc), 4),
        "validation_delong_se": round(report["delong"]["se"], 6),
        "validation_delong_degenerate": report["delong"]["degenerate"],
        "validation_bootstrap_ci": [
```

```
            round(report["bootstrap"]["ci_lo"], 4),
            round(report["bootstrap"]["ci_hi"], 4),
        ],
        "validation_bootstrap_degenerate": report["bootstrap"]["degenerate"],
        "validation_reported_ci_method": report["reported_ci_method"],
        "validation_one_sided_lower_bound": round(
            float(report["reported_ci_lo"]), 4
        ),
        "validation_rule_of_three_m": report["rule_of_three"][
            "m_independent_pairs"
        ],
        "wilson_interval_not_used": (
            "A Wilson interval over the 700 case-control pairs treated as "
            "independent gives 0.995-1.000. The pairs share subjects and are "
            "not independent, so that interval is too narrow and is not "
            "reported."
        ),
        "gap": round(float(discovery_auroc - auroc), 4),
        "brier_validation": round(float(brier), 4),
        "n_validation": int(len(labels)),
        "separation_max_healthy_score": round(report["max_control_score"], 4),
        "separation_min_sepsis_score": round(report["min_case_score"], 4),
        "separation_score_margin": round(report["score_margin"], 4),
        "separation_note": (
            "the score distributions are disjoint with a positive margin "
            "and no tied or saturated values, so the AUROC of 1.000 is "
            "genuine separation rather than a numerical artifact; that is "
            "why no interval is estimable, not a reason to invent one"
        ),
        "ci_interpretation_note": CI_NOTE,
    }
    with open(
        METRICS_DIR / "external_validation_summary.json", "w", encoding="utf-8"
    ) as summary_file:
        json.dump(summary, summary_file, indent=2)
    logger.info("%s", summary)


if __name__ == "__main__":
    main()
```

19 src/random_panel_benchmark.py

```
from __future__ import annotations

import hashlib
import json
import logging
import time
from dataclasses import dataclass
from typing import NamedTuple

import numpy as np
import pandas as pd
from scipy import stats
from sklearn.linear_model import LogisticRegression
from sklearn.metrics import roc_auc_score
from sklearn.preprocessing import StandardScaler

from config import (
    METRICS_DIR,
    PANEL_SIZES,
    PROCESSED_DATA_DIR,
    RANDOM_PANEL_DRAWS,
    RANDOM_SEED,
    TABLES_DIR,
    configure_logging,
)
from scoring import load_locked_model, predict_probability

logger = logging.getLogger(__name__)

RECIPES = ("l1_modalC", "unpenalized")
```

```python
POOLS = ("transportable", "top300_candidate")
PRIMARY_POOL = "transportable"
PRIMARY_RECIPE = "l1_modalC"

Tasks = dict[str, tuple[np.ndarray, np.ndarray]]


@dataclass
class BenchmarkData:

    training_features: np.ndarray
    training_labels: np.ndarray
    tasks: Tasks
    validation_expression: pd.DataFrame
    specificity_expression: pd.DataFrame


class SelectedPanel(NamedTuple):

    gene_indices: np.ndarray
    c_value: float
    nonzero_count: int
    selection_mode: str


def main() -> None:
    configure_logging()
    start_time = time.time()
    locked_model = load_locked_model()
    gene_order = locked_model["gene_names_order"]
    signature_genes = locked_model["signature_genes"]
    modal_c = float(locked_model["modal_C"])
    signature_size = len(signature_genes)
    logger.info(
        "locked signature: k=%d genes, modal C=%s", signature_size, modal_c
    )

    data = load_benchmark_data(gene_order)
    gene_names = np.array(gene_order)
    signature_indices = np.array(
        [int(np.where(gene_names == gene)[0][0]) for gene in signature_genes]
    )
    candidate_indices = np.array(locked_model["candidate_idx_final"])
    pools = {
        "transportable": np.arange(len(gene_order)),
        "top300_candidate": candidate_indices,
    }

    locked_aurocs = locked_model_aurocs(locked_model, data)
    real_panels = resize_signature_panels(
        data, candidate_indices, signature_indices, modal_c, signature_size
    )
    real_aurocs = refit_real_panels(data, real_panels, modal_c)

    rows, null_distributions, primary_panels = run_benchmark_grid(
        data,
        pools,
        benchmark_cells(signature_size),
        real_panels,
        real_aurocs,
        locked_aurocs,
        modal_c,
        signature_size,
        gene_names,
    )
    results = write_benchmark_tables(
        rows, null_distributions, primary_panels, signature_size, data.tasks
    )
    summary = benchmark_summary(
        results,
        signature_size,
        modal_c,
        len(gene_order),
        len(candidate_indices),
        locked_aurocs,
        start_time,
```

```
    )
    with open(
        METRICS_DIR / "random_panel_benchmark_summary.json",
        "w",
        encoding="utf-8",
    ) as summary_file:
        json.dump(summary, summary_file, indent=2)
    logger.info("%s", json.dumps(summary["primary_cell"], indent=2))
    logger.info("total runtime %ss", summary["runtime_s"])


def load_benchmark_data(gene_order: list[str]) -> BenchmarkData:
    columns = ["geo_accession", *gene_order]
    discovery = pd.read_csv(
        PROCESSED_DATA_DIR / "discovery_expr_gene.csv.gz",
        index_col=0,
        usecols=columns,
    ).astype(np.float32)
    discovery_labels = pd.read_csv(
        PROCESSED_DATA_DIR / "discovery_labels.csv", index_col=0
    )
    validation = pd.read_csv(
        PROCESSED_DATA_DIR / "validation_expr_gene.csv.gz",
        index_col=0,
        usecols=columns,
    ).astype(np.float32)
    validation_labels = pd.read_csv(
        PROCESSED_DATA_DIR / "validation_labels.csv", index_col=0
    )

    training = discovery_labels[
        discovery_labels["group"].isin(["sepsis", "healthy"])
    ]
    training_features = discovery.loc[training.index, gene_order].values
    training_labels = (training["group"] == "sepsis").astype(int).values

    validation = validation.loc[validation_labels.index, gene_order]
    validation_outcome = (
        (validation_labels["group"] == "sepsis").astype(int).values
    )

    specificity = discovery_labels[
        discovery_labels["group"].isin(["sepsis", "noninfected_icu"])
    ]
    specificity_expression = discovery.loc[specificity.index, gene_order]
    specificity_outcome = (specificity["group"] == "sepsis").astype(int).values

    tasks = {
        "validation": (validation.values, validation_outcome),
        "specificity": (specificity_expression.values, specificity_outcome),
    }
    logger.info(
        "train n=%d; validation n=%d; specificity n=%d",
        len(training_labels),
        len(validation_outcome),
        len(specificity_outcome),
    )
    return BenchmarkData(
        training_features,
        training_labels,
        tasks,
        validation,
        specificity_expression,
    )


def locked_model_aurocs(
    locked_model: dict, data: BenchmarkData
) -> dict[str, float]:
    validation_probability = predict_probability(
        data.validation_expression, locked_model
    )
    specificity_probability = predict_probability(
        data.specificity_expression, locked_model
    )
    aurocs = {
```

```python
        "validation": float(
            roc_auc_score(data.tasks["validation"][1], validation_probability)
        ),
        "specificity": float(
            roc_auc_score(
                data.tasks["specificity"][1], specificity_probability
            )
        ),
    }
    logger.info(
        "locked model (no refit): validation %.4f, specificity %.4f",
        aurocs["validation"],
        aurocs["specificity"],
    )
    return aurocs


def resize_signature_panels(
    data: BenchmarkData,
    candidate_indices: np.ndarray,
    signature_indices: np.ndarray,
    modal_c: float,
    signature_size: int,
) -> dict[int, SelectedPanel]:
    panels = {
        signature_size: SelectedPanel(
            signature_indices, modal_c, signature_size, "locked_signature"
        )
    }
    for panel_size in PANEL_SIZES:
        if panel_size in panels:
            continue
        panel = _select_k_gene_panel(
            data.training_features,
            data.training_labels,
            candidate_indices,
            panel_size,
        )
        panels[panel_size] = panel
        logger.info(
            "  resized panel k=%d: C=%.4g, %d non-zero, mode=%s",
            panel_size,
            panel.c_value,
            panel.nonzero_count,
            panel.selection_mode,
        )
    return panels


def _select_k_gene_panel(
    training_features: np.ndarray,
    training_labels: np.ndarray,
    candidate_indices: np.ndarray,
    panel_size: int,
    seed: int = RANDOM_SEED,
) -> SelectedPanel:
    scaler = StandardScaler().fit(training_features[:, candidate_indices])
    standardized = scaler.transform(training_features[:, candidate_indices])
    widest: tuple[float, np.ndarray, np.ndarray] | None = None
    widest_count = -1
    for c_value in np.logspace(-3, 4, 80):
        model = LogisticRegression(
            penalty="l1",
            solver="liblinear",
            C=float(c_value),
            max_iter=5000,
            random_state=seed,
        ).fit(standardized, training_labels)
        coefficients = model.coef_.ravel()
        nonzero = np.flatnonzero(coefficients)
        if len(nonzero) > widest_count:
            widest = (float(c_value), nonzero, coefficients)
            widest_count = len(nonzero)
        if len(nonzero) >= panel_size:
            strongest = nonzero[
                np.argsort(-np.abs(coefficients[nonzero]))[:panel_size]
```

```
            ]
            return SelectedPanel(
                candidate_indices[strongest],
                float(c_value),
                int(len(nonzero)),
                "l1_only",
            )

    if widest is None:
        raise RuntimeError("the C search grid is empty")
    c_value, nonzero, coefficients = widest
    chosen = list(nonzero[np.argsort(-np.abs(coefficients[nonzero]))])
    t_statistics, _ = stats.ttest_ind(
        standardized[training_labels == 1],
        standardized[training_labels == 0],
        axis=0,
        equal_var=False,
    )
    for column in np.argsort(-np.abs(t_statistics)):
        if len(chosen) >= panel_size:
            break
        if column not in chosen:
            chosen.append(int(column))
    return SelectedPanel(
        candidate_indices[np.array(chosen[:panel_size])],
        c_value,
        int(len(nonzero)),
        "l1_plus_t_filter",
    )


def refit_real_panels(
    data: BenchmarkData, real_panels: dict[int, SelectedPanel], modal_c: float
) -> dict[tuple[int, str], dict[str, float]]:
    aurocs = {
        (panel_size, recipe): fit_and_score(
            data.training_features,
            data.training_labels,
            real_panels[panel_size].gene_indices,
            data.tasks,
            recipe,
            modal_c,
            RANDOM_SEED,
        )
        for panel_size in real_panels
        for recipe in RECIPES
    }
    for (panel_size, recipe), task_aurocs in sorted(aurocs.items()):
        logger.info(
            "  real k=%d [%s]: validation %.4f, specificity %.4f",
            panel_size,
            recipe,
            task_aurocs["validation"],
            task_aurocs["specificity"],
        )
    return aurocs


def fit_and_score(
    training_features: np.ndarray,
    training_labels: np.ndarray,
    gene_indices: np.ndarray,
    tasks: Tasks,
    recipe: str,
    modal_c: float,
    seed: int,
) -> dict[str, float]:
    scaler = StandardScaler().fit(training_features[:, gene_indices])
    standardized = scaler.transform(training_features[:, gene_indices])
    model = _make_model(recipe, modal_c, seed).fit(
        standardized, training_labels
    )
    aurocs = {}
    for task, (features, outcome) in tasks.items():
        probability = model.predict_proba(
            scaler.transform(features[:, gene_indices])
```

```python
        )[:, 1]
        aurocs[task] = roc_auc_score(outcome, probability)
    return aurocs


def _make_model(recipe: str, modal_c: float, seed: int) -> LogisticRegression:
    if recipe == "l1_modalC":
        return LogisticRegression(
            penalty="l1",
            solver="liblinear",
            C=modal_c,
            max_iter=2000,
            random_state=seed,
        )
    if recipe == "unpenalized":
        return LogisticRegression(
            penalty=None, solver="lbfgs", max_iter=5000, random_state=seed
        )
    raise ValueError(recipe)


def benchmark_cells(signature_size: int) -> list[tuple[str, int, str]]:
    cells = [(PRIMARY_POOL, size, PRIMARY_RECIPE) for size in PANEL_SIZES]
    for pool in POOLS:
        for recipe in RECIPES:
            cell = (pool, signature_size, recipe)
            if cell not in cells:
                cells.append(cell)
    return cells


def run_benchmark_grid(
    data: BenchmarkData,
    pools: dict[str, np.ndarray],
    cells: list[tuple[str, int, str]],
    real_panels: dict[int, SelectedPanel],
    real_aurocs: dict[tuple[int, str], dict[str, float]],
    locked_aurocs: dict[str, float],
    modal_c: float,
    signature_size: int,
    gene_names: np.ndarray,
) -> tuple[list[dict], dict[str, np.ndarray], list[str]]:
    rows: list[dict] = []
    null_distributions: dict[str, np.ndarray] = {}
    primary_panels: list[str] = []
    for pool_name, panel_size, recipe in cells:
        tag = f"{pool_name}|k={panel_size}|{recipe}"
        pool_indices = pools[pool_name]
        if panel_size > len(pool_indices):
            logger.info(
                "  skip %s: pool has only %d genes", tag, len(pool_indices)
            )
            continue
        cell_start = time.time()
        null_aurocs, audit, panel_strings = _run_cell(
            data,
            pool_indices,
            panel_size,
            recipe,
            modal_c,
            gene_names,
        )
        real_panel = real_panels[panel_size]
        for task in data.tasks:
            rows.append(
                _null_summary_row(
                    (pool_name, panel_size, recipe, task),
                    null_aurocs[task],
                    real_aurocs[(panel_size, recipe)][task],
                    real_panel,
                    gene_names,
                    (
                        locked_aurocs[task]
                        if panel_size == signature_size
                        else None
                    ),
```

```python
                    audit,
                )
            )
            key = f"{pool_name}|{panel_size}|{recipe}|{task}"
            null_distributions[key] = null_aurocs[task]
        logger.info(
            "  %s: done in %.1fs (dupes %d)",
            tag,
            time.time() - cell_start,
            audit["n_redundant_draws"],
        )
        if (pool_name, panel_size, recipe) == (
            PRIMARY_POOL,
            signature_size,
            PRIMARY_RECIPE,
        ):
            primary_panels = panel_strings
    return rows, null_distributions, primary_panels


def _run_cell(
    data: BenchmarkData,
    pool_indices: np.ndarray,
    panel_size: int,
    recipe: str,
    modal_c: float,
    gene_names: np.ndarray,
) -> tuple[dict[str, np.ndarray], dict, list[str]]:
    random_generator = np.random.default_rng(RANDOM_SEED)
    draws = np.empty((RANDOM_PANEL_DRAWS, panel_size), dtype=int)
    null_aurocs = {task: np.empty(RANDOM_PANEL_DRAWS) for task in data.tasks}
    for draw in range(RANDOM_PANEL_DRAWS):
        gene_indices = random_generator.choice(
            pool_indices, size=panel_size, replace=False
        )
        draws[draw] = gene_indices
        task_aurocs = fit_and_score(
            data.training_features,
            data.training_labels,
            gene_indices,
            data.tasks,
            recipe,
            modal_c,
            RANDOM_SEED + draw + 1,
        )
        for task, auroc in task_aurocs.items():
            null_aurocs[task][draw] = auroc

    _, counts = np.unique(np.sort(draws, axis=1), axis=0, return_counts=True)
    panel_strings = [";".join(sorted(gene_names[panel])) for panel in draws]
    audit = {
        "n_duplicate_panels": int((counts > 1).sum()),
        "n_redundant_draws": int((counts[counts > 1] - 1).sum()),
        "panel_list_sha256": hashlib.sha256(
            "\n".join(panel_strings).encode()
        ).hexdigest(),
    }
    return null_aurocs, audit, panel_strings


def _null_summary_row(
    cell: tuple[str, int, str, str],
    null_aurocs: np.ndarray,
    real_auroc: float,
    real_panel: SelectedPanel,
    gene_names: np.ndarray,
    locked_auroc: float | None,
    audit: dict,
) -> dict:
    pool_name, panel_size, recipe, task = cell
    random_at_least_real = int(np.sum(null_aurocs >= real_auroc))
    empirical_p = (random_at_least_real + 1) / (RANDOM_PANEL_DRAWS + 1)
    proportion_low, proportion_high = _clopper_pearson(
        random_at_least_real, RANDOM_PANEL_DRAWS
    )
    return {
```

```python
        "pool": pool_name,
        "panel_size": panel_size,
        "recipe": recipe,
        "task": task,
        "real_auroc_refit": round(float(real_auroc), 4),
        "real_panel_is_locked_signature": locked_auroc is not None,
        "real_panel_selection_C": round(real_panel.c_value, 5),
        "real_panel_selection_mode": real_panel.selection_mode,
        "real_panel_genes": ";".join(
            sorted(gene_names[real_panel.gene_indices])
        ),
        "locked_model_auroc": (
            round(locked_auroc, 4) if locked_auroc is not None else None
        ),
        "null_mean": round(float(null_aurocs.mean()), 4),
        "null_sd": round(float(null_aurocs.std(ddof=1)), 4),
        "null_p05": round(float(np.percentile(null_aurocs, 5)), 4),
        "null_median": round(float(np.median(null_aurocs)), 4),
        "null_p95": round(float(np.percentile(null_aurocs, 95)), 4),
        "null_max": round(float(null_aurocs.max()), 4),
        "n_draws": RANDOM_PANEL_DRAWS,
        "n_random_ge_real": random_at_least_real,
        "prop_random_ge_real": round(
            random_at_least_real / RANDOM_PANEL_DRAWS, 4
        ),
        "prop_ci_lo": round(proportion_low, 4),
        "prop_ci_hi": round(proportion_high, 4),
        "empirical_p": round(float(empirical_p), 5),
        "n_duplicate_panels": audit["n_duplicate_panels"],
        "n_redundant_draws": audit["n_redundant_draws"],
        "panel_list_sha256": audit["panel_list_sha256"],
    }


def _clopper_pearson(
    successes: int, trials: int, confidence: float = 0.95
) -> tuple[float, float]:
    alpha = 1 - confidence
    lower = (
        0.0
        if successes == 0
        else stats.beta.ppf(alpha / 2, successes, trials - successes + 1)
    )
    upper = (
        1.0
        if successes == trials
        else stats.beta.ppf(1 - alpha / 2, successes + 1, trials - successes)
    )
    return float(lower), float(upper)


def write_benchmark_tables(
    rows: list[dict],
    null_distributions: dict[str, np.ndarray],
    primary_panels: list[str],
    signature_size: int,
    tasks: Tasks,
) -> pd.DataFrame:
    results = pd.DataFrame(rows)
    results.to_csv(TABLES_DIR / "random_panel_benchmark.csv", index=False)
    logger.info(
        "\n%s",
        results[
            [
                "pool",
                "panel_size",
                "recipe",
                "task",
                "real_auroc_refit",
                "null_mean",
                "prop_random_ge_real",
                "empirical_p",
            ]
        ].to_string(index=False),
    )
    pd.DataFrame(
```

```python
        {
            "draw": np.arange(1, len(primary_panels) + 1),
            "genes": primary_panels,
        }
    ).to_csv(
        TABLES_DIR / "random_panel_draws_primary.csv.gz",
        index=False,
        compression="gzip",
    )
    for task in tasks:
        key = f"{PRIMARY_POOL}|{signature_size}|{PRIMARY_RECIPE}|{task}"
        pd.Series(null_distributions[key], name="null_auroc").to_csv(
            PROCESSED_DATA_DIR / f"random_panel_null_{task}.csv", index=False
        )
    return results


def benchmark_summary(
    results: pd.DataFrame,
    signature_size: int,
    modal_c: float,
    transportable_count: int,
    candidate_count: int,
    locked_aurocs: dict[str, float],
    start_time: float,
) -> dict:
    primary = results[
        (results["pool"] == PRIMARY_POOL)
        & (results["panel_size"] == signature_size)
        & (results["recipe"] == PRIMARY_RECIPE)
    ]
    return {
        "what_this_tests": (
            "Whether the selected k-gene panel outperforms random k-gene "
            "panels drawn from a stated pool, with the modeling recipe held "
            "identical. It is not a test of classification against chance; "
            "see label_permutation_test.py."
        ),
        "signature_size": signature_size,
        "signature_size_origin": (
            "data-determined: the number of non-zero coefficients returned "
            "by the L1 fit at the modal outer-fold C, not a pre-specified "
            "target"
        ),
        "modal_C": modal_c,
        "recipes": {
            "l1_modalC": (
                "LogisticRegression(penalty='l1', solver='liblinear', "
                f"C={modal_c}, max_iter=2000)"
            ),
            "unpenalized": (
                "LogisticRegression(penalty=None, solver='lbfgs', "
                "max_iter=5000)"
            ),
        },
        "pools": {
            "transportable": int(transportable_count),
            "top300_candidate": int(candidate_count),
        },
        "panel_sizes_swept": list(PANEL_SIZES),
        "n_draws_per_cell": RANDOM_PANEL_DRAWS,
        "locked_model_auroc": {
            task: round(auroc, 4) for task, auroc in locked_aurocs.items()
        },
        "primary_cell": primary.to_dict(orient="records"),
        "runtime_s": round(time.time() - start_time, 1),
    }


if __name__ == "__main__":
    main()
```

20 src/label_permutation_test.py

```python
from __future__ import annotations

import json
import logging
import time

import matplotlib.pyplot as plt
import numpy as np
import pandas as pd
from scipy import stats
from sklearn.linear_model import LogisticRegression
from sklearn.metrics import roc_auc_score
from sklearn.preprocessing import StandardScaler

import plotting
from config import (
    FIGURES_DIR,
    LABEL_PERMUTATIONS,
    METRICS_DIR,
    PROCESSED_DATA_DIR,
    RANDOM_SEED,
    TABLES_DIR,
    configure_logging,
)
from scoring import load_locked_model

logger = logging.getLogger(__name__)

CANDIDATE_POOL_SIZE = 300
TASK_AXIS_LABELS = {
    "validation": "External validation AUROC",
    "specificity": "Sepsis vs. non-infected ICU AUROC",
}

Tasks = dict[str, tuple[np.ndarray, np.ndarray]]


def main() -> None:
    configure_logging()
    start_time = time.time()
    locked_model = load_locked_model()
    gene_order = locked_model["gene_names_order"]
    modal_c = float(locked_model["modal_C"])

    training_features, training_labels, tasks = load_permutation_data(
        gene_order
    )
    observed, _ = fit_pipeline_and_score(
        training_features, training_labels, tasks, modal_c, RANDOM_SEED
    )
    null_aurocs, nonzero_counts = permutation_null(
        training_features, training_labels, tasks, modal_c, start_time
    )
    rows = write_permutation_table(observed, null_aurocs, tasks)

    summary = {
        "what_this_tests": (
            "Whether the full selection-and-fit pipeline extracts more label "
            "information than chance. Class labels ARE permuted here, and the "
            "top-300 filter is re-derived inside every permutation. Distinct "
            "from the random-gene-panel benchmark, which holds labels fixed "
            "and varies the gene set."
        ),
        "null_hypothesis": (
            "the discovery sepsis/healthy label vector is exchangeable with "
            "respect to the expression matrix"
        ),
        "n_permutations": LABEL_PERMUTATIONS,
        "recipe": (
            f"top-{CANDIDATE_POOL_SIZE} |Welch t| filter re-derived per "
            "permutation, then LogisticRegression(penalty='l1', "
            f"solver='liblinear', C={modal_c})"
        ),
        "results": rows,
```

```python
        "null_nonzero_genes_mean": round(float(nonzero_counts.mean()), 2),
        "runtime_s": round(time.time() - start_time, 1),
    }
    with open(
        METRICS_DIR / "label_permutation_summary.json", "w", encoding="utf-8"
    ) as summary_file:
        json.dump(summary, summary_file, indent=2)

    plot_permutation_null(null_aurocs, observed)
    logger.info("total runtime %ss", summary["runtime_s"])


def load_permutation_data(
    gene_order: list[str],
) -> tuple[np.ndarray, np.ndarray, Tasks]:
    columns = ["geo_accession", *gene_order]
    discovery = pd.read_csv(
        PROCESSED_DATA_DIR / "discovery_expr_gene.csv.gz",
        index_col=0,
        usecols=columns,
    ).astype(np.float32)
    discovery_labels = pd.read_csv(
        PROCESSED_DATA_DIR / "discovery_labels.csv", index_col=0
    )
    validation = pd.read_csv(
        PROCESSED_DATA_DIR / "validation_expr_gene.csv.gz",
        index_col=0,
        usecols=columns,
    ).astype(np.float32)
    validation_labels = pd.read_csv(
        PROCESSED_DATA_DIR / "validation_labels.csv", index_col=0
    )

    training = discovery_labels[
        discovery_labels["group"].isin(["sepsis", "healthy"])
    ]
    training_features = discovery.loc[training.index, gene_order].to_numpy()
    training_labels = (training["group"] == "sepsis").astype(int).to_numpy()

    validation = validation.loc[validation_labels.index, gene_order]
    specificity = discovery_labels[
        discovery_labels["group"].isin(["sepsis", "noninfected_icu"])
    ]
    tasks = {
        "validation": (
            validation.to_numpy(),
            (validation_labels["group"] == "sepsis").astype(int).to_numpy(),
        ),
        "specificity": (
            discovery.loc[specificity.index, gene_order].to_numpy(),
            (specificity["group"] == "sepsis").astype(int).to_numpy(),
        ),
    }
    logger.info(
        "train n=%d (%d sepsis, %d healthy); validation n=%d; "
        "specificity n=%d",
        len(training_labels),
        training_labels.sum(),
        (1 - training_labels).sum(),
        len(tasks["validation"][1]),
        len(tasks["specificity"][1]),
    )
    return training_features, training_labels, tasks


def fit_pipeline_and_score(
    training_features: np.ndarray,
    training_labels: np.ndarray,
    tasks: Tasks,
    modal_c: float,
    seed: int,
) -> tuple[dict[str, float], int]:
    candidates = _top_t_indices(
        training_features, training_labels, CANDIDATE_POOL_SIZE
    )
    scaler = StandardScaler().fit(training_features[:, candidates])
```

```python
    standardized = scaler.transform(training_features[:, candidates])
    model = LogisticRegression(
        penalty="l1",
        solver="liblinear",
        C=modal_c,
        max_iter=2000,
        random_state=seed,
    ).fit(standardized, training_labels)
    aurocs = {}
    for task, (features, outcome) in tasks.items():
        probability = model.predict_proba(
            scaler.transform(features[:, candidates])
        )[:, 1]
        aurocs[task] = roc_auc_score(outcome, probability)
    return aurocs, int(np.sum(model.coef_ != 0))


def _top_t_indices(
    features: np.ndarray, labels: np.ndarray, count: int
) -> np.ndarray:
    t_statistics, _ = stats.ttest_ind(
        features[labels == 1], features[labels == 0], axis=0, equal_var=False
    )
    t_statistics = np.nan_to_num(t_statistics, nan=0.0)
    return np.argsort(-np.abs(t_statistics))[:count]


def permutation_null(
    training_features: np.ndarray,
    training_labels: np.ndarray,
    tasks: Tasks,
    modal_c: float,
    start_time: float,
) -> tuple[dict[str, np.ndarray], np.ndarray]:
    random_generator = np.random.default_rng(RANDOM_SEED)
    null_aurocs = {task: np.empty(LABEL_PERMUTATIONS) for task in tasks}
    nonzero_counts = np.empty(LABEL_PERMUTATIONS, dtype=int)
    for permutation in range(LABEL_PERMUTATIONS):
        permuted_labels = random_generator.permutation(training_labels)
        aurocs, nonzero_count = fit_pipeline_and_score(
            training_features,
            permuted_labels,
            tasks,
            modal_c,
            RANDOM_SEED + permutation + 1,
        )
        for task in tasks:
            null_aurocs[task][permutation] = aurocs[task]
        nonzero_counts[permutation] = nonzero_count
        if (permutation + 1) % 200 == 0:
            logger.info(
                "  %d/%d permutations (%.0fs elapsed)",
                permutation + 1,
                LABEL_PERMUTATIONS,
                time.time() - start_time,
            )
    return null_aurocs, nonzero_counts


def write_permutation_table(
    observed: dict[str, float],
    null_aurocs: dict[str, np.ndarray],
    tasks: Tasks,
) -> list[dict]:
    rows = []
    for task in tasks:
        null = null_aurocs[task]
        at_least_observed = int(np.sum(null >= observed[task]))
        empirical_p = (at_least_observed + 1) / (LABEL_PERMUTATIONS + 1)
        rows.append(
            {
                "task": task,
                "observed_auroc": round(float(observed[task]), 4),
                "n_permutations": LABEL_PERMUTATIONS,
                "null_mean": round(float(null.mean()), 4),
                "null_sd": round(float(null.std(ddof=1)), 4),
```

```
                "null_p95": round(float(np.percentile(null, 95)), 4),
                "null_max": round(float(null.max()), 4),
                "n_perm_ge_observed": at_least_observed,
                "empirical_p": round(float(empirical_p), 5),
            }
        )
    table = pd.DataFrame(rows)
    table.to_csv(TABLES_DIR / "label_permutation_test.csv", index=False)
    logger.info("\n%s", table.to_string(index=False))

    for task in tasks:
        pd.Series(null_aurocs[task], name="null_auroc").to_csv(
            PROCESSED_DATA_DIR / f"label_permutation_null_{task}.csv",
            index=False,
        )
    return rows


def plot_permutation_null(
    null_aurocs: dict[str, np.ndarray], observed: dict[str, float]
) -> None:
    plotting.apply_style()
    fig, axes = plt.subplots(
        1, 2, figsize=(plotting.FULL_WIDTH_INCHES * 2, 4.2)
    )
    for ax, task, letter in zip(
        axes, ["validation", "specificity"], "AB", strict=True
    ):
        ax.hist(
            null_aurocs[task],
            bins=40,
            color=plotting.PALETTE["sky"],
            edgecolor="white",
            label="Permuted",
        )
        ax.axvline(
            observed[task],
            color=plotting.PALETTE["red"],
            linestyle="--",
            label="Observed",
        )
        ax.set_xlabel(TASK_AXIS_LABELS[task])
        ax.set_ylabel("Count")
        ax.legend(
            loc="upper left", bbox_to_anchor=(0.0, 0.93), fontsize="small"
        )
        plotting.label_panel(ax, letter)
    plotting.save_figure(fig, FIGURES_DIR, "fig2_label_permutation")
    plt.close(fig)


if __name__ == "__main__":
    main()
```

21 src/pipeline_bootstrap.py

```
from __future__ import annotations

import json
import logging
import time
from collections import Counter
from dataclasses import dataclass, field

import numpy as np
import pandas as pd
from scipy import stats
from sklearn.linear_model import LogisticRegression
from sklearn.metrics import roc_auc_score
from sklearn.model_selection import StratifiedKFold
from sklearn.preprocessing import StandardScaler

from config import (
```

```python
    INNER_FOLDS,
    METRICS_DIR,
    PIPELINE_BOOTSTRAP_REPLICATES,
    PROCESSED_DATA_DIR,
    RANDOM_SEED,
    TABLES_DIR,
    configure_logging,
)
from scoring import load_locked_model

logger = logging.getLogger(__name__)

CANDIDATE_POOL_SIZE = 300
C_GRID = [0.003, 0.01, 0.03, 0.1, 0.3, 1.0]


@dataclass
class BootstrapData:

    features: np.ndarray
    labels: np.ndarray
    validation_features: np.ndarray
    validation_labels: np.ndarray
    specificity_features: np.ndarray
    specificity_labels: np.ndarray


@dataclass
class BootstrapResults:

    out_of_bag_aurocs: list[float] = field(default_factory=list)
    validation_aurocs: list[float] = field(default_factory=list)
    specificity_aurocs: list[float] = field(default_factory=list)
    out_of_bag_sizes: list[int] = field(default_factory=list)
    nonzero_counts: list[int] = field(default_factory=list)
    chosen_c_values: list[float] = field(default_factory=list)
    gene_counter: Counter = field(default_factory=Counter)
    skipped: int = 0


def main() -> None:
    configure_logging()
    start_time = time.time()
    locked_model = load_locked_model()
    gene_order = locked_model["gene_names_order"]
    locked_genes = set(locked_model["signature_genes"])

    data = load_bootstrap_data(gene_order)
    results = run_bootstrap(data, np.array(gene_order), start_time)
    intervals = log_bootstrap_intervals(results)
    stability, locked_stability = write_gene_stability(results, locked_genes)
    write_replicate_table(results)

    replicates = len(results.out_of_bag_aurocs)
    out_of_bag, validation, specificity = intervals
    summary = {
        "estimand": (
            "uncertainty of the full selection-and-fit pipeline, including "
            "the candidate filter and the hyperparameter search, which an "
            "interval on frozen out-of-fold predictions excludes"
        ),
        "n_replicates_requested": PIPELINE_BOOTSTRAP_REPLICATES,
        "n_replicates_usable": replicates,
        "n_replicates_skipped_single_class_oob": results.skipped,
        "mean_oob_size": round(float(np.mean(results.out_of_bag_sizes)), 1),
        "discovery_oob_auroc_mean": out_of_bag[0],
        "discovery_oob_auroc_ci": [out_of_bag[1], out_of_bag[2]],
        "validation_auroc_mean": validation[0],
        "validation_auroc_ci": [validation[1], validation[2]],
        "specificity_auroc_mean": specificity[0],
        "specificity_auroc_ci": [specificity[1], specificity[2]],
        "signature_size_median": int(np.median(results.nonzero_counts)),
        "signature_size_range": [
            int(min(results.nonzero_counts)),
            int(max(results.nonzero_counts)),
        ],
```

```python
        "chosen_C_distribution": {
            str(c_value): int(count)
            for c_value, count in Counter(results.chosen_c_values).items()
        },
        "locked_gene_reselection_frequency": {
            row["gene_symbol"]: row["selection_frequency"]
            for _, row in locked_stability.iterrows()
        },
        "n_distinct_genes_ever_selected": int(len(stability)),
        "runtime_s": round(time.time() - start_time, 1),
    }
    with open(
        METRICS_DIR / "pipeline_bootstrap_summary.json", "w", encoding="utf-8"
    ) as summary_file:
        json.dump(summary, summary_file, indent=2)
    logger.info("%s", json.dumps(summary, indent=2))


def load_bootstrap_data(gene_order: list[str]) -> BootstrapData:
    columns = ["geo_accession", *gene_order]
    discovery = pd.read_csv(
        PROCESSED_DATA_DIR / "discovery_expr_gene.csv.gz",
        index_col=0,
        usecols=columns,
    ).astype(np.float32)
    discovery_labels = pd.read_csv(
        PROCESSED_DATA_DIR / "discovery_labels.csv", index_col=0
    )
    validation = pd.read_csv(
        PROCESSED_DATA_DIR / "validation_expr_gene.csv.gz",
        index_col=0,
        usecols=columns,
    ).astype(np.float32)
    validation_labels = pd.read_csv(
        PROCESSED_DATA_DIR / "validation_labels.csv", index_col=0
    )

    training = discovery_labels[
        discovery_labels["group"].isin(["sepsis", "healthy"])
    ]
    specificity = discovery_labels[
        discovery_labels["group"].isin(["sepsis", "noninfected_icu"])
    ]
    validation = validation.loc[validation_labels.index, gene_order]
    return BootstrapData(
        features=discovery.loc[training.index, gene_order].values,
        labels=(training["group"] == "sepsis").astype(int).values,
        validation_features=validation.values,
        validation_labels=(
            (validation_labels["group"] == "sepsis").astype(int).values
        ),
        specificity_features=discovery.loc[
            specificity.index, gene_order
        ].values,
        specificity_labels=(
            (specificity["group"] == "sepsis").astype(int).values
        ),
    )


def run_bootstrap(
    data: BootstrapData, gene_names: np.ndarray, start_time: float
) -> BootstrapResults:
    labels = data.labels
    case_rows = np.where(labels == 1)[0]
    control_rows = np.where(labels == 0)[0]
    random_generator = np.random.default_rng(RANDOM_SEED)
    results = BootstrapResults()

    for replicate in range(PIPELINE_BOOTSTRAP_REPLICATES):
        resample = np.concatenate(
            [
                random_generator.choice(
                    case_rows, len(case_rows), replace=True
                ),
                random_generator.choice(
```

```python
                    control_rows, len(control_rows), replace=True
                ),
            ]
        )
        out_of_bag = np.setdiff1d(np.arange(len(labels)), np.unique(resample))
        if len(out_of_bag) == 0 or len(np.unique(labels[out_of_bag])) < 2:
            results.skipped += 1
            continue
        _fit_and_record_replicate(
            data, resample, out_of_bag, replicate, gene_names, results
        )
        if (replicate + 1) % 25 == 0:
            logger.info(
                "  %d/%d replicates (%.0fs)",
                replicate + 1,
                PIPELINE_BOOTSTRAP_REPLICATES,
                time.time() - start_time,
            )
    return results


def _fit_and_record_replicate(
    data: BootstrapData,
    resample: np.ndarray,
    out_of_bag: np.ndarray,
    replicate: int,
    gene_names: np.ndarray,
    results: BootstrapResults,
) -> None:
    features, labels = data.features[resample], data.labels[resample]
    c_value = _select_c_by_inner_cv(
        features, labels, seed=RANDOM_SEED + replicate
    )
    candidates = _top_t_indices(features, labels, CANDIDATE_POOL_SIZE)
    scaler = StandardScaler().fit(features[:, candidates])
    model = _fit_l1_model(
        scaler.transform(features[:, candidates]),
        labels,
        c_value,
        RANDOM_SEED + replicate,
    )

    def auroc_on(scoring_features: np.ndarray, outcome: np.ndarray) -> float:
        probability = model.predict_proba(
            scaler.transform(scoring_features[:, candidates])
        )[:, 1]
        return roc_auc_score(outcome, probability)

    results.out_of_bag_aurocs.append(
        auroc_on(data.features[out_of_bag], data.labels[out_of_bag])
    )
    results.validation_aurocs.append(
        auroc_on(data.validation_features, data.validation_labels)
    )
    results.specificity_aurocs.append(
        auroc_on(data.specificity_features, data.specificity_labels)
    )
    nonzero = model.coef_.ravel() != 0
    results.gene_counter.update(gene_names[candidates][nonzero].tolist())
    results.nonzero_counts.append(int(nonzero.sum()))
    results.chosen_c_values.append(c_value)
    results.out_of_bag_sizes.append(len(out_of_bag))


def _select_c_by_inner_cv(
    features: np.ndarray, labels: np.ndarray, seed: int
) -> float:
    inner_folds = StratifiedKFold(
        n_splits=INNER_FOLDS, shuffle=True, random_state=seed
    )
    best_c, best_auroc = C_GRID[0], -np.inf
    for c_value in C_GRID:
        fold_aurocs = []
        for train_rows, test_rows in inner_folds.split(features, labels):
            if len(np.unique(labels[test_rows])) < 2:
                continue
```

```
            candidates = _top_t_indices(
                features[train_rows], labels[train_rows], CANDIDATE_POOL_SIZE
            )
            scaler = StandardScaler().fit(features[train_rows][:, candidates])
            model = _fit_l1_model(
                scaler.transform(features[train_rows][:, candidates]),
                labels[train_rows],
                c_value,
                seed,
            )
            probability = model.predict_proba(
                scaler.transform(features[test_rows][:, candidates])
            )[:, 1]
            fold_aurocs.append(roc_auc_score(labels[test_rows], probability))
        if fold_aurocs and np.mean(fold_aurocs) > best_auroc:
            best_auroc, best_c = float(np.mean(fold_aurocs)), c_value
    return best_c


def _top_t_indices(
    features: np.ndarray, labels: np.ndarray, count: int
) -> np.ndarray:
    t_statistics, _ = stats.ttest_ind(
        features[labels == 1], features[labels == 0], axis=0, equal_var=False
    )
    return np.argsort(-np.abs(np.nan_to_num(t_statistics, nan=0.0)))[:count]


def _fit_l1_model(
    features: np.ndarray, labels: np.ndarray, c_value: float, seed: int
) -> LogisticRegression:
    return LogisticRegression(
        penalty="l1",
        solver="liblinear",
        C=c_value,
        max_iter=2000,
        random_state=seed,
    ).fit(features, labels)


def log_bootstrap_intervals(
    results: BootstrapResults,
) -> tuple[tuple[float, float, float], ...]:
    intervals = (
        _mean_and_percentile_interval(results.out_of_bag_aurocs),
        _mean_and_percentile_interval(results.validation_aurocs),
        _mean_and_percentile_interval(results.specificity_aurocs),
    )
    labels = (
        "out-of-bag discovery AUROC:",
        "external validation AUROC: ",
        "specificity AUROC:         ",
    )
    logger.info(
        "full-pipeline bootstrap, B=%d usable replicates",
        len(results.out_of_bag_aurocs),
    )
    for label, (mean, lower, upper) in zip(labels, intervals, strict=True):
        logger.info("  %s %.4f (%.4f-%.4f)", label, mean, lower, upper)
    logger.info(
        "  signature size across replicates: median %d, range %d-%d",
        int(np.median(results.nonzero_counts)),
        min(results.nonzero_counts),
        max(results.nonzero_counts),
    )
    return intervals


def _mean_and_percentile_interval(
    values: list[float],
) -> tuple[float, float, float]:
    array = np.asarray(values)
    lower, upper = np.percentile(array, [2.5, 97.5])
    return (
        round(float(array.mean()), 4),
        round(float(lower), 4),
```

```
        round(float(upper), 4),
    )


def write_gene_stability(
    results: BootstrapResults, locked_genes: set[str]
) -> tuple[pd.DataFrame, pd.DataFrame]:
    replicates = len(results.out_of_bag_aurocs)
    stability = pd.DataFrame(
        [
            {
                "gene_symbol": gene,
                "n_selected": count,
                "selection_frequency": round(count / replicates, 3),
                "in_locked_signature": gene in locked_genes,
            }
            for gene, count in results.gene_counter.most_common()
        ]
    )
    stability.to_csv(
        TABLES_DIR / "pipeline_bootstrap_gene_stability.csv", index=False
    )
    locked_stability = stability[stability["in_locked_signature"]].sort_values(
        "selection_frequency", ascending=False
    )
    logger.info(
        "locked-signature gene re-selection frequency under full-pipeline "
        "resampling:\n%s",
        locked_stability[["gene_symbol", "selection_frequency"]].to_string(
            index=False
        ),
    )
    return stability, locked_stability


def write_replicate_table(results: BootstrapResults) -> None:
    pd.DataFrame(
        {
            "replicate": np.arange(len(results.out_of_bag_aurocs)),
            "oob_auroc": results.out_of_bag_aurocs,
            "validation_auroc": results.validation_aurocs,
            "specificity_auroc": results.specificity_aurocs,
            "n_oob": results.out_of_bag_sizes,
            "n_nonzero_genes": results.nonzero_counts,
            "chosen_C": results.chosen_c_values,
        }
    ).to_csv(TABLES_DIR / "pipeline_bootstrap_replicates.csv", index=False)


if __name__ == "__main__":
    main()
```

22 src/clinical_metrics.py

```
from __future__ import annotations

import json
import logging

import numpy as np
import pandas as pd
from sklearn.metrics import (
    average_precision_score,
    brier_score_loss,
    confusion_matrix,
    roc_curve,
)
from statsmodels.tools.sm_exceptions import PerfectSeparationError

from auroc_inference import auroc_report
from config import (
    METRICS_DIR,
    PROCESSED_DATA_DIR,
```

```python
    TABLES_DIR,
    configure_logging,
)
from scoring import load_locked_model, predict_probability

logger = logging.getLogger(__name__)

ESTIMATION_ERRORS = (
    np.linalg.LinAlgError,
    PerfectSeparationError,
    ValueError,
)

REFERENCE_PREVALENCE = 0.10


def main() -> None:
    configure_logging()
    locked_model = load_locked_model()
    validation_labels, validation_probabilities = score_validation_cohort(
        locked_model
    )
    crossfit = pd.read_csv(TABLES_DIR / "specificity_crossfit_predictions.csv")
    crossfit_labels = crossfit["y_true"].values

    evaluations = [
        (
            "validation_locked",
            validation_labels,
            validation_probabilities,
            "locked model, no refit, external cohort GSE57065, "
            "sepsis vs healthy",
        ),
        (
            "specificity_crossfit",
            crossfit_labels,
            crossfit["p_crossfit"].values,
            "cross-fitted: every sample scored by a model that never saw it; "
            "sepsis vs non-infected ICU. This is the clinically relevant row.",
        ),
        (
            "specificity_crossfit_rank",
            crossfit_labels,
            crossfit["p_crossfit_within_fold_rank"].values,
            "cross-fitted, within-fold percentile ranks; use the AUROC and "
            "PR-AUC from this row, not its threshold metrics (ranks have no "
            "natural operating point)",
        ),
        (
            "specificity_locked_in_sample",
            crossfit_labels,
            crossfit["p_locked_in_sample"].values,
            "final locked model: all 242 sepsis cases were in the training "
            "set; retained only to quantify the optimism of that estimate",
        ),
    ]
    summaries, threshold_rows = [], []
    for cohort, labels, probabilities, note in evaluations:
        summary, rows = evaluate_cohort(cohort, labels, probabilities, note)
        summaries.append(summary)
        threshold_rows += rows

    write_tables(summaries, threshold_rows)


def score_validation_cohort(
    locked_model: dict,
) -> tuple[np.ndarray, np.ndarray]:
    gene_order = locked_model["gene_names_order"]
    expression = pd.read_csv(
        PROCESSED_DATA_DIR / "validation_expr_gene.csv.gz",
        index_col=0,
        usecols=["geo_accession", *gene_order],
    ).astype(np.float32)
    cohort = pd.read_csv(
        PROCESSED_DATA_DIR / "validation_labels.csv", index_col=0
```

```
    )
    expression = expression.loc[cohort.index, gene_order]
    labels = (cohort["group"] == "sepsis").astype(int).to_numpy()
    return labels, predict_probability(expression, locked_model)


def evaluate_cohort(
    cohort: str, labels: np.ndarray, probabilities: np.ndarray, note: str
) -> tuple[dict, list[dict]]:
    labels = np.asarray(labels).astype(int)
    probabilities = np.asarray(probabilities, dtype=float)
    report = auroc_report(labels, probabilities)
    rows = []
    for rule, threshold in (
        ("fixed_0.5", 0.5),
        ("youden_in_sample", _youden_threshold(labels, probabilities)),
    ):
        row = _metrics_at_threshold(labels, probabilities, threshold)
        row.update({"cohort": cohort, "threshold_rule": rule})
        rows.append(row)
    summary = {
        "cohort": cohort,
        "n": int(len(labels)),
        "n_case": int(labels.sum()),
        "n_control": int((1 - labels).sum()),
        "auroc": round(report["auroc"], 4),
        "auroc_ci_method": report["reported_ci_method"],
        "auroc_ci_lo": _rounded(report["reported_ci_lo"]),
        "auroc_ci_hi": _rounded(report["reported_ci_hi"]),
        "pr_auc": round(
            float(average_precision_score(labels, probabilities)), 4
        ),
        "pr_auc_baseline_prevalence": round(float(np.mean(labels)), 4),
        "brier": round(float(brier_score_loss(labels, probabilities)), 4),
        **_calibration_fit(labels, probabilities),
        "note": note,
    }
    return summary, rows


def _youden_threshold(labels: np.ndarray, probabilities: np.ndarray) -> float:
    false_positive_rate, true_positive_rate, thresholds = roc_curve(
        labels, probabilities
    )
    youden = true_positive_rate - false_positive_rate
    return float(thresholds[int(np.argmax(youden))])


def _metrics_at_threshold(
    labels: np.ndarray, probabilities: np.ndarray, threshold: float
) -> dict:
    predicted = (probabilities >= threshold).astype(int)
    true_negatives, false_positives, false_negatives, true_positives = (
        confusion_matrix(labels, predicted, labels=[0, 1]).ravel()
    )
    sensitivity = (
        true_positives / (true_positives + false_negatives)
        if (true_positives + false_negatives)
        else float("nan")
    )
    specificity = (
        true_negatives / (true_negatives + false_positives)
        if (true_negatives + false_positives)
        else float("nan")
    )
    prevalence = float(np.mean(labels))
    ppv_cohort, npv_cohort = _predictive_values(
        sensitivity, specificity, prevalence
    )
    ppv_reference, npv_reference = _predictive_values(
        sensitivity, specificity, REFERENCE_PREVALENCE
    )
    precision = (
        true_positives / (true_positives + false_positives)
        if (true_positives + false_positives)
        else float("nan")
```

```
    )
    f1 = (
        2 * precision * sensitivity / (precision + sensitivity)
        if (precision + sensitivity) and not np.isnan(precision)
        else float("nan")
    )
    return {
        "threshold": round(float(threshold), 4),
        "tp": int(true_positives),
        "fp": int(false_positives),
        "tn": int(true_negatives),
        "fn": int(false_negatives),
        "sensitivity": round(float(sensitivity), 4),
        "specificity": round(float(specificity), 4),
        "accuracy": round(
            float((true_positives + true_negatives) / len(labels)), 4
        ),
        "balanced_accuracy": round(float((sensitivity + specificity) / 2), 4),
        "f1": round(float(f1), 4),
        "cohort_prevalence": round(prevalence, 4),
        "ppv_at_cohort_prevalence": round(ppv_cohort, 4),
        "npv_at_cohort_prevalence": round(npv_cohort, 4),
        f"ppv_at_prevalence_{REFERENCE_PREVALENCE}": round(ppv_reference, 4),
        f"npv_at_prevalence_{REFERENCE_PREVALENCE}": round(npv_reference, 4),
    }


def _predictive_values(
    sensitivity: float, specificity: float, prevalence: float
) -> tuple[float, float]:
    ppv_numerator = sensitivity * prevalence
    ppv_denominator = ppv_numerator + (1 - specificity) * (1 - prevalence)
    npv_numerator = specificity * (1 - prevalence)
    npv_denominator = npv_numerator + (1 - sensitivity) * prevalence
    ppv = (
        float(ppv_numerator / ppv_denominator)
        if ppv_denominator > 0
        else float("nan")
    )
    npv = (
        float(npv_numerator / npv_denominator)
        if npv_denominator > 0
        else float("nan")
    )
    return ppv, npv


def _rounded(value: float | None) -> float | None:
    return None if value is None else round(value, 4)


def _calibration_fit(
    labels: np.ndarray, probabilities: np.ndarray, epsilon: float = 1e-6
) -> dict:
    import statsmodels.api as sm

    clipped = np.clip(probabilities, epsilon, 1 - epsilon)
    logit = np.log(clipped / (1 - clipped))
    try:
        slope_model = sm.Logit(labels, sm.add_constant(logit)).fit(disp=0)
        slope = float(slope_model.params[1])
        intercept_model = sm.Logit(
            labels, np.ones_like(logit), offset=logit
        ).fit(disp=0)
        intercept = float(intercept_model.params[0])
    except ESTIMATION_ERRORS as error:
        return {
            "calibration_intercept": None,
            "calibration_slope": None,
            "calibration_note": f"not estimable ({type(error).__name__}); "
            "logistic recalibration diverges under complete separation",
        }
    return {
        "calibration_intercept": round(intercept, 4),
        "calibration_slope": round(slope, 4),
        "calibration_note": None,
```

```
    }


def write_tables(summaries: list[dict], threshold_rows: list[dict]) -> None:
    summary_table = pd.DataFrame(summaries)
    threshold_table = pd.DataFrame(threshold_rows)
    summary_table.to_csv(
        TABLES_DIR / "clinical_metrics_summary.csv", index=False
    )
    threshold_table.to_csv(
        TABLES_DIR / "clinical_metrics_by_threshold.csv", index=False
    )
    logger.info("\n%s", summary_table.to_string(index=False))
    with open(
        METRICS_DIR / "clinical_metrics_summary.json", "w", encoding="utf-8"
    ) as summary_file:
        json.dump(
            {
                "threshold_free": summaries,
                "by_threshold": threshold_rows,
                "reference_prevalence": REFERENCE_PREVALENCE,
                "youden_caveat": "the Youden threshold is chosen on the "
                "evaluation data and its operating characteristics are "
                "therefore optimistic",
            },
            summary_file,
            indent=2,
        )


if __name__ == "__main__":
    main()
```

23 src/demographic_sensitivity.py

```
from __future__ import annotations

import json
import logging

import numpy as np
import pandas as pd
import statsmodels.api as sm
from scipy import stats
from sklearn.linear_model import LogisticRegression
from sklearn.metrics import roc_auc_score
from statsmodels.tools.sm_exceptions import PerfectSeparationError

from auroc_inference import auroc_report
from config import (
    INTERIM_DATA_DIR,
    METRICS_DIR,
    PROCESSED_DATA_DIR,
    TABLES_DIR,
    configure_logging,
)
from scoring import load_locked_model, predict_probability

logger = logging.getLogger(__name__)

ESTIMATION_ERRORS = (
    np.linalg.LinAlgError,
    PerfectSeparationError,
    ValueError,
)

AGE_CALIPER = 5.0
REQUIRED_COLUMNS = ["y", "score", "age", "female"]


def main() -> None:
    configure_logging()
    contrasts = {
```

```python
        "sepsis_vs_noninfected_icu_crossfit": load_specificity_contrast(),
        "sepsis_vs_healthy_validation_locked": load_validation_contrast(),
    }

    incremental, stratified_rows, matched_rows, balance_rows = [], [], [], []
    for name, contrast in contrasts.items():
        incremental.append(incremental_discrimination(contrast, name))
        stratified_rows += stratified_auroc(contrast, name)
        matched_rows.append(matched_auroc(contrast, name))
        balance_rows += demographic_balance(contrast, name)

    write_outputs(incremental, stratified_rows, matched_rows, balance_rows)


def load_specificity_contrast() -> pd.DataFrame:
    metadata = pd.read_csv(
        INTERIM_DATA_DIR / "discovery_sample_metadata.csv"
    ).set_index("geo_accession")
    crossfit = pd.read_csv(
        TABLES_DIR / "specificity_crossfit_predictions.csv"
    ).set_index("geo_accession")
    contrast = pd.DataFrame(
        {
            "y": crossfit["y_true"],
            "score": crossfit["p_crossfit_within_fold_rank"],
            "age": pd.to_numeric(
                metadata.loc[crossfit.index, "age"], errors="coerce"
            ),
            "female": _female_indicator(
                metadata.loc[crossfit.index, "gender"]
            ),
        }
    )
    logger.info(
        "specificity contrast: n=%d, missing age=%d, missing sex=%d",
        len(contrast),
        int(contrast["age"].isna().sum()),
        int(contrast["female"].isna().sum()),
    )
    return contrast


def _female_indicator(sex: pd.Series) -> pd.Series:
    return sex.astype(str).str.lower().str.startswith("f").astype(float)


def load_validation_contrast() -> pd.DataFrame:
    locked_model = load_locked_model()
    gene_order = locked_model["gene_names_order"]
    metadata = pd.read_csv(INTERIM_DATA_DIR / "validation_sample_metadata.csv")
    age_sex = pd.read_csv(INTERIM_DATA_DIR / "validation_age_gender.csv")
    metadata = metadata.merge(
        age_sex[["geo_accession", "age", "gender"]],
        on="geo_accession",
        how="left",
    ).set_index("geo_accession")
    cohort = pd.read_csv(
        PROCESSED_DATA_DIR / "validation_labels.csv", index_col=0
    )
    expression = pd.read_csv(
        PROCESSED_DATA_DIR / "validation_expr_gene.csv.gz",
        index_col=0,
        usecols=["geo_accession", *gene_order],
    ).astype(np.float32)
    expression = expression.loc[cohort.index, gene_order]
    contrast = pd.DataFrame(
        {
            "y": (cohort["group"] == "sepsis").astype(int),
            "score": predict_probability(expression, locked_model),
            "age": pd.to_numeric(
                metadata.loc[cohort.index, "age"], errors="coerce"
            ),
            "female": _female_indicator(metadata.loc[cohort.index, "gender"]),
        }
    )
    logger.info(
```

```
        "validation contrast: n=%d, missing age=%d",
        len(contrast),
        int(contrast["age"].isna().sum()),
    )
    return contrast


def incremental_discrimination(contrast: pd.DataFrame, name: str) -> dict:
    complete = contrast.dropna(subset=REQUIRED_COLUMNS).copy()
    labels = complete["y"].values.astype(int)
    result: dict = {
        "contrast": name,
        "n_analyzed": int(len(complete)),
        "n_dropped_missing_demographics": int(len(contrast) - len(complete)),
    }
    if len(np.unique(labels)) < 2:
        result["error"] = "only one class after dropping missing demographics"
        return result

    designs = {
        "demography_only": ["age", "female"],
        "score_only": ["score"],
        "score_plus_demography": ["score", "age", "female"],
    }
    fits, aurocs = {}, {}
    for model_name, columns in designs.items():
        design = sm.add_constant(complete[columns].astype(float))
        try:
            model = sm.Logit(labels, design).fit(disp=0, maxiter=200)
            fits[model_name] = model
            aurocs[model_name] = float(
                roc_auc_score(labels, model.predict(design))
            )
        except ESTIMATION_ERRORS as error:
            fits[model_name] = None
            result[f"{model_name}_note"] = (
                f"unpenalized MLE not estimable ({type(error).__name__}); "
                "complete or quasi-complete separation. AUROC computed "
                "without it."
            )
            aurocs[model_name] = _auroc_without_mle(
                complete, labels, model_name, columns
            )
    result["auroc"] = {
        key: (None if value is None else round(value, 4))
        for key, value in aurocs.items()
    }
    result.update(_likelihood_ratio_test(fits))
    return result


def _auroc_without_mle(
    complete: pd.DataFrame,
    labels: np.ndarray,
    model_name: str,
    columns: list[str],
) -> float:
    if model_name == "score_only":
        return float(roc_auc_score(labels, complete["score"].values))
    penalized = LogisticRegression(
        penalty="l2", C=1e6, solver="lbfgs", max_iter=10000
    ).fit(complete[columns].values, labels)
    return float(
        roc_auc_score(
            labels, penalized.predict_proba(complete[columns].values)[:, 1]
        )
    )


def _likelihood_ratio_test(fits: dict) -> dict:
    reduced = fits["demography_only"]
    full = fits["score_plus_demography"]
    if reduced is None or full is None:
        return {
            "lr_test_score_given_demography": {
                "p_value": None,
```

```python
                "note": "not estimable under complete separation of the "
                "two classes",
            }
        }
    statistic = 2 * (full.llf - reduced.llf)
    p_value = float(stats.chi2.sf(statistic, df=1))
    beta = float(full.params["score"])
    standard_error = float(full.bse["score"])
    return {
        "lr_test_score_given_demography": {
            "lr_statistic": round(float(statistic), 3),
            "df": 1,
            "p_value": float(f"{p_value:.3g}"),
        },
        "adjusted_score_coefficient": {
            "beta": round(beta, 4),
            "se": round(standard_error, 4),
            "or_per_unit": round(float(np.exp(beta)), 4),
            "or_ci_lo": round(float(np.exp(beta - 1.96 * standard_error)), 4),
            "or_ci_hi": round(float(np.exp(beta + 1.96 * standard_error)), 4),
        },
    }


def stratified_auroc(contrast: pd.DataFrame, name: str) -> list[dict]:
    complete = contrast.dropna(subset=REQUIRED_COLUMNS).copy()
    rows = []
    for sex, mask in (
        ("female", complete["female"] == 1),
        ("male", complete["female"] == 0),
    ):
        stratum = complete[mask]
        if len(stratum) and stratum["y"].nunique() == 2:
            rows.append(
                {
                    "contrast": name,
                    "stratum": f"sex={sex}",
                    "n": int(len(stratum)),
                    "n_case": int(stratum["y"].sum()),
                    "auroc": _stratum_auroc(stratum),
                }
            )
    try:
        complete["age_tertile"] = pd.qcut(
            complete["age"], 3, labels=["T1_young", "T2_mid", "T3_old"]
        )
    except ValueError:
        return rows
    for tertile, stratum in complete.groupby("age_tertile", observed=True):
        if len(stratum) and stratum["y"].nunique() == 2:
            rows.append(
                {
                    "contrast": name,
                    "stratum": f"age_{tertile}",
                    "n": int(len(stratum)),
                    "n_case": int(stratum["y"].sum()),
                    "age_range": f"{stratum['age'].min():.0f}-"
                    f"{stratum['age'].max():.0f}",
                    "auroc": _stratum_auroc(stratum),
                }
            )
    return rows


def _stratum_auroc(stratum: pd.DataFrame) -> float:
    return round(float(roc_auc_score(stratum["y"], stratum["score"])), 4)


def matched_auroc(
    contrast: pd.DataFrame, name: str, caliper: float = AGE_CALIPER
) -> dict:
    complete = contrast.dropna(subset=REQUIRED_COLUMNS).copy()
    pairs = _greedy_pairs(complete, caliper)
    if len(pairs) < 5:
        return {
            "contrast": name,
```

```
            "n_pairs": len(pairs),
            "error": "too few matched pairs for a stable estimate",
            "caliper_years": caliper,
        }
    rows = [case for case, _ in pairs] + [control for _, control in pairs]
    matched = complete.loc[rows]
    report = auroc_report(
        matched["y"].values.astype(int), matched["score"].values
    )
    case_ages = matched[matched["y"] == 1]["age"]
    control_ages = matched[matched["y"] == 0]["age"]
    t_statistic, p_value = stats.ttest_ind(
        case_ages, control_ages, equal_var=False
    )
    return {
        "contrast": name,
        "n_pairs": len(pairs),
        "caliper_years": caliper,
        "matched_auroc": round(report["auroc"], 4),
        "matched_ci_method": report["reported_ci_method"],
        "matched_ci_lo": _rounded(report["reported_ci_lo"]),
        "matched_ci_hi": _rounded(report["reported_ci_hi"]),
        "matched_age_case_mean": round(float(case_ages.mean()), 1),
        "matched_age_control_mean": round(float(control_ages.mean()), 1),
        "matched_age_welch_t": round(float(t_statistic), 3),
        "matched_age_p": float(f"{p_value:.3g}"),
        "matched_pct_female": round(float(100 * matched["female"].mean()), 1),
    }


def _greedy_pairs(
    complete: pd.DataFrame, caliper: float
) -> list[tuple[object, object]]:
    cases = complete[complete["y"] == 1].sort_values("age").copy()
    controls = complete[complete["y"] == 0].sort_values("age").copy()
    used: set = set()
    pairs = []
    for control_id, control in controls.iterrows():
        candidates = cases[
            (cases["female"] == control["female"])
            & (~cases.index.isin(used))
            & ((cases["age"] - control["age"]).abs() <= caliper)
        ]
        if len(candidates) == 0:
            continue
        case_id = (candidates["age"] - control["age"]).abs().idxmin()
        used.add(case_id)
        pairs.append((case_id, control_id))
    return pairs


def _rounded(value: float | None) -> float | None:
    return None if value is None else round(value, 4)


def demographic_balance(contrast: pd.DataFrame, name: str) -> list[dict]:
    complete = contrast.dropna(subset=["age", "female"])
    rows = []
    for label, group in ((1, "case"), (0, "control")):
        members = complete[complete["y"] == label]
        rows.append(
            {
                "contrast": name,
                "group": group,
                "n": int(len(members)),
                "age_mean": round(float(members["age"].mean()), 1),
                "age_sd": round(float(members["age"].std()), 1),
                "pct_female": round(float(100 * members["female"].mean()), 1),
            }
        )
    cases = complete[complete["y"] == 1]
    controls = complete[complete["y"] == 0]
    t_statistic, age_p = stats.ttest_ind(
        cases["age"], controls["age"], equal_var=False
    )
    chi_square, sex_p = stats.chi2_contingency(
```

```python
        pd.crosstab(complete["y"], complete["female"])
    )[:2]
    rows.append(
        {
            "contrast": name,
            "group": "TEST",
            "n": int(len(complete)),
            "age_welch_t": round(float(t_statistic), 3),
            "age_p": float(f"{age_p:.3g}"),
            "sex_chi2": round(float(chi_square), 3),
            "sex_p": float(f"{sex_p:.3g}"),
        }
    )
    return rows


def write_outputs(
    incremental: list[dict],
    stratified_rows: list[dict],
    matched_rows: list[dict],
    balance_rows: list[dict],
) -> None:
    balance_table = pd.DataFrame(balance_rows)
    balance_table.to_csv(TABLES_DIR / "demographic_balance.csv", index=False)
    pd.DataFrame(stratified_rows).to_csv(
        TABLES_DIR / "demographic_stratified_auroc.csv", index=False
    )
    logger.info("\n%s", balance_table.to_string(index=False))
    logger.info("%s", json.dumps(matched_rows, indent=2))
    with open(
        METRICS_DIR / "demographic_sensitivity_summary.json",
        "w",
        encoding="utf-8",
    ) as summary_file:
        json.dump(
            {
                "incremental_discrimination": incremental,
                "stratified_auroc": stratified_rows,
                "matched_analysis": matched_rows,
                "balance": balance_rows,
                "age_caliper_years": AGE_CALIPER,
            },
            summary_file,
            indent=2,
        )


if __name__ == "__main__":
    main()
```

24 src/enrichment_analysis.py

```python
from __future__ import annotations

import json
import logging
from pathlib import Path

import pandas as pd
from scipy.stats import hypergeom
from statsmodels.stats.multitest import multipletests

from config import (
    GO_BIOLOGICAL_PROCESS_GENE_SETS,
    HALLMARK_GENE_SETS,
    KEGG_GENE_SETS,
    METRICS_DIR,
    TABLES_DIR,
    configure_logging,
)
from scoring import load_locked_model

logger = logging.getLogger(__name__)
```

```python

DISPLAY_MIN_OVERLAP = 3
FDR_THRESHOLD = 0.05
LIBRARY_FILES = {
    "Hallmark": HALLMARK_GENE_SETS,
    "KEGG": KEGG_GENE_SETS,
    "GO_BP": GO_BIOLOGICAL_PROCESS_GENE_SETS,
}
SIGNIFICANT_TERM_FILES = {
    "DE_genes_fdr05_fc1": "enrichment_significant_DE_genes.csv",
    "locked_signature_10gene": "enrichment_significant_signature.csv",
}


def main() -> None:
    configure_logging()
    universe, queries = load_queries()
    libraries = {
        name: load_gene_sets(path) for name, path in LIBRARY_FILES.items()
    }
    for name, library in libraries.items():
        logger.info("%s: %s terms", name, len(library))

    frames, family_counts = [], {}
    for query_name, query in queries.items():
        results, untestable = test_query(query, universe, libraries)
        results.insert(0, "gene_list", query_name)
        results["fdr_family"] = (
            "all testable terms across Hallmark + KEGG + GO_BP, per query set"
        )
        frames.append(results)
        family_counts[query_name] = count_family(
            results, untestable, query, universe
        )
        logger.info("%s: %s", query_name, family_counts[query_name])

    all_results = pd.concat(frames, ignore_index=True).sort_values(
        ["gene_list", "p_raw"]
    )
    all_results.to_csv(TABLES_DIR / "enrichment_all_results.csv", index=False)
    write_significant_terms(all_results)
    write_summary(family_counts)


def load_queries() -> tuple[set[str], dict[str, set[str]]]:
    differential = pd.read_csv(
        TABLES_DIR / "differential_expression_discovery.csv"
    )
    universe = set(differential["gene_symbol"].str.upper())
    significant = differential[
        (differential["p_adj"] < FDR_THRESHOLD)
        & (differential["log2fc"].abs() >= 1.0)
    ]
    queries = {
        "DE_genes_fdr05_fc1": set(significant["gene_symbol"].str.upper()),
        "locked_signature_10gene": {
            gene.upper() for gene in load_locked_model()["signature_genes"]
        },
    }
    logger.info(
        "universe %s; DE query %s; signature query %s",
        len(universe),
        len(queries["DE_genes_fdr05_fc1"]),
        len(queries["locked_signature_10gene"]),
    )
    return universe, queries


def load_gene_sets(path: Path) -> dict[str, set[str]]:
    gene_sets = {}
    with open(path, encoding="utf-8", errors="replace") as gmt_file:
        for line in gmt_file:
            fields = line.rstrip("\n").split("\t")
            genes = {
                gene.strip().upper() for gene in fields[2:] if gene.strip()
            }
```

```python
            if genes:
                gene_sets[fields[0]] = genes
    return gene_sets


def test_query(
    query: set[str],
    universe: set[str],
    libraries: dict[str, dict[str, set[str]]],
) -> tuple[pd.DataFrame, int]:
    per_library, untestable = [], 0
    for library_name, library in libraries.items():
        results, library_untestable = test_all_terms(
            query, universe, library, library_name
        )
        untestable += library_untestable
        results["p_adj_within_library"] = multipletests(
            results["p_raw"], method="fdr_bh"
        )[1]
        per_library.append(results)
    combined = pd.concat(per_library, ignore_index=True)
    combined["p_adj"] = multipletests(combined["p_raw"], method="fdr_bh")[1]
    return combined, untestable


def test_all_terms(
    query: set[str],
    universe: set[str],
    library: dict[str, set[str]],
    library_name: str,
) -> tuple[pd.DataFrame, int]:
    universe_size = len(universe)
    query_size = len(query & universe)
    rows, untestable = [], 0
    for term, genes in library.items():
        term_genes = genes & universe
        if not term_genes:
            untestable += 1
            continue
        overlap = query & term_genes
        rows.append(
            {
                "library": library_name,
                "term": term,
                "k_overlap": len(overlap),
                "K_term_size": len(term_genes),
                "n_query": query_size,
                "N_universe": universe_size,
                "genes": ";".join(sorted(overlap)),
                "p_raw": hypergeom.sf(
                    len(overlap) - 1,
                    universe_size,
                    len(term_genes),
                    query_size,
                ),
            }
        )
    return pd.DataFrame(rows), untestable


def count_family(
    results: pd.DataFrame,
    untestable: int,
    query: set[str],
    universe: set[str],
) -> dict[str, int]:
    family_wide = results["p_adj"] < FDR_THRESHOLD
    return {
        "n_terms_tested": int(len(results)),
        "n_terms_untestable_no_gene_in_universe": int(untestable),
        "n_query_genes_in_universe": int(len(query & universe)),
        "n_sig_fdr05_family_wide": int(family_wide.sum()),
        "n_sig_fdr05_within_library": int(
            (results["p_adj_within_library"] < FDR_THRESHOLD).sum()
        ),
        "n_sig_fdr05_family_wide_and_overlap_ge_3": int(
```

```python
            (family_wide & (results["k_overlap"] >= DISPLAY_MIN_OVERLAP)).sum()
        ),
    }


def write_significant_terms(all_results: pd.DataFrame) -> None:
    for query_name, file_name in SIGNIFICANT_TERM_FILES.items():
        significant = all_results[
            (all_results["gene_list"] == query_name)
            & (all_results["p_adj"] < FDR_THRESHOLD)
            & (all_results["k_overlap"] >= DISPLAY_MIN_OVERLAP)
        ].sort_values(["library", "p_adj"])
        significant.to_csv(TABLES_DIR / file_name, index=False)
        logger.info(
            "%s: %s terms at FDR<0.05 and overlap>=%s",
            query_name,
            len(significant),
            DISPLAY_MIN_OVERLAP,
        )


def write_summary(family_counts: dict[str, dict[str, int]]) -> None:
    summary = {
        "test": (
            "hypergeometric over-representation analysis (ORA), "
            "one-sided P(X >= k)"
        ),
        "not_gsea": (
            "This is not gene set enrichment analysis in the ranked-list "
            "sense."
        ),
        "directionality": (
            "ORA is non-directional: over-representation does not indicate "
            "up- or down-regulation, and no activation claim is made"
        ),
        "universe": (
            "the 9983-gene transportable candidate pool that entered "
            "differential expression"
        ),
        "fdr_primary_family": (
            "all terms with at least one gene in the universe, pooled "
            "across Hallmark, KEGG and GO Biological Process, corrected "
            "once per query set"
        ),
        "fdr_secondary_family": (
            "the same terms corrected within each library separately"
        ),
        "display_filter": (
            f"overlap >= {DISPLAY_MIN_OVERLAP} applied AFTER correction, for "
            "display only; it affects no p-value"
        ),
        "counts": family_counts,
    }
    with open(
        METRICS_DIR / "enrichment_summary.json", "w", encoding="utf-8"
    ) as summary_file:
        json.dump(summary, summary_file, indent=2)


if __name__ == "__main__":
    main()
```

25 src/table1_cohorts.py

```python
from __future__ import annotations

import json
import logging

import numpy as np
import pandas as pd
from scipy import stats

```

```python
import formatting
from config import INTERIM_DATA_DIR, METRICS_DIR, TABLES_DIR, configure_logging

logger = logging.getLogger(__name__)

DISCOVERY_COHORT = "Discovery (GSE65682)"
VALIDATION_COHORT = "Validation (GSE57065)"
GROUP_COMPARISONS = (
    ("discovery_sepsis_vs_healthy", "discovery", "sepsis", "healthy"),
    (
        "discovery_sepsis_vs_noninfected_icu",
        "discovery",
        "sepsis",
        "noninfected_icu",
    ),
    ("validation_sepsis_vs_healthy", "validation", "sepsis", "healthy"),
)


def main() -> None:
    configure_logging()
    discovery = pd.read_csv(INTERIM_DATA_DIR / "discovery_sample_metadata.csv")
    validation = load_validation_metadata()

    table1 = build_table1(discovery, validation)
    comparisons = compare_groups(
        {"discovery": discovery, "validation": validation}
    )

    table1.to_csv(
        TABLES_DIR / "table1_cohort_characteristics.csv", index=False
    )
    logger.info("\n%s", table1.to_string(index=False))
    with open(
        METRICS_DIR / "table1_age_test.json", "w", encoding="utf-8"
    ) as output:
        json.dump(comparisons, output, indent=2)


def load_validation_metadata() -> pd.DataFrame:
    validation = pd.read_csv(
        INTERIM_DATA_DIR / "validation_sample_metadata.csv"
    )
    age_gender = pd.read_csv(INTERIM_DATA_DIR / "validation_age_gender.csv")
    return validation.merge(
        age_gender[["geo_accession", "age", "gender"]],
        on="geo_accession",
        how="left",
    )


def build_table1(
    discovery: pd.DataFrame, validation: pd.DataFrame
) -> pd.DataFrame:
    rows = [
        {
            "cohort": DISCOVERY_COHORT,
            "group": group,
            **_summarize_group(discovery, group),
        }
        for group in ("sepsis", "noninfected_icu", "healthy")
    ]
    rows += [
        {
            "cohort": VALIDATION_COHORT,
            "group": group,
            **_summarize_group(validation, group),
        }
        for group in ("sepsis", "healthy")
    ]
    return pd.DataFrame(rows)


def _summarize_group(metadata: pd.DataFrame, group: str) -> dict:
    members = metadata[metadata["group"] == group]
    if "age" in members.columns:
```

```python
        age = pd.to_numeric(members["age"], errors="coerce")
    else:
        age = pd.Series(dtype=float)
    if "gender" in members.columns:
        percent_female = 100 * (_is_female(members)).mean()
    else:
        percent_female = np.nan
    return {
        "n": len(members),
        "age_mean": age.mean() if age.notna().any() else np.nan,
        "age_sd": age.std() if age.notna().any() else np.nan,
        "pct_female": percent_female,
    }


def compare_groups(cohorts: dict[str, pd.DataFrame]) -> dict[str, dict]:
    comparisons = {}
    for label, cohort, first_group, second_group in GROUP_COMPARISONS:
        metadata = cohorts[cohort]
        first_age = _ages(metadata, first_group)
        second_age = _ages(metadata, second_group)
        t_statistic, age_p_value = stats.ttest_ind(
            first_age, second_age, equal_var=False
        )
        first_female = _is_female(metadata[metadata["group"] == first_group])
        second_female = _is_female(metadata[metadata["group"] == second_group])
        counts = np.array(
            [
                [int(first_female.sum()), int((~first_female).sum())],
                [int(second_female.sum()), int((~second_female).sum())],
            ]
        )
        chi_square, sex_p_value = stats.chi2_contingency(counts)[:2]
        comparisons[label] = {
            "age_welch_t": round(float(t_statistic), 3),
            "age_p_value": float(f"{age_p_value:.3g}"),
            "age_mean_diff": round(
                float(first_age.mean() - second_age.mean()), 2
            ),
            "sex_chi2": round(float(chi_square), 3),
            "sex_p_value": float(f"{sex_p_value:.3g}"),
            "pct_female_group1": round(float(100 * first_female.mean()), 1),
            "pct_female_group2": round(float(100 * second_female.mean()), 1),
        }
        logger.info(
            "%s: age Welch t=%.3f, %s; sex chi2=%.3f, %s",
            label,
            t_statistic,
            formatting.format_p_value(age_p_value),
            chi_square,
            formatting.format_p_value(sex_p_value),
        )
    return comparisons


def _ages(metadata: pd.DataFrame, group: str) -> pd.Series:
    return pd.to_numeric(
        metadata.loc[metadata["group"] == group, "age"], errors="coerce"
    ).dropna()


def _is_female(members: pd.DataFrame) -> pd.Series:
    return members["gender"].astype(str).str.lower().str.startswith("f")


if __name__ == "__main__":
    main()
```

26 src/make_summary_figures.py

```
from __future__ import annotations

import logging

import matplotlib.pyplot as plt
import numpy as np
import pandas as pd
from matplotlib.patches import Patch

import plotting
from config import FIGURES_DIR, TABLES_DIR, configure_logging

logger = logging.getLogger(__name__)

TOP_TERMS = 10
TERM_LABEL_LENGTH = 45


def main() -> None:
    configure_logging()
    plot_enrichment_terms()
    plot_signature_coefficients()
    logger.info(
        "wrote fig7_enrichment_top_terms and fig8_signature_coefficients"
    )


def plot_enrichment_terms() -> None:
    results = pd.read_csv(TABLES_DIR / "enrichment_all_results.csv")
    candidates = results[
        (results["gene_list"] == "DE_genes_fdr05_fc1")
        & (results["library"].isin(["Hallmark", "KEGG"]))
    ]
    top_terms = candidates.sort_values("p_adj").head(TOP_TERMS).iloc[::-1]

    plotting.apply_style()
    fig, ax = plt.subplots(figsize=(plotting.FULL_WIDTH_INCHES, 4.8))
    library_colors = {
        "Hallmark": plotting.PALETTE["blue"],
        "KEGG": plotting.PALETTE["orange"],
    }
    ax.barh(
        range(len(top_terms)),
        -np.log10(top_terms["p_adj"].clip(lower=1e-300)),
        color=[library_colors[library] for library in top_terms["library"]],
    )
    ax.set_yticks(range(len(top_terms)))
    ax.set_yticklabels(
        [term[:TERM_LABEL_LENGTH] for term in top_terms["term"]], fontsize=8
    )
    ax.set_xlabel(r"$-\log_{10}$(FDR-adjusted $p$)")
    ax.legend(
        handles=[
            Patch(facecolor=color, label=library)
            for library, color in library_colors.items()
        ],
        loc="lower right",
    )
    plotting.save_figure(fig, FIGURES_DIR, "fig7_enrichment_top_terms")
    plt.close(fig)


def plot_signature_coefficients() -> None:
    signature = pd.read_csv(TABLES_DIR / "locked_signature.csv").sort_values(
        "coef"
    )
    plotting.apply_style()
    fig, ax = plt.subplots(figsize=(plotting.FULL_WIDTH_INCHES, 4.2))
    ax.barh(
        signature["gene_symbol"],
        signature["coef"],
        color=[
            (
                plotting.PALETTE["red"]
```

```
                if coefficient > 0
                else plotting.PALETTE["blue"]
            )
            for coefficient in signature["coef"]
        ],
    )
    ax.axvline(0, color=plotting.PALETTE["gray"], linewidth=0.8)
    ax.set_xlabel(r"Standardized logistic-regression coefficient ($\beta$)")
    ax.legend(
        handles=[
            Patch(facecolor=plotting.PALETTE["red"], label="Higher in sepsis"),
            Patch(
                facecolor=plotting.PALETTE["blue"], label="Higher in healthy"
            ),
        ],
        loc="lower right",
    )
    plotting.save_figure(fig, FIGURES_DIR, "fig8_signature_coefficients")
    plt.close(fig)


if __name__ == "__main__":
    main()
```

27 src/make_figures_3_4.py

```
from __future__ import annotations

import json
import logging
from pathlib import Path

import matplotlib.pyplot as plt
import pandas as pd
from sklearn.metrics import roc_curve

import formatting
import plotting
from config import (
    FIGURES_DIR,
    METRICS_DIR,
    PROCESSED_DATA_DIR,
    TABLES_DIR,
    configure_logging,
)

logger = logging.getLogger(__name__)

TASK_LABELS = {
    "validation": "External validation AUROC (sepsis vs. healthy)",
    "specificity": "Sepsis vs. non-infected ICU AUROC",
}
SPECIFICITY_KEY = "sepsis_vs_noninfected_icu_crossfit"


def main() -> None:
    configure_logging()
    plot_random_panel_benchmark()
    plot_specificity()


def plot_random_panel_benchmark() -> None:
    benchmark = pd.read_csv(TABLES_DIR / "random_panel_benchmark.csv")
    signature_size = _read_json(
        METRICS_DIR / "random_panel_benchmark_summary.json"
    )["signature_size"]

    plotting.apply_style()
    fig, axes = plt.subplots(
        1, 3, figsize=(plotting.FULL_WIDTH_INCHES * 3, 4.2)
    )
    for ax, task, letter in zip(
        axes[:2], ["validation", "specificity"], "AB", strict=False
```

```
    ):
        _plot_null_distribution(ax, benchmark, task, signature_size)
        plotting.label_panel(ax, letter)
    _plot_panel_size_sweep(axes[2], benchmark)
    plotting.label_panel(axes[2], "C")

    plotting.save_figure(fig, FIGURES_DIR, "fig3_random_panel_benchmark")
    plt.close(fig)
    logger.info("wrote fig3_random_panel_benchmark")


def _read_json(path: Path) -> dict:
    with open(path, encoding="utf-8") as json_file:
        return json.load(json_file)


def _plot_null_distribution(
    ax, benchmark: pd.DataFrame, task: str, signature_size: int
) -> None:
    null_aurocs = pd.read_csv(
        PROCESSED_DATA_DIR / f"random_panel_null_{task}.csv"
    )["null_auroc"].values
    primary = benchmark[
        (benchmark["pool"] == "transportable")
        & (benchmark["panel_size"] == signature_size)
        & (benchmark["recipe"] == "l1_modalC")
        & (benchmark["task"] == task)
    ].iloc[0]
    draws = formatting.format_count(int(primary["n_draws"]))
    selected = formatting.format_number(primary["real_auroc_refit"], "auroc")
    ax.hist(
        null_aurocs,
        bins=40,
        color=plotting.PALETTE["sky"],
        edgecolor="white",
        label=f"Random {signature_size}-gene panels (n = {draws})",
    )
    ax.axvline(
        primary["real_auroc_refit"],
        color=plotting.PALETTE["red"],
        linestyle="--",
        label=f"Selected panel (AUROC {selected})",
    )
    ax.set_xlabel(TASK_LABELS[task])
    ax.set_ylabel("Count")
    ax.set_ylim(0, ax.get_ylim()[1] * 1.35)
    ax.legend(loc="upper left", bbox_to_anchor=(0.0, 0.93), fontsize="small")


def _plot_panel_size_sweep(ax, benchmark: pd.DataFrame) -> None:
    sweep = benchmark[
        (benchmark["pool"] == "transportable")
        & (benchmark["recipe"] == "l1_modalC")
    ]
    for task, color, marker in (
        ("validation", plotting.PALETTE["orange"], "o"),
        ("specificity", plotting.PALETTE["pink"], "s"),
    ):
        task_rows = sweep[sweep["task"] == task].sort_values("panel_size")
        proportion = task_rows["prop_random_ge_real"]
        ax.errorbar(
            task_rows["panel_size"],
            proportion,
            yerr=[
                proportion - task_rows["prop_ci_lo"],
                task_rows["prop_ci_hi"] - proportion,
            ],
            color=color,
            marker=marker,
            linestyle="-",
            capsize=2.5,
            label=task.capitalize(),
        )
    ax.axhline(
        0.05, color=plotting.PALETTE["gray"], linestyle=":", label="α = 0.05"
    )
```

```python
    panel_sizes = sorted(sweep["panel_size"].unique())
    ax.set_xscale("log")
    ax.set_xticks(panel_sizes)
    ax.set_xticklabels([str(size) for size in panel_sizes])
    ax.set_xlabel("Panel size $k$")
    ax.set_ylabel(
        "Fraction of random panels matching or\nexceeding the selected panel"
    )
    ax.legend(loc="upper left", bbox_to_anchor=(0.0, 0.93))


def plot_specificity() -> None:
    predictions = pd.read_csv(
        TABLES_DIR / "specificity_crossfit_predictions.csv"
    )
    folds = pd.read_csv(TABLES_DIR / "specificity_crossfit_folds.csv")
    crossfit = _read_json(METRICS_DIR / "specificity_crossfit_summary.json")
    demographics = _read_json(
        METRICS_DIR / "demographic_sensitivity_summary.json"
    )

    plotting.apply_style()
    fig, axes = plt.subplots(
        1, 2, figsize=(plotting.FULL_WIDTH_INCHES * 2, 4.2)
    )
    fig.subplots_adjust(wspace=0.42)
    _plot_roc_curves(axes[0], predictions, crossfit)
    plotting.label_panel(axes[0], "A")
    _plot_estimate_comparison(axes[1], folds, crossfit, demographics)
    plotting.label_panel(axes[1], "B")

    plotting.save_figure(fig, FIGURES_DIR, "fig4_specificity_crossfit")
    plt.close(fig)
    logger.info("wrote fig4_specificity_crossfit")


def _plot_roc_curves(
    ax, predictions: pd.DataFrame, crossfit: dict[str, float]
) -> None:
    labels = predictions["y_true"].values
    crossfit_auroc = formatting.format_number(
        crossfit["crossfit_pooled_rank_auroc"], "auroc"
    )
    locked_auroc = formatting.format_number(
        crossfit["locked_in_sample_auroc"], "auroc"
    )
    for column, color, linestyle, label in (
        (
            "p_crossfit_within_fold_rank",
            plotting.PALETTE["pink"],
            "-",
            f"Cross-fitted (AUROC {crossfit_auroc})",
        ),
        (
            "p_locked_in_sample",
            plotting.PALETTE["gray"],
            "--",
            f"Locked model, partly in-sample (AUROC {locked_auroc})",
        ),
    ):
        false_positive_rate, true_positive_rate, _ = roc_curve(
            labels, predictions[column].values
        )
        ax.plot(
            false_positive_rate,
            true_positive_rate,
            color=color,
            linestyle=linestyle,
            label=label,
        )
    ax.plot(
        [0, 1],
        [0, 1],
        color=plotting.PALETTE["gray"],
        linestyle=":",
        label="Chance",
```

```python
    )
    ax.set_xlabel("False positive rate")
    ax.set_ylabel("True positive rate")
    ax.legend(
        loc="lower right",
        fontsize="x-small",
        handlelength=1.5,
        borderaxespad=0.3,
        frameon=True,
        framealpha=0.9,
    )


def _plot_estimate_comparison(
    ax, folds: pd.DataFrame, crossfit: dict, demographics: dict
) -> None:
    rows = _estimate_rows(folds, crossfit, demographics)
    for position, (label, values, lower, upper) in enumerate(rows):
        color = (
            plotting.PALETTE["gray"]
            if label == "Age + sex alone"
            else plotting.PALETTE["pink"]
        )
        if len(values) > 1:
            ax.scatter(
                values,
                [position] * len(values),
                color=plotting.PALETTE["sky"],
                s=22,
                zorder=3,
                edgecolors="none",
            )
            continue
        ax.scatter(
            values, [position], color=color, s=42, zorder=3, edgecolors="none"
        )
        if lower is not None:
            ax.plot(
                [lower, upper],
                [position, position],
                color=color,
                linewidth=1.4,
                zorder=2,
            )
    ax.axvline(
        0.5, color=plotting.PALETTE["gray"], linestyle=":", label="Chance"
    )
    ax.set_yticks(range(len(rows)))
    ax.set_yticklabels([row[0] for row in rows], fontsize=8)
    ax.set_xlim(0.4, 0.95)
    ax.set_xlabel("AUROC, sepsis vs. non-infected ICU controls")
    ax.set_ylim(-0.6, len(rows) - 0.4)
    ax.legend(loc="lower left")


def _estimate_rows(
    folds: pd.DataFrame, crossfit: dict, demographics: dict
) -> list[tuple[str, list[float], float | None, float | None]]:
    matched = {
        entry["contrast"]: entry for entry in demographics["matched_analysis"]
    }[SPECIFICITY_KEY]
    incremental = {
        entry["contrast"]: entry
        for entry in demographics["incremental_discrimination"]
    }[SPECIFICITY_KEY]
    return [
        ("Per-fold estimates", folds["auroc"].tolist(), None, None),
        (
            "Cross-fitted mean",
            [crossfit["crossfit_fold_auroc_mean"]],
            crossfit["crossfit_fold_auroc_ci_lo"],
            crossfit["crossfit_fold_auroc_ci_hi"],
        ),
        (
            "Age/sex matched",
            [matched["matched_auroc"]],
```

```python
            matched["matched_ci_lo"],
            matched["matched_ci_hi"],
        ),
        (
            "Age + sex alone",
            [incremental["auroc"]["demography_only"]],
            None,
            None,
        ),
    ]


if __name__ == "__main__":
    main()
```

28 src/fig_design_schematic.py

```python
from __future__ import annotations

import json
import logging
from dataclasses import dataclass
from pathlib import Path

import matplotlib.pyplot as plt
import pandas as pd
from matplotlib.patches import FancyArrowPatch, FancyBboxPatch

import plotting
from config import (
    FIGURES_DIR,
    METRICS_DIR,
    PROCESSED_DATA_DIR,
    configure_logging,
)
from formatting import format_count

logger = logging.getLogger(__name__)

PALE = "#f4f6f8"
EVALUATION_BOTTOM = 0.055
EVALUATION_HEIGHT = 0.185


@dataclass
class DesignCounts:

    sepsis: int
    healthy: int
    noninfected: int
    validation: int
    validation_sepsis: int
    discovery_genes: int
    transportable_genes: int
    absent_genes: int
    signature_genes: int
    modal_c: float
    crossfit: dict
    random_panels: dict
    label_permutation: dict


def main() -> None:
    configure_logging()
    counts = load_design_counts()
    plotting.apply_style()
    fig, ax = plt.subplots(figsize=(plotting.FULL_WIDTH_INCHES * 2, 6.4))
    ax.set_xlim(0, 1)
    ax.set_ylim(0, 1)
    ax.axis("off")
    draw_sources_and_audit(ax, counts)
    draw_discovery_groups(ax, counts)
    draw_selection_and_lock(ax, counts)
```

```
    draw_evaluations(ax, counts)
    plotting.save_figure(fig, FIGURES_DIR, "fig1_design_schematic")
    plt.close(fig)
    logger.info("wrote fig1_design_schematic")


def load_design_counts() -> DesignCounts:
    discovery_groups = (
        pd.read_csv(PROCESSED_DATA_DIR / "discovery_labels.csv", index_col=0)[
            "group"
        ]
        .value_counts()
        .to_dict()
    )
    validation_labels = pd.read_csv(
        PROCESSED_DATA_DIR / "validation_labels.csv", index_col=0
    )
    transportable = pd.read_csv(PROCESSED_DATA_DIR / "transportable_genes.csv")
    locked_model = _read_json(PROCESSED_DATA_DIR / "locked_model.json")
    audit = _read_json(METRICS_DIR / "transport_audit_summary.json")
    return DesignCounts(
        sepsis=discovery_groups["sepsis"],
        healthy=discovery_groups["healthy"],
        noninfected=discovery_groups["noninfected_icu"],
        validation=len(validation_labels),
        validation_sepsis=int((validation_labels["group"] == "sepsis").sum()),
        discovery_genes=int(audit["n_discovery_genes"]),
        transportable_genes=len(transportable),
        absent_genes=int(audit["n_absent_from_validation"]),
        signature_genes=len(locked_model["signature_genes"]),
        modal_c=locked_model["modal_C"],
        crossfit=_read_json(METRICS_DIR / "specificity_crossfit_summary.json"),
        random_panels=_read_json(
            METRICS_DIR / "random_panel_benchmark_summary.json"
        ),
        label_permutation=_read_json(
            METRICS_DIR / "label_permutation_summary.json"
        ),
    )


def _read_json(path: Path) -> dict:
    with open(path, encoding="utf-8") as json_file:
        return json.load(json_file)


def draw_sources_and_audit(ax, counts: DesignCounts) -> None:
    palette = plotting.PALETTE
    labeled = counts.sepsis + counts.healthy + counts.noninfected
    _box(
        ax,
        (0.02, 0.86, 0.44, 0.11),
        f"Discovery: GSE65682 (HG-U219)\n802 samples $\\rightarrow$ "
        f"{labeled} with an admission-diagnosis label",
        PALE,
        palette["blue"],
        weight="bold",
    )
    _box(
        ax,
        (0.54, 0.86, 0.44, 0.11),
        f"Validation: GSE57065 (HG-U133 Plus 2.0)\n"
        f"{counts.validation} analyzed ({counts.validation_sepsis} septic "
        f"shock, {counts.validation - counts.validation_sepsis} healthy)",
        PALE,
        palette["orange"],
        weight="bold",
    )
    _box(
        ax,
        (0.20, 0.735, 0.60, 0.075),
        f"Cross-platform feature-availability audit, run before "
        f"selection\n{format_count(counts.discovery_genes)} discovery genes "
        f"$\\rightarrow$ {format_count(counts.transportable_genes)} "
        f"measurable on both platforms "
        f"({format_count(counts.absent_genes)} dropped)",
```

```python
        "#fff8e8",
        palette["gray"],
    )
    _arrow(ax, (0.24, 0.86), (0.32, 0.812), palette["gray"])
    _arrow(ax, (0.76, 0.86), (0.68, 0.812), palette["gray"])


def _box(
    ax,
    bounds: tuple[float, float, float, float],
    text: str,
    face: str,
    edge: str,
    weight: str = "normal",
) -> None:
    x, y, width, height = bounds
    ax.add_patch(
        FancyBboxPatch(
            (x, y),
            width,
            height,
            boxstyle="round,pad=0.008",
            linewidth=0.9,
            facecolor=face,
            edgecolor=edge,
        )
    )
    ax.text(
        x + width / 2,
        y + height / 2,
        text,
        ha="center",
        va="center",
        fontsize=7.2,
        fontweight=weight,
        linespacing=1.35,
        zorder=5,
    )


def _arrow(
    ax,
    start: tuple[float, float],
    end: tuple[float, float],
    color: str,
) -> None:
    ax.add_patch(
        FancyArrowPatch(
            start,
            end,
            arrowstyle="-|>",
            mutation_scale=9,
            linewidth=0.9,
            color=color,
            shrinkA=1.5,
            shrinkB=1.5,
        )
    )


def draw_discovery_groups(ax, counts: DesignCounts) -> None:
    palette = plotting.PALETTE
    gray = palette["gray"]
    _box(
        ax,
        (0.02, 0.585, 0.28, 0.095),
        f"Sepsis\n$n$ = {counts.sepsis}",
        "#eaf2fb",
        palette["blue"],
    )
    _box(
        ax,
        (0.33, 0.585, 0.28, 0.095),
        f"Healthy\n$n$ = {counts.healthy}",
        "#eaf7ee",
        palette["green"],
```

```
    )
    _box(
        ax,
        (0.64, 0.585, 0.34, 0.095),
        f"Non-infected ICU controls\n$n$ = {counts.noninfected}\n"
        f"not used in selection or fitting",
        "#f2eefa",
        palette["pink"],
    )
    branch_positions = (0.16, 0.47, 0.81)
    for x in branch_positions:
        ax.plot([x, x], [0.712, 0.700], color=gray, linewidth=0.9)
    ax.plot([0.16, 0.81], [0.712, 0.712], color=gray, linewidth=0.9)
    ax.plot([0.50, 0.50], [0.735, 0.712], color=gray, linewidth=0.9)
    for x in branch_positions:
        _arrow(ax, (x, 0.705), (x, 0.681), gray)


def draw_selection_and_lock(ax, counts: DesignCounts) -> None:
    palette = plotting.PALETTE
    red = palette["red"]
    _box(
        ax,
        (0.06, 0.425, 0.52, 0.115),
        f"Selection, discovery only, {counts.sepsis + counts.healthy} sepsis "
        f"+ healthy samples\n"
        f"5 outer $\\times$ 5 inner nested CV; inside every outer training "
        f"fold:\n"
        f"top-300 |Welch $t$| filter, then L1-logistic, $C$ tuned in the "
        f"inner loop",
        "#eaf2fb",
        palette["blue"],
    )
    _arrow(ax, (0.16, 0.585), (0.22, 0.541), palette["blue"])
    _arrow(ax, (0.47, 0.585), (0.42, 0.541), palette["green"])

    ax.plot(
        [0.01, 0.99], [0.392, 0.392], color=red, linewidth=1.4, linestyle="--"
    )
    ax.text(
        0.975,
        0.399,
        "Model locked: gene list, scaler, and coefficients fixed; "
        "nothing below is refitted",
        ha="right",
        va="bottom",
        fontsize=7.0,
        color=red,
        fontweight="bold",
    )
    _box(
        ax,
        (0.20, 0.285, 0.60, 0.075),
        f"Locked signature: {counts.signature_genes} genes with non-zero "
        f"coefficients at the modal $C$ = {counts.modal_c}\n"
        f"(panel size set by L1, not fixed in advance)",
        "#fdecec",
        red,
    )
    _arrow(ax, (0.32, 0.425), (0.36, 0.361), red)


def draw_evaluations(ax, counts: DesignCounts) -> None:
    palette = plotting.PALETTE
    crossfit = counts.crossfit
    benchmark = {
        row["task"]: row for row in counts.random_panels["primary_cell"]
    }
    permutation = {
        row["task"]: row for row in counts.label_permutation["results"]
    }
    lower = crossfit["crossfit_fold_auroc_ci_lo"]
    upper = crossfit["crossfit_fold_auroc_ci_hi"]
    evaluations = (
        (
            0.015,
```

```python
            0.23,
            f"1. External validation\nGSE57065, $n$ = {counts.validation}\n"
            f"locked model, no refit\n\nAUROC 1.000\nno two-sided CI\n"
            f"estimable at the ceiling",
            PALE,
            palette["orange"],
        ),
        (
            0.26,
            0.23,
            f"2. Specificity, cross-fitted\nsepsis vs. non-infected ICU\n"
            f"$n$ = {counts.sepsis + counts.noninfected}, fully held out\n\n"
            f"AUROC {crossfit['crossfit_fold_auroc_mean']:.3f}\n"
            f"({lower:.3f-}{upper:.3f})",
            "#f2eefa",
            palette["pink"],
        ),
        (
            0.505,
            0.23,
            f"3. Random-panel benchmark\n"
            f"{format_count(counts.random_panels['n_draws_per_cell'])} "
            f"random "
            f"{counts.signature_genes}-gene panels\nidentical recipe\n\n"
            f"validation $p$ = "
            f"{benchmark['validation']['empirical_p']:.3f}\n"
            f"specificity $p$ = "
            f"{benchmark['specificity']['empirical_p']:.3f}\n"
            f"tests the gene set",
            "#eaf7ee",
            palette["green"],
        ),
        (
            0.75,
            0.235,
            f"4. Label permutation\n"
            f"{format_count(counts.label_permutation['n_permutations'])} "
            f"permutations\n"
            f"whole pipeline re-run\n\n"
            f"validation $p$ = "
            f"{permutation['validation']['empirical_p']:.3f}\n"
            f"specificity $p$ = "
            f"{permutation['specificity']['empirical_p']:.3f}\n"
            f"tests against chance",
            "#eaf2fb",
            palette["blue"],
        ),
    )
    for x, width, text, face, edge in evaluations:
        _box(
            ax,
            (x, EVALUATION_BOTTOM, width, EVALUATION_HEIGHT),
            text,
            face,
            edge,
        )
    _draw_evaluation_arrows(ax)


def _draw_evaluation_arrows(ax) -> None:
    palette = plotting.PALETTE
    top = EVALUATION_BOTTOM + EVALUATION_HEIGHT
    for x_start, x_end in (
        (0.28, 0.13),
        (0.42, 0.375),
        (0.58, 0.62),
        (0.72, 0.87),
    ):
        _arrow(ax, (x_start, 0.285), (x_end, top), palette["gray"])
    ax.plot(
        [0.985, 0.985],
        [0.585, 0.258],
        color=palette["pink"],
        linewidth=0.9,
        linestyle=":",
    )
```

```python
    ax.plot(
        [0.985, 0.375],
        [0.258, 0.258],
        color=palette["pink"],
        linewidth=0.9,
        linestyle=":",
    )
    _arrow(ax, (0.375, 0.258), (0.375, top + 0.002), palette["pink"])


if __name__ == "__main__":
    main()
```

29 scripts/run_all.py

```python
from __future__ import annotations

import argparse
import hashlib
import logging
import shutil
import subprocess
import sys
import time
from pathlib import Path

logger = logging.getLogger(__name__)

PROJECT_ROOT = Path(__file__).resolve().parents[1]
SOURCE_DIR = PROJECT_ROOT / "src"
STATE_DIR = PROJECT_ROOT / "data" / "interim" / "run_state"
RULE_WIDTH = 70
SECONDS_PER_MINUTE = 60

STEPS = [
    "parse_platforms.py",
    "build_discovery.py",
    "build_validation.py",
    "validation_metadata_extra.py",
    "transport_audit.py",
    "quantify_batch_platform.py",
    "differential_expression.py",
    "nested_cv_signature.py",
    "specificity_check.py",
    "specificity_crossfit.py",
    "batch_source_structure.py",
    "external_validation.py",
    "random_panel_benchmark.py",
    "label_permutation_test.py",
    "pipeline_bootstrap.py",
    "clinical_metrics.py",
    "demographic_sensitivity.py",
    "enrichment_analysis.py",
    "table1_cohorts.py",
    "make_summary_figures.py",
    "make_figures_3_4.py",
    "fig_design_schematic.py",
]


def main() -> None:
    logging.basicConfig(level=logging.INFO, format="%(message)s")
    args = parse_arguments()
    if args.list:
        list_steps()
        return
    code_hash = hash_source_files()
    prepare_state(args.resume, code_hash)
    last = len(STEPS) if args.stop_after is None else args.stop_after
    for step in STEPS[:last]:
        if locate_marker(step).exists():
            logger.info("skip (finished earlier): %s", step)
            continue
```

```
        run_step(step)
        locate_marker(step).write_text(code_hash, encoding="utf-8")
    remaining = [step for step in STEPS if not locate_marker(step).exists()]
    if remaining:
        logger.info(
            "\nStopped with %d step(s) left. Continue with --resume.",
            len(remaining),
        )
    else:
        logger.info("\nAll steps completed.")


def parse_arguments() -> argparse.Namespace:
    parser = argparse.ArgumentParser(
        description="Run the analysis pipeline in order."
    )
    parser.add_argument(
        "--resume",
        action="store_true",
        help="skip steps that finished in an earlier run of the same code",
    )
    parser.add_argument(
        "--stop-after",
        type=int,
        choices=range(1, len(STEPS) + 1),
        metavar="N",
        help="stop after step N (see --list), to run in chunks",
    )
    parser.add_argument(
        "--list", action="store_true", help="list steps and their state"
    )
    return parser.parse_args()


def list_steps() -> None:
    for number, step in enumerate(STEPS, start=1):
        state = "done" if locate_marker(step).exists() else "    "
        logger.info("%2d  %s  %s", number, state, step)


def hash_source_files() -> str:
    digest = hashlib.sha256()
    for path in sorted(SOURCE_DIR.glob("*.py")) + [Path(__file__)]:
        digest.update(path.name.encode("utf-8"))
        digest.update(path.read_bytes())
    return digest.hexdigest()


def locate_marker(step: str) -> Path:
    return STATE_DIR / f"{step}.done"


def prepare_state(resume: bool, code_hash: str) -> None:
    if resume and STATE_DIR.exists():
        stale = sorted(
            path.name
            for path in STATE_DIR.glob("*.done")
            if path.read_text(encoding="utf-8") != code_hash
        )
        if stale:
            raise RuntimeError(
                "The code changed since these steps finished: "
                f"{', '.join(stale)}. Run again without --resume."
            )
        return
    if STATE_DIR.exists():
        shutil.rmtree(STATE_DIR)
    STATE_DIR.mkdir(parents=True)


def run_step(step: str) -> None:
    logger.info("\n%s\n%s\n%s", "=" * RULE_WIDTH, step, "=" * RULE_WIDTH)
    started = time.monotonic()
    result = subprocess.run(
        [sys.executable, str(SOURCE_DIR / step)],
        cwd=str(SOURCE_DIR),
```

```python
        check=False,
    )
    if result.returncode != 0:
        logger.error("FAILED at %s (exit %s)", step, result.returncode)
        logger.error("Fix the cause, then continue with --resume.")
        sys.exit(result.returncode)
    minutes = (time.monotonic() - started) / SECONDS_PER_MINUTE
    logger.info("%s finished in %.1f min", step, minutes)


if __name__ == "__main__":
    main()
```
