# Supplementary material for: What a Near-Perfect AUROC Does Not Show: Random-Panel and Label-Permutation Controls for a Sepsis Blood-Transcriptome Signature
Source: Int J Mol Sci. 2026 Oct 19;27(20):9273. doi: 10.3390/ijms27209273 (PMC13704978; doi:10.3390/ijms27209273)
Supplement: Supplementary file 1 [file ijms-27-09273-s001.zip › Supplement_S2_Tables_CEL_R2.pdf]

Supplementary Tables

Tables S1, S3, S6 and S8 are given here as a defined subset, stated in each caption. Every row of all four is in the data workbook, Supplementary File S3. Every other table is given in full.

Table S1. Differential-expression results for the discovery cohort (GSE65682, sepsis, $n = 242$, vs. healthy, $n = 42$), across all 9983 transportable genes tested (Welch two-sample t-test, unequal variance, Benjamini-Hochberg FDR correction; Methods 4.5). Sorted by ascending FDR-adjusted p. Listed here are the 1508 genes at FDR $p < 0.05$ with an absolute $\log_2$ fold change of at least 1, the differential-expression set the manuscript analyses; all 9983 tested genes are in the data workbook, Supplementary File S3.

gene_symbol	log2fc	t_stat	p_raw	p_adj	mean_sepsis	mean_healthy
STOM	2.513	26.92	0	0	9.465	6.952
TP53I3	2.155	24.69	0	0	5.916	3.761
OLAH	3.021	25.6	0	0	5.053	2.031
NFATC2IP	-1.51	-27.2	0	0	6.039	7.55
DUSP13	1.472	19.57	0	0	4.059	2.587
NFATC2	-2.465	-26.87	0	0	5.442	7.907
BANP	-1.102	-19.17	0	0	6.264	7.366
SULF2	-2.586	-21.04	0	0	6.203	8.789
RNASEH2B	-1.587	-21.96	0	0	4.675	6.261
PECR	1.599	21.37	0	0	4.885	3.286
MMRN1	1.743	17.8	0	0	4.609	2.866
PGS1	1.859	27.01	0	0	9.631	7.772
LEF1	-3.703	-29.43	0	0	4.225	7.928
BCL11B	-3.489	-34.77	0	0	3.638	7.128
LDHB	-2.282	-27.98	0	0	7.679	9.961
LDHA	1.268	20.09	0	0	10.83	9.558
ARHGEF18	-1.436	-27.58	0	0	7.296	8.732
SAMD8	1.02	19.44	0	0	3.563	2.543
ZFP36L2	-1.99	-25.33	0	0	7.777	9.767
CCND2	-2.341	-27.01	0	0	6.19	8.531
IKBKB	-1.22	-22.2	0	0	5.579	6.799
TRAF5	-2.499	-28.06	0	0	3.719	6.218
PPTC7	-1.328	-21.22	0	0	6.532	7.86
GIMAP1	-1.521	-23.25	0	0	5.333	6.854
SDAD1	-1.657	-25.76	0	0	5.115	6.772
IRAK3	2.402	27.89	0	0	8.668	6.265
PLB1	1.63	19.11	0	0	4.712	3.082
CARD6	1.99	24.57	0	0	7.146	5.156
TRBC1	-3.089	-30.98	0	0	6.958	10.05
RORA	-2.328	-25.68	0	0	4.069	6.397
LIN7A	2.204	25.25	0	0	5.413	3.21
LFNG	-1.623	-26.53	0	0	4.22	5.843
SET	-1.122	-23.1	0	0	7.78	8.901
CD27	-2.54	-28.06	0	0	5.397	7.937
RCC2	-1.306	-22.82	0	0	6.213	7.519
SLC25A6	-1.273	-23.3	0	0	9.191	10.46
RPL22	-1.254	-23.5	0	0	9.902	11.16
FYN	-1.852	-26.15	0	0	5.718	7.569
RPL14	-1.258	-28	0	0	9.837	11.1
STAT4	-2.275	-24.64	0	0	5.219	7.494
RPL10A	-1.386	-24.68	0	0	9.4	10.79
PDCD4	-1.743	-26.11	0	0	6.737	8.48
MS4A4A	3.325	26.82	0	0	7.386	4.061
MMP8	5.872	25.74	0	0	8.429	2.557
CKAP4	2.219	30.95	0	0	9.66	7.441
NLRC4	2.483	26.17	0	0	6.89	4.407
DGKZ	-1.251	-25.29	0	0	7.981	9.232
JMJD6	1.436	20.59	0	0	5.719	4.283
UPP1	2.467	36.34	0	0	9.281	6.814
MKNK1	1.83	25.03	0	0	7.484	5.654
FAM20A	1.614	17.37	0	0	3.709	2.095
EVL	-2.456	-35.35	0	0	4.768	7.225
ASPH	2.81	24.3	0	0	6.137	3.327
LYSMD2	-1.708	-18.11	0	0	5.925	7.633
OPLAH	2.473	25.1	0	0	5.319	2.846
PLEKHA1	-2.53	-30.22	0	0	3.496	6.026
SLC26A8	1.735	25.01	0	0	4.264	2.529
LBH	-2.416	-26.51	0	0	5.076	7.492

gene_symbol	log2fc	t_stat	p_raw	p_adj	mean_sepsis	mean_healthy
LCK	-2.759	-29.99	0	0	4.493	7.252
ZNF275	-1.722	-24.27	0	0	4.137	5.859
CLEC1B	2.009	19.51	0	0	5.935	3.926
CLEC4D	4.667	30.49	0	0	8.151	3.484
HLA-DPB1	-2.478	-26.85	0	0	6.846	9.324
CD177	6.76	43.28	0	0	8.734	1.974
CD3G	-2.574	-26.58	0	0	4.461	7.035
RASGRP1	-2.788	-30.61	0	0	5.527	8.315
FGF13	2.119	17.23	0	0	4.513	2.394
ST6GALNAC3	2.161	23.83	0	0	4.699	2.538
ST6GAL1	-2.112	-25.59	0	0	6.12	8.232
CBX7	-1.574	-26.37	0	0	5.33	6.904
TTC39C	-2.273	-31.65	0	0	3.603	5.876
IL23A	-2.844	-30.6	0	0	7.761	10.61
IMPDH2	-1.872	-23.29	0	0	5.743	7.616
ATP9A	2.143	22.8	0	0	4.43	2.287
TAGAP	-2.083	-22.88	0	0	6.789	8.872
RAB32	1.67	26.07	0	0	7.824	6.154
NLRP1	-1.734	-22.86	0	0	5.067	6.8
CCDC88C	-2.14	-30.17	0	0	3.65	5.791
ETS1	-2.557	-26.45	0	0	7.52	10.08
GRB10	3.011	31.75	0	0	6.401	3.39
HLA-DMB	-2.679	-27.08	0	0	6.48	9.159
HLA-DMA	-2.459	-24.61	0	0	5.898	8.357
HK3	2.632	33.59	0	0	9.177	6.546
PHF20	-1.128	-18.93	0	0	5.255	6.383
GIMAP4	-1.793	-22.34	0	0	7.503	9.296
KCNE1	1.464	19.2	0	0	3.991	2.527
IL1R2	3.841	30.95	0	0	11.09	7.251
P2RY8	-1.868	-23.73	0	0	6.386	8.254
FBXO21	-2.011	-27.32	0	0	3.751	5.762
CCR7	-2.633	-23.03	0	0	6.071	8.704
SGK1	-2.668	-27.06	0	0	4.259	6.927
CD2	-2.61	-27.59	0	0	5.345	7.955
HLA-DPA1	-2.396	-30.46	0	0	9.426	11.82
AGFG1	2.138	28.95	0	0	7.855	5.717
TNFAIP2	-2.033	-19.6	0	0	6.33	8.363
GPR183	-2.865	-28.62	0	0	4.433	7.297
GPR18	-2.679	-27.66	0	0	4.708	7.387
RFX5	-1.996	-29.51	0	0	3.875	5.871
PRKCQ	-2.65	-34.01	0	0	3.486	6.136
NONO	-1.203	-30.68	0	0	8.498	9.702
PPP1R3D	1.275	23.31	0	0	6.324	5.049
DENND2D	-1.741	-26.71	0	0	6.925	8.666
DPP4	-2.875	-31.19	0	0	3.146	6.021
BMX	3.113	28.41	0	0	6.235	3.122
SKAP1	-2.644	-29.78	0	0	5.279	7.923
ATP6V1C1	1.833	20.59	0	0	7.335	5.502
ATP8B2	-2.626	-32.87	0	0	4.989	7.616
RFTN1	-2.421	-28.96	0	0	4.685	7.106
HLA-DRA	-1.787	-24.04	0	0	9.648	11.43
CTSO	-1.889	-24.71	0	0	4.331	6.22
CUTA	-1.362	-23.14	0	0	7.066	8.428
NOB1	-1.698	-26.4	0	0	4.292	5.99
IL7R	-2.897	-27.37	0	0	6.229	9.126
IMP3	-1.763	-27.77	0	0	5.887	7.65
LPXN	-1.756	-29.22	0	0	7.164	8.92
GLTSCR2	-1.665	-28.52	0	0	9.706	11.37
ARL4C	-2.813	-29.26	0	0	5.846	8.659
NARS	-1.211	-21.07	0	0	6.854	8.065
RPS25	-1.068	-22.36	0	0	9.763	10.83
ERP29	-1.073	-22.6	0	0	8.616	9.69
ESYT1	-2.414	-29.92	0	0	5.903	8.317
ABLIM1	-2.827	-27.66	0	0	5.358	8.185
SULT1B1	2.414	27.22	0	0	5.395	2.981
PRPS1	-1.577	-26.26	0	0	5.346	6.924
RAB10	1.111	25.95	0	0	7.991	6.88
NHP2L1	-1.473	-25.78	0	0	5.093	6.566

gene_symbol	log2fc	t_stat	p_raw	p_adj	mean_sepsis	mean_healthy
APOL3	-2.129	-25.01	0	0	4.417	6.546
RUNX3	-2.208	-27.09	0	0	5.795	8.003
HVCN1	-2.278	-29.87	0	0	5.838	8.116
TMEM109	-1.665	-30.85	0	0	4.349	6.015
SPOCK2	-2.863	-29.4	0	0	5.415	8.278
CD8A	-2.784	-20.6	0	0	5.407	8.19
IARS	-1.841	-22.71	0	0	5.429	7.27
GALNT14	2.216	22.24	0	0	4.823	2.607
FUS	-1.21	-24.23	0	0	8.458	9.668
GPR84	4.555	34.1	0	0	7.274	2.719
RGL4	3.276	27.05	0	0	9.142	5.866
RARRES3	-2.212	-19.48	0	0	5.89	8.102
TSPO	1.536	24.22	0	0	10.83	9.291
TDRD9	2.441	24.62	0	0	4.679	2.238
RPS6KA5	-2.824	-30.51	0	0	4.624	7.449
RPS4X	-1.369	-25.31	0	0	9.612	10.98
ZDHHC19	3.038	23.25	0	0	7.026	3.988
DACH1	2.788	24.27	0	0	5.658	2.869
SYNE2	-1.501	-19.79	0	0	5.75	7.25
SORT1	2	23.12	0	0	7.365	5.364
SH2B3	-1.598	-24.21	0	0	5.635	7.233
LTB	-1.527	-23.66	0	0	8.609	10.14
SERPINB1	1.929	31.12	0	0	10.61	8.676
ITK	-2.867	-28.78	0	0	4.208	7.075
GIMAP6	-1.949	-25.29	0	0	5.586	7.535
ITGB7	-2.169	-25.68	0	0	3.766	5.935
CD63	1.178	23.85	0	0	10.38	9.198
HP1BP3	-1.295	-24.41	0	0	7.388	8.683
AKR1B1	-1.362	-24.73	0	0	5.417	6.78
AQP3	-2.106	-25.7	0	0	3.189	5.295
SIGLEC9	1.279	18.97	0	0	5.969	4.689
DCTD	-1.691	-27.12	0	0	4.773	6.464
BTN3A3	-2.398	-24.56	0	0	4.182	6.58
DNMT1	-2.211	-28.54	0	0	5.488	7.699
METTL7B	2.155	20.27	0	0	4.372	2.218
PPIL2	-1.449	-25.24	0	0	5.129	6.578
GADD45A	3.774	36.78	0	0	8.326	4.551
HNRNPA1	-1.247	-23.45	0	0	9.694	10.94
PRKCH	-2.595	-28.76	0	0	5.679	8.275
METAP1	-1.589	-28.04	0	0	4.903	6.491
UGCG	2.752	28.38	0	0	8.137	5.385
TRIM44	-1.811	-27.41	0	0	5.03	6.841
ALDH9A1	-1.217	-22.02	0	0	6.418	7.635
FGL2	-1.602	-18.67	0	0	8.74	10.34
PIK3IP1	-2.104	-24.83	0	0	5.775	7.879
PTGER4	-2.314	-20.76	0	0	5.558	7.871
CX3CR1	-2.764	-25.05	0	0	7.695	10.46
CDC25B	-2.24	-27.17	0	0	4.737	6.976
U2AF2	-1.406	-26.08	0	0	6.537	7.943
EEF2	-1.037	-26.78	0	0	10.62	11.66
CD5	-2.386	-29.46	0	0	4.012	6.398
CD74	-2.337	-30.52	0	0	7.068	9.405
NSUN7	1.99	21.82	0	0	5.447	3.457
EIF3D	-1.051	-27.57	0	0	7.852	8.903
EIF3F	-1.216	-25.39	0	0	8.796	10.01
DDAH2	2.197	30.03	0	0	6.658	4.461
UNC119B	-1.394	-25.66	0	0	5.406	6.799
HNRNPR	-1.568	-27.39	0	0	6.557	8.124
FAIM3	-2.963	-29.79	0	0	4.822	7.785
PFKFB2	3.638	28.35	0	0	6.528	2.889
NFATC3	-1.644	-24.55	0	0	5.618	7.262
MPEG1	-1.99	-21.96	0	0	7.502	9.492
TCF7	-3.472	-31.65	0	0	6.053	9.525
NHP2	-1.317	-20.94	0	0	5.913	7.23
CPVL	-2.185	-19.03	0	0	6.487	8.673
GYG1	3.054	35.69	0	0	9.904	6.849
EXOSC4	2.72	28.21	0	0	7.108	4.389
HGF	1.505	18.06	0	0	4.102	2.596

gene_symbol	log2fc	t_stat	p_raw	p_adj	mean_sepsis	mean_healthy
ACSS1	-1.464	-25.53	0	0	5.282	6.746
TIAM1	-2.458	-27.78	0	0	3.66	6.117
DDX24	-1.885	-27.75	0	0	5.364	7.249
ZDHHC3	1.601	24.28	0	0	6.628	5.028
ATP2B1	-1.857	-20.32	0	0	5.642	7.499
NPM1	-1.518	-23.92	0	0	8.451	9.969
IL2RB	-3.409	-28.64	0	0	5.246	8.654
CECR1	-2.177	-22.59	0	0	5.853	8.031
SLC38A1	-2.262	-25.62	0	0	6.686	8.948
LRRC47	-1.263	-24.99	0	0	5.624	6.887
CEACAM1	2.816	22.1	0	0	7.762	4.946
CDK5RAP2	2.472	21.53	0	0	6.907	4.435
RRP1B	-2.33	-27.93	0	0	4.009	6.339
EIF4B	-1.378	-25.29	0	0	6.894	8.272
MAP2K6	2.708	29.94	0	0	6.624	3.916
HPGD	2.958	23.97	0	0	5.362	2.404
IL32	-2.248	-25.44	0	0	4.327	6.576
EIF3L	-1.468	-24.44	0	0	8.053	9.521
RANGRF	-1.385	-22.76	0	0	5.556	6.941
UTP6	-1.291	-22.19	0	0	5.793	7.084
ENTPD7	1.887	19.05	0	0	3.907	2.02
PEBP1	-2.09	-25.61	3e-45	1.186e-43	4.073	6.163
ACER3	2.075	20.7	3e-45	1.186e-43	6.607	4.531
DRAM1	1.778	21.9	3e-45	1.186e-43	4.802	3.024
RPL18	-1.265	-23.05	3e-45	1.186e-43	9.772	11.04
EIF2B1	-1.205	-21.01	3e-45	1.186e-43	5.825	7.03
NDST2	1.189	17.95	3e-45	1.186e-43	6.15	4.961
AES	-1.485	-23.53	3e-45	1.186e-43	6.83	8.315
KARS	-1.052	-21.89	3e-45	1.186e-43	8.107	9.159
CCT4	-1.396	-21.14	3e-45	1.186e-43	6.641	8.037
PDSS1	2.029	18.12	3e-45	1.186e-43	5.277	3.247
FBL	-1.573	-21.74	3e-45	1.186e-43	6.305	7.878
CLEC5A	2.326	20.15	6e-45	2.303e-43	4.926	2.6
UBE2G2	-1.284	-23.41	6e-45	2.303e-43	5.461	6.745
KLRG1	-3.089	-25.96	6e-45	2.303e-43	3.535	6.624
ESYT2	-1.81	-24.53	6e-45	2.303e-43	4.672	6.482
IDI1	2.497	19.98	6e-45	2.303e-43	6.921	4.424
TAF15	-1.533	-24.45	6e-45	2.303e-43	5.428	6.96
EPRS	-1.4	-22.47	6e-45	2.303e-43	4.933	6.332
AHNAK	-1.743	-19.91	1.1e-44	4.568e-43	7.259	9.001
NCL	-1.822	-24.29	1.1e-44	4.568e-43	7.404	9.226
ARL4A	1.762	22.04	1.7e-44	6.824e-43	6.613	4.851
RABGEF1	1.685	20.66	2e-44	7.929e-43	6.971	5.285
PUF60	-1.067	-22.52	2.2e-44	9.025e-43	6.794	7.861
PGD	1.805	23.76	2.5e-44	1.011e-42	9.496	7.691
WDR82	-1.305	-27.64	2.8e-44	1.115e-42	7.874	9.179
TGFBI	-2.261	-18.98	3.9e-44	1.548e-42	4.975	7.236
NECAB1	1.366	16.88	3.9e-44	1.548e-42	3.229	1.863
ACSL5	-1.469	-22.08	4.2e-44	1.652e-42	4.415	5.885
LUC7L2	-1.319	-22.99	4.5e-44	1.749e-42	5.367	6.686
MSL3	1.429	19.06	4.5e-44	1.749e-42	7.378	5.949
PTPRCAP	-1.876	-21.47	4.8e-44	1.851e-42	4.989	6.865
FOXP1	-1.273	-22.08	5e-44	1.952e-42	7.785	9.058
SUMF2	-1.238	-24	5.6e-44	2.16e-42	6.005	7.243
PAQR8	-1.774	-24.87	8.1e-44	3.121e-42	4.417	6.191
HSPA9	-1.975	-22.58	9.8e-44	3.752e-42	4.35	6.325
TSHZ1	-2.061	-26.86	1.06e-43	4.058e-42	3.89	5.952
PRPF19	-1.558	-26.25	1.1e-43	4.149e-42	4.461	6.019
RAB11FIP4	-1.471	-18.44	1.18e-43	4.434e-42	4.512	5.983
ARL2BP	-1.062	-20.04	1.29e-43	4.838e-42	6.722	7.784
IL18R1	3.165	23.78	1.46e-43	5.449e-42	7.46	4.295
POR	1.424	20.27	2.1e-43	7.83e-42	5.991	4.567
PCMT1	1.441	23.17	2.16e-43	8.009e-42	8.057	6.617
BCL11A	-2.033	-23.11	2.47e-43	9.119e-42	3.832	5.865
NLRC3	-2.434	-28.13	2.63e-43	9.705e-42	4.479	6.913
MGA	-1.737	-24.61	2.89e-43	1.059e-41	4.13	5.867
KIAA0907	-1.258	-21.05	3.45e-43	1.261e-41	5.541	6.799
GZMM	-2.416	-25.96	3.84e-43	1.399e-41	3.703	6.119

gene_symbol	log2fc	t_stat	p_raw	p_adj	mean_sepsis	mean_healthy
PPM1M	1.25	18.21	4.2e-43	1.526e-41	7.183	5.933
KL	1.012	16.51	4.62e-43	1.673e-41	3.045	2.033
MAPK14	1.46	22.08	5.13e-43	1.842e-41	9.701	8.241
PHTF1	1.5	21.11	5.63e-43	2.016e-41	4.842	3.342
CRTC3	-1.276	-19.13	5.75e-43	2.048e-41	5.859	7.134
DPEP2	-1.541	-16.54	6.36e-43	2.26e-41	6.963	8.504
GPN1	-1.046	-20.78	6.39e-43	2.262e-41	5.293	6.338
ARG1	5.643	31.06	7.48e-43	2.64e-41	8.706	3.063
CRISP3	2.934	18.6	8.04e-43	2.827e-41	5.556	2.621
MYC	-2.171	-21.96	9.36e-43	3.279e-41	5.611	7.782
MFNG	-1.338	-23.69	1.029e-42	3.59e-41	4.842	6.18
RASA3	-1.811	-24.94	1.11e-42	3.86e-41	7.132	8.943
ACVR1B	1.064	18.7	1.163e-42	4.032e-41	7.282	6.218
PWP1	-1.379	-21.86	1.23e-42	4.235e-41	4.827	6.206
ITGA7	1.441	16.66	1.244e-42	4.269e-41	3.787	2.346
LILRA5	2.376	26.25	1.253e-42	4.283e-41	10	7.626
IRF8	-1.413	-19.29	1.469e-42	5.004e-41	5.768	7.181
LRRN1	1.156	17.52	1.676e-42	5.691e-41	3.635	2.48
RETN	4.351	23.24	1.78e-42	6.022e-41	9.051	4.7
HMOX2	-1.046	-22.46	2.175e-42	7.335e-41	5.542	6.589
ASTE1	-1.259	-22.34	2.2e-42	7.395e-41	3.982	5.241
NOL8	-1.254	-20.43	2.234e-42	7.483e-41	5.004	6.258
CDKN2C	1.799	19.32	2.298e-42	7.673e-41	4.681	2.883
EOMES	-2.989	-24.44	2.539e-42	8.449e-41	4.321	7.31
PDE4B	-1.922	-17.97	2.702e-42	8.961e-41	4.644	6.567
ITPKC	1.326	16.74	2.8e-42	9.255e-41	5.168	3.843
MLLT6	-1.542	-22.56	3.044e-42	9.995e-41	4.468	6.01
DDX18	-1.923	-25.64	3.114e-42	1.019e-40	4.752	6.675
HNRNPH1	-1.532	-21.68	3.15e-42	1.028e-40	5.29	6.822
PPP1R16B	-1.988	-22.87	3.19e-42	1.037e-40	3.46	5.448
PCOLCE2	1.641	16.25	3.212e-42	1.041e-40	3.684	2.043
IL10RA	-1.488	-24.5	3.287e-42	1.062e-40	6.071	7.559
P2RY10	-2.804	-31.57	3.974e-42	1.276e-40	2.643	5.447
MYCBP2	-1.334	-22.2	4.613e-42	1.476e-40	7.338	8.672
CCDC109B	-1.561	-20.28	4.96e-42	1.577e-40	6.082	7.643
AMPH	1.022	16.82	5.076e-42	1.609e-40	3.748	2.726
RPA1	-1.468	-22.59	5.168e-42	1.633e-40	6.684	8.152
SMAD3	-2.104	-27.06	5.748e-42	1.81e-40	3.539	5.644
ZNF438	1.76	22.33	5.77e-42	1.812e-40	5.627	3.868
ANKRD22	4.214	24.88	6.505e-42	2.036e-40	8.254	4.04
SH3GLB1	1.628	23.04	7.077e-42	2.208e-40	9.439	7.811
TRAF3	-1.28	-19.78	7.34e-42	2.283e-40	5.378	6.659
RPS16	-1.043	-20.39	8.963e-42	2.779e-40	10.04	11.08
TLR5	2.803	24.49	1.012e-41	3.128e-40	7.869	5.067
ATP5G2	-1.153	-22.92	1.145e-41	3.517e-40	8.189	9.342
KLRB1	-2.738	-23.98	1.288e-41	3.945e-40	3.794	6.532
SF3A3	-1.269	-22.6	1.823e-41	5.548e-40	4.919	6.188
ERLIN1	1.569	18.87	1.892e-41	5.741e-40	7.051	5.482
KHSRP	-1.127	-19.81	2.039e-41	6.149e-40	5.928	7.055
RALGAPA2	1.141	20.63	2.127e-41	6.395e-40	6.318	5.176
CD81	-1.799	-21.46	2.264e-41	6.787e-40	5.26	7.059
LPIN1	-2.187	-24.53	2.526e-41	7.527e-40	4.161	6.347
MAFG	1.455	25.2	2.521e-41	7.527e-40	7.275	5.82
HSH2D	-1.968	-18.1	2.654e-41	7.884e-40	4.582	6.55
RAB13	1.564	20.91	2.707e-41	8.019e-40	4.57	3.006
PLEKHO1	-1.394	-18.5	3.021e-41	8.922e-40	6.38	7.773
ZNF395	-1.567	-22.43	3.032e-41	8.93e-40	4.051	5.618
NOLC1	-2.069	-24.79	3.544e-41	1.04e-39	4.514	6.583
M6PR	-1.067	-21.89	3.745e-41	1.096e-39	6.428	7.495
NUP205	-1.666	-20.74	3.821e-41	1.115e-39	4.622	6.289
TRIB2	-2.573	-24.62	3.872e-41	1.127e-39	3.693	6.266
ACN9	1.576	18.39	4.035e-41	1.168e-39	5.468	3.892
COL17A1	1.947	16.6	4.03e-41	1.168e-39	4.425	2.477
NDNL2	-1.362	-24.28	4.233e-41	1.214e-39	4.783	6.145
RHOF	-1.639	-24.23	4.81e-41	1.372e-39	4.653	6.292
NAIP	2.267	21.02	5.24e-41	1.49e-39	8.546	6.279
TFF3	1.51	15.88	6.718e-41	1.9e-39	4.235	2.725
ZNF362	-1.072	-21.16	7.296e-41	2.058e-39	5.408	6.48

gene_symbol	log2fc	t_stat	p_raw	p_adj	mean_sepsis	mean_healthy
FOXN3	-1.197	-18.78	7.52e-41	2.115e-39	6.892	8.089
PEA15	-1.608	-21.7	7.739e-41	2.17e-39	5.139	6.747
ADA	-1.868	-20.79	8.451e-41	2.363e-39	4.636	6.504
OXA1L	-1.097	-20.45	9.187e-41	2.562e-39	6.64	7.737
ELAC2	-1.473	-24.86	1.086e-40	3.019e-39	5.082	6.555
RCSD1	-1.041	-19.03	1.227e-40	3.404e-39	6.464	7.505
PDXDC1	-1.24	-20.52	1.333e-40	3.685e-39	4.504	5.744
MPRIP	-1.428	-23.3	1.358e-40	3.746e-39	5.353	6.781
STXBP2	1.385	19	1.63e-40	4.484e-39	4.893	3.508
SMPDL3A	2.133	16.58	1.753e-40	4.795e-39	6.525	4.392
ADORA3	1.223	15.75	1.786e-40	4.873e-39	3.545	2.322
IL16	-1.155	-18.07	2.004e-40	5.453e-39	6.34	7.495
GTF3C2	-1.326	-24.21	2.493e-40	6.764e-39	5.906	7.232
SRPRB	-1.429	-19.49	2.703e-40	7.312e-39	4.637	6.066
UTP3	-1.278	-20.37	4.01e-40	1.073e-38	4.804	6.082
KCNMA1	1.733	15.64	4.039e-40	1.075e-38	4.647	2.914
TBC1D8	1.909	18.77	4.036e-40	1.075e-38	7.748	5.839
GIMAP7	-1.697	-19.6	4.738e-40	1.258e-38	6.863	8.56
CUL1	-1.554	-19.84	5.02e-40	1.329e-38	4.947	6.501
SLC37A3	1.79	18.61	5.046e-40	1.333e-38	5.387	3.597
LRPAP1	1.105	17.5	5.146e-40	1.356e-38	6.982	5.877
TNFRSF10B	-1.308	-17.84	5.189e-40	1.363e-38	5.93	7.238
FAM162A	-1.72	-22.85	5.396e-40	1.414e-38	3.597	5.317
TNFRSF25	-2.768	-28.09	5.508e-40	1.439e-38	3.735	6.504
ZNF573	-2.113	-25.8	7.342e-40	1.914e-38	3.291	5.403
EMB	2.384	20.43	8.518e-40	2.215e-38	6.663	4.279
CD3D	-2.073	-18.35	9.013e-40	2.337e-38	5.506	7.579
UTRN	-1.506	-20.66	9.3e-40	2.405e-38	5.278	6.785
CD96	-2.15	-28.07	9.834e-40	2.537e-38	2.879	5.03
GNLY	-3.159	-19.61	1.156e-39	2.966e-38	6.922	10.08
RASSF1	-1.436	-21.24	1.167e-39	2.988e-38	5.574	7.009
CHST11	-1.227	-16.46	1.181e-39	3.008e-38	7.016	8.243
CCL5	-1.624	-17.28	1.201e-39	3.051e-38	9.006	10.63
FAM117B	-2.228	-20.92	1.242e-39	3.147e-38	4.259	6.486
C21orf59	-1.319	-20.44	1.294e-39	3.27e-38	4.979	6.298
DDX27	-1.305	-21.64	1.393e-39	3.512e-38	4.836	6.142
MRPS6	-1.106	-19.65	1.549e-39	3.894e-38	4.709	5.814
NAAA	-1.782	-19.91	1.567e-39	3.931e-38	6.129	7.91
HP	5.14	25.52	2.074e-39	5.188e-38	8.915	3.775
MPHOSPH8	-1.018	-19.88	2.135e-39	5.329e-38	6.801	7.819
C1orf174	-1.243	-20.82	2.295e-39	5.715e-38	5.597	6.84
TTC19	-1.665	-21.99	2.368e-39	5.882e-38	4.59	6.255
MEF2C	-1.807	-20.38	2.467e-39	6.11e-38	4.792	6.599
CD244	-1.924	-22.18	2.598e-39	6.421e-38	3.524	5.448
LILRA6	2.18	23.01	2.696e-39	6.646e-38	9.632	7.452
APEX1	-1.478	-19.99	2.889e-39	7.103e-38	6.2	7.678
RPS19	-1.046	-19.21	3.127e-39	7.669e-38	9.939	10.98
NUP62	-1.484	-21.84	3.434e-39	8.402e-38	6.359	7.843
CHCHD7	1.681	16.72	4.276e-39	1.039e-37	7.128	5.447
SYNCRIP	-1.348	-20.37	4.799e-39	1.163e-37	5.768	7.115
BTN3A1	-1.433	-17.73	6.122e-39	1.476e-37	6.362	7.795
OSM	1.597	19.8	6.383e-39	1.532e-37	5.561	3.964
SOCS3	3.238	21.81	7.372e-39	1.761e-37	6.752	3.513
C21orf33	-1.185	-21.8	7.886e-39	1.879e-37	5.988	7.174
SIT1	-1.557	-21.19	8.61e-39	2.046e-37	5.32	6.877
ATXN10	-1.152	-18.86	9.287e-39	2.192e-37	6.156	7.309
NCBP2	-1.736	-22.97	9.867e-39	2.323e-37	3.44	5.176
CIRBP	-1.139	-18.02	1.001e-38	2.35e-37	6.285	7.424
SLC1A3	1.036	15.25	1.109e-38	2.6e-37	2.87	1.834
C5orf30	1.361	15.25	1.112e-38	2.6e-37	4.07	2.708
SAMSN1	3.015	22.47	1.224e-38	2.848e-37	8.977	5.961
GSTP1	-1.189	-17.89	1.819e-38	4.202e-37	6.531	7.72
KIAA1147	-2.009	-24.66	1.95e-38	4.487e-37	3.45	5.459
CDADC1	1.402	15.67	2.006e-38	4.603e-37	4.442	3.04
FAM168B	-1.219	-21	2.127e-38	4.87e-37	6.414	7.633
DCAF7	-1.11	-21.11	2.155e-38	4.922e-37	5.722	6.832
PTPN4	-1.778	-20.6	2.17e-38	4.946e-37	5.818	7.596
NIPAL3	-1.919	-24.62	2.349e-38	5.341e-37	3.91	5.828

gene_symbol	log2fc	t_stat	p_raw	p_adj	mean_sepsis	mean_healthy
SSRP1	-1.399	-21.81	2.411e-38	5.47e-37	5.731	7.13
SUPT16H	-1.18	-21.42	2.465e-38	5.579e-37	5.5	6.68
RIT1	1.487	22.01	2.746e-38	6.202e-37	6.086	4.599
CCR3	-3.594	-25.14	2.795e-38	6.299e-37	3.689	7.283
FNBP4	-1.585	-24	3.866e-38	8.673e-37	5.212	6.797
DOCK10	-2.246	-24.31	3.965e-38	8.874e-37	4.381	6.627
SRRM1	-1.317	-23.13	5.05e-38	1.123e-36	6.448	7.765
CRIP1	-1.527	-16.4	5.109e-38	1.133e-36	7	8.528
CYB561	-2.088	-23.58	5.364e-38	1.187e-36	3.877	5.965
SRP68	-1.084	-20.02	5.418e-38	1.197e-36	6.472	7.556
HMGN3	-1.448	-18.65	5.442e-38	1.199e-36	5.303	6.751
C9orf84	1.129	15.64	5.688e-38	1.251e-36	3.389	2.26
CDKN2D	1.15	23.29	5.861e-38	1.286e-36	9.289	8.14
ABCF1	-1.264	-20.33	6.311e-38	1.379e-36	4.56	5.824
LARS	-1.467	-21.46	6.476e-38	1.411e-36	4.375	5.842
TRPS1	1.328	16.67	7.328e-38	1.594e-36	5.597	4.269
REPS1	-1.516	-22.68	8.378e-38	1.814e-36	4.123	5.639
LDOC1L	-1.951	-25.4	8.737e-38	1.888e-36	3.457	5.408
SPN	-1.928	-24.69	8.781e-38	1.893e-36	5.129	7.056
BIN1	-1.646	-23.17	8.988e-38	1.934e-36	5.974	7.62
AMPD2	-1.552	-19.77	9.13e-38	1.96e-36	6.531	8.084
PTDSS1	-1.023	-18.61	9.316e-38	1.996e-36	6.752	7.775
ICAM2	-1.278	-19.68	9.367e-38	2.002e-36	6.406	7.683
KRI1	-1.249	-20.52	1.156e-37	2.467e-36	5.023	6.272
ZNF609	-1.195	-18.53	1.221e-37	2.6e-36	4.382	5.577
CDC42EP3	1.301	19.11	1.276e-37	2.709e-36	9.03	7.73
IMMT	-1.409	-18.93	1.281e-37	2.714e-36	5.46	6.869
PRF1	-2.771	-19.32	1.405e-37	2.966e-36	5.522	8.292
PTPLAD1	-1.382	-18.53	1.541e-37	3.245e-36	4.071	5.454
PDGFC	1.846	15.32	1.653e-37	3.466e-36	4.912	3.065
STMN3	-2.415	-25.87	1.805e-37	3.778e-36	3.471	5.886
NUP93	-1.953	-25.08	2.031e-37	4.233e-36	3.424	5.376
FCER1G	1.343	21.63	2.328e-37	4.841e-36	11.41	10.07
ECHDC3	2.045	17.5	2.347e-37	4.872e-36	6.75	4.705
CACNA1E	1.156	15.78	2.482e-37	5.141e-36	3.574	2.418
FCAR	2.819	23.68	2.536e-37	5.241e-36	7.204	4.386
MRPL9	-1.09	-18.1	2.616e-37	5.397e-36	6.189	7.28
WIPI1	1.395	18.72	2.655e-37	5.466e-36	7.166	5.772
SMARCC1	-1.108	-18.96	2.762e-37	5.673e-36	5.229	6.337
PQLC3	-1.681	-18.31	3.489e-37	7.109e-36	5.306	6.986
HNRNPA3	-1.179	-21.68	3.511e-37	7.139e-36	7.5	8.679
PDIA3	-1.123	-18.27	5.274e-37	1.07e-35	7.305	8.428
XRCC6	-1.24	-20.92	5.653e-37	1.145e-35	6.952	8.192
MPHOSPH10	-1.471	-20.29	6.695e-37	1.353e-35	4.391	5.862
SIRPG	-2.036	-26.74	7.289e-37	1.467e-35	2.984	5.02
CD59	2.123	20.8	7.719e-37	1.55e-35	8.098	5.975
NSUN2	-1.496	-19.68	8.073e-37	1.618e-35	5.193	6.689
SUN1	-1.878	-21.78	8.606e-37	1.722e-35	4.005	5.884
PFKFB3	2.685	21.71	9.385e-37	1.874e-35	9.375	6.69
ARHGEF3	-1.701	-20.3	9.522e-37	1.897e-35	5.276	6.977
NPAT	-1.406	-23.28	1.026e-36	2.041e-35	5.909	7.315
FCER1A	-3.641	-26.08	1.072e-36	2.127e-35	3.087	6.728
ELP3	-1.664	-23.16	1.408e-36	2.778e-35	3.411	5.075
ZAP70	-2.303	-25.99	1.545e-36	3.035e-35	3.542	5.845
CNNM3	-1.293	-21.81	1.553e-36	3.046e-35	4.896	6.19
CIB1	-1.015	-20.48	1.574e-36	3.081e-35	7.128	8.143
GRAMD1A	1.249	16.99	1.777e-36	3.472e-35	7.72	6.47
PDE4DIP	-2.192	-24.2	1.811e-36	3.53e-35	3.319	5.511
PBXIP1	-1.306	-19.74	1.949e-36	3.792e-35	4.379	5.685
BCL9L	-1.703	-26.11	2.133e-36	4.142e-35	4.463	6.166
EXT1	1.097	16.05	2.184e-36	4.234e-35	5.534	4.437
CCNA1	1.544	14.65	2.24e-36	4.334e-35	3.763	2.219
APRT	-1.267	-19.45	2.293e-36	4.419e-35	6.155	7.422
TDP1	-1.471	-18.98	2.293e-36	4.419e-35	3.722	5.194
CD247	-2.287	-25.21	2.59e-36	4.982e-35	4.223	6.51
EIF3A	-1.323	-21.54	3.211e-36	6.128e-35	5.585	6.908
FXYD5	-1.052	-19.63	3.315e-36	6.315e-35	7.568	8.62
BET1L	-1.071	-21.04	3.537e-36	6.713e-35	6.289	7.36

gene_symbol	log2fc	t_stat	p_raw	p_adj	mean_sepsis	mean_healthy
FAM65A	-1.059	-17.54	3.57e-36	6.763e-35	4.948	6.007
PAPD7	-1.428	-22.49	3.98e-36	7.524e-35	4.664	6.091
SCRN1	-1.347	-20.6	4.061e-36	7.664e-35	4.236	5.584
SRPK2	-1.029	-14.92	4.226e-36	7.96e-35	7.829	8.858
CCT8	-1.082	-18.43	4.34e-36	8.159e-35	7.876	8.959
RPL3	-1.235	-19.13	4.486e-36	8.418e-35	9.115	10.35
COG1	-1.165	-21.56	5.299e-36	9.907e-35	5.986	7.151
SRM	-1.266	-16.85	5.31e-36	9.908e-35	4.952	6.218
GTDC1	1.207	17.44	5.551e-36	1.034e-34	5.152	3.944
TXK	-2.58	-22.88	5.875e-36	1.092e-34	4.291	6.871
SBK1	-1.928	-25.88	6.065e-36	1.125e-34	3.739	5.667
MXD3	1.781	18.16	7.549e-36	1.393e-34	7.417	5.636
PCYOX1L	-1.483	-20.87	7.78e-36	1.433e-34	4.166	5.649
CXXC5	-1.78	-19.71	7.851e-36	1.443e-34	5.552	7.331
CCDC115	-1.28	-20.35	8.66e-36	1.586e-34	4.724	6.004
CTCF	-1.357	-20.68	9.48e-36	1.733e-34	5.063	6.42
PJA1	-2.025	-25.6	9.893e-36	1.802e-34	2.64	4.664
XPC	-1.241	-17.35	1.019e-35	1.853e-34	5.286	6.527
EXOSC10	-1.405	-22.7	1.026e-35	1.862e-34	2.941	4.346
SSBP2	-1.609	-23.36	1.058e-35	1.916e-34	3.714	5.323
CIRH1A	-1.502	-20.57	1.12e-35	2.023e-34	3.55	5.052
GANAB	-1.006	-18.49	1.337e-35	2.409e-34	5.86	6.866
SDR39U1	-1.152	-19.52	1.416e-35	2.547e-34	5.455	6.607
VNN1	4.105	24.83	1.516e-35	2.722e-34	8.809	4.704
RAB31	1.171	19.71	1.525e-35	2.728e-34	10.3	9.13
CSE1L	-1.108	-17.14	1.523e-35	2.728e-34	4.654	5.761
FGFBP2	-3.05	-18.58	1.817e-35	3.232e-34	6.068	9.117
LYAR	-1.633	-21.45	1.855e-35	3.294e-34	5.237	6.87
TMEM138	-1.127	-20.82	1.97e-35	3.487e-34	3.061	4.188
GALNT2	1.205	14.72	2.056e-35	3.626e-34	7.491	6.285
CWF19L2	-1.336	-20.67	2.07e-35	3.644e-34	4.132	5.468
C4orf3	1.059	19.32	2.682e-35	4.714e-34	9.751	8.692
SVIP	1.285	15.27	2.956e-35	5.158e-34	5.506	4.221
SNRNP200	-1.182	-20.11	4.112e-35	7.114e-34	6.313	7.496
SLFN5	-2.107	-19.85	4.406e-35	7.61e-34	3.971	6.078
IL27RA	-1.298	-20.38	5.195e-35	8.956e-34	3.775	5.073
MAL	-1.739	-17.17	5.252e-35	9.04e-34	5.374	7.112
CD6	-1.892	-22.49	5.362e-35	9.213e-34	3.353	5.245
ZNF512	-1.071	-20.42	5.704e-35	9.768e-34	5.697	6.769
DUSP6	-1.53	-17.2	6.114e-35	1.045e-33	7.694	9.224
RNF125	-2.239	-24.19	7.381e-35	1.257e-33	3.574	5.813
MAGED1	-1.889	-20.48	7.703e-35	1.31e-33	4.409	6.298
ANKH	-1.529	-15.78	8.552e-35	1.449e-33	4.646	6.175
IL4R	1.471	18.1	1.502e-34	2.532e-33	7.988	6.518
SEC31A	-1.147	-19.72	1.605e-34	2.703e-33	6.337	7.484
ZNF281	1.014	16.53	1.66e-34	2.789e-33	9.311	8.297
JAK3	1.273	18.69	1.794e-34	3.01e-33	9.25	7.977
PURA	-1.695	-19.66	1.846e-34	3.092e-33	5.216	6.911
PCID2	-1.186	-17.4	1.889e-34	3.158e-33	5.023	6.209
EMILIN2	1.256	15.38	1.948e-34	3.252e-33	7.391	6.134
MCTP2	1.695	18.73	2.034e-34	3.39e-33	7.757	6.062
ADAM9	1.997	18.68	2.059e-34	3.426e-33	6.395	4.397
BAZ1A	1.001	16.83	2.212e-34	3.668e-33	9.558	8.557
RBM14	-1.016	-19.54	2.264e-34	3.749e-33	5.545	6.561
ANXA3	3.923	26.91	2.368e-34	3.913e-33	9.724	5.801
PSTPIP2	1.744	18.91	2.508e-34	4.138e-33	7.779	6.035
DAZAP1	-1.137	-19.14	2.521e-34	4.152e-33	5.842	6.978
CHST7	-1.504	-19.26	2.577e-34	4.238e-33	2.868	4.372
HSPD1	-1.356	-15.63	2.665e-34	4.375e-33	5.957	7.312
SIDT1	-1.954	-24.31	2.72e-34	4.458e-33	3.191	5.145
BATF	1.62	18.14	2.804e-34	4.59e-33	6.063	4.443
RSAD1	-1.588	-22.48	3.015e-34	4.926e-33	3.254	4.843
NFX1	-1.391	-20.41	3.061e-34	4.993e-33	4.252	5.643
IRF2BP2	-1.011	-16.09	3.079e-34	5.015e-33	7.98	8.991
GORASP2	-1.229	-20.82	3.187e-34	5.181e-33	4.817	6.046
YTHDC1	-1.02	-17.11	3.457e-34	5.603e-33	6.891	7.911
METTL13	-1.295	-21.33	3.527e-34	5.706e-33	4.521	5.816
AGTPBP1	1.265	15.63	3.558e-34	5.747e-33	7.792	6.527

gene_symbol	log2fc	t_stat	p_raw	p_adj	mean_sepsis	mean_healthy
SLBP	-1.122	-16.48	3.904e-34	6.296e-33	4.93	6.052
EXOSC9	-1.063	-17.64	3.953e-34	6.365e-33	5.081	6.144
SRRM2	-1.09	-15.86	4.053e-34	6.505e-33	7.635	8.725
DIRC2	1.31	16.99	4.074e-34	6.529e-33	5.494	4.183
UBAP2	-1.386	-20.08	4.166e-34	6.665e-33	4.5	5.886
CDC42SE2	-1.254	-17.82	4.312e-34	6.887e-33	8.013	9.268
SFXN1	1.179	14.08	4.381e-34	6.987e-33	4.657	3.478
THOC1	-1.29	-20.13	4.494e-34	7.155e-33	4.466	5.756
THUMPD1	-1.354	-18.83	4.547e-34	7.228e-33	5.768	7.122
FTSJ1	-1.332	-20.52	4.929e-34	7.811e-33	4.316	5.648
OLFM4	4.778	17.53	4.938e-34	7.812e-33	7.52	2.741
BLOC1S1	1.682	20.71	4.996e-34	7.892e-33	6.923	5.241
CNIH4	2.27	23.19	5.029e-34	7.932e-33	9.065	6.795
PROS1	1.287	14.34	5.333e-34	8.397e-33	4.476	3.189
EIF3M	-1.061	-17.35	5.471e-34	8.601e-33	6.805	7.866
TOMM70A	-1.26	-19.46	5.587e-34	8.769e-33	5.19	6.45
WWP1	-1.515	-23.36	5.779e-34	9.056e-33	3.73	5.244
GIMAP8	-1.404	-15.67	6.004e-34	9.38e-33	5.932	7.337
BIRC3	-1.845	-17.98	6.369e-34	9.935e-33	4.818	6.662
INPP4A	-1.107	-16.56	6.659e-34	1.037e-32	6.616	7.723
USP11	-1.846	-21.43	6.749e-34	1.049e-32	4.15	5.996
UHRF2	-1.131	-17.74	6.788e-34	1.054e-32	5.327	6.458
NCOA5	-1.153	-21.83	7.138e-34	1.103e-32	3.988	5.141
HNRNPA0	-1.518	-20.79	7.32e-34	1.13e-32	5.335	6.853
CR1	2.358	20.46	7.852e-34	1.21e-32	8.474	6.116
GCLM	1.636	17.97	8.357e-34	1.284e-32	6.363	4.727
METTL9	1.822	23.45	8.646e-34	1.326e-32	10.54	8.717
PLAC8	1.273	14.3	1.031e-33	1.579e-32	10.82	9.551
MSRA	1.038	14.9	1.05e-33	1.603e-32	6.968	5.93
SMARCA4	-1.146	-19.17	1.076e-33	1.64e-32	5.283	6.428
TOP2B	-1.017	-19.82	1.128e-33	1.716e-32	7.922	8.939
TPST2	1.212	19.16	1.174e-33	1.783e-32	8	6.789
MAML2	-1.613	-17.38	1.207e-33	1.831e-32	5.253	6.866
CIAO1	-1.016	-18.97	1.209e-33	1.831e-32	6.55	7.566
WDR54	-1.592	-20.94	1.228e-33	1.858e-32	3.314	4.906
TECR	-1.248	-17.22	1.328e-33	2.005e-32	4.062	5.31
CST7	2.089	20.6	1.33e-33	2.006e-32	10.73	8.637
SLC20A1	-1.08	-17.69	1.341e-33	2.017e-32	6.934	8.014
CARKD	-1.047	-18.49	1.344e-33	2.018e-32	5.334	6.381
NKTR	-1.114	-18.74	1.398e-33	2.095e-32	7.586	8.701
RBBP7	-1.561	-20.53	1.414e-33	2.117e-32	3.629	5.19
SNX3	1.485	21.85	1.527e-33	2.278e-32	10.91	9.421
PARN	-1.067	-18.28	1.622e-33	2.413e-32	4.854	5.921
PILRA	-1.341	-13.99	1.659e-33	2.465e-32	7.056	8.397
NAP1L1	-1.092	-17.38	1.753e-33	2.6e-32	7.689	8.781
LETMD1	-1.325	-20.34	1.924e-33	2.85e-32	4.908	6.233
IFT20	1.262	18.02	1.974e-33	2.92e-32	7.092	5.83
CHMP7	-1.447	-21.93	2.055e-33	3.03e-32	5.485	6.932
TMEM203	-1.107	-19.43	2.145e-33	3.159e-32	4.543	5.649
CREB3L2	-1.007	-15.43	2.476e-33	3.635e-32	5.973	6.979
GAS7	1.25	15.71	2.563e-33	3.752e-32	8.992	7.742
BCL2A1	3.677	21.97	2.703e-33	3.939e-32	8.244	4.568
PRUNE2	1.275	13.75	2.82e-33	4.092e-32	3.314	2.039
LSG1	-1.158	-20.38	2.908e-33	4.214e-32	4.379	5.536
IPO5	-1.232	-19.86	2.918e-33	4.222e-32	3.593	4.824
TMEM120A	1.362	17.81	2.96e-33	4.276e-32	7.228	5.866
ADCY7	-1.06	-15.47	3.138e-33	4.526e-32	6.683	7.743
OGFOD1	-1.437	-21.45	3.244e-33	4.673e-32	4.383	5.82
ZNF652	-1.102	-16.43	3.582e-33	5.146e-32	6.199	7.301
TCF20	-1.073	-19.71	3.792e-33	5.414e-32	4.603	5.676
FGD4	1.745	19.38	3.796e-33	5.414e-32	6.792	5.047
MAP1LC3B	1.155	20.84	3.838e-33	5.466e-32	8.655	7.5
ROPN1L	1.961	19.77	4.596e-33	6.535e-32	7.993	6.033
SLAMF6	-1.565	-18.95	4.659e-33	6.616e-32	4.698	6.262
EXOC6	1.692	18.38	5.684e-33	8.037e-32	6.704	5.012
FUT7	1.556	17.79	6.443e-33	9.098e-32	6.064	4.508
WBP11	-1.129	-22.16	6.562e-33	9.253e-32	4.632	5.761
SPTLC2	1.054	14.72	6.593e-33	9.283e-32	7.39	6.336

gene_symbol	log2fc	t_stat	p_raw	p_adj	mean_sepsis	mean_healthy
PXK	1.003	15.72	6.74e-33	9.476e-32	7.018	6.015
ILF3	-1.356	-20.58	7.32e-33	1.026e-31	5.105	6.461
SH3YL1	-2.657	-21.84	7.434e-33	1.041e-31	3.398	6.054
QRICH1	-1.031	-23.66	8.105e-33	1.133e-31	5.843	6.875
RNF44	-1.037	-15.67	8.702e-33	1.213e-31	6.733	7.77
PRMT1	-1.292	-16.62	9.653e-33	1.344e-31	4.919	6.212
QSOX1	1.444	20.62	9.828e-33	1.367e-31	6.123	4.679
PRPF4	-1.137	-17.52	1.1e-32	1.528e-31	4.897	6.034
MTX1	1.078	19.16	1.184e-32	1.642e-31	6.495	5.417
NUP43	-1.243	-18.95	1.248e-32	1.729e-31	5.543	6.786
GGH	1.462	15.22	1.33e-32	1.838e-31	4.456	2.995
DHX15	-1.086	-16.82	1.404e-32	1.933e-31	5.972	7.058
CAMK1D	-1.477	-14.6	1.429e-32	1.964e-31	6.445	7.923
CBLB	-1.863	-19.73	1.432e-32	1.967e-31	3.769	5.632
KIAA1430	-1.311	-18.76	1.459e-32	2e-31	5.093	6.404
TGIF2	-1.171	-20.98	1.495e-32	2.047e-31	3.967	5.138
CA4	3.078	20.96	1.503e-32	2.055e-31	7.713	4.635
MERTK	1.001	13.56	1.531e-32	2.088e-31	3.119	2.118
TMED10	-1.155	-19.21	1.598e-32	2.176e-31	7.849	9.004
EMR3	-1.661	-14.18	1.613e-32	2.194e-31	5.147	6.808
SMARCC2	-1.142	-19.67	1.622e-32	2.202e-31	4.81	5.952
PGLYRP1	2.902	20.63	1.673e-32	2.269e-31	6.903	4.001
CDC16	-1.264	-17.52	1.787e-32	2.42e-31	5.874	7.139
KIF3C	1.156	15.28	1.96e-32	2.651e-31	4.072	2.916
ABI2	-1.309	-19.24	2.058e-32	2.78e-31	3.767	5.075
STK16	1.167	18.76	2.093e-32	2.824e-31	5.409	4.242
WFDC1	1.29	13.51	2.168e-32	2.921e-31	3.54	2.25
POM121	-1.107	-17.67	2.331e-32	3.136e-31	5.447	6.554
R3HDM1	-1.197	-17.96	2.334e-32	3.136e-31	4.592	5.789
CPPED1	-1.231	-14.8	2.345e-32	3.146e-31	8.278	9.509
B3GNT5	2.23	16.64	2.62e-32	3.497e-31	5.732	3.502
THEMIS	-2.19	-19.59	3.052e-32	4.059e-31	3.25	5.439
NDUFB3	1.968	21.57	3.084e-32	4.094e-31	8.3	6.332
HCP5	-1.141	-14.37	3.101e-32	4.111e-31	6.636	7.776
SPG7	-1.09	-18.87	3.134e-32	4.149e-31	3.689	4.779
RUNX2	-1.735	-16.87	3.196e-32	4.226e-31	4.415	6.15
CHIT1	1.471	13.45	3.261e-32	4.306e-31	3.681	2.21
ARID1B	-1.372	-18.11	3.285e-32	4.327e-31	4.38	5.751
EAF2	1.021	14.22	3.872e-32	5.066e-31	3.701	2.68
RUFY3	-1.459	-17.65	4.193e-32	5.479e-31	4.189	5.648
HSP90AB1	-1.233	-15.36	4.278e-32	5.582e-31	6.702	7.936
KCNA3	-1.806	-21.26	4.485e-32	5.845e-31	3.294	5.1
ALOX5	1.153	16.62	4.868e-32	6.337e-31	9.666	8.513
LRPPRC	-1.736	-20.86	5.144e-32	6.686e-31	3.183	4.919
FBXL16	-2.049	-24.93	5.669e-32	7.341e-31	2.828	4.878
OSCAR	1.352	19.33	5.874e-32	7.586e-31	6.7	5.349
MEAF6	-1.383	-18.84	5.888e-32	7.594e-31	5.548	6.931
ZAK	1.003	14.53	5.916e-32	7.611e-31	5.002	3.999
FAM53B	-1.38	-19.9	6.119e-32	7.862e-31	5.119	6.498
ITFG2	-1.679	-21.06	6.471e-32	8.303e-31	3.644	5.323
FKBP5	1.92	20.02	6.973e-32	8.924e-31	10.17	8.248
MEF2A	1.387	15.78	7.576e-32	9.684e-31	7.742	6.355
TEP1	-1.021	-14.98	7.792e-32	9.948e-31	5.365	6.386
ITPKB	-1.423	-18.99	7.871e-32	1.004e-30	5.909	7.332
LGALS8	1.018	15.14	8.129e-32	1.034e-30	6.838	5.82
PRPF8	-1.276	-20.05	8.154e-32	1.036e-30	6.118	7.394
PPIE	-1.263	-17.28	8.98e-32	1.138e-30	4.603	5.865
ZNF317	-1.099	-19.1	8.986e-32	1.138e-30	4.177	5.276
RPS5	-1.032	-16.54	9.072e-32	1.148e-30	9.678	10.71
ZBTB4	-1.222	-19.97	1.011e-31	1.275e-30	5.715	6.937
PLIN5	1.325	15.77	1.182e-31	1.485e-30	5.929	4.604
AGAP4	-1.214	-19.5	1.198e-31	1.503e-30	3.344	4.558
FAM102A	-1.229	-20.35	1.314e-31	1.644e-30	4.105	5.334
TNIK	-1.585	-20.38	1.318e-31	1.647e-30	3.074	4.659
KIF1B	2.6	20.34	1.361e-31	1.699e-30	6.838	4.237
FOXJ3	-1.158	-17	1.422e-31	1.772e-30	5.534	6.693
G0S2	1.92	15.48	1.436e-31	1.787e-30	6.425	4.505
KDM1A	-1.063	-18.81	1.486e-31	1.847e-30	4.015	5.079

gene_symbol	log2fc	t_stat	p_raw	p_adj	mean_sepsis	mean_healthy
PARP1	-1.862	-20.27	1.956e-31	2.419e-30	4.22	6.083
WHAMM	-1.126	-20.78	2.412e-31	2.972e-30	4.227	5.353
BCL2	-2.251	-22.39	2.737e-31	3.357e-30	2.973	5.224
NELL2	-3.138	-24.93	2.872e-31	3.509e-30	3.124	6.262
MID1IP1	-1.168	-17.24	2.96e-31	3.612e-30	5.211	6.38
TAPBPL	-1.198	-15.75	3.04e-31	3.705e-30	5.218	6.416
TSPYL1	-1.038	-17.24	3.201e-31	3.887e-30	6.714	7.752
ARMC1	-1.385	-16.69	3.852e-31	4.667e-30	5.552	6.937
SIPA1L2	1.881	17.77	5.442e-31	6.561e-30	7.291	5.41
HACL1	-1.025	-19.32	5.97e-31	7.181e-30	4.654	5.679
ID2	-1.344	-15.24	6.739e-31	8.077e-30	6.344	7.688
PPP3CC	-1.678	-19.95	7.175e-31	8.589e-30	3.348	5.026
NXT1	-1.207	-16.32	7.683e-31	9.185e-30	4.441	5.648
SRPK1	1.693	19.17	8.152e-31	9.735e-30	8.595	6.902
TBX21	-2.218	-19.52	1.015e-30	1.208e-29	4.659	6.877
MCM7	-1.024	-14.36	1.025e-30	1.218e-29	6.363	7.387
PWP2	-1.097	-19.35	1.051e-30	1.248e-29	4.282	5.379
ITGA2B	2.181	15.52	1.103e-30	1.305e-29	5.334	3.153
TCN1	2.998	16.73	1.168e-30	1.38e-29	7.903	4.905
DPYSL2	-1.425	-14.54	1.259e-30	1.486e-29	5.22	6.645
OFD1	-1.406	-17.62	1.273e-30	1.501e-29	4.635	6.041
PHF10	-1.087	-17.26	1.306e-30	1.537e-29	4.574	5.66
STAG3L3	-1.157	-17.62	1.327e-30	1.559e-29	5.059	6.215
STRBP	-1.267	-17.17	1.33e-30	1.56e-29	3.679	4.946
C16orf80	-1.27	-20.21	1.34e-30	1.571e-29	4.156	5.426
LMNB1	1.367	17.63	1.425e-30	1.667e-29	6.731	5.364
TCTN1	-1.335	-22.07	1.484e-30	1.735e-29	2.715	4.05
MOAP1	-1.385	-16.85	1.585e-30	1.85e-29	4.71	6.095
ERCC5	-1.077	-17.4	1.654e-30	1.926e-29	4.804	5.881
ZNF266	-1.633	-18.09	1.667e-30	1.939e-29	5.22	6.852
MICAL1	1.112	15.33	1.678e-30	1.951e-29	7.281	6.17
UBE2F	1.778	20.36	1.781e-30	2.067e-29	7.698	5.92
CD55	1.649	20.37	2.059e-30	2.385e-29	10.54	8.896
PLIN3	1.121	16.56	2.081e-30	2.407e-29	7.922	6.801
TRAP1	-1.095	-18.1	2.19e-30	2.53e-29	3.532	4.627
ACTL6A	-1.036	-16.38	2.312e-30	2.659e-29	4.65	5.686
SAP30	1.409	14.83	2.326e-30	2.672e-29	5.166	3.757
ZNHIT6	-1.76	-21.7	2.584e-30	2.965e-29	2.964	4.724
CTSD	1.094	15.77	2.904e-30	3.324e-29	6.544	5.45
NDE1	-1.002	-14.01	3.03e-30	3.465e-29	7.002	8.004
JAK1	-1.061	-18	3.114e-30	3.553e-29	9.051	10.11
SART3	-1.202	-19.69	3.159e-30	3.6e-29	4.449	5.651
GZMB	-2.291	-15.34	3.437e-30	3.899e-29	5.544	7.835
ATP11B	1.353	14.9	3.498e-30	3.964e-29	8.024	6.671
ITGA4	-1.952	-19.16	3.537e-30	4.004e-29	4.608	6.56
METTL3	-1.054	-17.04	3.579e-30	4.046e-29	5.27	6.324
HMGB2	2.273	19.67	3.73e-30	4.212e-29	8.088	5.815
FCRL3	-2.555	-20.68	4.42e-30	4.975e-29	3.83	6.385
STIM2	-1.097	-16.04	4.644e-30	5.209e-29	3.74	4.838
NUDT9	-1.275	-18.36	4.65e-30	5.209e-29	3.303	4.578
C2orf42	-1.042	-18.8	4.817e-30	5.391e-29	4.015	5.057
ITGAM	1.169	16.38	5.025e-30	5.617e-29	9.858	8.689
FAM43A	-1.719	-22.51	5.093e-30	5.687e-29	3.198	4.916
FLOT1	1.419	20.54	5.103e-30	5.692e-29	10.97	9.552
TMEM110	1.08	13.7	5.11e-30	5.693e-29	3.86	2.78
ZNF91	-1.566	-17.47	5.185e-30	5.771e-29	5.556	7.122
MCCC2	-1.193	-20.13	5.209e-30	5.785e-29	4.675	5.868
S1PR5	-2.417	-20.05	5.31e-30	5.883e-29	3.414	5.83
GPATCH1	-1.184	-19.57	5.706e-30	6.308e-29	3.549	4.733
CRTAP	-1.282	-16.33	6.091e-30	6.692e-29	6.672	7.954
GAR1	-1.248	-18.64	6.088e-30	6.692e-29	3.407	4.654
GTPBP8	-1.117	-16.44	6.093e-30	6.692e-29	4.953	6.07
LY9	-1.837	-20.51	6.328e-30	6.934e-29	3.257	5.093
ECHDC2	-1.373	-19.39	6.721e-30	7.357e-29	4.909	6.281
CYP19A1	1.227	12.93	6.896e-30	7.54e-29	3.254	2.027
REV3L	-1.025	-14.92	7.06e-30	7.711e-29	3.704	4.729
DHRS13	1.714	16.79	7.383e-30	8.055e-29	7.181	5.468
GCN1L1	-1.351	-19.49	7.416e-30	8.082e-29	3.838	5.189

gene_symbol	log2fc	t_stat	p_raw	p_adj	mean_sepsis	mean_healthy
ENTPD1	1.043	16.13	7.518e-30	8.185e-29	7.632	6.589
ETS2	1.542	15.61	7.668e-30	8.338e-29	7.072	5.53
LY75	-1.508	-14.21	7.772e-30	8.443e-29	4.609	6.117
FSTL1	1.183	13.34	8.338e-30	9.047e-29	4.449	3.266
MRPS18B	-1.063	-18.98	9.049e-30	9.798e-29	3.931	4.994
LRG1	1.686	17.88	9.132e-30	9.877e-29	9.741	8.055
MRI1	-1.394	-19.78	9.946e-30	1.073e-28	4.2	5.594
NAT10	-1.243	-21.12	1.01e-29	1.087e-28	2.79	4.032
GPR97	1.686	18.37	1.069e-29	1.15e-28	7.539	5.853
LCN2	4.147	17.66	1.08e-29	1.16e-28	9.896	5.749
FXN	-1.026	-19.37	1.111e-29	1.193e-28	3.441	4.468
BZW2	-1.286	-18.13	1.115e-29	1.196e-28	4.7	5.986
BEX4	-1.305	-16.27	1.232e-29	1.316e-28	4.685	5.99
DHRS3	-2.087	-21.19	1.369e-29	1.455e-28	3.08	5.167
TOMM20	-1.401	-17.48	1.373e-29	1.458e-28	5.107	6.508
ST3GAL5	-1.297	-18.58	1.414e-29	1.5e-28	4.694	5.991
USP24	-1.053	-16.86	1.433e-29	1.518e-28	5.26	6.314
JARID2	-1.065	-15.26	1.689e-29	1.778e-28	6.733	7.798
IER5	-1.241	-15.29	1.903e-29	2e-28	4.547	5.788
SLC7A6	-1.508	-22.41	2.131e-29	2.237e-28	2.903	4.411
ELAVL1	-1.03	-17.78	2.143e-29	2.248e-28	5.633	6.663
GOT2	-1.131	-17.67	2.189e-29	2.293e-28	4.428	5.559
BST1	1.452	16.37	2.208e-29	2.309e-28	8.765	7.314
BLMH	-1.25	-16.9	2.649e-29	2.764e-28	4.308	5.558
FNBP1	-1.02	-15.94	2.658e-29	2.77e-28	5.694	6.714
HLA-DOA	-2.327	-19.51	2.669e-29	2.778e-28	4.311	6.637
KHDRBS1	-1.076	-17.25	2.79e-29	2.901e-28	8.064	9.14
SAMD3	-2.311	-20.68	2.875e-29	2.983e-28	3.263	5.574
ID3	-1.509	-18.78	2.918e-29	3.025e-28	3.71	5.219
GPR160	2.31	18.02	3.094e-29	3.201e-28	7.685	5.375
C9orf89	1.013	15.94	3.36e-29	3.473e-28	7.238	6.225
GPR56	-2.6	-17.58	3.854e-29	3.971e-28	4.19	6.79
CD58	1.423	15.81	3.951e-29	4.066e-28	7.644	6.221
PDIA4	-1.202	-14.09	4.007e-29	4.111e-28	6.104	7.306
NOL11	-1.366	-19.73	4.005e-29	4.111e-28	4.057	5.423
AKAP1	-1.534	-17.78	4.299e-29	4.401e-28	3.694	5.229
DCAF16	-1.212	-19.84	4.337e-29	4.431e-28	3.198	4.411
SELM	-1.051	-19.08	4.448e-29	4.535e-28	2.53	3.581
CAPRIN1	-1.019	-16.3	4.643e-29	4.73e-28	6.528	7.547
NEDD4L	1.161	12.89	4.825e-29	4.91e-28	4.321	3.159
TXNDC17	1.375	17.41	4.976e-29	5.059e-28	5.528	4.154
HEBP2	1.354	18.81	6.31e-29	6.369e-28	7.623	6.268
EIF4A2	-1.4	-15.64	6.394e-29	6.447e-28	6.974	8.374
SLAMF7	-1.501	-13.21	6.672e-29	6.721e-28	5.2	6.701
CYB5B	-1.078	-17.91	7.187e-29	7.233e-28	5.341	6.42
WBSCR22	-1.086	-18.35	8.003e-29	8.029e-28	4.282	5.369
ZNF664	-1.55	-17.44	8.151e-29	8.17e-28	4.069	5.62
S100A12	3.483	23.89	8.31e-29	8.319e-28	12.02	8.537
UBR4	1.064	15.77	8.483e-29	8.477e-28	6.173	5.109
LOC100128751	-1.158	-15.37	8.809e-29	8.786e-28	5.986	7.144
PAG1	1.116	15.36	8.823e-29	8.791e-28	10.31	9.194
TMC8	-1.468	-18.6	8.988e-29	8.946e-28	6.119	7.587
VSIG4	1.209	12.52	9.222e-29	9.17e-28	3.432	2.223
MMP9	3.104	19.03	9.767e-29	9.702e-28	11.39	8.286
LDLRAP1	-1.236	-16.25	1.028e-28	1.02e-27	5.644	6.881
NMT2	-1.976	-23.16	1.046e-28	1.037e-27	2.686	4.662
FAR2	1.595	14.87	1.048e-28	1.038e-27	6.788	5.193
TTYH2	-1.315	-19.03	1.129e-28	1.117e-27	3.417	4.732
RNMT	-1.073	-19.76	1.15e-28	1.137e-27	2.86	3.933
LYRM1	1.052	14.9	1.167e-28	1.152e-27	7.66	6.608
TCERG1	-1.51	-18.82	1.241e-28	1.222e-27	3.576	5.086
MBOAT2	1.973	20.68	1.268e-28	1.245e-27	7.557	5.584
PDS5A	-1.212	-16.41	1.29e-28	1.265e-27	4.778	5.99
PAFAH2	-1.214	-20.84	1.329e-28	1.302e-27	3.375	4.589
OXNAD1	-1.847	-20.8	1.369e-28	1.34e-27	4.4	6.247
POLD2	-1.117	-16.56	1.446e-28	1.413e-27	3.925	5.042
CCT6A	-1.335	-17.35	1.49e-28	1.454e-27	3.405	4.74
SCAMP3	-1.07	-16.8	1.565e-28	1.525e-27	4.351	5.421

gene_symbol	log2fc	t_stat	p_raw	p_adj	mean_sepsis	mean_healthy
C3AR1	1.624	15	1.579e-28	1.536e-27	8.661	7.037
ATM	-1.779	-18.25	1.614e-28	1.569e-27	4.453	6.232
SLC26A6	1.144	15.49	1.628e-28	1.581e-27	5.042	3.898
UBASH3A	-1.959	-22.01	1.695e-28	1.645e-27	2.843	4.801
NUBP1	-1.058	-15.69	1.71e-28	1.657e-27	4.223	5.281
TIGD3	-1.742	-18.28	1.763e-28	1.707e-27	2.984	4.726
ABI3	-1.4	-17.37	1.808e-28	1.748e-27	3.789	5.189
IL18RAP	2.429	18.78	1.865e-28	1.798e-27	9.187	6.759
MTR	-1.583	-20.34	1.877e-28	1.807e-27	3.232	4.815
ZBTB38	-1.256	-16	1.939e-28	1.865e-27	5.08	6.335
RAB22A	-1.035	-17.18	1.987e-28	1.907e-27	4.48	5.515
KCTD6	-1.068	-14.79	1.999e-28	1.917e-27	5.083	6.152
MRE11A	-1.182	-19.32	2.148e-28	2.056e-27	3.667	4.848
MORC2	-1.662	-20.1	2.197e-28	2.101e-27	3.246	4.909
HEATR2	-1.687	-18.96	2.331e-28	2.225e-27	3.836	5.523
PANK4	-1.114	-16.16	2.38e-28	2.27e-27	3.739	4.853
TGS1	-1.115	-18.76	2.424e-28	2.309e-27	4.249	5.364
RHOH	-1.422	-15.25	2.615e-28	2.489e-27	5.127	6.549
F5	1.958	16.15	2.685e-28	2.552e-27	7.923	5.965
PATZ1	-1.335	-21.04	2.69e-28	2.555e-27	2.753	4.087
PAN2	-1.207	-19.51	2.753e-28	2.61e-27	3.089	4.297
CAMK2D	-1.832	-19.08	3.056e-28	2.892e-27	3.521	5.354
CD300LB	-1.147	-14.85	3.155e-28	2.983e-27	3.913	5.06
N4BP2L1	-1.418	-15.61	3.289e-28	3.106e-27	4.503	5.92
IL1R1	2.023	17.09	3.337e-28	3.145e-27	7.049	5.027
FTO	-1.438	-19.46	3.393e-28	3.195e-27	4.642	6.08
FURIN	1.201	17.26	3.655e-28	3.429e-27	6.698	5.497
KLF5	1.198	14.54	3.741e-28	3.506e-27	4.042	2.844
GATA3	-1.934	-21.47	4.419e-28	4.127e-27	4.283	6.218
CORO2A	1.339	14.09	4.622e-28	4.313e-27	5.528	4.189
MS4A1	-2.493	-17.5	5.309e-28	4.944e-27	4.859	7.352
PYHIN1	-2.127	-20.28	5.522e-28	5.138e-27	3.103	5.23
FHIT	-2.38	-19.8	5.825e-28	5.415e-27	3.472	5.852
C12orf65	-1.693	-20.46	5.964e-28	5.538e-27	3.786	5.479
TRANK1	-1.226	-14.69	6.115e-28	5.673e-27	3.175	4.401
ITM2A	-1.613	-15.33	6.53e-28	6.047e-27	6.034	7.647
PLBD1	1.27	16.56	6.662e-28	6.159e-27	10.92	9.654
C1GALT1C1	1.275	13.78	6.663e-28	6.159e-27	4.768	3.493
SH2D2A	-1.958	-17.23	7.538e-28	6.955e-27	3.694	5.652
PCNX	1.198	14.76	7.662e-28	7.063e-27	6.479	5.282
GZMK	-2.468	-15.79	8.09e-28	7.444e-27	4.179	6.647
PSIP1	-1.587	-15.89	8.265e-28	7.591e-27	4.389	5.975
SLC25A36	-1.164	-15.6	9.298e-28	8.524e-27	4.84	6.004
POLRMT	-1.133	-18.24	1.142e-27	1.045e-26	3.71	4.843
SNX5	-1.256	-16.72	1.182e-27	1.081e-26	5.29	6.546
P2RX1	1.445	15.74	1.191e-27	1.088e-26	6.973	5.528
DPP7	-1.191	-16.34	1.227e-27	1.12e-26	4.616	5.807
NSUN6	-1.026	-15.23	1.229e-27	1.121e-26	5.095	6.121
CTNNAL1	1.382	12.78	1.281e-27	1.166e-26	4.045	2.663
ELP2	-1.354	-18.7	1.29e-27	1.174e-26	3.433	4.787
SAR1B	1.811	14.69	1.3e-27	1.182e-26	5.441	3.629
KIAA1715	1.014	13.83	1.388e-27	1.258e-26	5.305	4.29
FLOT2	1.275	16.89	1.425e-27	1.291e-26	8.388	7.113
PPIH	-1.332	-17.61	1.44e-27	1.304e-26	3.836	5.168
MRPS27	-1.028	-19.58	1.65e-27	1.492e-26	2.595	3.623
TRIB1	1.238	14.43	2.006e-27	1.808e-26	7.22	5.982
ZMYND11	-1.766	-17.25	2.05e-27	1.845e-26	4.721	6.487
ISOC1	-1.548	-17.87	2.151e-27	1.934e-26	3.315	4.863
TPM1	1.425	13.97	2.27e-27	2.036e-26	6.083	4.658
ATIC	-1.817	-18.67	2.281e-27	2.044e-26	3.354	5.171
USP32	1.288	18.6	2.529e-27	2.26e-26	7.202	5.914
TAF4	-1.448	-18.44	2.78e-27	2.48e-26	3.985	5.433
ZNF529	-1.668	-19.77	2.961e-27	2.635e-26	2.807	4.475
UXS1	-1.112	-16.04	3.038e-27	2.698e-26	4.776	5.888
QSER1	-1.269	-16.19	3.212e-27	2.85e-26	3.467	4.736
ZNF274	-1.189	-15.34	3.457e-27	3.065e-26	4.245	5.433
ALG2	-1.009	-15.17	3.762e-27	3.32e-26	4.995	6.004
GPR171	-1.458	-13.43	4.002e-27	3.527e-26	5.774	7.231

gene_symbol	log2fc	t_stat	p_raw	p_adj	mean_sepsis	mean_healthy
MDN1	-1.607	-19.22	4.248e-27	3.733e-26	3.728	5.335
IPO7	-1.232	-15.99	4.481e-27	3.931e-26	4.32	5.552
G3BP1	-1.196	-16.3	4.842e-27	4.24e-26	5.37	6.565
CYSLTR1	-1.145	-12.98	5.113e-27	4.466e-26	5.185	6.329
B4GALT5	1.114	15.8	5.123e-27	4.471e-26	9.52	8.406
DYRK2	-2.107	-20.24	5.19e-27	4.525e-26	3.5	5.607
ADRBK2	-1.125	-14.66	5.386e-27	4.678e-26	4.034	5.159
PRKAG2	-1.06	-13.38	5.389e-27	4.678e-26	4.966	6.026
CEP164	-1.01	-15.93	5.413e-27	4.695e-26	4.265	5.276
GTPBP3	-1.39	-20.67	5.565e-27	4.818e-26	2.723	4.113
FLT3LG	-1.616	-19.62	5.899e-27	5.095e-26	2.977	4.593
CDK5RAP1	-1.039	-17.88	6.172e-27	5.316e-26	3.279	4.318
CLSTN1	-1.277	-19.06	6.665e-27	5.731e-26	3.74	5.017
GPATCH2	-1.151	-15.9	6.66e-27	5.731e-26	3.472	4.624
STK39	-1.434	-18.48	6.687e-27	5.745e-26	4.151	5.585
GMFG	1.201	16.61	8.206e-27	7.02e-26	9.88	8.679
RNF114	-1.017	-13.92	8.742e-27	7.466e-26	4.818	5.835
VEGFB	-1.273	-18.3	9.46e-27	8.065e-26	3.914	5.186
TNRC6A	-1.066	-15.48	1.023e-26	8.699e-26	5.146	6.211
S100A8	1.917	22.29	1.304e-26	1.103e-25	12.94	11.02
ZBTB40	-1.484	-19.27	1.309e-26	1.106e-25	3.905	5.389
VPS13C	-1.419	-15.84	1.337e-26	1.129e-25	5.832	7.251
SNRPA1	-1.099	-15.5	1.371e-26	1.157e-25	4.95	6.049
CDC23	-1.237	-17.17	1.4e-26	1.18e-25	3.97	5.207
MFSD6	-1.178	-15.8	1.565e-26	1.314e-25	3.512	4.69
SLC36A1	1.199	15.62	1.583e-26	1.328e-25	5.622	4.423
LY96	2.502	16.81	1.635e-26	1.369e-25	7.464	4.962
RAB20	1.587	14.45	1.766e-26	1.478e-25	7.149	5.562
HDAC4	1.044	14.06	1.773e-26	1.482e-25	6.379	5.334
TMTC1	1.699	13.21	1.888e-26	1.576e-25	5.237	3.537
AIM2	1.933	14.57	2.029e-26	1.689e-25	7.277	5.344
OGG1	-1.037	-15.83	2.362e-26	1.965e-25	3.731	4.768
DIP2A	-1.2	-16.07	2.393e-26	1.989e-25	6.095	7.295
C19orf60	-1.04	-15.39	2.611e-26	2.167e-25	4.017	5.057
FBXO30	1.601	15.25	2.656e-26	2.202e-25	6.046	4.445
DDX28	-1.273	-18.91	2.888e-26	2.387e-25	3.131	4.404
PID1	-2.176	-20.54	3.076e-26	2.538e-25	2.616	4.791
PTCD3	-1.195	-18.13	3.284e-26	2.705e-25	4.411	5.605
GZMH	-2.516	-13.63	3.318e-26	2.73e-25	6.034	8.549
IL11RA	-1.354	-20.63	3.324e-26	2.734e-25	3.056	4.41
LIPA	-1.088	-13.67	3.34e-26	2.744e-25	6.602	7.69
FN3KRP	-1.674	-18.32	3.371e-26	2.767e-25	3.639	5.313
NF2	-1.154	-17.61	3.453e-26	2.828e-25	3.63	4.784
MTA1	-1.086	-18.28	3.48e-26	2.848e-25	2.605	3.691
CLIC3	-2.59	-18.97	3.598e-26	2.939e-25	2.922	5.512
NUP210	-1.016	-16.71	3.785e-26	3.084e-25	3.244	4.26
CHCHD10	-1.289	-15.71	4.006e-26	3.26e-25	4.831	6.12
DHRS9	2.32	14.76	4.022e-26	3.269e-25	8.753	6.433
SGK223	-1.558	-19.4	4.037e-26	3.279e-25	5.124	6.682
HADH	-1.413	-17.08	4.09e-26	3.32e-25	3.397	4.811
SLC22A4	1.786	18.58	4.322e-26	3.495e-25	8.404	6.618
RERE	-1.01	-12.8	4.324e-26	3.495e-25	5.678	6.688
HTATSF1	-1.491	-16.48	4.396e-26	3.551e-25	3.95	5.441
FRY	-1.075	-13.13	4.645e-26	3.746e-25	4.464	5.539
WDFY3	1.561	14.24	4.859e-26	3.915e-25	6.81	5.249
NFE2	1.322	18.13	5.28e-26	4.251e-25	9.592	8.27
KLRD1	-2.217	-16.24	5.848e-26	4.686e-25	4.14	6.357
CD163	1.803	12.86	6.237e-26	4.989e-25	6.754	4.951
GRINA	1.399	18.26	6.652e-26	5.308e-25	7.358	5.959
NTNG2	1.392	14.83	7.176e-26	5.713e-25	6.445	5.053
ZC3H14	-1.1	-16.47	7.194e-26	5.718e-25	3.94	5.04
PLSCR1	1.793	15.87	8.778e-26	6.916e-25	9.499	7.706
PYGL	1.209	14.36	9.466e-26	7.447e-25	9.556	8.348
TNRC6C	-1.331	-16.69	9.672e-26	7.597e-25	4.004	5.335
TSFM	-1.061	-16.35	1.008e-25	7.89e-25	3.847	4.908
BCL6	1.682	17.06	1.166e-25	9.062e-25	11.08	9.4
ZMIZ1	-1.32	-14.01	1.184e-25	9.196e-25	6.083	7.403
PARP14	-1.395	-12.07	1.202e-25	9.334e-25	7.117	8.512

gene_symbol	log2fc	t_stat	p_raw	p_adj	mean_sepsis	mean_healthy
RRS1	-1.388	-17.3	1.216e-25	9.422e-25	3.808	5.196
ST3GAL4	1.148	15.32	1.248e-25	9.659e-25	5.684	4.536
MRPS26	-1.169	-16.87	1.276e-25	9.87e-25	3.217	4.386
EMR1	1.645	13.45	1.375e-25	1.06e-24	7.409	5.764
OCIAD2	-1.392	-16.93	1.417e-25	1.091e-24	2.934	4.326
CACNA2D3	-1.765	-18.59	1.58e-25	1.213e-24	2.907	4.672
ZNF805	-1.132	-15.31	1.607e-25	1.233e-24	3.575	4.707
CHD1L	-1.006	-14.08	1.648e-25	1.263e-24	4.665	5.67
PTER	-1.44	-17.24	1.773e-25	1.354e-24	3.185	4.625
HEATR5B	-1.137	-15.19	1.899e-25	1.447e-24	4.288	5.425
RPAIN	-1.099	-15.62	1.943e-25	1.48e-24	4.776	5.875
CALHM2	-1.333	-15.54	1.977e-25	1.505e-24	4.275	5.608
MAF	-1.951	-16.48	2.036e-25	1.548e-24	3.401	5.352
MAP3K4	-1.553	-18.33	2.068e-25	1.571e-24	3.26	4.813
DAAM2	1.788	12.57	2.074e-25	1.575e-24	4.947	3.159
TGFBR3	-2.13	-17.12	3.182e-25	2.394e-24	3.838	5.969
C1QB	1.285	12.38	3.346e-25	2.51e-24	4.165	2.88
MAD1L1	-1.668	-16.75	3.554e-25	2.661e-24	4.271	5.939
PASK	-2.329	-19.2	3.865e-25	2.893e-24	2.67	4.999
NAA15	-1.308	-15.91	3.905e-25	2.92e-24	3.556	4.863
CST3	-1.324	-13.86	4.06e-25	3.025e-24	7.32	8.644
C6orf48	-1.216	-15.34	4.113e-25	3.062e-24	7.015	8.231
TRIT1	-1.292	-18.21	4.147e-25	3.085e-24	2.763	4.054
PRMT7	-1.193	-17.15	4.206e-25	3.127e-24	4.007	5.2
CARD16	1.799	15.87	4.262e-25	3.166e-24	7.345	5.545
RBL2	-1.011	-14.07	4.635e-25	3.438e-24	7.007	8.017
CD84	-1.064	-13.98	4.96e-25	3.67e-24	4.977	6.041
AGPAT5	-1.541	-16	5.369e-25	3.962e-24	3.561	5.101
SFPQ	-1.062	-14.64	5.409e-25	3.988e-24	5.815	6.877
TSEN54	-1.329	-17.46	5.519e-25	4.063e-24	3.584	4.913
ADRB2	-1.129	-14.06	5.673e-25	4.174e-24	5.512	6.641
VPRBP	-1.108	-15.57	5.934e-25	4.356e-24	4.367	5.476
DNPEP	-1.071	-15.85	6.057e-25	4.443e-24	3.271	4.342
DNAJC9	-1.075	-13.52	6.367e-25	4.663e-24	4.971	6.046
UMPS	-1.14	-15.74	6.373e-25	4.664e-24	3.663	4.803
OSGEPL1	-1.479	-19.34	7.045e-25	5.14e-24	2.235	3.714
CAPRIN2	-1.154	-14.9	7.503e-25	5.46e-24	4.542	5.696
SH2D1A	-1.641	-17.22	7.828e-25	5.687e-24	2.684	4.325
RUVBL1	-1.084	-16.45	7.962e-25	5.781e-24	3.265	4.349
EPHX2	-2.27	-20.69	8.347e-25	6.034e-24	2.223	4.492
FEM1C	1.454	13.39	8.435e-25	6.093e-24	4.98	3.526
HNRNPH3	-1.18	-12.95	9.757e-25	7.028e-24	6.456	7.636
MYL9	1.66	13.45	9.831e-25	7.076e-24	6.283	4.623
MAP4K1	-1.406	-18.22	1.01e-24	7.267e-24	3.002	4.408
C14orf159	-1.157	-12.02	1.053e-24	7.558e-24	4.662	5.82
ZNF146	-1.412	-16.13	1.115e-24	7.994e-24	4.267	5.679
SAMHD1	-1.115	-12.43	1.227e-24	8.775e-24	7.042	8.157
TRIM28	-1.079	-15.99	1.249e-24	8.925e-24	3.468	4.547
SERPINB2	1.303	12.93	1.252e-24	8.941e-24	4.097	2.793
RTN1	-1.694	-16.3	1.335e-24	9.513e-24	3.279	4.973
CAPG	1.399	15.37	1.34e-24	9.543e-24	8.36	6.961
CD7	-1.065	-16.86	1.439e-24	1.022e-23	2.842	3.907
HSPH1	-1.357	-12.28	1.459e-24	1.035e-23	5.374	6.731
C16orf58	-1.009	-16.44	1.61e-24	1.137e-23	3.633	4.643
NSUN3	1.244	13.41	1.618e-24	1.141e-23	5.25	4.006
KLF12	-1.116	-16.24	1.66e-24	1.17e-23	3.511	4.628
RBBP4	-1.082	-13.87	1.667e-24	1.174e-23	6.178	7.26
CCDC84	-1.064	-16.76	1.672e-24	1.177e-23	4.363	5.427
DDX21	-1.102	-14.33	1.711e-24	1.204e-23	6.945	8.047
ZSCAN18	-1.544	-16.46	1.917e-24	1.345e-23	3.206	4.75
MME	-1.714	-11.46	2.083e-24	1.455e-23	7.416	9.13
LAX1	-1.327	-15.1	2.138e-24	1.492e-23	2.969	4.297
TAF3	-1.173	-16.94	2.37e-24	1.648e-23	3.027	4.2
FOXO1	-1.13	-14.77	2.445e-24	1.696e-23	6.155	7.285
ALPK1	1.182	13.37	2.554e-24	1.77e-23	6.142	4.96
SATB1	-1.029	-16.36	2.562e-24	1.775e-23	7.152	8.182
PPRC1	-1.014	-17.14	3.132e-24	2.164e-23	2.683	3.697
LDLR	1.323	12	3.147e-24	2.173e-23	5.084	3.761

gene_symbol	log2fc	t_stat	p_raw	p_adj	mean_sepsis	mean_healthy
LANCL1	-1.344	-16.97	3.228e-24	2.223e-23	4.093	5.437
UBA2	-1.429	-14.09	3.338e-24	2.297e-23	5.231	6.66
ZNF331	-1.074	-14.68	3.424e-24	2.353e-23	3.813	4.887
ZNF689	-1.015	-16.18	3.775e-24	2.589e-23	3.46	4.476
SNRNP40	-1.012	-12.63	3.92e-24	2.686e-23	4.486	5.497
ABCB7	-1.028	-14.15	4.036e-24	2.763e-23	4.126	5.154
APOBEC3F	-1.079	-15.97	4.103e-24	2.807e-23	3.464	4.543
TMEM134	-1.122	-16.32	4.129e-24	2.822e-23	4.113	5.235
RNASE2	2.914	17.39	4.144e-24	2.829e-23	8.678	5.764
CD3E	-1.648	-17.76	4.609e-24	3.139e-23	2.647	4.295
MRPS35	-1.174	-14.2	4.752e-24	3.234e-23	3.685	4.859
CD86	-1.543	-13.45	4.873e-24	3.312e-23	4.18	5.724
FAM98B	-1.396	-14.9	5.101e-24	3.464e-23	3.758	5.154
GLOD4	-1.332	-18	5.201e-24	3.527e-23	3.314	4.646
GSTO1	1.054	14.76	5.214e-24	3.534e-23	9.094	8.04
SLAMF1	-1.143	-16.75	5.452e-24	3.693e-23	3.157	4.3
ERO1L	1.021	13.3	5.58e-24	3.774e-23	5.972	4.952
BLNK	-1.769	-15.07	6.028e-24	4.058e-23	3.351	5.12
ENY2	1.207	13.35	6.095e-24	4.098e-23	6.845	5.638
RBM34	-1.052	-14.01	6.544e-24	4.387e-23	4.732	5.784
MTSS1	-1.677	-14.97	6.62e-24	4.436e-23	4.429	6.106
IRF4	-1.247	-12.83	6.847e-24	4.575e-23	3.523	4.77
MYL6	1.295	17.35	6.863e-24	4.583e-23	10.75	9.453
ARHGAP17	-1.153	-15.4	7.611e-24	5.065e-23	3.596	4.75
RSL1D1	-1.005	-14.61	7.639e-24	5.081e-23	4.609	5.613
TPCN1	-1.086	-15.21	7.75e-24	5.148e-23	4.234	5.32
BCKDHB	-1.275	-17.94	8.315e-24	5.498e-23	3.592	4.867
KREMEN1	2.355	14.79	8.371e-24	5.531e-23	6.321	3.966
PIK3R1	-1.034	-14.97	8.891e-24	5.866e-23	6.279	7.313
MYBL1	-2.434	-16.17	9.819e-24	6.457e-23	3.026	5.459
WDR11	-1.22	-13.84	1.295e-23	8.488e-23	5.334	6.553
FAM105A	1.154	13	1.413e-23	9.217e-23	5.599	4.446
DYNC1H1	-1.094	-14.95	1.429e-23	9.319e-23	4.528	5.622
LY86	-1.27	-11.99	1.441e-23	9.387e-23	5.527	6.797
UBE2L6	-1.163	-11.69	1.458e-23	9.497e-23	8.42	9.583
NAE1	-1.114	-13.21	1.529e-23	9.942e-23	4.88	5.994
MRPS9	-1.031	-15.4	1.539e-23	9.997e-23	3.74	4.77
LTB4R	1.378	14.34	1.602e-23	1.038e-22	7.125	5.746
PCYOX1	-1.062	-15.22	1.665e-23	1.077e-22	3.355	4.417
TMCC1	-1.049	-12.4	1.677e-23	1.084e-22	5.343	6.392
TMEM19	-1.247	-15.71	1.703e-23	1.099e-22	3.249	4.496
UBE2J1	1.416	14.87	1.733e-23	1.118e-22	8.483	7.067
C12orf57	-1.529	-14.19	1.774e-23	1.144e-22	5.561	7.09
NSA2	-1.164	-14.08	1.853e-23	1.194e-22	7.22	8.384
TMEM40	1.355	12.53	1.966e-23	1.263e-22	4.172	2.817
C15orf39	-1.048	-12.47	1.983e-23	1.273e-22	6.604	7.652
CHI3L1	-2.579	-13.29	2.132e-23	1.365e-22	5.67	8.249
DPH3	1.206	14.94	2.418e-23	1.545e-22	5.681	4.476
EIF5B	-1.183	-14.98	2.565e-23	1.633e-22	3.614	4.797
ADARB1	-1.549	-16.59	2.778e-23	1.767e-22	2.997	4.546
AGK	-1.094	-16.31	2.973e-23	1.885e-22	3.309	4.403
SLC22A15	1.17	13.13	3.025e-23	1.915e-22	5.725	4.555
ALPL	2.501	15.57	3.248e-23	2.054e-22	8.317	5.816
PTPLB	-1.295	-13.76	3.322e-23	2.096e-22	4.284	5.579
TNFSF13B	1.031	12.67	3.351e-23	2.113e-22	9.668	8.637
SUCLG2	-1.161	-15.54	3.532e-23	2.223e-22	3.079	4.239
AZI2	1.265	13.5	3.736e-23	2.35e-22	6.956	5.691
CYP1B1	1.827	14.51	3.747e-23	2.355e-22	9.112	7.285
CLEC4E	1.376	14.02	3.875e-23	2.43e-22	9.719	8.343
TWISTNB	-1	-14.01	3.999e-23	2.503e-22	3.801	4.802
CSF1R	-1.342	-14.97	4.2e-23	2.626e-22	3.588	4.929
HKR1	-1.168	-15.57	4.531e-23	2.827e-22	4.055	5.223
IFIT2	-2.089	-11.15	4.917e-23	3.06e-22	6.218	8.307
TXN	1.478	17.43	5.352e-23	3.325e-22	10.26	8.786
PPBP	1.728	13.71	5.424e-23	3.365e-22	10.8	9.073
NUP54	-1.544	-13.84	5.541e-23	3.429e-22	4.383	5.927
HMG20A	-1.153	-16.06	6.356e-23	3.917e-22	2.822	3.975
MTIF2	-1.005	-13.34	6.627e-23	4.081e-22	4.426	5.431

gene_symbol	log2fc	t_stat	p_raw	p_adj	mean_sepsis	mean_healthy
YEATS2	-1.088	-15.2	6.695e-23	4.121e-22	2.953	4.041
SLC2A6	-1.27	-12.99	6.88e-23	4.232e-22	4.782	6.052
CASS4	-1.135	-14.61	7.001e-23	4.301e-22	3.093	4.228
EXOSC6	-1.04	-15.43	7.369e-23	4.519e-22	3.012	4.052
PADI4	1.53	14.61	7.962e-23	4.87e-22	9.177	7.647
DYSF	1.733	15.35	7.981e-23	4.879e-22	9.056	7.323
DDX19A	-1.098	-16.73	8.835e-23	5.378e-22	2.762	3.86
DGKA	-1.087	-14.39	8.979e-23	5.459e-22	4.499	5.586
EXOSC8	-1.231	-13.25	9.549e-23	5.781e-22	4.181	5.412
ENOPH1	-1.33	-13.78	9.663e-23	5.836e-22	4.455	5.784
GJB6	1.021	10.7	1.272e-22	7.588e-22	3.204	2.184
SMYD4	-1.18	-16.28	1.35e-22	8.043e-22	2.819	3.999
SMYD2	-1.205	-14.46	1.379e-22	8.211e-22	3.757	4.962
NOV	-1.968	-17.27	1.43e-22	8.504e-22	2.326	4.294
TTC27	-1.316	-17.2	1.548e-22	9.169e-22	2.495	3.81
GCC2	-1.121	-12.93	1.749e-22	1.032e-21	6.35	7.471
PIK3C2B	-1.054	-16.19	1.833e-22	1.078e-21	2.722	3.776
HIST2H2BE	1.21	12.79	1.884e-22	1.107e-21	6.307	5.097
TCL1A	-2.156	-13.82	1.889e-22	1.11e-21	4.369	6.525
TMEM42	-1.12	-14.93	1.934e-22	1.134e-21	3.591	4.71
PRR5L	-1.437	-15.31	2.051e-22	1.201e-21	3.044	4.481
MPI	-1.167	-16.09	2.208e-22	1.292e-21	3.35	4.518
PLEKHG3	-1.026	-14.18	2.221e-22	1.298e-21	5.907	6.933
RRAGD	1.082	13.26	2.342e-22	1.368e-21	7.347	6.265
CSGALNACT2	1.479	12.01	2.707e-22	1.575e-21	8.203	6.725
CAND1	-1.298	-14.62	2.729e-22	1.586e-21	3.103	4.401
ENOSF1	-1.276	-16.46	2.791e-22	1.621e-21	3.189	4.464
BACH2	-1.82	-16.93	2.952e-22	1.71e-21	2.648	4.468
GLT1D1	1.066	13.21	2.952e-22	1.71e-21	7.23	6.163
KIAA0355	-1.027	-15.99	3.073e-22	1.776e-21	2.858	3.885
TBC1D9	-2.054	-14.76	3.082e-22	1.78e-21	3.231	5.285
PRKX	-1.14	-14.08	3.258e-22	1.88e-21	3.73	4.87
KLF4	-1.336	-13.06	3.773e-22	2.162e-21	4.816	6.151
CD1D	-1.133	-12.4	3.846e-22	2.2e-21	5.946	7.08
LTF	3.77	13.93	4.387e-22	2.5e-21	8.537	4.767
OSBPL3	-1.007	-16.02	4.47e-22	2.543e-21	2.731	3.739
FCGR1B	2.12	14.51	4.648e-22	2.642e-21	5.477	3.357
DDX20	-1.061	-15.12	4.668e-22	2.652e-21	3.106	4.167
ATF7IP2	-1.218	-15.47	4.761e-22	2.702e-21	2.833	4.05
PAICS	-1.043	-13.57	4.938e-22	2.801e-21	2.853	3.896
BRD1	-1.075	-15.31	4.961e-22	2.812e-21	4.908	5.983
C14orf2	2.14	15.42	5.288e-22	2.993e-21	7.241	5.101
GOLGA8A	-2.086	-15.69	5.456e-22	3.081e-21	2.962	5.048
ZNF549	-1.253	-15.98	5.515e-22	3.112e-21	2.528	3.781
SELP	1.412	12.44	5.523e-22	3.115e-21	5.687	4.275
BTBD11	-1.418	-16.72	5.693e-22	3.205e-21	2.601	4.019
UBL5	1.059	14.5	6.027e-22	3.386e-21	9.029	7.97
ZNF608	1.029	10.79	6.988e-22	3.911e-21	3.377	2.348
YLPM1	-1.105	-14.95	7.452e-22	4.156e-21	3.473	4.578
COX7B	2.677	14.74	7.936e-22	4.421e-21	6.477	3.801
FUCA1	-1.336	-12.61	8.447e-22	4.692e-21	4.46	5.795
RBX1	1.575	14.3	8.869e-22	4.919e-21	8.177	6.601
PCSK7	-1.045	-14.84	9.098e-22	5.043e-21	3.845	4.889
MCCC1	-1.107	-15.23	9.481e-22	5.25e-21	3.427	4.534
CXCR5	-1.474	-14.93	9.783e-22	5.411e-21	2.933	4.407
MGAM	1.512	13.17	1.038e-21	5.73e-21	8.677	7.165
EIF4E3	1.042	12.47	1.133e-21	6.234e-21	8.803	7.76
ESF1	-1.242	-14.45	1.174e-21	6.449e-21	3.172	4.414
PLCG1	-1.175	-16.4	1.207e-21	6.619e-21	2.596	3.771
NOP58	-1.155	-12.34	1.314e-21	7.193e-21	5.229	6.384
THEM4	-1.582	-16.82	1.389e-21	7.603e-21	2.523	4.105
TIGIT	-1.646	-16.65	1.492e-21	8.15e-21	2.499	4.144
CCR6	-1.334	-16.73	1.548e-21	8.442e-21	2.566	3.9
NBPF8	-1.046	-12.32	1.769e-21	9.623e-21	6.887	7.934
KLRF1	-2.502	-14.05	1.799e-21	9.781e-21	3.661	6.163
EBAG9	-1.153	-13.67	1.958e-21	1.063e-20	3.749	4.902
TRRAP	-1.078	-14.93	2.075e-21	1.124e-20	3.05	4.128
TMCO3	1.088	11.62	2.094e-21	1.134e-20	6.465	5.377

gene_symbol	log2fc	t_stat	p_raw	p_adj	mean_sepsis	mean_healthy
SMARCE1	-1.108	-13.64	2.095e-21	1.134e-20	5.547	6.655
YOD1	2.531	14.09	2.315e-21	1.248e-20	7.783	5.251
CBS	1.65	12.41	2.32e-21	1.25e-20	4.61	2.96
PPP4R2	1.619	12.57	2.479e-21	1.335e-20	7.979	6.359
BCLAF1	-1.026	-11.99	2.54e-21	1.366e-20	6.785	7.811
TTC9	-1.623	-15.1	2.658e-21	1.426e-20	3.375	4.998
KDM3A	-1.079	-12.09	2.675e-21	1.432e-20	6.094	7.173
RBM26	-1.158	-14.78	2.867e-21	1.53e-20	3.061	4.219
CRIPT	1.068	13.2	2.901e-21	1.547e-20	5.292	4.224
XYLT1	-1.395	-14.82	3.072e-21	1.634e-20	2.983	4.378
HSDL2	1.136	12.09	3.071e-21	1.634e-20	6.524	5.388
PDCD10	1.924	14.08	3.894e-21	2.055e-20	7.715	5.791
METAP2	-1.026	-12.04	4.198e-21	2.21e-20	4.506	5.532
TPD52	-1.458	-12.87	4.196e-21	2.21e-20	3.249	4.707
HIRA	-1.047	-11.67	4.257e-21	2.24e-20	4.636	5.683
TBC1D4	-1.947	-16.47	4.607e-21	2.414e-20	2.558	4.505
HLA-DQA1	-2.899	-14.36	4.934e-21	2.583e-20	5.691	8.59
CCR5	-1.289	-14.53	4.946e-21	2.588e-20	2.907	4.196
TMEM56	1.513	12.33	4.95e-21	2.588e-20	5.519	4.006
FYCO1	-1.002	-14.18	5.952e-21	3.098e-20	3.441	4.443
RDH13	-1.176	-15.06	6.317e-21	3.281e-20	3.149	4.326
RELL1	-1.121	-11.44	6.336e-21	3.289e-20	7.283	8.404
TOP2A	1.267	11.04	6.722e-21	3.484e-20	4.327	3.06
HEG1	-1.247	-15.02	7.539e-21	3.893e-20	2.789	4.035
ANXA1	1.438	13.63	7.716e-21	3.979e-20	9.928	8.49
JAK2	1.376	12.6	8.645e-21	4.442e-20	5.655	4.279
ALDH1A1	-1.727	-15.14	8.881e-21	4.56e-20	3.156	4.882
FANCF	-1.119	-15	9.442e-21	4.841e-20	2.818	3.937
HIST1H2AE	1.167	12.01	1.057e-20	5.38e-20	5.099	3.932
RAD50	-1.13	-14.47	1.266e-20	6.397e-20	3.127	4.256
KLHL2	1.814	12.01	1.267e-20	6.402e-20	6.93	5.115
IL1RN	1.273	12.29	1.286e-20	6.488e-20	8.852	7.579
OTUD4	-1.034	-13.78	1.378e-20	6.933e-20	4.028	5.062
PPP1R12B	-1.152	-12.26	1.43e-20	7.184e-20	3.963	5.115
PPM1K	-1.334	-13.43	1.488e-20	7.469e-20	2.66	3.994
ZFP64	-1.249	-14.98	1.534e-20	7.694e-20	3.092	4.341
TNFAIP6	2.079	13.96	1.578e-20	7.906e-20	9.226	7.147
LIMA1	-1.1	-14.54	1.649e-20	8.258e-20	2.489	3.588
EPB41L2	-1.017	-14.84	1.813e-20	9.055e-20	2.379	3.395
CCPG1	1.216	11.78	1.827e-20	9.114e-20	7.896	6.68
LIME1	-1.063	-13.89	1.833e-20	9.135e-20	3.782	4.846
HIST1H2BD	1.697	13.06	1.879e-20	9.36e-20	7.307	5.61
FNDC3B	1.123	12.32	1.884e-20	9.382e-20	7.757	6.634
PRKCA	-1.152	-12.27	1.941e-20	9.655e-20	4.79	5.942
WDR59	-1.221	-14.94	1.971e-20	9.798e-20	3.121	4.342
MS4A7	-1.587	-11.61	2.015e-20	1e-19	5.235	6.822
CDKN1C	-2.446	-13.94	2.061e-20	1.022e-19	3.856	6.302
POP5	-1.115	-13.49	2.372e-20	1.171e-19	3.429	4.544
QDPR	-1.102	-14.55	2.536e-20	1.25e-19	3.172	4.274
PDPR	-1.32	-13.53	2.678e-20	1.318e-19	3.95	5.27
PCMTD2	1.357	12.01	2.683e-20	1.32e-19	7.099	5.742
POLB	-1.146	-10.92	2.819e-20	1.385e-19	4.437	5.582
SLC37A2	-1.034	-13.37	2.928e-20	1.436e-19	3.758	4.792
TIFA	1.346	11.28	3.566e-20	1.736e-19	6.337	4.991
TULP4	-1.18	-14.25	3.733e-20	1.814e-19	2.799	3.979
GBP5	-1.852	-10.81	3.934e-20	1.909e-19	6.458	8.31
CCNB1IP1	-1.107	-13.41	3.95e-20	1.916e-19	3.568	4.676
RHOU	1.437	11.22	3.997e-20	1.937e-19	5.212	3.775
ISCA1	1.7	11.57	4.371e-20	2.115e-19	6.699	5
CLCC1	-1.07	-14.65	4.442e-20	2.149e-19	3.407	4.477
ACSL4	1.334	11.67	4.508e-20	2.177e-19	7.276	5.942
ITGB3	1.229	11.06	4.54e-20	2.191e-19	4.735	3.506
LOC100131541	-1.871	-13.26	4.674e-20	2.251e-19	3.387	5.258
GPR114	-1.184	-14.21	4.932e-20	2.373e-19	2.731	3.915
USPL1	-1.166	-13.35	5.097e-20	2.45e-19	3.413	4.58
C19orf12	-1.168	-12.42	5.232e-20	2.514e-19	3.667	4.835
CEP78	-1.42	-14.55	5.478e-20	2.628e-19	2.922	4.342
HPSE	1.332	11.46	5.519e-20	2.646e-19	6.303	4.971

gene_symbol	log2fc	t_stat	p_raw	p_adj	mean_sepsis	mean_healthy
GAL3ST4	-1.436	-15.53	6.25e-20	2.987e-19	2.338	3.774
TMEM165	1.186	11.77	7.396e-20	3.522e-19	7.626	6.44
TTC37	-1.051	-13.91	7.547e-20	3.591e-19	4.108	5.159
LRRN3	-3.2	-15.94	7.637e-20	3.632e-19	2.601	5.802
MIS12	-1.001	-12.49	7.788e-20	3.7e-19	4.07	5.071
PRKACB	-1.637	-12.36	8.023e-20	3.809e-19	6.368	8.004
MATK	-1.719	-14.54	8.764e-20	4.139e-19	2.816	4.534
NUP107	-1.159	-13.05	9.105e-20	4.296e-19	3.953	5.112
GK	1.417	11.73	9.187e-20	4.332e-19	7.288	5.871
GM2A	1.153	11.82	9.642e-20	4.538e-19	5.72	4.567
ATMIN	-1.103	-12.71	9.773e-20	4.598e-19	4.051	5.154
SH2D1B	-1.8	-14.65	1.023e-19	4.807e-19	3.092	4.891
TIMP1	1.092	12.22	1.139e-19	5.338e-19	9.088	7.996
ANKRD55	1.385	11.1	1.229e-19	5.751e-19	5.926	4.541
PDE4D	1.331	10.57	1.271e-19	5.925e-19	4.723	3.392
GPRASP1	-1.315	-14.32	1.289e-19	6.01e-19	2.576	3.891
GBP4	-1.33	-11.21	1.322e-19	6.15e-19	3.374	4.704
NFIL3	1.028	11.07	1.367e-19	6.352e-19	8.156	7.128
FOLR3	2.596	12.96	1.448e-19	6.715e-19	7.331	4.735
RALGAPA1	-1.206	-13.26	1.453e-19	6.734e-19	3.257	4.463
IKBKAP	-1.127	-13.98	1.629e-19	7.53e-19	3.233	4.36
NQO2	2.018	13.46	1.645e-19	7.598e-19	9.086	7.068
ZNF827	-1.079	-14.22	1.653e-19	7.627e-19	2.382	3.461
DOCK9	-1.282	-14.37	1.918e-19	8.786e-19	3.086	4.368
PLXNB2	-1.038	-11.7	1.927e-19	8.819e-19	3.993	5.032
CHPT1	1.382	13.2	2.011e-19	9.195e-19	9.981	8.599
ARRDC3	-1.424	-11.55	2.053e-19	9.385e-19	5.38	6.805
PTGDS	-2.03	-13.73	2.071e-19	9.458e-19	2.962	4.992
TFDP1	1.248	11.43	2.091e-19	9.544e-19	7.496	6.249
CD28	-1.323	-14.8	2.1e-19	9.581e-19	2.512	3.835
HIST1H1C	1.247	11.23	2.243e-19	1.021e-18	7.279	6.032
QRSL1	-1.098	-13.45	2.659e-19	1.203e-18	3.269	4.367
ATP8B4	1.508	11.04	3.17e-19	1.429e-18	5.659	4.152
CD160	-2.191	-14.83	3.252e-19	1.464e-18	2.87	5.061
TMEM181	-1.192	-12.25	3.32e-19	1.494e-18	4.794	5.987
TGM2	1.305	10.37	3.38e-19	1.52e-18	4.645	3.34
RNF144A	-1.194	-13.58	3.412e-19	1.533e-18	3.069	4.263
TSHZ3	1.108	10.62	3.981e-19	1.782e-18	4.581	3.473
AUTS2	-1.774	-13.83	4.247e-19	1.899e-18	2.736	4.511
TPST1	1.43	11.56	4.72e-19	2.098e-18	4.999	3.569
TRAT1	-1.611	-12.7	4.752e-19	2.11e-18	2.97	4.58
SIK2	-1.059	-13.79	5.291e-19	2.342e-18	3.195	4.255
NCALD	-1.134	-14.26	5.336e-19	2.361e-18	2.598	3.732
GLRX	1.457	12.71	5.372e-19	2.374e-18	8.626	7.169
BANK1	-1.711	-12.8	5.379e-19	2.376e-18	3.28	4.991
AKAP11	-1.355	-12.27	5.804e-19	2.562e-18	4.415	5.77
RNF157	-1.109	-13.38	5.915e-19	2.608e-18	3.052	4.161
MANSC1	1.038	11.19	6.121e-19	2.695e-18	7.521	6.484
TRERF1	-1.16	-12.76	6.167e-19	2.715e-18	3.468	4.628
CBLL1	-1.205	-12.45	6.291e-19	2.765e-18	3.995	5.2
IFIH1	-1.447	-9.941	6.295e-19	2.766e-18	4.905	6.352
SP140L	-1.009	-12.53	6.402e-19	2.809e-18	4.773	5.782
VMA21	-1.1	-12.08	6.493e-19	2.848e-18	5.422	6.522
TAP2	-1.304	-10.72	6.846e-19	3.002e-18	6.38	7.684
DIS3L	-1.103	-13.41	7.389e-19	3.23e-18	2.744	3.847
ZNF559	-1.32	-13.49	7.597e-19	3.313e-18	3.652	4.972
POLR1B	-1.341	-14.3	8.681e-19	3.76e-18	2.882	4.223
CMTM5	1.444	11.13	9.085e-19	3.926e-18	6.347	4.904
DCUN1D1	1.238	11.59	9.516e-19	4.111e-18	5.813	4.575
UBE2Q2	-1.477	-12.52	1.025e-18	4.417e-18	3.863	5.34
IER3	1.338	11.93	1.109e-18	4.775e-18	6.955	5.617
CEACAM4	1.174	10.81	1.171e-18	5.029e-18	7.467	6.293
KIF21A	-1.011	-13.27	1.206e-18	5.165e-18	2.486	3.496
ZDHHC2	1.472	11.89	1.272e-18	5.441e-18	7.48	6.008
CLEC2D	-1.02	-12.4	1.305e-18	5.574e-18	4.025	5.045
ITGA6	-1.45	-11.88	1.337e-18	5.703e-18	4.752	6.201
CKLF	1.193	12.51	1.602e-18	6.807e-18	9.513	8.319
MTX3	-1.282	-13.06	1.617e-18	6.867e-18	2.951	4.233

gene_symbol	log2fc	t_stat	p_raw	p_adj	mean_sepsis	mean_healthy
ZCCHC2	-1.252	-10.1	1.665e-18	7.067e-18	5.403	6.656
WDR75	-1.138	-13.45	1.737e-18	7.365e-18	3.086	4.223
RCAN3	-1.108	-14.02	1.74e-18	7.374e-18	2.667	3.775
CYP4V2	-1.207	-13.58	1.938e-18	8.174e-18	2.58	3.787
PDE7A	-1.043	-11.67	2.076e-18	8.739e-18	5.929	6.972
LRIG1	-1.467	-13.73	2.11e-18	8.868e-18	2.849	4.316
JMY	-1.06	-13	2.587e-18	1.077e-17	2.833	3.892
PDCD7	-1.137	-13.19	2.599e-18	1.081e-17	2.614	3.751
CTDSPL2	-1.149	-12.16	2.776e-18	1.153e-17	4.156	5.305
PRSS23	-1.978	-13.94	2.842e-18	1.178e-17	2.817	4.795
SLC39A6	-1.059	-11.73	2.903e-18	1.203e-17	3.64	4.698
GNPTAB	-1.056	-12.4	3.047e-18	1.261e-17	4.025	5.081
HAT1	1.167	10.96	3.205e-18	1.325e-17	5.347	4.18
SEMA4A	1.199	11.63	3.541e-18	1.458e-17	7.744	6.546
CEACAM8	3.233	11.57	3.561e-18	1.465e-17	6.826	3.593
CASP5	1.354	10.19	3.581e-18	1.473e-17	4.469	3.115
CA1	3.137	11.95	3.6e-18	1.48e-17	9.612	6.475
C2orf88	1.815	11.41	3.647e-18	1.498e-17	6.992	5.177
SERPINB9	-1.082	-10.84	4.165e-18	1.706e-17	6.469	7.55
MTMR11	-1.178	-11.07	4.67e-18	1.904e-17	4.16	5.338
LIMK2	1.205	11.67	4.939e-18	2.01e-17	7.734	6.529
FAM129C	-1.456	-12.41	5.575e-18	2.261e-17	3.095	4.551
SGMS2	1.291	11.02	5.965e-18	2.407e-17	4.543	3.252
SLC25A32	-1.048	-12.31	6.752e-18	2.715e-17	2.589	3.637
GFM1	-1.06	-12.81	7.055e-18	2.829e-17	2.674	3.734
SLC11A1	1.016	10.55	8.269e-18	3.299e-17	7.75	6.734
ABCG1	-1.345	-10.6	8.382e-18	3.343e-17	4.609	5.953
FCGR1A	2.279	12.43	8.941e-18	3.553e-17	9.134	6.856
PRNP	-1.164	-10.22	9.107e-18	3.616e-17	6.386	7.55
GNG11	1.103	10.35	9.358e-18	3.712e-17	7.759	6.656
CEP68	-1.195	-11.41	9.897e-18	3.908e-17	4.52	5.715
ABHD13	1.087	10.95	9.971e-18	3.936e-17	5.042	3.955
SIGLEC5	1.412	11.82	1.069e-17	4.204e-17	8.57	7.158
SPTBN1	-1.216	-11.19	1.3e-17	5.075e-17	6.372	7.588
CTDSPL	1.14	10.15	1.335e-17	5.206e-17	4.476	3.336
TSEN15	-1.054	-12.36	1.425e-17	5.544e-17	3.029	4.083
RRM1	-1.048	-11.16	1.517e-17	5.888e-17	3.733	4.781
GZMA	-1.475	-10.3	1.563e-17	6.065e-17	6.17	7.645
SLCO4C1	1.367	10.58	1.58e-17	6.125e-17	5.561	4.194
ANK3	-1.169	-12.43	1.695e-17	6.553e-17	3.041	4.21
SNRK	-1	-10.32	1.876e-17	7.218e-17	8.101	9.101
S1PR1	-1.266	-9.649	1.95e-17	7.494e-17	5.427	6.693
SMARCD3	1.035	10.19	2.043e-17	7.84e-17	6.261	5.227
XK	2.632	11.42	2.158e-17	8.27e-17	6.916	4.284
ATP10D	-1.049	-11.01	2.208e-17	8.459e-17	3.749	4.799
NDUFB1	1.256	11.7	2.291e-17	8.765e-17	7.047	5.79
GPR68	-1.006	-12.84	2.426e-17	9.259e-17	3.371	4.377
PRRC1	-1.048	-10.82	2.996e-17	1.136e-16	3.901	4.949
LPCAT2	1.164	10.43	3.065e-17	1.161e-16	7.091	5.927
CLU	1.591	11.55	3.729e-17	1.406e-16	8.483	6.892
IKZF2	-1.511	-13.35	3.893e-17	1.467e-16	2.194	3.705
SLC40A1	1.086	10.45	3.914e-17	1.474e-16	7.361	6.276
TREML1	1.634	10.43	4.212e-17	1.583e-16	6.739	5.105
FKBP1B	1.082	10.13	4.325e-17	1.625e-16	4.855	3.773
KLF10	-1.251	-10.53	4.835e-17	1.809e-16	4.506	5.757
BCAT1	1.092	9.899	4.972e-17	1.858e-16	4.79	3.698
ANKRD36	-1.199	-12.29	5.152e-17	1.924e-16	3.828	5.026
TSPAN3	-1.019	-12.04	5.292e-17	1.973e-16	2.948	3.967
ASGR2	1.321	10.23	5.336e-17	1.988e-16	5.538	4.216
TC2N	-1.13	-12.72	5.393e-17	2.008e-16	2.536	3.666
SIAH2	1.43	10.86	5.412e-17	2.014e-16	9.599	8.169
IKZF3	-1.25	-12.13	5.985e-17	2.221e-16	2.601	3.851
AMIGO2	-1.17	-11.4	6.01e-17	2.229e-16	3.003	4.173
MRFAP1L1	-1.139	-11.38	6.14e-17	2.275e-16	4.311	5.45
MEX3C	-1.366	-11.76	8.403e-17	3.079e-16	3.041	4.407
ZZZ3	-1.01	-12.16	9.004e-17	3.285e-16	2.826	3.836
FAM160B1	1.105	10.66	9.706e-17	3.534e-16	7.647	6.542
CPD	1.095	10.06	1.022e-16	3.719e-16	7.394	6.299

gene_symbol	log2fc	t_stat	p_raw	p_adj	mean_sepsis	mean_healthy
STYX	-1.052	-10.59	1.092e-16	3.964e-16	5.072	6.124
CRTAM	-1.442	-12.45	1.159e-16	4.201e-16	2.453	3.896
HOPX	-1.318	-10.52	1.247e-16	4.51e-16	4.066	5.384
ANKRD9	1.399	10.44	1.285e-16	4.641e-16	5.701	4.302
AFF3	-1.583	-11.88	1.35e-16	4.868e-16	2.333	3.916
DUSP5	-1.042	-10.78	1.395e-16	5.023e-16	2.81	3.852
GATA2	-2.603	-12.75	1.42e-16	5.105e-16	2.735	5.338
DAPK1	-1.058	-10.21	1.484e-16	5.329e-16	5.159	6.217
CRY1	-1.206	-12.17	1.492e-16	5.357e-16	2.492	3.698
SLC39A10	-1.134	-11.11	1.507e-16	5.407e-16	3.491	4.625
ADM	1.596	11.69	1.54e-16	5.513e-16	9.089	7.493
RNASE3	2.085	10.82	2.051e-16	7.273e-16	6.09	4.005
HIP1	1.648	11.41	2.166e-16	7.669e-16	6.799	5.152
RNF182	1.241	8.811	2.166e-16	7.669e-16	3.392	2.151
OAS3	-2.019	-9.181	2.228e-16	7.878e-16	5.083	7.102
CD79A	-1.144	-11.2	2.407e-16	8.485e-16	3.272	4.416
MGMT	-1.065	-11.58	2.419e-16	8.526e-16	3.354	4.419
ELANE	1.729	9.602	2.782e-16	9.745e-16	4.998	3.269
ALDH18A1	-1.072	-11.6	2.993e-16	1.046e-15	3.343	4.415
CD24	1.917	9.756	3.177e-16	1.107e-15	7.959	6.042
TMCC2	1.15	9.255	3.185e-16	1.109e-15	5.664	4.514
E2F5	-1.036	-10.93	3.212e-16	1.118e-15	2.716	3.752
SYTL2	-1.464	-12.24	3.342e-16	1.162e-15	2.588	4.052
SLPI	1.97	10.92	3.91e-16	1.355e-15	7.672	5.702
CEACAM21	1.008	10.43	3.936e-16	1.363e-15	6.106	5.098
CHMP5	1.449	10.55	4.355e-16	1.505e-15	7.153	5.704
ZBTB1	-1.245	-11.23	4.424e-16	1.526e-15	3.182	4.426
AHSP	1.949	10.23	5.163e-16	1.77e-15	8.736	6.786
UQCR11	1.16	11.28	7.58e-16	2.571e-15	9.01	7.849
ZFP62	-1.028	-11.36	7.908e-16	2.678e-15	2.594	3.622
BNIP2	1.062	9.599	7.928e-16	2.682e-15	6.044	4.981
PLCXD2	-1.132	-11.52	8.107e-16	2.74e-15	2.523	3.655
OAS2	-1.33	-9.094	8.499e-16	2.869e-15	4.458	5.788
KBTBD7	1.076	9.474	9.523e-16	3.205e-15	6.55	5.474
THBS1	1.325	9.926	9.963e-16	3.346e-15	5.653	4.328
PTGS1	1.288	9.806	1.038e-15	3.482e-15	6.449	5.161
WARS	-1.002	-9.433	1.086e-15	3.64e-15	7.238	8.24
CLEC2B	1.831	10.15	1.223e-15	4.085e-15	6.793	4.962
NDUFA4	1.937	11.32	1.316e-15	4.375e-15	8.046	6.109
ZMYND8	-1.038	-9.821	1.527e-15	5.062e-15	5.188	6.226
SUB1	1.294	10.17	1.602e-15	5.298e-15	7.836	6.542
PTGDR	-1.033	-11.86	1.693e-15	5.592e-15	2.117	3.15
FAM174A	-1.011	-10.36	1.709e-15	5.639e-15	3.702	4.712
MPO	1.234	8.924	1.846e-15	6.077e-15	5.306	4.072
ASF1A	-1.199	-10.98	2.006e-15	6.589e-15	3.726	4.926
BRIX1	-1.04	-10.5	2.06e-15	6.756e-15	3.398	4.438
CREG1	1.011	10.06	2.116e-15	6.923e-15	8.544	7.533
CTSW	-1.009	-10.78	2.171e-15	7.094e-15	3.441	4.45
PTCH1	-1.105	-11.7	3.215e-15	1.035e-14	2.316	3.421
ABCC13	1.527	9.312	3.214e-15	1.035e-14	4.532	3.005
VPREB3	-1.467	-10.6	3.59e-15	1.151e-14	3.023	4.49
BPGM	2.116	9.744	4.085e-15	1.302e-14	6.177	4.06
CLIP4	-1.198	-10.38	4.261e-15	1.356e-14	3.388	4.587
CD8B	-1.158	-10.64	4.949e-15	1.57e-14	2.955	4.113
P2RX5	-1.196	-9.474	5.169e-15	1.638e-14	4.505	5.701
ABCE1	-1.018	-10.64	5.213e-15	1.651e-14	4.089	5.107
CLEC10A	-1.531	-11	5.265e-15	1.666e-14	2.995	4.526
SWAP70	-1.002	-9.785	5.454e-15	1.723e-14	4.138	5.14
RRAS2	-1.379	-10.6	5.845e-15	1.841e-14	3.047	4.426
GBP1	-1.441	-8.559	6.241e-15	1.963e-14	7.012	8.453
BLK	-1.227	-10.26	8.172e-15	2.55e-14	2.933	4.16
CISD2	1.341	8.926	9.069e-15	2.82e-14	6.166	4.825
XRRA1	-1.534	-10.62	9.691e-15	3.01e-14	3.903	5.437
SNRPN	-1.065	-10.52	1.016e-14	3.154e-14	2.69	3.756
SLC39A8	1.042	8.625	1.133e-14	3.513e-14	4.78	3.738
MGST1	1.062	9.644	1.426e-14	4.393e-14	7.413	6.35
TGFA	1.162	9.394	1.551e-14	4.765e-14	5.243	4.081
CEACAM6	1.827	9.054	1.554e-14	4.772e-14	5.323	3.496

gene_symbol	log2fc	t_stat	p_raw	p_adj	mean_sepsis	mean_healthy
SLC4A7	-1.089	-10.51	1.558e-14	4.781e-14	2.885	3.975
RNASE6	-1.091	-8.97	1.664e-14	5.095e-14	6.239	7.33
HOXB2	-1.268	-10.92	1.689e-14	5.17e-14	2.651	3.92
SLC25A40	1.168	8.763	2.29e-14	6.95e-14	6.657	5.489
PROK2	1.05	9.328	2.732e-14	8.256e-14	10.76	9.712
LGALS1	1.006	9.914	2.884e-14	8.691e-14	9.751	8.745
S100P	2.037	10.5	2.897e-14	8.724e-14	11.3	9.262
KIAA0101	1.173	8.742	3.114e-14	9.364e-14	4.02	2.847
ZNF354A	1.1	8.763	3.124e-14	9.391e-14	4.872	3.772
ERAP1	-1.214	-9.885	3.831e-14	1.144e-13	5.369	6.584
PRSS33	-1.075	-10.59	4.562e-14	1.355e-13	2.252	3.327
MTHFD2	1.176	9.229	4.574e-14	1.358e-13	6.64	5.465
FAR1	1.034	9.068	4.72e-14	1.4e-13	7.154	6.12
KIAA1324	-1.511	-9.623	4.918e-14	1.458e-13	3.231	4.742
FAM118A	-1.048	-10.32	4.958e-14	1.469e-13	3.262	4.311
VSTM1	1.158	9.011	6.147e-14	1.81e-13	5.779	4.621
COPS2	1.05	9.126	7.204e-14	2.105e-13	4.417	3.367
GRPEL2	-1.061	-10.17	7.44e-14	2.17e-13	3.408	4.47
ALCAM	1.053	8.472	7.573e-14	2.207e-13	6.01	4.957
NUDT4	2.14	9.596	7.662e-14	2.233e-13	7.188	5.047
IGHM	-1.455	-8.666	7.668e-14	2.234e-13	6.2	7.655
CPEB4	1.38	9.954	8.488e-14	2.466e-13	7.626	6.246
ORM1	2.165	9.352	8.874e-14	2.574e-13	6.707	4.542
RNF11	1.177	8.917	9.41e-14	2.725e-13	8.102	6.925
CD40	-1.031	-9.496	1.265e-13	3.637e-13	3.1	4.131
TSTA3	1.144	8.562	1.602e-13	4.582e-13	7.057	5.913
FAM3C	-1.134	-9.68	2.086e-13	5.929e-13	2.62	3.754
PMS1	-1.077	-9.784	2.152e-13	6.11e-13	2.776	3.853
GLS	-1.355	-9.043	2.269e-13	6.434e-13	4.927	6.282
CPA3	-1.588	-10.16	2.274e-13	6.447e-13	2.266	3.855
GALNT3	1.062	8.895	2.299e-13	6.515e-13	5.784	4.722
TSPAN2	1.004	8.61	2.738e-13	7.719e-13	7.079	6.075
CAPZA2	1.091	9.157	3.412e-13	9.551e-13	7.969	6.878
PBX1	1.109	8.408	3.456e-13	9.672e-13	5.399	4.29
TMEM158	1.312	8.4	3.504e-13	9.803e-13	5.721	4.408
GYPB	1.779	8.638	3.969e-13	1.105e-12	6.973	5.194
GP9	1.067	8.398	4.652e-13	1.289e-12	5.382	4.315
LGALS2	-1.948	-9.335	4.829e-13	1.337e-12	2.674	4.622
PCDH9	-1.189	-8.959	6.21e-13	1.706e-12	3.351	4.54
CFD	-1.625	-8.444	7.739e-13	2.11e-12	6.711	8.336
CYP27A1	-1.075	-8.417	8.176e-13	2.225e-12	6.201	7.276
CPSF6	-1.139	-9.129	1.029e-12	2.783e-12	4.527	5.666
PLEK2	1.48	8.229	1.597e-12	4.264e-12	6.249	4.769
ERAP2	-1.655	-8.771	1.703e-12	4.534e-12	4.671	6.326
SLC14A1	1.362	8.047	1.88e-12	4.992e-12	4.923	3.56
CAMP	1.646	8.64	2.242e-12	5.917e-12	7.517	5.871
RPS27L	1.162	8.271	4.616e-12	1.199e-11	5.384	4.222
RGS18	1.229	8.392	4.861e-12	1.261e-11	8.079	6.85
IFI27	2.427	7.944	5.472e-12	1.416e-11	7.057	4.63
PSMC6	1.145	8.155	6.687e-12	1.721e-11	5.733	4.588
ZNF83	-1.254	-8.287	6.903e-12	1.775e-11	3.972	5.226
HBQ1	1.179	8.2	7.111e-12	1.826e-11	8.954	7.775
HBD	1.595	8.088	1.776e-11	4.462e-11	10.13	8.539
NPCDR1	-1.034	-8.931	2.138e-11	5.339e-11	2.188	3.222
XAF1	-1.087	-7.085	3.164e-11	7.789e-11	6.566	7.653
PADI2	1.085	8.055	4.405e-11	1.074e-10	7.879	6.793
SPTB	1.208	7.528	4.71e-11	1.146e-10	5.404	4.196
WLS	-1.067	-7.521	5.379e-11	1.304e-10	4.278	5.345
KCNH2	1.162	7.221	5.736e-11	1.388e-10	5.675	4.514
CSTA	1.006	8.045	7.474e-11	1.794e-10	9.078	8.072
TKTL1	-1.077	-8.253	9.371e-11	2.235e-10	2.355	3.432
MS4A3	1.739	7.329	9.987e-11	2.378e-10	5.396	3.657
SESN3	1.287	7.322	2.061e-10	4.783e-10	7.023	5.736
COMMD6	1.309	7.543	2.197e-10	5.085e-10	5.796	4.487
ALAS2	1.305	7.54	2.445e-10	5.634e-10	10.79	9.488
EVI2A	1.313	7.575	3.149e-10	7.203e-10	5.431	4.118
RSL24D1	1.523	7.551	3.83e-10	8.713e-10	5.549	4.026
RAP1GAP	1.287	6.915	4.878e-10	1.101e-09	4.364	3.077

gene_symbol	log2fc	t_stat	p_raw	p_adj	mean_sepsis	mean_healthy
IDO1	-1.352	-7.339	5.458e-10	1.227e-09	2.829	4.181
DDX60	-1.136	-6.641	7.153e-10	1.595e-09	4.429	5.565
BTN3A2	-1.437	-7.391	9.5e-10	2.101e-09	5.728	7.166
LILRA3	1.045	7.014	1.039e-09	2.293e-09	7.43	6.386
COMMD8	1.109	7.087	1.797e-09	3.883e-09	5.335	4.226
MCTP1	1.29	6.905	3.143e-09	6.685e-09	6.818	5.529
KRT23	-1.13	-6.439	3.575e-09	7.572e-09	6.35	7.48
DSC2	1.259	6.718	3.904e-09	8.245e-09	6.414	5.155
IFIT1	-1.718	-6.401	3.962e-09	8.357e-09	4.914	6.632
IFIT3	-1.336	-6.152	6.665e-09	1.384e-08	5.965	7.302
C6orf25	1.103	6.48	6.732e-09	1.397e-08	6.6	5.497
DEFA4	2.03	6.591	7.779e-09	1.606e-08	7.262	5.232
MX1	-1.303	-6.049	1.722e-08	3.479e-08	6.39	7.693
PI3	-1.257	-5.838	5.268e-08	1.03e-07	6.892	8.149
TMOD1	1.036	5.983	6.381e-08	1.24e-07	6.282	5.245
CTSG	1.072	5.776	7.218e-08	1.396e-07	4.658	3.586
FAM46C	1.094	5.884	1.016e-07	1.945e-07	9.12	8.026
MYOM2	-1.491	-5.882	5.191e-07	9.459e-07	3.072	4.564
APOBEC3B	1.467	5.071	2.901e-06	5.02e-06	4.62	3.153
HLA-DRB4	-1.632	-3.438	0.0012	0.001673	4.242	5.874
HLA-DQB1	-1.127	-3.313	0.001807	0.002481	4.171	5.298
TUBB2A	1.064	2.985	0.0042	0.005564	6.969	5.905

Table S2. Standardized L1-logistic-regression coefficients for the 10 genes of the locked signature (final fit on all 284 discovery sepsis/healthy samples, top-300 $|t|$ filter then L1-logistic at $C = 0.1$; Methods 4.5), with the fraction of 200 full-pipeline bootstrap replicates in which each gene was re-selected (Methods 4.7, Section 2.3).

gene_symbol	coef	reselection_frequency_200_bootstraps
S100A8	0.7835	0.415
NELL2	-0.3351	0.515
P2RY10	-0.2592	0.765
FAM43A	-0.2368	0.33
SBK1	-0.1788	0.265
FBXL16	-0.1443	0.355
FCER1A	-0.1334	0.39
NMT2	-0.06543	0.41
KLRB1	-0.02766	0.08
SLC7A6	-0.01127	0.18

Table S2 note. The re-selection column shows that the panel is not reproducible under resampling even though its discrimination is: no gene is re-selected in more than 77% of replicates, and the lead gene S100A8 is re-selected in 41.5%.

Table S3. Complete hypergeometric over-representation results: every term in any of the three gene-set libraries (MSigDB Hallmark 2020, KEGG 2021 Human, GO Biological Process 2023) with at least one member gene in the 9983-gene universe, for both query sets, unfiltered by significance (11,484 rows, 5,742 terms × 2 query sets; Methods 4.12). `p_adj` is corrected over the full family of testable terms per query set; `p_adj_within_library` is the secondary per-library correction. Sorted by query set and ascending raw *p*. Listed here are the 100 lowest raw-*p* terms of each query set; all 11,484 rows, with the overlapping genes, are in the data workbook, Supplementary File S3.

gene_list	library	term	k_ overlap	K_term_ size	n_ query	N_ universe	p_raw	p_adj_within_ library	p_adj	fdr_family
DE_genes_ fdr05_fc1	KEGG	Hematopoietic cell lineage	38	70	1508	9983	2.589e-14	8.285e-12	1.487e-10	all testable terms across Hallmark + KEGG + GO_BP, per query set
DE_genes_ fdr05_fc1	Hallmark	Allograft Rejection	61	156	1508	9983	1.666e-13	8.329e-12	4.782e-10	all testable terms across Hallmark + KEGG + GO_BP, per query set
DE_genes_ fdr05_fc1	GO_ BP	Positive Regulation Of T Cell Activation (GO:0050870)	36	79	1508	9983	9.842e-11	5.287e-07	1.884e-07	all testable terms across Hallmark + KEGG + GO_BP, per query set
DE_genes_ fdr05_fc1	KEGG	Th1 and Th2 cell differentiation	33	73	1508	9983	7.704e-10	1.233e-07	1.106e-06	all testable terms across Hallmark + KEGG + GO_BP, per query set
DE_genes_ fdr05_fc1	KEGG	Th17 cell differentiation	36	86	1508	9983	1.788e-09	1.908e-07	2.054e-06	all testable terms across Hallmark + KEGG + GO_BP, per query set
DE_genes_ fdr05_fc1	GO_ BP	Antigen Receptor-Mediated Signaling Pathway (GO:0050851)	39	99	1508	9983	3.124e-09	8.345e-06	2.99e-06	all testable terms across Hallmark + KEGG + GO_BP, per query set
DE_genes_ fdr05_fc1	GO_ BP	T Cell Activation (GO:0042110)	31	70	1508	9983	4.66e-09	8.345e-06	3.823e-06	all testable terms across Hallmark + KEGG + GO_BP, per query set
DE_genes_ fdr05_fc1	GO_ BP	Regulation Of T Cell Activation (GO:0050863)	22	42	1508	9983	1.825e-08	2.45e-05	1.31e-05	all testable terms across Hallmark + KEGG + GO_BP, per query set
DE_genes_ fdr05_fc1	KEGG	Asthma	13	17	1508	9983	2.668e-08	2.134e-06	1.702e-05	all testable terms across Hallmark + KEGG + GO_BP, per query set
DE_genes_ fdr05_fc1	Hallmark	IL-2/STAT5 Signaling	50	162	1508	9983	2.521e-07	6.303e-06	0.0001448	all testable terms across Hallmark + KEGG + GO_BP, per query set
DE_genes_ fdr05_fc1	GO_ BP	Regulation Of T Cell Differentiation (GO:0045580)	18	35	1508	9983	5.247e-07	0.0005638	0.0002635	all testable terms across Hallmark + KEGG + GO_BP, per query set
DE_genes_ fdr05_fc1	Hallmark	Interferon Gamma Response	52	175	1508	9983	5.507e-07	9.178e-06	0.0002635	all testable terms across Hallmark + KEGG + GO_BP, per query set
DE_genes_ fdr05_fc1	KEGG	Inflammatory bowel disease	20	43	1508	9983	9.816e-07	6.109e-05	0.0004336	all testable terms across Hallmark + KEGG + GO_BP, per query set
DE_genes_ fdr05_fc1	KEGG	Staphylococcus aureus infection	19	40	1508	9983	1.227e-06	6.109e-05	0.0005033	all testable terms across Hallmark + KEGG + GO_BP, per query set
DE_genes_ fdr05_fc1	KEGG	Graft-versus-host disease	14	24	1508	9983	1.336e-06	6.109e-05	0.0005116	all testable terms across Hallmark + KEGG + GO_BP, per query set
DE_genes_ fdr05_fc1	GO_ BP	Nucleosome Organization (GO:0034728)	20	45	1508	9983	2.39e-06	0.00214	0.0008343	all testable terms across Hallmark + KEGG + GO_BP, per query set
DE_genes_ fdr05_fc1	KEGG	Allograft rejection	14	25	1508	9983	2.615e-06	9.299e-05	0.0008343	all testable terms across Hallmark + KEGG + GO_BP, per query set
DE_genes_ fdr05_fc1	KEGG	Autoimmune thyroid disease	14	25	1508	9983	2.615e-06	9.299e-05	0.0008343	all testable terms across Hallmark + KEGG + GO_BP, per query set
DE_genes_ fdr05_fc1	GO_ BP	protein-DNA Complex Disassembly (GO:0032986)	10	14	1508	9983	3.359e-06	0.002339	0.001	all testable terms across Hallmark + KEGG + GO_BP, per query set
DE_genes_ fdr05_fc1	GO_ BP	T Cell Receptor Signaling Pathway (GO:0050852)	27	73	1508	9983	3.483e-06	0.002339	0.001	all testable terms across Hallmark + KEGG + GO_BP, per query set
DE_genes_ fdr05_fc1	GO_ BP	Immunoglobulin Mediated Immune Response (GO:0016064)	13	23	1508	9983	5.241e-06	0.00298	0.001433	all testable terms across Hallmark + KEGG + GO_BP, per query set
DE_genes_ fdr05_fc1	GO_ BP	Positive Regulation Of Cytokine Production (GO:0001819)	58	217	1508	9983	5.547e-06	0.00298	0.001448	all testable terms across Hallmark + KEGG + GO_BP, per query set
DE_genes_ fdr05_fc1	KEGG	Systemic lupus erythematosus	16	33	1508	9983	6.134e-06	0.0001963	0.001531	all testable terms across Hallmark + KEGG + GO_BP, per query set
DE_genes_ fdr05_fc1	GO_ BP	Defense Response To Bacterium (GO:0042742)	33	101	1508	9983	7.07e-06	0.003453	0.001691	all testable terms across Hallmark + KEGG + GO_BP, per query set
DE_genes_ fdr05_fc1	GO_ BP	Positive Regulation Of Lymphocyte Proliferation (GO:0050671)	21	52	1508	9983	8.595e-06	0.003848	0.001928	all testable terms across Hallmark + KEGG + GO_BP, per query set
DE_genes_ fdr05_fc1	KEGG	Type I diabetes mellitus	14	27	1508	9983	8.73e-06	0.000254	0.001928	all testable terms across Hallmark + KEGG + GO_BP, per query set
DE_genes_ fdr05_fc1	KEGG	Intestinal immune network for IgA production	15	31	1508	9983	1.239e-05	0.0003303	0.002634	all testable terms across Hallmark + KEGG + GO_BP, per query set
DE_genes_ fdr05_fc1	GO_ BP	Positive Regulation Of T Cell Differentiation (GO:0045582)	19	46	1508	9983	1.564e-05	0.005754	0.003075	all testable terms across Hallmark + KEGG + GO_BP, per query set
DE_genes_ fdr05_fc1	GO_ BP	Nucleosome Disassembly (GO:0006337)	9	13	1508	9983	1.607e-05	0.005754	0.003075	all testable terms across Hallmark + KEGG + GO_BP, per query set

gene_list	library	term	k_overlap	K_term_size	n_query	N_universe	p_raw	p_adj_within_library	p_adj	fdr_family
DE_genes_fdr05_fc1	GO_BP	Peptide Antigen Assembly With MHC Protein Complex (GO:0002501)	9	13	1508	9983	1.607e-05	0.005754	0.003075	all testable terms across Hallmark + KEGG + GO_BP, per query set
DE_genes_fdr05_fc1	GO_BP	Positive Regulation Of Lymphocyte Differentiation (GO:0045621)	15	32	1508	9983	2.008e-05	0.006741	0.003719	all testable terms across Hallmark + KEGG + GO_BP, per query set
DE_genes_fdr05_fc1	KEGG	Primary immunodeficiency	14	30	1508	9983	4.048e-05	0.0009963	0.007263	all testable terms across Hallmark + KEGG + GO_BP, per query set
DE_genes_fdr05_fc1	GO_BP	Antibacterial Humoral Response (GO:0019731)	10	17	1508	9983	4.215e-05	0.01294	0.007333	all testable terms across Hallmark + KEGG + GO_BP, per query set
DE_genes_fdr05_fc1	GO_BP	MHC Class II Protein Complex Assembly (GO:0002399)	7	9	1508	9983	4.816e-05	0.01294	0.007681	all testable terms across Hallmark + KEGG + GO_BP, per query set
DE_genes_fdr05_fc1	GO_BP	T-helper Cell Lineage Commitment (GO:0002295)	7	9	1508	9983	4.816e-05	0.01294	0.007681	all testable terms across Hallmark + KEGG + GO_BP, per query set
DE_genes_fdr05_fc1	GO_BP	Peptide Antigen Assembly With MHC Class II Protein Complex (GO:0002503)	7	9	1508	9983	4.816e-05	0.01294	0.007681	all testable terms across Hallmark + KEGG + GO_BP, per query set
DE_genes_fdr05_fc1	GO_BP	Defense Response To Gram-negative Bacterium (GO:0050829)	15	35	1508	9983	7.363e-05	0.01883	0.01143	all testable terms across Hallmark + KEGG + GO_BP, per query set
DE_genes_fdr05_fc1	Hallmark	Myc Targets V1	46	175	1508	9983	7.841e-05	0.0009801	0.01174	all testable terms across Hallmark + KEGG + GO_BP, per query set
DE_genes_fdr05_fc1	GO_BP	Nucleosome Assembly (GO:0006334)	13	28	1508	9983	8.171e-05	0.01995	0.01174	all testable terms across Hallmark + KEGG + GO_BP, per query set
DE_genes_fdr05_fc1	KEGG	Cell adhesion molecules	27	85	1508	9983	8.181e-05	0.00187	0.01174	all testable terms across Hallmark + KEGG + GO_BP, per query set
DE_genes_fdr05_fc1	GO_BP	B Cell Receptor Signaling Pathway (GO:0050853)	14	32	1508	9983	9.746e-05	0.02149	0.01332	all testable terms across Hallmark + KEGG + GO_BP, per query set
DE_genes_fdr05_fc1	GO_BP	Positive Regulation Of Leukocyte Cell-Cell Adhesion (GO:1903039)	14	32	1508	9983	9.746e-05	0.02149	0.01332	all testable terms across Hallmark + KEGG + GO_BP, per query set
DE_genes_fdr05_fc1	GO_BP	Inflammatory Response (GO:0006954)	40	147	1508	9983	0.0001	0.02149	0.01335	all testable terms across Hallmark + KEGG + GO_BP, per query set
DE_genes_fdr05_fc1	GO_BP	Antimicrobial Humoral Response (GO:0019730)	15	37	1508	9983	0.0001576	0.03114	0.02011	all testable terms across Hallmark + KEGG + GO_BP, per query set
DE_genes_fdr05_fc1	KEGG	Antigen processing and presentation	17	45	1508	9983	0.0001643	0.003505	0.02011	all testable terms across Hallmark + KEGG + GO_BP, per query set
DE_genes_fdr05_fc1	GO_BP	Antigen Processing And Presentation Of Exogenous Peptide Antigen Via MHC Class II (GO:0019886)	9	16	1508	9983	0.0001668	0.03114	0.02011	all testable terms across Hallmark + KEGG + GO_BP, per query set
DE_genes_fdr05_fc1	GO_BP	Antigen Processing And Presentation Of Peptide Antigen Via MHC Class II (GO:0002495)	9	16	1508	9983	0.0001668	0.03114	0.02011	all testable terms across Hallmark + KEGG + GO_BP, per query set
DE_genes_fdr05_fc1	GO_BP	Lymphocyte Migration (GO:0072676)	8	13	1508	9983	0.0001681	0.03114	0.02011	all testable terms across Hallmark + KEGG + GO_BP, per query set
DE_genes_fdr05_fc1	GO_BP	Positive Regulation Of Type II Interferon Production (GO:0032729)	17	46	1508	9983	0.0002243	0.04017	0.02629	all testable terms across Hallmark + KEGG + GO_BP, per query set
DE_genes_fdr05_fc1	Hallmark	Complement	39	148	1508	9983	0.0002524	0.002524	0.02899	all testable terms across Hallmark + KEGG + GO_BP, per query set
DE_genes_fdr05_fc1	GO_BP	Regulation Of Type II Interferon Production (GO:0032649)	21	64	1508	9983	0.0002985	0.05173	0.03361	all testable terms across Hallmark + KEGG + GO_BP, per query set
DE_genes_fdr05_fc1	GO_BP	Immunoglobulin Production Involved In Immunoglobulin-Mediated Immune Response (GO:0002381)	7	11	1508	9983	0.0003335	0.05598	0.03682	all testable terms across Hallmark + KEGG + GO_BP, per query set
DE_genes_fdr05_fc1	GO_BP	T-helper 17 Cell Lineage Commitment (GO:0072540)	5	6	1508	9983	0.0004103	0.06386	0.04388	all testable terms across Hallmark + KEGG + GO_BP, per query set
DE_genes_fdr05_fc1	GO_BP	protein-DNA Complex Assembly (GO:0065004)	13	32	1508	9983	0.00042	0.06386	0.04388	all testable terms across Hallmark + KEGG + GO_BP, per query set
DE_genes_fdr05_fc1	GO_BP	Negative Regulation Of Viral Genome Replication (GO:0045071)	16	44	1508	9983	0.000422	0.06386	0.04388	all testable terms across Hallmark + KEGG + GO_BP, per query set
DE_genes_fdr05_fc1	GO_BP	Antigen Processing And Presentation Of Exogenous Peptide Antigen (GO:0002478)	10	21	1508	9983	0.0004279	0.06386	0.04388	all testable terms across Hallmark + KEGG + GO_BP, per query set
DE_genes_fdr05_fc1	GO_BP	T-helper 1 Cell Differentiation (GO:0045063)	4	4	1508	9983	0.0005189	0.07534	0.05227	all testable terms across Hallmark + KEGG + GO_BP, per query set
DE_genes_fdr05_fc1	GO_BP	Cellular Defense Response (GO:0006968)	12	29	1508	9983	0.0005718	0.08083	0.0566	all testable terms across Hallmark + KEGG + GO_BP, per query set
DE_genes_fdr05_fc1	GO_BP	Positive Regulation Of Lymphocyte Activation (GO:0051251)	13	33	1508	9983	0.0005982	0.0824	0.05822	all testable terms across Hallmark + KEGG + GO_BP, per query set
DE_genes_fdr05_fc1	GO_BP	Positive Regulation Of B Cell Proliferation (GO:0030890)	10	22	1508	9983	0.0006791	0.08902	0.0643	all testable terms across Hallmark + KEGG + GO_BP, per query set
DE_genes_fdr05_fc1	GO_BP	T-helper Cell Differentiation (GO:0042093)	7	12	1508	9983	0.000696	0.08902	0.0643	all testable terms across Hallmark + KEGG + GO_BP, per query set

gene_list	library	term	k_ overlap	K_term_ size	n_ query	N_ universe	p_raw	p_adj_within_ library	p_adj	fdr_family
DE_genes_ fdr05_fc1	GO_ BP	Regulation Of Lymphocyte Activation (GO:0051249)	7	12	1508	9983	0.000696	0.08902	0.0643	all testable terms across Hallmark + KEGG + GO_BP, per query set
DE_genes_ fdr05_fc1	KEGG	Transcriptional misregulation in cancer	33	125	1508	9983	0.0007055	0.01365	0.0643	all testable terms across Hallmark + KEGG + GO_BP, per query set
DE_genes_ fdr05_fc1	KEGG	Human T-cell leukemia virus 1 infection	43	176	1508	9983	0.0007251	0.01365	0.06506	all testable terms across Hallmark + KEGG + GO_BP, per query set
DE_genes_ fdr05_fc1	GO_ BP	B Cell Activation (GO:0042113)	19	59	1508	9983	0.0007417	0.09267	0.06552	all testable terms across Hallmark + KEGG + GO_BP, per query set
DE_genes_ fdr05_fc1	KEGG	Viral myocarditis	15	42	1508	9983	0.0007898	0.01404	0.06872	all testable terms across Hallmark + KEGG + GO_BP, per query set
DE_genes_ fdr05_fc1	Hallmark	KRAS Signaling Up	32	121	1508	9983	0.0008168	0.006807	0.07	all testable terms across Hallmark + KEGG + GO_BP, per query set
DE_genes_ fdr05_fc1	GO_ BP	Antigen Processing And Presentation Of Peptide Antigen Via MHC Class I (GO:0002474)	8	16	1508	9983	0.001099	0.1342	0.09279	all testable terms across Hallmark + KEGG + GO_BP, per query set
DE_genes_ fdr05_fc1	GO_ BP	T-helper 17 Cell Differentiation (GO:0072539)	5	7	1508	9983	0.001257	0.1431	0.1019	all testable terms across Hallmark + KEGG + GO_BP, per query set
DE_genes_ fdr05_fc1	GO_ BP	Innate Immune Response In Mucosa (GO:0002227)	5	7	1508	9983	0.001257	0.1431	0.1019	all testable terms across Hallmark + KEGG + GO_BP, per query set
DE_genes_ fdr05_fc1	GO_ BP	Regulation Of T Cell Proliferation (GO:0042129)	16	48	1508	9983	0.001259	0.1431	0.1019	all testable terms across Hallmark + KEGG + GO_BP, per query set
DE_genes_ fdr05_fc1	GO_ BP	Negative Regulation Of Cytokine Production (GO:0001818)	31	119	1508	9983	0.001278	0.1431	0.1019	all testable terms across Hallmark + KEGG + GO_BP, per query set
DE_genes_ fdr05_fc1	GO_ BP	Regulation Of Cytokine Production (GO:0001817)	26	95	1508	9983	0.001423	0.1447	0.1064	all testable terms across Hallmark + KEGG + GO_BP, per query set
DE_genes_ fdr05_fc1	GO_ BP	Cellular Response To Interleukin-4 (GO:0071353)	6	10	1508	9983	0.001427	0.1447	0.1064	all testable terms across Hallmark + KEGG + GO_BP, per query set
DE_genes_ fdr05_fc1	GO_ BP	Negative Regulation Of Cation Transmembrane Transport (GO:1904063)	6	10	1508	9983	0.001427	0.1447	0.1064	all testable terms across Hallmark + KEGG + GO_BP, per query set
DE_genes_ fdr05_fc1	GO_ BP	Nuclear RNA Surveillance (GO:0071027)	6	10	1508	9983	0.001427	0.1447	0.1064	all testable terms across Hallmark + KEGG + GO_BP, per query set
DE_genes_ fdr05_fc1	GO_ BP	Positive Regulation Of Endothelial Cell Apoptotic Process (GO:2000353)	6	10	1508	9983	0.001427	0.1447	0.1064	all testable terms across Hallmark + KEGG + GO_BP, per query set
DE_genes_ fdr05_fc1	GO_ BP	Antimicrobial Humoral Immune Response Mediated By Antimicrobial Peptide (GO:0061844)	10	24	1508	9983	0.001544	0.1536	0.1137	all testable terms across Hallmark + KEGG + GO_BP, per query set
DE_genes_ fdr05_fc1	GO_ BP	Response To Cytokine (GO:0034097)	26	96	1508	9983	0.001678	0.1638	0.1219	all testable terms across Hallmark + KEGG + GO_BP, per query set
DE_genes_ fdr05_fc1	GO_ BP	Cellular Response To Cytokine Stimulus (GO:0071345)	43	185	1508	9983	0.002087	0.1996	0.1498	all testable terms across Hallmark + KEGG + GO_BP, per query set
DE_genes_ fdr05_fc1	GO_ BP	T Cell Differentiation (GO:0030217)	12	33	1508	9983	0.002181	0.1996	0.1506	all testable terms across Hallmark + KEGG + GO_BP, per query set
DE_genes_ fdr05_fc1	GO_ BP	Regulation Of B Cell Proliferation (GO:0030888)	12	33	1508	9983	0.002181	0.1996	0.1506	all testable terms across Hallmark + KEGG + GO_BP, per query set
DE_genes_ fdr05_fc1	KEGG	Toxoplasmosis	23	83	1508	9983	0.002191	0.0369	0.1506	all testable terms across Hallmark + KEGG + GO_BP, per query set
DE_genes_ fdr05_fc1	GO_ BP	Positive Regulation Of Myoblast Differentiation (GO:0045663)	10	25	1508	9983	0.002229	0.1996	0.1506	all testable terms across Hallmark + KEGG + GO_BP, per query set
DE_genes_ fdr05_fc1	GO_ BP	Positive Regulation Of Viral Genome Replication (GO:0045070)	10	25	1508	9983	0.002229	0.1996	0.1506	all testable terms across Hallmark + KEGG + GO_BP, per query set
DE_genes_ fdr05_fc1	GO_ BP	Regulation Of Telomerase RNA Localization To Cajal Body (GO:1904872)	7	14	1508	9983	0.002283	0.2011	0.1525	all testable terms across Hallmark + KEGG + GO_BP, per query set
DE_genes_ fdr05_fc1	GO_ BP	RNA Processing (GO:0006396)	35	144	1508	9983	0.002379	0.2062	0.157	all testable terms across Hallmark + KEGG + GO_BP, per query set
DE_genes_ fdr05_fc1	GO_ BP	Regulation Of Cell Differentiation (GO:0045595)	31	124	1508	9983	0.002584	0.2204	0.1686	all testable terms across Hallmark + KEGG + GO_BP, per query set
DE_genes_ fdr05_fc1	GO_ BP	Response To Lipopolysaccharide (GO:0032496)	26	99	1508	9983	0.002686	0.2234	0.1733	all testable terms across Hallmark + KEGG + GO_BP, per query set
DE_genes_ fdr05_fc1	GO_ BP	Positive Regulation Of Homotypic Cell-Cell Adhesion (GO:0034112)	6	11	1508	9983	0.00274	0.2234	0.1746	all testable terms across Hallmark + KEGG + GO_BP, per query set
DE_genes_ fdr05_fc1	GO_ BP	Regulation Of Lymphocyte Differentiation (GO:0045619)	8	18	1508	9983	0.002819	0.2234	0.1746	all testable terms across Hallmark + KEGG + GO_BP, per query set
DE_genes_ fdr05_fc1	GO_ BP	Regulation Of Platelet Aggregation (GO:0090330)	8	18	1508	9983	0.002819	0.2234	0.1746	all testable terms across Hallmark + KEGG + GO_BP, per query set
DE_genes_ fdr05_fc1	GO_ BP	Regulation Of Viral Genome Replication (GO:0045069)	17	56	1508	9983	0.002828	0.2234	0.1746	all testable terms across Hallmark + KEGG + GO_BP, per query set
DE_genes_ fdr05_fc1	GO_ BP	Mucosal Immune Response (GO:0002385)	5	8	1508	9983	0.002934	0.2252	0.1773	all testable terms across Hallmark + KEGG + GO_BP, per query set

gene_list	library	term	k_ overlap	K_term_ size	n_ query	N_ universe	p_raw	p_adj_within_ library	p_adj	fdr_family
DE_genes_ fdr05_fc1	GO_ BP	Positive Regulation Of T-helper 1 Type Immune Response (GO:0002827)	5	8	1508	9983	0.002934	0.2252	0.1773	all testable terms across Hallmark + KEGG + GO_BP, per query set
DE_genes_ fdr05_fc1	GO_ BP	Endothelial Cell Migration (GO:0043542)	10	26	1508	9983	0.003138	0.2342	0.1858	all testable terms across Hallmark + KEGG + GO_BP, per query set
DE_genes_ fdr05_fc1	GO_ BP	Positive Regulation Of Cell-Cell Adhesion (GO:0022409)	10	26	1508	9983	0.003138	0.2342	0.1858	all testable terms across Hallmark + KEGG + GO_BP, per query set
DE_genes_ fdr05_fc1	GO_ BP	Regulation Of Phagocytosis (GO:0050764)	14	43	1508	9983	0.003193	0.235	0.1871	all testable terms across Hallmark + KEGG + GO_BP, per query set
DE_genes_ fdr05_fc1	Hallmark	TNF-alpha Signaling via NF-kB	36	152	1508	9983	0.003319	0.02371	0.1925	all testable terms across Hallmark + KEGG + GO_BP, per query set
DE_genes_ fdr05_fc1	GO_ BP	Leukocyte Adhesion To Vascular Endothelial Cell (GO:0061756)	7	15	1508	9983	0.003728	0.2706	0.214	all testable terms across Hallmark + KEGG + GO_BP, per query set
locked_ signature_10gene	GO_ BP	Axon Extension Involved In Axon Guidance (GO:0048846)	1	2	10	9983	0.002003	1	1	all testable terms across Hallmark + KEGG + GO_BP, per query set
locked_ signature_10gene	GO_ BP	Neuron Projection Extension Involved In Neuron Projection Guidance (GO:1902284)	1	2	10	9983	0.002003	1	1	all testable terms across Hallmark + KEGG + GO_BP, per query set
locked_ signature_10gene	GO_ BP	Ganglion Development (GO:0061548)	1	3	10	9983	0.003002	1	1	all testable terms across Hallmark + KEGG + GO_BP, per query set
locked_ signature_10gene	GO_ BP	Mast Cell Activation Involved In Immune Response (GO:0002279)	1	3	10	9983	0.003002	1	1	all testable terms across Hallmark + KEGG + GO_BP, per query set
locked_ signature_10gene	GO_ BP	Mast Cell Degranulation (GO:0043303)	1	3	10	9983	0.003002	1	1	all testable terms across Hallmark + KEGG + GO_BP, per query set
locked_ signature_10gene	GO_ BP	Ornithine Transport (GO:0015822)	1	3	10	9983	0.003002	1	1	all testable terms across Hallmark + KEGG + GO_BP, per query set
locked_ signature_10gene	GO_ BP	Regulation Of Natural Killer Cell Mediated Immunity (GO:0002715)	1	3	10	9983	0.003002	1	1	all testable terms across Hallmark + KEGG + GO_BP, per query set
locked_ signature_10gene	GO_ BP	Regulation Of Rhodopsin Mediated Signaling Pathway (GO:0022400)	1	3	10	9983	0.003002	1	1	all testable terms across Hallmark + KEGG + GO_BP, per query set
locked_ signature_10gene	GO_ BP	Sympathetic Ganglion Development (GO:0061549)	1	3	10	9983	0.003002	1	1	all testable terms across Hallmark + KEGG + GO_BP, per query set
locked_ signature_10gene	GO_ BP	Mast Cell Mediated Immunity (GO:0002448)	1	4	10	9983	0.004001	1	1	all testable terms across Hallmark + KEGG + GO_BP, per query set
locked_ signature_10gene	GO_ BP	Amino-Acid Betaine Transport (GO:0015838)	1	5	10	9983	0.004999	1	1	all testable terms across Hallmark + KEGG + GO_BP, per query set
locked_ signature_10gene	GO_ BP	Leukocyte Aggregation (GO:0070486)	1	5	10	9983	0.004999	1	1	all testable terms across Hallmark + KEGG + GO_BP, per query set
locked_ signature_10gene	GO_ BP	Regulation Of Leukocyte Mediated Cytotoxicity (GO:0001910)	1	5	10	9983	0.004999	1	1	all testable terms across Hallmark + KEGG + GO_BP, per query set
locked_ signature_10gene	GO_ BP	L-arginine Transmembrane Transport (GO:1903826)	1	6	10	9983	0.005997	1	1	all testable terms across Hallmark + KEGG + GO_BP, per query set
locked_ signature_10gene	GO_ BP	Basic Amino Acid Transmembrane Transport (GO:1990822)	1	6	10	9983	0.005997	1	1	all testable terms across Hallmark + KEGG + GO_BP, per query set
locked_ signature_10gene	GO_ BP	Leucine Transport (GO:0015820)	1	6	10	9983	0.005997	1	1	all testable terms across Hallmark + KEGG + GO_BP, per query set
locked_ signature_10gene	GO_ BP	Peptidyl-Cysteine Modification (GO:0018198)	1	6	10	9983	0.005997	1	1	all testable terms across Hallmark + KEGG + GO_BP, per query set
locked_ signature_10gene	GO_ BP	Sympathetic Nervous System Development (GO:0048485)	1	6	10	9983	0.005997	1	1	all testable terms across Hallmark + KEGG + GO_BP, per query set
locked_ signature_10gene	GO_ BP	Branched-Chain Amino Acid Transport (GO:0015803)	1	7	10	9983	0.006993	1	1	all testable terms across Hallmark + KEGG + GO_BP, per query set
locked_ signature_10gene	GO_ BP	Nitric Oxide Biosynthetic Process (GO:0006809)	1	8	10	9983	0.007988	1	1	all testable terms across Hallmark + KEGG + GO_BP, per query set
locked_ signature_10gene	GO_ BP	Nitric Oxide Metabolic Process (GO:0046209)	1	8	10	9983	0.007988	1	1	all testable terms across Hallmark + KEGG + GO_BP, per query set
locked_ signature_10gene	GO_ BP	Outflow Tract Septum Morphogenesis (GO:0003148)	1	8	10	9983	0.007988	1	1	all testable terms across Hallmark + KEGG + GO_BP, per query set
locked_ signature_10gene	GO_ BP	Semaphorin-Plexin Signaling Pathway Involved In Neuron Projection Guidance (GO:1902285)	1	8	10	9983	0.007988	1	1	all testable terms across Hallmark + KEGG + GO_BP, per query set
locked_ signature_10gene	GO_ BP	Positive Regulation Of Cytosolic Calcium Ion Concentration Involved In Phospholipase C-activating G Protein-Coupled Signaling Pathway (GO:0051482)	1	13	10	9983	0.01295	1	1	all testable terms across Hallmark + KEGG + GO_BP, per query set
locked_ signature_10gene	GO_ BP	Cellular Nitrogen Compound Biosynthetic Process (GO:0044271)	1	15	10	9983	0.01493	1	1	all testable terms across Hallmark + KEGG + GO_BP, per query set
locked_ signature_10gene	GO_ BP	Defense Response To Fungus (GO:0050832)	1	16	10	9983	0.01592	1	1	all testable terms across Hallmark + KEGG + GO_BP, per query set

gene_list	library	term	k_overlap	K_term_size	n_query	N_universe	p_raw	p_adj_within_library	p_adj	fdr_family
locked_signature_10gene	GO_BP	Positive Regulation Of Rho Protein Signal Transduction (GO:0035025)	1	16	10	9983	0.01592	1	1	all testable terms across Hallmark + KEGG + GO_BP, per query set
locked_signature_10gene	KEGG	Asthma	1	17	10	9983	0.01691	1	1	all testable terms across Hallmark + KEGG + GO_BP, per query set
locked_signature_10gene	GO_BP	Axon Extension (GO:0048675)	1	17	10	9983	0.01691	1	1	all testable terms across Hallmark + KEGG + GO_BP, per query set
locked_signature_10gene	GO_BP	Organic Cation Transport (GO:0015695)	1	17	10	9983	0.01691	1	1	all testable terms across Hallmark + KEGG + GO_BP, per query set
locked_signature_10gene	GO_BP	Modified Amino Acid Transport (GO:0072337)	1	18	10	9983	0.01789	1	1	all testable terms across Hallmark + KEGG + GO_BP, per query set
locked_signature_10gene	GO_BP	Regulation Of Natural Killer Cell Mediated Cytotoxicity (GO:0042269)	1	19	10	9983	0.01888	1	1	all testable terms across Hallmark + KEGG + GO_BP, per query set
locked_signature_10gene	GO_BP	Neutral Amino Acid Transport (GO:0015804)	1	20	10	9983	0.01986	1	1	all testable terms across Hallmark + KEGG + GO_BP, per query set
locked_signature_10gene	GO_BP	Semaphorin-Plexin Signaling Pathway (GO:0071526)	1	21	10	9983	0.02085	1	1	all testable terms across Hallmark + KEGG + GO_BP, per query set
locked_signature_10gene	GO_BP	Amino Acid Transmembrane Transport (GO:0003333)	1	23	10	9983	0.02281	1	1	all testable terms across Hallmark + KEGG + GO_BP, per query set
locked_signature_10gene	GO_BP	Carboxylic Acid Transmembrane Transport (GO:1905039)	1	23	10	9983	0.02281	1	1	all testable terms across Hallmark + KEGG + GO_BP, per query set
locked_signature_10gene	GO_BP	Immunoglobulin Mediated Immune Response (GO:0016064)	1	23	10	9983	0.02281	1	1	all testable terms across Hallmark + KEGG + GO_BP, per query set
locked_signature_10gene	GO_BP	Amino Acid Transport (GO:0006865)	1	25	10	9983	0.02477	1	1	all testable terms across Hallmark + KEGG + GO_BP, per query set
locked_signature_10gene	GO_BP	Lysosome Localization (GO:0032418)	1	25	10	9983	0.02477	1	1	all testable terms across Hallmark + KEGG + GO_BP, per query set
locked_signature_10gene	GO_BP	Neuron Projection Extension (GO:1990138)	1	25	10	9983	0.02477	1	1	all testable terms across Hallmark + KEGG + GO_BP, per query set
locked_signature_10gene	GO_BP	L-alpha-amino Acid Transmembrane Transport (GO:1902475)	1	26	10	9983	0.02575	1	1	all testable terms across Hallmark + KEGG + GO_BP, per query set
locked_signature_10gene	GO_BP	Phospholipase C-activating G Protein-Coupled Receptor Signaling Pathway (GO:0007200)	1	28	10	9983	0.02771	1	1	all testable terms across Hallmark + KEGG + GO_BP, per query set
locked_signature_10gene	KEGG	Malaria	1	29	10	9983	0.02869	1	1	all testable terms across Hallmark + KEGG + GO_BP, per query set
locked_signature_10gene	GO_BP	Neutrophil Chemotaxis (GO:0030593)	1	29	10	9983	0.02869	1	1	all testable terms across Hallmark + KEGG + GO_BP, per query set
locked_signature_10gene	GO_BP	Positive Regulation Of Ras Protein Signal Transduction (GO:0046579)	1	29	10	9983	0.02869	1	1	all testable terms across Hallmark + KEGG + GO_BP, per query set
locked_signature_10gene	GO_BP	Positive Regulation Of Intrinsic Apoptotic Signaling Pathway (GO:2001244)	1	30	10	9983	0.02966	1	1	all testable terms across Hallmark + KEGG + GO_BP, per query set
locked_signature_10gene	GO_BP	SCF-dependent Proteasomal Ubiquitin-Dependent Protein Catabolic Process (GO:0031146)	1	31	10	9983	0.03064	1	1	all testable terms across Hallmark + KEGG + GO_BP, per query set
locked_signature_10gene	GO_BP	Granulocyte Chemotaxis (GO:0071621)	1	32	10	9983	0.03161	1	1	all testable terms across Hallmark + KEGG + GO_BP, per query set
locked_signature_10gene	GO_BP	Neutrophil Migration (GO:1990266)	1	34	10	9983	0.03356	1	1	all testable terms across Hallmark + KEGG + GO_BP, per query set
locked_signature_10gene	GO_BP	Regulation Of Intrinsic Apoptotic Signaling Pathway (GO:2001242)	1	39	10	9983	0.0384	1	1	all testable terms across Hallmark + KEGG + GO_BP, per query set
locked_signature_10gene	GO_BP	Positive Regulation Of Endothelial Cell Proliferation (GO:0001938)	1	41	10	9983	0.04034	1	1	all testable terms across Hallmark + KEGG + GO_BP, per query set
locked_signature_10gene	GO_BP	Regulation Of G Protein-Coupled Receptor Signaling Pathway (GO:0008277)	1	43	10	9983	0.04227	1	1	all testable terms across Hallmark + KEGG + GO_BP, per query set
locked_signature_10gene	GO_BP	Regulation Of Rho Protein Signal Transduction (GO:0035023)	1	44	10	9983	0.04323	1	1	all testable terms across Hallmark + KEGG + GO_BP, per query set
locked_signature_10gene	GO_BP	Response To Molecule Of Bacterial Origin (GO:0002237)	1	44	10	9983	0.04323	1	1	all testable terms across Hallmark + KEGG + GO_BP, per query set
locked_signature_10gene	GO_BP	Positive Regulation Of Apoptotic Signaling Pathway (GO:2001235)	1	46	10	9983	0.04515	1	1	all testable terms across Hallmark + KEGG + GO_BP, per query set
locked_signature_10gene	GO_BP	Regulation Of Endothelial Cell Migration (GO:0010594)	1	48	10	9983	0.04708	1	1	all testable terms across Hallmark + KEGG + GO_BP, per query set
locked_signature_10gene	GO_BP	Peptidyl-Threonine Phosphorylation (GO:0018107)	1	49	10	9983	0.04803	1	1	all testable terms across Hallmark + KEGG + GO_BP, per query set
locked_signature_10gene	GO_BP	Positive Regulation Of Growth (GO:0045927)	1	50	10	9983	0.04899	1	1	all testable terms across Hallmark + KEGG + GO_BP, per query set
locked_signature_10gene	GO_BP	Regulation Of Endothelial Cell Proliferation (GO:0001936)	1	50	10	9983	0.04899	1	1	all testable terms across Hallmark + KEGG + GO_BP, per query set

gene_list	library	term	k_overlap	K_term_size	n_query	N_universe	p_raw	p_adj_within_library	p_adj	fdr_family
locked_signature_10gene	KEGG	Fc epsilon RI signaling pathway	1	51	10	9983	0.04995	1	1	all testable terms across Hallmark + KEGG + GO_BP, per query set
locked_signature_10gene	GO_BP	Activation Of Cysteine-Type Endopeptidase Activity Involved In Apoptotic Process (GO:0006919)	1	52	10	9983	0.05091	1	1	all testable terms across Hallmark + KEGG + GO_BP, per query set
locked_signature_10gene	GO_BP	Positive Regulation Of Endothelial Cell Migration (GO:0010595)	1	52	10	9983	0.05091	1	1	all testable terms across Hallmark + KEGG + GO_BP, per query set
locked_signature_10gene	GO_BP	Peptidyl-Threonine Modification (GO:0018210)	1	53	10	9983	0.05186	1	1	all testable terms across Hallmark + KEGG + GO_BP, per query set
locked_signature_10gene	KEGG	IL-17 signaling pathway	1	54	10	9983	0.05282	1	1	all testable terms across Hallmark + KEGG + GO_BP, per query set
locked_signature_10gene	GO_BP	Positive Regulation Of Epithelial Cell Proliferation (GO:0050679)	1	56	10	9983	0.05472	1	1	all testable terms across Hallmark + KEGG + GO_BP, per query set
locked_signature_10gene	GO_BP	Neuron Projection Guidance (GO:0097485)	1	61	10	9983	0.05948	1	1	all testable terms across Hallmark + KEGG + GO_BP, per query set
locked_signature_10gene	GO_BP	Positive Regulation Of Epithelial Cell Migration (GO:0010634)	1	63	10	9983	0.06137	1	1	all testable terms across Hallmark + KEGG + GO_BP, per query set
locked_signature_10gene	GO_BP	Positive Regulation Of Cell Growth (GO:0030307)	1	64	10	9983	0.06232	1	1	all testable terms across Hallmark + KEGG + GO_BP, per query set
locked_signature_10gene	GO_BP	Response To Lipid (GO:0033993)	1	70	10	9983	0.06798	1	1	all testable terms across Hallmark + KEGG + GO_BP, per query set
locked_signature_10gene	GO_BP	Positive Regulation Of Inflammatory Response (GO:0050729)	1	72	10	9983	0.06986	1	1	all testable terms across Hallmark + KEGG + GO_BP, per query set
locked_signature_10gene	GO_BP	Axon Guidance (GO:0007411)	1	74	10	9983	0.07173	1	1	all testable terms across Hallmark + KEGG + GO_BP, per query set
locked_signature_10gene	GO_BP	Regulation Of Cytoskeleton Organization (GO:0051493)	1	75	10	9983	0.07267	1	1	all testable terms across Hallmark + KEGG + GO_BP, per query set
locked_signature_10gene	GO_BP	Regulation Of Response To External Stimulus (GO:0032101)	1	78	10	9983	0.07547	1	1	all testable terms across Hallmark + KEGG + GO_BP, per query set
locked_signature_10gene	GO_BP	Regulation Of Organelle Organization (GO:0033043)	1	82	10	9983	0.0792	1	1	all testable terms across Hallmark + KEGG + GO_BP, per query set
locked_signature_10gene	GO_BP	Positive Regulation Of Cysteine-Type Endopeptidase Activity Involved In Apoptotic Process (GO:0043280)	1	85	10	9983	0.08199	1	1	all testable terms across Hallmark + KEGG + GO_BP, per query set
locked_signature_10gene	GO_BP	Positive Regulation Of Defense Response (GO:0031349)	1	88	10	9983	0.08477	1	1	all testable terms across Hallmark + KEGG + GO_BP, per query set
locked_signature_10gene	KEGG	Sphingolipid signaling pathway	1	92	10	9983	0.08847	1	1	all testable terms across Hallmark + KEGG + GO_BP, per query set
locked_signature_10gene	KEGG	Neuroactive ligand-receptor interaction	1	97	10	9983	0.09307	1	1	all testable terms across Hallmark + KEGG + GO_BP, per query set
locked_signature_10gene	KEGG	Phospholipase D signaling pathway	1	98	10	9983	0.09398	1	1	all testable terms across Hallmark + KEGG + GO_BP, per query set
locked_signature_10gene	GO_BP	Response To Lipopolysaccharide (GO:0032496)	1	99	10	9983	0.0949	1	1	all testable terms across Hallmark + KEGG + GO_BP, per query set
locked_signature_10gene	GO_BP	Axonogenesis (GO:0007409)	1	101	10	9983	0.09673	1	1	all testable terms across Hallmark + KEGG + GO_BP, per query set
locked_signature_10gene	GO_BP	Defense Response To Bacterium (GO:0042742)	1	101	10	9983	0.09673	1	1	all testable terms across Hallmark + KEGG + GO_BP, per query set
locked_signature_10gene	GO_BP	Positive Regulation Of Response To External Stimulus (GO:0032103)	1	110	10	9983	0.1049	1	1	all testable terms across Hallmark + KEGG + GO_BP, per query set
locked_signature_10gene	GO_BP	Positive Regulation Of NF-kappaB Transcription Factor Activity (GO:0051092)	1	113	10	9983	0.1076	1	1	all testable terms across Hallmark + KEGG + GO_BP, per query set
locked_signature_10gene	GO_BP	Peptidyl-Serine Modification (GO:0018209)	1	120	10	9983	0.114	1	1	all testable terms across Hallmark + KEGG + GO_BP, per query set
locked_signature_10gene	GO_BP	Peptidyl-Serine Phosphorylation (GO:0018105)	1	121	10	9983	0.1149	1	1	all testable terms across Hallmark + KEGG + GO_BP, per query set
locked_signature_10gene	GO_BP	Regulation Of Cell Growth (GO:0001558)	1	146	10	9983	0.137	1	1	all testable terms across Hallmark + KEGG + GO_BP, per query set
locked_signature_10gene	GO_BP	Inflammatory Response (GO:0006954)	1	147	10	9983	0.1379	1	1	all testable terms across Hallmark + KEGG + GO_BP, per query set
locked_signature_10gene	GO_BP	Regulation Of Inflammatory Response (GO:0050727)	1	149	10	9983	0.1397	1	1	all testable terms across Hallmark + KEGG + GO_BP, per query set
locked_signature_10gene	GO_BP	Positive Regulation Of DNA-binding Transcription Factor Activity (GO:0051091)	1	172	10	9983	0.1596	1	1	all testable terms across Hallmark + KEGG + GO_BP, per query set
locked_signature_10gene	GO_BP	Proteasome-Mediated Ubiquitin-Dependent Protein Catabolic Process (GO:0043161)	1	258	10	9983	0.2305	1	1	all testable terms across Hallmark + KEGG + GO_BP, per query set

gene_list	library	term	k_ overlap	K_term_ size	n_ query	N_ universe	p_raw	p_adj_within_ library	p_adj	fdr_family
locked_ signature_10gene	GO_ BP	Positive Regulation Of Cellular Process (GO:0048522)	1	343	10	9983	0.2952	1	1	all testable terms across Hallmark + KEGG + GO_BP, per query set
locked_ signature_10gene	GO_ BP	Positive Regulation Of Intracellular Signal Transduction (GO:1902533)	1	346	10	9983	0.2974	1	1	all testable terms across Hallmark + KEGG + GO_BP, per query set
locked_ signature_10gene	GO_ BP	Protein Phosphorylation (GO:0006468)	1	347	10	9983	0.2981	1	1	all testable terms across Hallmark + KEGG + GO_BP, per query set
locked_ signature_10gene	Hallmark	TNF-alpha Signaling via NF-kB	0	152	10	9983	1	1	1	all testable terms across Hallmark + KEGG + GO_BP, per query set
locked_ signature_10gene	Hallmark	Hypoxia	0	139	10	9983	1	1	1	all testable terms across Hallmark + KEGG + GO_BP, per query set
locked_ signature_10gene	Hallmark	Cholesterol Homeostasis	0	56	10	9983	1	1	1	all testable terms across Hallmark + KEGG + GO_BP, per query set
locked_ signature_10gene	Hallmark	Mitotic Spindle	0	169	10	9983	1	1	1	all testable terms across Hallmark + KEGG + GO_BP, per query set
locked_ signature_10gene	Hallmark	Wnt-beta Catenin Signaling	0	29	10	9983	1	1	1	all testable terms across Hallmark + KEGG + GO_BP, per query set
locked_ signature_10gene	Hallmark	TGF-beta Signaling	0	44	10	9983	1	1	1	all testable terms across Hallmark + KEGG + GO_BP, per query set

Table S3 note. Every testable term enters the correction. The minimum-overlap filter is applied afterwards, for display only, and affects no p-value. All 56 terms that reached FDR $p < 0.05$ are listed in full in Table S4, with their overlapping genes, so no significant term is omitted here.

Table S4. FDR-significant ($p$adj < 0.05) over-representation terms for the 1508-gene discovery differential-expression set (56 terms, with overlap ≥ 3 applied for display only). Underlies Figure 7.

gene_list	library	term	k_overlap	K_term_size	n_query	N_universe	genes	p_raw	p_adj_within_library	p_adj	fdr_family
DE_genes_fdr05_fc1	GO_BP	Positive Regulation Of T Cell Activation (GO:0050870)	36	79	1508	9983	ACTL6A;ANXA1;ARID1B;CCL5;CCR7;CD1D;CD28;CD3E;CD6;CD81;CD86;FLOT2;GATA3;HLA-DMA;HLA-DMB;HLA-DOA;HLA-DPA1;HLA-DQA1;HLA-DRA;HLA-DRB4;HSPD1;IL23A;JAK2;LCK;PHF10;PRKCQ;RHOH;SIRPG;SMARCA4;SMARCC1;SMARCC2;SMARCD3;SMARCE1;ZAP70;ZBTB1;ZMIZ1	9.842e-11	5.287e-07	1.884e-07	all testable terms across Hallmark + KEGG + GO_BP, per query set
DE_genes_fdr05_fc1	GO_BP	Antigen Receptor-Mediated Signaling Pathway (GO:0050851)	39	99	1508	9983	BCL2;BLK;BLNK;BMX;BTN3A1;BTN3A2;BTN3A3;CD247;CD28;CD3D;CD3E;CD3G;CD79A;CD8A;CD8B;EIF2B1;FYN;GATA3;HLA-DPA1;IGHM;IKBKB;ITK;KHDRBS1;LAX1;LCK;LIME1;MEF2C;MS4A1;NFATC2;PDE4B;PDE4D;PLCG1;PLEKHA1;RFTN1;SKAP1;THEMIS;TRBC1;TXK;ZAP70	3.124e-09	8.345e-06	2.99e-06	all testable terms across Hallmark + KEGG + GO_BP, per query set
DE_genes_fdr05_fc1	GO_BP	T Cell Activation (GO:0042110)	31	70	1508	9983	CCR7;CD2;CD247;CD28;CD3D;CD3E;CD3G;CD7;CD74;CD84;CD86;CD8A;CD8B;CEACAM21;CRTAM;DPP4;GATA3;HSPD1;IRF4;ITK;LCK;LFNG;LY9;NLRC3;PIK3R1;RASGRP1;RHOH;SLAMF6;SLAMF7;TRBC1;ZAP70	4.66e-09	8.345e-06	3.823e-06	all testable terms across Hallmark + KEGG + GO_BP, per query set
DE_genes_fdr05_fc1	GO_BP	Regulation Of T Cell Activation (GO:0050863)	22	42	1508	9983	CCL5;CD2;CRTAM;CTSG;HLA-DMA;HLA-DMB;HLA-DOA;HLA-DPA1;HLA-DQA1;HLA-DRA;HLA-DRB4;HSPD1;JAK3;LAX1;LCK;METTL3;PAG1;PRKCQ;SIRPG;SIT1;TBX21;TIGIT	1.825e-08	2.45e-05	1.31e-05	all testable terms across Hallmark + KEGG + GO_BP, per query set
DE_genes_fdr05_fc1	GO_BP	Regulation Of T Cell Differentiation (GO:0045580)	18	35	1508	9983	ACTL6A;ARID1B;CD2;CRTAM;GATA3;METTL3;PHF10;RHOH;SMARCA4;SMARCC1;SMARCC2;SMARCD3;SMARCE1;TBX21;TCF7;ZAP70;ZBTB1;ZMIZ1	5.247e-07	0.0005638	0.0002635	all testable terms across Hallmark + KEGG + GO_BP, per query set
DE_genes_fdr05_fc1	GO_BP	Nucleosome Organization (GO:0034728)	20	45	1508	9983	ASF1A;BAZ1A;DNAJC9;HAT1;HIRA;HMGB2;NAP1L1;NFE2;NPM1;PADI4;RBBP4;SART3;SET;SMARCA4;SMARCC1;SMARCC2;SMARCD3;SMARCE1;SSRP1;SUPT16H	2.39e-06	0.00214	0.0008343	all testable terms across Hallmark + KEGG + GO_BP, per query set
DE_genes_fdr05_fc1	GO_BP	protein-DNA Complex Disassembly (GO:0032986)	10	14	1508	9983	MYC;NFE2;SET;SMARCA4;SMARCC1;SMARCC2;SMARCD3;SMARCE1;SSRP1;SUPT16H	3.359e-06	0.002339	0.001	all testable terms across Hallmark + KEGG + GO_BP, per query set
DE_genes_fdr05_fc1	GO_BP	T Cell Receptor Signaling Pathway (GO:0050852)	27	73	1508	9983	BTN3A1;BTN3A2;BTN3A3;CD247;CD28;CD3D;CD3E;CD3G;CD8A;CD8B;EIF2B1;FYN;GATA3;HLA-DPA1;IKBKB;ITK;KHDRBS1;LCK;PDE4B;PDE4D;PLCG1;RFTN1;SKAP1;THEMIS;TRBC1;TXK;ZAP70	3.483e-06	0.002339	0.001	all testable terms across Hallmark + KEGG + GO_BP, per query set
DE_genes_fdr05_fc1	GO_BP	Immunoglobulin Mediated Immune Response (GO:0016064)	13	23	1508	9983	CD27;CD74;CD81;FCER1A;FCER1G;HLA-DMA;HLA-DMB;HLA-DOA;HLA-DPA1;HLA-DQA1;HLA-DRA;HLA-DRB4;IL2RB	5.241e-06	0.00298	0.001433	all testable terms across Hallmark + KEGG + GO_BP, per query set
DE_genes_fdr05_fc1	GO_BP	Positive Regulation Of Cytokine Production (GO:0001819)	58	217	1508	9983	ANXA1;BATF;BTN3A1;BTN3A2;CCR7;CD2;CD244;CD28;CD40;CD58;CD6;CD74;CD86;CHI3L1;CRTAM;CSF1R;CYP1B1;ELANE;FCER1G;FLOT1;FOXP1;G3BP1;GATA3;GBP5;HLA-DPA1;HPSE;HSPD1;IFIH1;IL16;IL18R1;IL1R1;IL23A;IRF4;ITK;JAK2;LTB;LY9;MAPK14;MMP8;ORM1;OSM;PDE4B;PDE4D;PRKCQ;PTGER4;RASGRP1;RFTN1;RORA;SLAMF1;SLAMF6;SLC11A1;SPTBN1;SULF2;TIGIT;TLR5;TMED10;TRAF3;TXK	5.547e-06	0.00298	0.001448	all testable terms across Hallmark + KEGG + GO_BP, per query set

gene_list	library	term	k_ overlap	K_term_ size	n_ query	N_ universe	genes	p_raw	p_adj_within_ library	p_adj	fdr_family
DE_ genes_ fdr05_fc1	GO_ BP	Defense Response To Bacterium (GO:0042742)	33	101	1508	9983	ANXA3;CAMP;CTSG;DEFA4;ELANE; FCER1G;GBP1;GBP4;GNLY;HMGB2;IGHM; IL23A;LCN2;LTF;MPEG1;MPO;NAIP; NLRC4;NLRP1;OAS2;OAS3;PGLYRP1;PI3; RNASE2;RNASE3;RNASE6;RPS19;S100A12; S100A8;SELP;SLC11A1;SLPI;SPN	7.07e-06	0.003453	0.001691	all testable terms across Hallmark + KEGG + GO_BP, per query set
DE_ genes_ fdr05_fc1	GO_ BP	Positive Regulation Of Lymphocyte Proliferation (GO:0050671)	21	52	1508	9983	ANXA1;BCL2;BST1;CCL5;CD1D;CD28; CD3E;CD40;CD6;CD74;CD81;CD86;FCRL3; GPR183;HLA-DMB;HLA-DPA1;IL23A;JAK2; MEF2C;NFATC2;TNFSF13B	8.595e-06	0.003848	0.001928	all testable terms across Hallmark + KEGG + GO_BP, per query set
DE_ genes_ fdr05_fc1	GO_ BP	Positive Regulation Of T Cell Differentiation (GO:0045582)	19	46	1508	9983	ACTL6A;ARID1B;BCL6;CR1;GATA3; HLA-DRA;IL23A;LEF1;PHF10;RHOH; SMARCA4;SMARCC1;SMARCC2; SMARCD3;SMARCE1;VNN1;ZAP70;ZBTB1; ZMIZ1	1.564e-05	0.005754	0.003075	all testable terms across Hallmark + KEGG + GO_BP, per query set
DE_ genes_ fdr05_fc1	GO_ BP	Nucleosome Disassembly (GO:0006337)	9	13	1508	9983	NFE2;SET;SMARCA4;SMARCC1;SMARCC2; SMARCD3;SMARCE1;SSRP1;SUPT16H	1.607e-05	0.005754	0.003075	all testable terms across Hallmark + KEGG + GO_BP, per query set
DE_ genes_ fdr05_fc1	GO_ BP	Peptide Antigen Assembly With MHC Protein Complex (GO:0002501)	9	13	1508	9983	HLA-DMA;HLA-DMB;HLA-DOA;HLA-DPA1; HLA-DQA1;HLA-DRA;HLA-DRB4;PDIA3; TAPBPL	1.607e-05	0.005754	0.003075	all testable terms across Hallmark + KEGG + GO_BP, per query set
DE_ genes_ fdr05_fc1	GO_ BP	Positive Regulation Of Lymphocyte Differentiation (GO:0045621)	15	32	1508	9983	ACTL6A;ARID1B;CD27;GATA3;PHF10; RHOH;SMARCA4;SMARCC1;SMARCC2; SMARCD3;SMARCE1;XRCC6;ZAP70; ZBTB1;ZMIZ1	2.008e-05	0.006741	0.003719	all testable terms across Hallmark + KEGG + GO_BP, per query set
DE_ genes_ fdr05_fc1	GO_ BP	Antibacterial Humoral Response (GO:0019731)	10	17	1508	9983	CAMP;CTSG;DEFA4;IGHM;LTF;PI3; RNASE2;RNASE3;RNASE6;SLPI	4.215e-05	0.01294	0.007333	all testable terms across Hallmark + KEGG + GO_BP, per query set
DE_ genes_ fdr05_fc1	GO_ BP	MHC Class II Protein Complex Assembly (GO:0002399)	7	9	1508	9983	HLA-DMA;HLA-DMB;HLA-DOA;HLA-DPA1; HLA-DQA1;HLA-DRA;HLA-DRB4	4.816e-05	0.01294	0.007681	all testable terms across Hallmark + KEGG + GO_BP, per query set
DE_ genes_ fdr05_fc1	GO_ BP	T-helper Cell Lineage Commitment (GO:0002295)	7	9	1508	9983	IRF4;JAK1;LY9;SLAMF6;SOCS3;SPN;TBX21	4.816e-05	0.01294	0.007681	all testable terms across Hallmark + KEGG + GO_BP, per query set
DE_ genes_ fdr05_fc1	GO_ BP	Peptide Antigen Assembly With MHC Class II Protein Complex (GO:0002503)	7	9	1508	9983	HLA-DMA;HLA-DMB;HLA-DOA;HLA-DPA1; HLA-DQA1;HLA-DRA;HLA-DRB4	4.816e-05	0.01294	0.007681	all testable terms across Hallmark + KEGG + GO_BP, per query set
DE_ genes_ fdr05_fc1	GO_ BP	Defense Response To Gram-negative Bacterium (GO:0050829)	15	35	1508	9983	CAMP;CTSG;DEFA4;ELANE;HMGB2; IGHM;IL23A;LTF;MPEG1;RNASE2;RNASE3; RNASE6;RPS19;SELP;SLC11A1	7.363e-05	0.01883	0.01143	all testable terms across Hallmark + KEGG + GO_BP, per query set
DE_ genes_ fdr05_fc1	GO_ BP	Nucleosome Assembly (GO:0006334)	13	28	1508	9983	ASF1A;BAZ1A;DNAJC9;HAT1;HIRA; HMGB2;NAP1L1;NPM1;PADI4;RBBP4; SART3;SSRP1;SUPT16H	8.171e-05	0.01995	0.01174	all testable terms across Hallmark + KEGG + GO_BP, per query set
DE_ genes_ fdr05_fc1	GO_ BP	B Cell Receptor Signaling Pathway (GO:0050853)	14	32	1508	9983	BCL2;BLK;BLNK;BMX;CD79A;IGHM;ITK; LCK;LIME1;MEF2C;MS4A1;NFATC2; PLEKHA1;RFTN1	9.746e-05	0.02149	0.01332	all testable terms across Hallmark + KEGG + GO_BP, per query set
DE_ genes_ fdr05_fc1	GO_ BP	Positive Regulation Of Leukocyte Cell-Cell Adhesion (GO:1903039)	14	32	1508	9983	ETS1;FUT7;HLA-DMA;HLA-DMB; HLA-DOA;HLA-DPA1;HLA-DQA1; HLA-DRA;HLA-DRB4;HSPD1;LCK;PRKCQ; SIRPG;SKAP1	9.746e-05	0.02149	0.01332	all testable terms across Hallmark + KEGG + GO_BP, per query set

gene_list	library	term	k_overlap	K_term_size	n_query	N_universe	genes	p_raw	p_adj_within_library	p_adj	fdr_family
DE_genes_fdr05_fc1	GO_BP	Inflammatory Response (GO:0006954)	40	147	1508	9983	ADM;ADORA3;ANXA1;BLNK;C3AR1;CCL5;CCR3;CCR5;CCR7;CD40;CD96;CHI3L1;CSF1R;FUT7;GATA3;GPR68;HDAC4;HNRNPA0;HP;IKBKB;KLRG1;LTB4R;LY75;NAIP;NFATC3;NFX1;NLRC4;ORM1;PPBP;PROK2;PTGDR;PTGER4;PXK;S100A8;SLC11A1;THBS1;TLR5;TNFAIP6;TPST1;VNN1	0.0001	0.02149	0.01335	all testable terms across Hallmark + KEGG + GO_BP, per query set
DE_genes_fdr05_fc1	GO_BP	Antimicrobial Humoral Response (GO:0019730)	15	37	1508	9983	CAMP;CTSG;DEFA4;GNLY;IGHM;LTF;PGLYRP1;PI3;PPBP;RNASE3;RNASE6;RPS19;S100A12;SLC11A1;SLPI	0.0001576	0.03114	0.02011	all testable terms across Hallmark + KEGG + GO_BP, per query set
DE_genes_fdr05_fc1	GO_BP	Antigen Processing And Presentation Of Exogenous Peptide Antigen Via MHC Class II (GO:0019886)	9	16	1508	9983	CTSD;FCER1G;HLA-DMA;HLA-DMB;HLA-DOA;HLA-DPA1;HLA-DQA1;HLA-DRA;HLA-DRB4	0.0001668	0.03114	0.02011	all testable terms across Hallmark + KEGG + GO_BP, per query set
DE_genes_fdr05_fc1	GO_BP	Antigen Processing And Presentation Of Peptide Antigen Via MHC Class II (GO:0002495)	9	16	1508	9983	CTSD;FCER1G;HLA-DMA;HLA-DMB;HLA-DOA;HLA-DPA1;HLA-DQA1;HLA-DRA;HLA-DRB4	0.0001668	0.03114	0.02011	all testable terms across Hallmark + KEGG + GO_BP, per query set
DE_genes_fdr05_fc1	GO_BP	Lymphocyte Migration (GO:0072676)	8	13	1508	9983	CCL5;CCR6;CRTAM;GATA3;KLF5;S1PR1;TBX21;ZAP70	0.0001681	0.03114	0.02011	all testable terms across Hallmark + KEGG + GO_BP, per query set
DE_genes_fdr05_fc1	GO_BP	Positive Regulation Of Type II Interferon Production (GO:0032729)	17	46	1508	9983	BTN3A2;CD2;CD244;CRTAM;HLA-DPA1;HSPD1;IL18R1;IL1R1;IL23A;JAK2;PDE4B;PDE4D;RASGRP1;SLAMF1;SLAMF6;SLC11A1;TXK	0.0002243	0.04017	0.02629	all testable terms across Hallmark + KEGG + GO_BP, per query set
DE_genes_fdr05_fc1	GO_BP	Regulation Of Type II Interferon Production (GO:0032649)	21	64	1508	9983	BTN3A1;BTN3A2;CCR7;CD2;CD244;CR1;CRTAM;HLA-DPA1;HSPD1;IL18R1;IL1R1;IL23A;JAK2;PDE4B;PDE4D;PRNP;RASGRP1;SLAMF1;SLAMF6;SLC11A1;TXK	0.0002985	0.05173	0.03361	all testable terms across Hallmark + KEGG + GO_BP, per query set
DE_genes_fdr05_fc1	GO_BP	Immunoglobulin Production Involved In Immunoglobulin-Mediated Immune Response (GO:0002381)	7	11	1508	9983	HLA-DMA;HLA-DMB;HLA-DOA;HLA-DPA1;HLA-DQA1;HLA-DRA;HLA-DRB4	0.0003335	0.05598	0.03682	all testable terms across Hallmark + KEGG + GO_BP, per query set
DE_genes_fdr05_fc1	GO_BP	T-helper 17 Cell Lineage Commitment (GO:0072540)	5	6	1508	9983	IRF4;JAK1;LY9;SLAMF6;SOCS3	0.0004103	0.06386	0.04388	all testable terms across Hallmark + KEGG + GO_BP, per query set
DE_genes_fdr05_fc1	GO_BP	protein-DNA Complex Assembly (GO:0065004)	13	32	1508	9983	ASF1A;BAZ1A;DNAJC9;HAT1;HIRA;HMGB2;NAP1L1;NPM1;PADI4;RBBP4;SART3;SSRP1;SUPT16H	0.00042	0.06386	0.04388	all testable terms across Hallmark + KEGG + GO_BP, per query set
DE_genes_fdr05_fc1	GO_BP	Negative Regulation Of Viral Genome Replication (GO:0045071)	16	44	1508	9983	APOBEC3B;APOBEC3F;CCL5;IFIH1;IFIT1;ILF3;LTF;MORC2;MPHOSPH8;MX1;OAS2;OAS3;PLSCR1;SLPI;SRPK1;SRPK2	0.000422	0.06386	0.04388	all testable terms across Hallmark + KEGG + GO_BP, per query set
DE_genes_fdr05_fc1	GO_BP	Antigen Processing And Presentation Of Exogenous Peptide Antigen (GO:0002478)	10	21	1508	9983	CTSD;FCER1G;HLA-DMA;HLA-DMB;HLA-DOA;HLA-DPA1;HLA-DQA1;HLA-DRA;HLA-DRB4;MFSD6	0.0004279	0.06386	0.04388	all testable terms across Hallmark + KEGG + GO_BP, per query set
DE_genes_fdr05_fc1	Hallmark	Allograft Rejection	61	156	1508	9983	ABCE1;BCAT1;CAPG;CCL5;CCND2;CCR5;CD1D;CD2;CD247;CD28;CD3D;CD3E;CD3G;CD40;CD7;CD74;CD79A;CD86;CD8A;CD8B;CD96;CRTAM;EIF3A;EIF3D;ELANE;ETS1;GZMA;GZMB;HLA-DMA;HLA-DMB;HLA-DOA;HLA-DQA1;HLA-DRA;IKBKB;IL16;IL18RAP;IL27RA;IL2RB;IL4R;IRF4;IRF8;ITK;JAK2;KLRD1;LCK;LTB;LY75;LY86;MAP4K1;MMP9;MTIF2;NPM1;PRF1;RPS19;SIT1;STAT4;TAP2;TIMP1;TPD52;TRAT1;ZAP70	1.666e-13	8.329e-12	4.782e-10	all testable terms across Hallmark + KEGG + GO_BP, per query set

gene_list	library	term	k_overlap	K_term_size	n_query	N_universe	genes	p_raw	p_adj_within_library	p_adj	fdr_family
DE_genes_fdr05_fc1	Hallmark	IL-2/STAT5 Signaling	50	162	1508	9983	AHNAK;ALCAM;ARL4A;BATF;BCL2;CAPG;CCND2;CD81;CD86;CDC42SE2;CDKN1C;CKAP4;CST7;DHRS3;EOMES;FGL2;FLT3LG;FURIN;GBP4;GSTO1;HOPX;IKZF2;IL10RA;IL18R1;IL1R2;IL2RB;IL4R;IRF4;IRF8;ITGA6;LRIG1;LTB;MYC;NFIL3;PLSCR1;PRKCH;PRNP;PTCH1;RHOH;RORA;RRAGD;SELP;SLC39A8;SMPDL3A;ST3GAL4;SWAP70;TGM2;TIAM1;UMPS;WLS	2.521e-07	6.303e-06	0.0001448	all testable terms across Hallmark + KEGG + GO_BP, per query set
DE_genes_fdr05_fc1	Hallmark	Interferon Gamma Response	52	175	1508	9983	ARL4A;AUTS2;BANK1;BPGM;CCL5;CD40;CD74;CD86;DDX60;EIF4E3;FCGR1A;FGL2;GBP4;GPR18;GZMA;HLA-DMA;HLA-DQA1;IDO1;IFI27;IFIH1;IFIT1;IFIT2;IFIT3;IL10RA;IL2RB;IL4R;IRF4;IRF8;ISOC1;ITGB7;JAK2;LYSMD2;METTL7B;MTHFD2;MX1;NUP93;OAS2;OAS3;PARP14;PDE4B;PLSCR1;SAMHD1;SELP;SLAMF7;SOCS3;ST3GAL5;STAT4;TNFAIP2;TNFAIP6;UBE2L6;UPP1;XAF1	5.507e-07	9.178e-06	0.0002635	all testable terms across Hallmark + KEGG + GO_BP, per query set
DE_genes_fdr05_fc1	Hallmark	Myc Targets V1	46	175	1508	9983	ABCE1;APEX1;CCT4;CUL1;DDX18;DDX21;DHX15;EIF3D;FBL;G3BP1;GOT2;HNRNPA1;HNRNPA3;HNRNPR;HSP90AB1;HSPD1;IMPDH2;LDHA;MCM7;MRPL9;MRPS18B;MYC;NAP1L1;NCBP2;NHP2;NOLC1;NPM1;POLD2;PSMC6;PWP1;RPL14;RPL18;RPL22;RPS5;RRM1;RSL1D1;SET;SMARCC1;SNRPA1;SRM;SRPK1;SYNCRIP;TFDP1;TRIM28;UBA2;XRCC6	7.841e-05	0.0009801	0.01174	all testable terms across Hallmark + KEGG + GO_BP, per query set
DE_genes_fdr05_fc1	Hallmark	Complement	39	148	1508	9983	ADAM9;APOBEC3F;CASP5;CBLB;CCL5;CD55;CD59;CLU;CR1;CTSD;CTSO;DOCK10;DOCK9;DPP4;DUSP5;DUSP6;DYRK2;ERAP2;F5;FCER1G;FYN;GATA3;GP9;GZMA;GZMB;GZMK;ITGAM;JAK2;LCK;LIPA;LTF;MMP8;PLSCR1;RASGRP1;S100A12;SERPINB2;SH2B3;SPOCK2;TIMP1	0.0002524	0.002524	0.02899	all testable terms across Hallmark + KEGG + GO_BP, per query set
DE_genes_fdr05_fc1	KEGG	Hematopoietic cell lineage	38	70	1508	9983	CD1D;CD2;CD24;CD3D;CD3E;CD3G;CD5;CD55;CD59;CD7;CD8A;CD8B;CR1;CSF1R;FCGR1A;FLT3LG;GP9;HLA-DMA;HLA-DMB;HLA-DOA;HLA-DPA1;HLA-DPB1;HLA-DQA1;HLA-DQB1;HLA-DRA;HLA-DRB4;IL11RA;IL1R1;IL1R2;IL4R;IL7R;ITGA2B;ITGA4;ITGA6;ITGAM;ITGB3;MME;MS4A1	2.589e-14	8.285e-12	1.487e-10	all testable terms across Hallmark + KEGG + GO_BP, per query set
DE_genes_fdr05_fc1	KEGG	Th1 and Th2 cell differentiation	33	73	1508	9983	CD247;CD3D;CD3E;CD3G;GATA3;HLA-DMA;HLA-DMB;HLA-DOA;HLA-DPA1;HLA-DPB1;HLA-DQA1;HLA-DQB1;HLA-DRA;HLA-DRB4;IKBKB;IL2RB;IL4R;JAK1;JAK2;JAK3;LCK;MAF;MAML2;MAPK14;NFATC2;NFATC3;PLCG1;PPP3CC;PRKCQ;RUNX3;STAT4;TBX21;ZAP70	7.704e-10	1.233e-07	1.106e-06	all testable terms across Hallmark + KEGG + GO_BP, per query set
DE_genes_fdr05_fc1	KEGG	Th17 cell differentiation	36	86	1508	9983	CD247;CD3D;CD3E;CD3G;GATA3;HLA-DMA;HLA-DMB;HLA-DOA;HLA-DPA1;HLA-DPB1;HLA-DQA1;HLA-DQB1;HLA-DRA;HLA-DRB4;HSP90AB1;IKBKB;IL1R1;IL23A;IL27RA;IL2RB;IL4R;IRF4;JAK1;JAK2;JAK3;LCK;MAPK14;NFATC2;NFATC3;PLCG1;PPP3CC;PRKCQ;RORA;SMAD3;TBX21;ZAP70	1.788e-09	1.908e-07	2.054e-06	all testable terms across Hallmark + KEGG + GO_BP, per query set
DE_genes_fdr05_fc1	KEGG	Asthma	13	17	1508	9983	CD40;FCER1A;FCER1G;HLA-DMA;HLA-DMB;HLA-DOA;HLA-DPA1;HLA-DPB1;HLA-DQA1;HLA-DQB1;HLA-DRA;HLA-DRB4;RNASE3	2.668e-08	2.134e-06	1.702e-05	all testable terms across Hallmark + KEGG + GO_BP, per query set

gene_list	library	term	k_overlap	K_term_size	n_query	N_universe	genes	p_raw	p_adj_within_library	p_adj	fdr_family
DE_genes_fdr05_fc1	KEGG	Inflammatory bowel disease	20	43	1508	9983	GATA3;HLA-DMA;HLA-DMB;HLA-DOA;HLA-DPA1;HLA-DPB1;HLA-DQA1;HLA-DQB1;HLA-DRA;HLA-DRB4;IL18R1;IL18RAP;IL23A;IL4R;MAF;RORA;SMAD3;STAT4;TBX21;TLR5	9.816e-07	6.109e-05	0.0004336	all testable terms across Hallmark + KEGG + GO_BP, per query set
DE_genes_fdr05_fc1	KEGG	Staphylococcus aureus infection	19	40	1508	9983	C1QB;C3AR1;CAMP;CFD;DEFA4;FCAR;FCGR1A;HLA-DMA;HLA-DMB;HLA-DOA;HLA-DPA1;HLA-DPB1;HLA-DQA1;HLA-DQB1;HLA-DRA;HLA-DRB4;ITGAM;KRT23;SELP	1.227e-06	6.109e-05	0.0005033	all testable terms across Hallmark + KEGG + GO_BP, per query set
DE_genes_fdr05_fc1	KEGG	Graft-versus-host disease	14	24	1508	9983	CD28;CD86;GZMB;HLA-DMA;HLA-DMB;HLA-DOA;HLA-DPA1;HLA-DPB1;HLA-DQA1;HLA-DQB1;HLA-DRA;HLA-DRB4;KLRD1;PRF1	1.336e-06	6.109e-05	0.0005116	all testable terms across Hallmark + KEGG + GO_BP, per query set
DE_genes_fdr05_fc1	KEGG	Allograft rejection	14	25	1508	9983	CD28;CD40;CD86;GZMB;HLA-DMA;HLA-DMB;HLA-DOA;HLA-DPA1;HLA-DPB1;HLA-DQA1;HLA-DQB1;HLA-DRA;HLA-DRB4;PRF1	2.615e-06	9.299e-05	0.0008343	all testable terms across Hallmark + KEGG + GO_BP, per query set
DE_genes_fdr05_fc1	KEGG	Autoimmune thyroid disease	14	25	1508	9983	CD28;CD40;CD86;GZMB;HLA-DMA;HLA-DMB;HLA-DOA;HLA-DPA1;HLA-DPB1;HLA-DQA1;HLA-DQB1;HLA-DRA;HLA-DRB4;PRF1	2.615e-06	9.299e-05	0.0008343	all testable terms across Hallmark + KEGG + GO_BP, per query set
DE_genes_fdr05_fc1	KEGG	Systemic lupus erythematosus	16	33	1508	9983	C1QB;CD28;CD40;CD86;CTSG;ELANE;FCGR1A;HLA-DMA;HLA-DMB;HLA-DOA;HLA-DPA1;HLA-DPB1;HLA-DQA1;HLA-DQB1;HLA-DRA;HLA-DRB4	6.134e-06	0.0001963	0.001531	all testable terms across Hallmark + KEGG + GO_BP, per query set
DE_genes_fdr05_fc1	KEGG	Type I diabetes mellitus	14	27	1508	9983	CD28;CD86;GZMB;HLA-DMA;HLA-DMB;HLA-DOA;HLA-DPA1;HLA-DPB1;HLA-DQA1;HLA-DQB1;HLA-DRA;HLA-DRB4;HSPD1;PRF1	8.73e-06	0.000254	0.001928	all testable terms across Hallmark + KEGG + GO_BP, per query set
DE_genes_fdr05_fc1	KEGG	Intestinal immune network for IgA production	15	31	1508	9983	CD28;CD40;CD86;HLA-DMA;HLA-DMB;HLA-DOA;HLA-DPA1;HLA-DPB1;HLA-DQA1;HLA-DQB1;HLA-DRA;HLA-DRB4;ITGA4;ITGB7;TNFSF13B	1.239e-05	0.0003303	0.002634	all testable terms across Hallmark + KEGG + GO_BP, per query set
DE_genes_fdr05_fc1	KEGG	Primary immunodeficiency	14	30	1508	9983	ADA;BLNK;CD3D;CD3E;CD40;CD79A;CD8A;CD8B;IL7R;JAK3;LCK;RFX5;TAP2;ZAP70	4.048e-05	0.0009963	0.007263	all testable terms across Hallmark + KEGG + GO_BP, per query set
DE_genes_fdr05_fc1	KEGG	Cell adhesion molecules	27	85	1508	9983	ALCAM;CD2;CD28;CD40;CD58;CD6;CD86;CD8A;CD8B;HLA-DMA;HLA-DMB;HLA-DOA;HLA-DPA1;HLA-DPB1;HLA-DQA1;HLA-DQB1;HLA-DRA;HLA-DRB4;ICAM2;ITGA4;ITGA6;ITGAM;ITGB7;NTNG2;SELP;SPN;TIGIT	8.181e-05	0.00187	0.01174	all testable terms across Hallmark + KEGG + GO_BP, per query set
DE_genes_fdr05_fc1	KEGG	Antigen processing and presentation	17	45	1508	9983	CD74;CD8A;CD8B;HLA-DMA;HLA-DMB;HLA-DOA;HLA-DPA1;HLA-DPB1;HLA-DQA1;HLA-DQB1;HLA-DRA;HLA-DRB4;HSP90AB1;KLRD1;PDIA3;RFX5;TAP2	0.0001643	0.003505	0.02011	all testable terms across Hallmark + KEGG + GO_BP, per query set

Table S5. FDR-significant over-representation terms for the 10-gene locked signature. **No term reached FDR $p <$ 0.05 in any library.** The table is therefore empty; it has the same columns as Tables S3 and S4.

Table S6. Cross-platform feature-availability audit of all 11,222 discovery (GSE65682, HG-U219/GPL13667) genes against the validation cohort's (GSE57065, HG-U133 Plus 2.0/GPL570) gene-level matrix, run before any differential expression or signature selection (Methods 4.3). 9983 genes (89.0%) were transportable and 1239 (11.0%) were not. Listed here are the 1239 genes that were not; the 9983 that were are the universe of Table S1, and all 11,222 audited genes are in the data workbook, Supplementary File S3.

feature	source_coverage	target_coverage	present_in_target	verdict	note
AAA1	1	0	False	absent	not supplied by the target cohort
ABHD14A	1	0	False	absent	not supplied by the target cohort
ABP1	1	0	False	absent	not supplied by the target cohort
ACBD7	1	0	False	absent	not supplied by the target cohort
ACCN4	1	0	False	absent	not supplied by the target cohort
ACPL2	1	0	False	absent	not supplied by the target cohort
ACTG1	1	0	False	absent	not supplied by the target cohort
ADC	1	0	False	absent	not supplied by the target cohort
AGAP5	1	0	False	absent	not supplied by the target cohort
AGXT2L2	1	0	False	absent	not supplied by the target cohort
AK3L1	1	0	False	absent	not supplied by the target cohort
AMAC1	1	0	False	absent	not supplied by the target cohort
AMAC1L2	1	0	False	absent	not supplied by the target cohort
AMDHD2	1	0	False	absent	not supplied by the target cohort
AMH	1	0	False	absent	not supplied by the target cohort
AMIGO3	1	0	False	absent	not supplied by the target cohort
AMY2B	1	0	False	absent	not supplied by the target cohort
ANAPC1	1	0	False	absent	not supplied by the target cohort
ANKK1	1	0	False	absent	not supplied by the target cohort
ANKRD20A5	1	0	False	absent	not supplied by the target cohort
ANKRD34B	1	0	False	absent	not supplied by the target cohort
ANKRD42	1	0	False	absent	not supplied by the target cohort
ANKRD57	1	0	False	absent	not supplied by the target cohort
ANKRD58	1	0	False	absent	not supplied by the target cohort
AP3S2	1	0	False	absent	not supplied by the target cohort
APBB3	1	0	False	absent	not supplied by the target cohort
APOB48R	1	0	False	absent	not supplied by the target cohort
APOBEC3A	1	0	False	absent	not supplied by the target cohort
APOBEC3H	1	0	False	absent	not supplied by the target cohort
APOC4	1	0	False	absent	not supplied by the target cohort
AQP7	1	0	False	absent	not supplied by the target cohort
ARAP2	1	0	False	absent	not supplied by the target cohort
ARHGAP15	1	0	False	absent	not supplied by the target cohort
ARL17A	1	0	False	absent	not supplied by the target cohort
ARL5A	1	0	False	absent	not supplied by the target cohort
ARRDC5	1	0	False	absent	not supplied by the target cohort
ASTL	1	0	False	absent	not supplied by the target cohort
ATP1A3	1	0	False	absent	not supplied by the target cohort
ATP6V1F	1	0	False	absent	not supplied by the target cohort
BAT1	1	0	False	absent	not supplied by the target cohort
BAT2	1	0	False	absent	not supplied by the target cohort
BAT2L1	1	0	False	absent	not supplied by the target cohort
BAT2L2	1	0	False	absent	not supplied by the target cohort
BAT3	1	0	False	absent	not supplied by the target cohort
BAT4	1	0	False	absent	not supplied by the target cohort
BAT5	1	0	False	absent	not supplied by the target cohort
BBC3	1	0	False	absent	not supplied by the target cohort
BCL3	1	0	False	absent	not supplied by the target cohort
BMI1	1	0	False	absent	not supplied by the target cohort
BOD1L	1	0	False	absent	not supplied by the target cohort
BPI	1	0	False	absent	not supplied by the target cohort
BRP44	1	0	False	absent	not supplied by the target cohort
BRP44L	1	0	False	absent	not supplied by the target cohort
BSCL2	1	0	False	absent	not supplied by the target cohort
BTBD12	1	0	False	absent	not supplied by the target cohort
BTN2A3	1	0	False	absent	not supplied by the target cohort
C10orf118	1	0	False	absent	not supplied by the target cohort
C10orf119	1	0	False	absent	not supplied by the target cohort
C10orf122	1	0	False	absent	not supplied by the target cohort
C10orf125	1	0	False	absent	not supplied by the target cohort
C10orf137	1	0	False	absent	not supplied by the target cohort
C10orf18	1	0	False	absent	not supplied by the target cohort
C10orf26	1	0	False	absent	not supplied by the target cohort
C10orf27	1	0	False	absent	not supplied by the target cohort
C10orf28	1	0	False	absent	not supplied by the target cohort
C10orf4	1	0	False	absent	not supplied by the target cohort
C10orf46	1	0	False	absent	not supplied by the target cohort
C10orf47	1	0	False	absent	not supplied by the target cohort
C10orf57	1	0	False	absent	not supplied by the target cohort
C10orf58	1	0	False	absent	not supplied by the target cohort
C10orf78	1	0	False	absent	not supplied by the target cohort
C10orf84	1	0	False	absent	not supplied by the target cohort
C11orf10	1	0	False	absent	not supplied by the target cohort
C11orf17	1	0	False	absent	not supplied by the target cohort
C11orf2	1	0	False	absent	not supplied by the target cohort
C11orf35	1	0	False	absent	not supplied by the target cohort

feature	source_coverage	target_coverage	present_in_target	verdict	note
C11orf46	1	0	False	absent	not supplied by the target cohort
C11orf48	1	0	False	absent	not supplied by the target cohort
C11orf51	1	0	False	absent	not supplied by the target cohort
C11orf59	1	0	False	absent	not supplied by the target cohort
C11orf61	1	0	False	absent	not supplied by the target cohort
C11orf67	1	0	False	absent	not supplied by the target cohort
C11orf75	1	0	False	absent	not supplied by the target cohort
C11orf82	1	0	False	absent	not supplied by the target cohort
C11orf90	1	0	False	absent	not supplied by the target cohort
C12orf11	1	0	False	absent	not supplied by the target cohort
C12orf23	1	0	False	absent	not supplied by the target cohort
C12orf24	1	0	False	absent	not supplied by the target cohort
C12orf26	1	0	False	absent	not supplied by the target cohort
C12orf32	1	0	False	absent	not supplied by the target cohort
C12orf35	1	0	False	absent	not supplied by the target cohort
C12orf39	1	0	False	absent	not supplied by the target cohort
C12orf41	1	0	False	absent	not supplied by the target cohort
C12orf44	1	0	False	absent	not supplied by the target cohort
C12orf51	1	0	False	absent	not supplied by the target cohort
C12orf52	1	0	False	absent	not supplied by the target cohort
C12orf53	1	0	False	absent	not supplied by the target cohort
C12orf62	1	0	False	absent	not supplied by the target cohort
C13orf15	1	0	False	absent	not supplied by the target cohort
C13orf18	1	0	False	absent	not supplied by the target cohort
C13orf23	1	0	False	absent	not supplied by the target cohort
C13orf27	1	0	False	absent	not supplied by the target cohort
C13orf31	1	0	False	absent	not supplied by the target cohort
C13orf34	1	0	False	absent	not supplied by the target cohort
C13orf37	1	0	False	absent	not supplied by the target cohort
C14orf101	1	0	False	absent	not supplied by the target cohort
C14orf102	1	0	False	absent	not supplied by the target cohort
C14orf106	1	0	False	absent	not supplied by the target cohort
C14orf109	1	0	False	absent	not supplied by the target cohort
C14orf118	1	0	False	absent	not supplied by the target cohort
C14orf126	1	0	False	absent	not supplied by the target cohort
C14orf129	1	0	False	absent	not supplied by the target cohort
C14orf135	1	0	False	absent	not supplied by the target cohort
C14orf138	1	0	False	absent	not supplied by the target cohort
C14orf139	1	0	False	absent	not supplied by the target cohort
C14orf143	1	0	False	absent	not supplied by the target cohort
C14orf147	1	0	False	absent	not supplied by the target cohort
C14orf153	1	0	False	absent	not supplied by the target cohort
C14orf156	1	0	False	absent	not supplied by the target cohort
C14orf181	1	0	False	absent	not supplied by the target cohort
C14orf21	1	0	False	absent	not supplied by the target cohort
C14orf4	1	0	False	absent	not supplied by the target cohort
C14orf43	1	0	False	absent	not supplied by the target cohort
C14orf45	1	0	False	absent	not supplied by the target cohort
C14orf68	1	0	False	absent	not supplied by the target cohort
C15orf17	1	0	False	absent	not supplied by the target cohort
C15orf23	1	0	False	absent	not supplied by the target cohort
C15orf24	1	0	False	absent	not supplied by the target cohort
C15orf29	1	0	False	absent	not supplied by the target cohort
C15orf44	1	0	False	absent	not supplied by the target cohort
C15orf5	1	0	False	absent	not supplied by the target cohort
C15orf58	1	0	False	absent	not supplied by the target cohort
C16orf42	1	0	False	absent	not supplied by the target cohort
C16orf5	1	0	False	absent	not supplied by the target cohort
C16orf53	1	0	False	absent	not supplied by the target cohort
C16orf55	1	0	False	absent	not supplied by the target cohort
C16orf57	1	0	False	absent	not supplied by the target cohort
C16orf61	1	0	False	absent	not supplied by the target cohort
C16orf63	1	0	False	absent	not supplied by the target cohort
C16orf68	1	0	False	absent	not supplied by the target cohort
C16orf7	1	0	False	absent	not supplied by the target cohort
C16orf75	1	0	False	absent	not supplied by the target cohort
C16orf79	1	0	False	absent	not supplied by the target cohort
C16orf88	1	0	False	absent	not supplied by the target cohort
C17orf101	1	0	False	absent	not supplied by the target cohort
C17orf103	1	0	False	absent	not supplied by the target cohort
C17orf108	1	0	False	absent	not supplied by the target cohort
C17orf28	1	0	False	absent	not supplied by the target cohort
C17orf37	1	0	False	absent	not supplied by the target cohort
C17orf39	1	0	False	absent	not supplied by the target cohort
C17orf42	1	0	False	absent	not supplied by the target cohort
C17orf48	1	0	False	absent	not supplied by the target cohort
C17orf49	1	0	False	absent	not supplied by the target cohort
C17orf55	1	0	False	absent	not supplied by the target cohort
C17orf56	1	0	False	absent	not supplied by the target cohort
C17orf60	1	0	False	absent	not supplied by the target cohort
C17orf61	1	0	False	absent	not supplied by the target cohort
C17orf63	1	0	False	absent	not supplied by the target cohort
C17orf68	1	0	False	absent	not supplied by the target cohort
C17orf71	1	0	False	absent	not supplied by the target cohort
C17orf76	1	0	False	absent	not supplied by the target cohort
C17orf79	1	0	False	absent	not supplied by the target cohort

feature	source_coverage	target_coverage	present_in_target	verdict	note
C17orf81	1	0	False	absent	not supplied by the target cohort
C17orf87	1	0	False	absent	not supplied by the target cohort
C17orf90	1	0	False	absent	not supplied by the target cohort
C17orf91	1	0	False	absent	not supplied by the target cohort
C17orf95	1	0	False	absent	not supplied by the target cohort
C18orf1	1	0	False	absent	not supplied by the target cohort
C18orf10	1	0	False	absent	not supplied by the target cohort
C18orf19	1	0	False	absent	not supplied by the target cohort
C18orf22	1	0	False	absent	not supplied by the target cohort
C18orf32	1	0	False	absent	not supplied by the target cohort
C18orf45	1	0	False	absent	not supplied by the target cohort
C18orf49	1	0	False	absent	not supplied by the target cohort
C18orf55	1	0	False	absent	not supplied by the target cohort
C18orf56	1	0	False	absent	not supplied by the target cohort
C19orf2	1	0	False	absent	not supplied by the target cohort
C19orf20	1	0	False	absent	not supplied by the target cohort
C19orf22	1	0	False	absent	not supplied by the target cohort
C19orf28	1	0	False	absent	not supplied by the target cohort
C19orf29	1	0	False	absent	not supplied by the target cohort
C19orf35	1	0	False	absent	not supplied by the target cohort
C19orf38	1	0	False	absent	not supplied by the target cohort
C19orf39	1	0	False	absent	not supplied by the target cohort
C19orf42	1	0	False	absent	not supplied by the target cohort
C19orf48	1	0	False	absent	not supplied by the target cohort
C19orf55	1	0	False	absent	not supplied by the target cohort
C19orf56	1	0	False	absent	not supplied by the target cohort
C19orf59	1	0	False	absent	not supplied by the target cohort
C19orf6	1	0	False	absent	not supplied by the target cohort
C19orf61	1	0	False	absent	not supplied by the target cohort
C19orf62	1	0	False	absent	not supplied by the target cohort
C19orf63	1	0	False	absent	not supplied by the target cohort
C19orf71	1	0	False	absent	not supplied by the target cohort
C19orf77	1	0	False	absent	not supplied by the target cohort
C1QTNF8	1	0	False	absent	not supplied by the target cohort
C1orf103	1	0	False	absent	not supplied by the target cohort
C1orf107	1	0	False	absent	not supplied by the target cohort
C1orf124	1	0	False	absent	not supplied by the target cohort
C1orf128	1	0	False	absent	not supplied by the target cohort
C1orf130	1	0	False	absent	not supplied by the target cohort
C1orf144	1	0	False	absent	not supplied by the target cohort
C1orf151	1	0	False	absent	not supplied by the target cohort
C1orf152	1	0	False	absent	not supplied by the target cohort
C1orf156	1	0	False	absent	not supplied by the target cohort
C1orf170	1	0	False	absent	not supplied by the target cohort
C1orf183	1	0	False	absent	not supplied by the target cohort
C1orf186	1	0	False	absent	not supplied by the target cohort
C1orf212	1	0	False	absent	not supplied by the target cohort
C1orf217	1	0	False	absent	not supplied by the target cohort
C1orf25	1	0	False	absent	not supplied by the target cohort
C1orf26	1	0	False	absent	not supplied by the target cohort
C1orf31	1	0	False	absent	not supplied by the target cohort
C1orf38	1	0	False	absent	not supplied by the target cohort
C1orf55	1	0	False	absent	not supplied by the target cohort
C1orf57	1	0	False	absent	not supplied by the target cohort
C1orf58	1	0	False	absent	not supplied by the target cohort
C1orf59	1	0	False	absent	not supplied by the target cohort
C1orf63	1	0	False	absent	not supplied by the target cohort
C1orf69	1	0	False	absent	not supplied by the target cohort
C1orf70	1	0	False	absent	not supplied by the target cohort
C1orf77	1	0	False	absent	not supplied by the target cohort
C1orf83	1	0	False	absent	not supplied by the target cohort
C1orf9	1	0	False	absent	not supplied by the target cohort
C1orf91	1	0	False	absent	not supplied by the target cohort
C1orf93	1	0	False	absent	not supplied by the target cohort
C1orf96	1	0	False	absent	not supplied by the target cohort
C20orf103	1	0	False	absent	not supplied by the target cohort
C20orf106	1	0	False	absent	not supplied by the target cohort
C20orf108	1	0	False	absent	not supplied by the target cohort
C20orf11	1	0	False	absent	not supplied by the target cohort
C20orf111	1	0	False	absent	not supplied by the target cohort
C20orf112	1	0	False	absent	not supplied by the target cohort
C20orf117	1	0	False	absent	not supplied by the target cohort
C20orf134	1	0	False	absent	not supplied by the target cohort
C20orf160	1	0	False	absent	not supplied by the target cohort
C20orf177	1	0	False	absent	not supplied by the target cohort
C20orf199	1	0	False	absent	not supplied by the target cohort
C20orf200	1	0	False	absent	not supplied by the target cohort
C20orf201	1	0	False	absent	not supplied by the target cohort
C20orf24	1	0	False	absent	not supplied by the target cohort
C20orf29	1	0	False	absent	not supplied by the target cohort
C20orf3	1	0	False	absent	not supplied by the target cohort
C20orf30	1	0	False	absent	not supplied by the target cohort
C20orf4	1	0	False	absent	not supplied by the target cohort
C20orf43	1	0	False	absent	not supplied by the target cohort
C20orf54	1	0	False	absent	not supplied by the target cohort
C20orf69	1	0	False	absent	not supplied by the target cohort

feature	source_coverage	target_coverage	present_in_target	verdict	note
C20orf7	1	0	False	absent	not supplied by the target cohort
C20orf72	1	0	False	absent	not supplied by the target cohort
C20orf94	1	0	False	absent	not supplied by the target cohort
C21orf45	1	0	False	absent	not supplied by the target cohort
C21orf57	1	0	False	absent	not supplied by the target cohort
C21orf63	1	0	False	absent	not supplied by the target cohort
C21orf7	1	0	False	absent	not supplied by the target cohort
C22orf13	1	0	False	absent	not supplied by the target cohort
C22orf25	1	0	False	absent	not supplied by the target cohort
C22orf28	1	0	False	absent	not supplied by the target cohort
C22orf29	1	0	False	absent	not supplied by the target cohort
C22orf30	1	0	False	absent	not supplied by the target cohort
C22orf32	1	0	False	absent	not supplied by the target cohort
C22orf40	1	0	False	absent	not supplied by the target cohort
C22orf9	1	0	False	absent	not supplied by the target cohort
C2CD4D	1	0	False	absent	not supplied by the target cohort
C2orf18	1	0	False	absent	not supplied by the target cohort
C2orf24	1	0	False	absent	not supplied by the target cohort
C2orf28	1	0	False	absent	not supplied by the target cohort
C2orf29	1	0	False	absent	not supplied by the target cohort
C2orf51	1	0	False	absent	not supplied by the target cohort
C2orf53	1	0	False	absent	not supplied by the target cohort
C2orf55	1	0	False	absent	not supplied by the target cohort
C2orf56	1	0	False	absent	not supplied by the target cohort
C2orf60	1	0	False	absent	not supplied by the target cohort
C2orf62	1	0	False	absent	not supplied by the target cohort
C2orf64	1	0	False	absent	not supplied by the target cohort
C2orf7	1	0	False	absent	not supplied by the target cohort
C2orf79	1	0	False	absent	not supplied by the target cohort
C2orf89	1	0	False	absent	not supplied by the target cohort
C3orf1	1	0	False	absent	not supplied by the target cohort
C3orf10	1	0	False	absent	not supplied by the target cohort
C3orf19	1	0	False	absent	not supplied by the target cohort
C3orf21	1	0	False	absent	not supplied by the target cohort
C3orf23	1	0	False	absent	not supplied by the target cohort
C3orf25	1	0	False	absent	not supplied by the target cohort
C3orf31	1	0	False	absent	not supplied by the target cohort
C3orf34	1	0	False	absent	not supplied by the target cohort
C3orf37	1	0	False	absent	not supplied by the target cohort
C3orf39	1	0	False	absent	not supplied by the target cohort
C3orf54	1	0	False	absent	not supplied by the target cohort
C3orf59	1	0	False	absent	not supplied by the target cohort
C3orf63	1	0	False	absent	not supplied by the target cohort
C3orf71	1	0	False	absent	not supplied by the target cohort
C3orf75	1	0	False	absent	not supplied by the target cohort
C4orf14	1	0	False	absent	not supplied by the target cohort
C4orf23	1	0	False	absent	not supplied by the target cohort
C4orf34	1	0	False	absent	not supplied by the target cohort
C4orf41	1	0	False	absent	not supplied by the target cohort
C4orf52	1	0	False	absent	not supplied by the target cohort
C5orf13	1	0	False	absent	not supplied by the target cohort
C5orf25	1	0	False	absent	not supplied by the target cohort
C5orf32	1	0	False	absent	not supplied by the target cohort
C5orf33	1	0	False	absent	not supplied by the target cohort
C5orf36	1	0	False	absent	not supplied by the target cohort
C5orf39	1	0	False	absent	not supplied by the target cohort
C5orf4	1	0	False	absent	not supplied by the target cohort
C5orf41	1	0	False	absent	not supplied by the target cohort
C5orf43	1	0	False	absent	not supplied by the target cohort
C5orf44	1	0	False	absent	not supplied by the target cohort
C5orf53	1	0	False	absent	not supplied by the target cohort
C5orf62	1	0	False	absent	not supplied by the target cohort
C6orf105	1	0	False	absent	not supplied by the target cohort
C6orf108	1	0	False	absent	not supplied by the target cohort
C6orf115	1	0	False	absent	not supplied by the target cohort
C6orf125	1	0	False	absent	not supplied by the target cohort
C6orf129	1	0	False	absent	not supplied by the target cohort
C6orf130	1	0	False	absent	not supplied by the target cohort
C6orf134	1	0	False	absent	not supplied by the target cohort
C6orf142	1	0	False	absent	not supplied by the target cohort
C6orf150	1	0	False	absent	not supplied by the target cohort
C6orf153	1	0	False	absent	not supplied by the target cohort
C6orf162	1	0	False	absent	not supplied by the target cohort
C6orf192	1	0	False	absent	not supplied by the target cohort
C6orf204	1	0	False	absent	not supplied by the target cohort
C6orf26	1	0	False	absent	not supplied by the target cohort
C6orf27	1	0	False	absent	not supplied by the target cohort
C6orf35	1	0	False	absent	not supplied by the target cohort
C6orf54	1	0	False	absent	not supplied by the target cohort
C6orf64	1	0	False	absent	not supplied by the target cohort
C6orf72	1	0	False	absent	not supplied by the target cohort
C6orf81	1	0	False	absent	not supplied by the target cohort
C6orf97	1	0	False	absent	not supplied by the target cohort
C7orf11	1	0	False	absent	not supplied by the target cohort
C7orf16	1	0	False	absent	not supplied by the target cohort
C7orf23	1	0	False	absent	not supplied by the target cohort

feature	source_coverage	target_coverage	present_in_target	verdict	note
C7orf27	1	0	False	absent	not supplied by the target cohort
C7orf29	1	0	False	absent	not supplied by the target cohort
C7orf30	1	0	False	absent	not supplied by the target cohort
C7orf41	1	0	False	absent	not supplied by the target cohort
C7orf42	1	0	False	absent	not supplied by the target cohort
C7orf44	1	0	False	absent	not supplied by the target cohort
C7orf47	1	0	False	absent	not supplied by the target cohort
C7orf52	1	0	False	absent	not supplied by the target cohort
C7orf53	1	0	False	absent	not supplied by the target cohort
C7orf54	1	0	False	absent	not supplied by the target cohort
C7orf59	1	0	False	absent	not supplied by the target cohort
C7orf64	1	0	False	absent	not supplied by the target cohort
C7orf70	1	0	False	absent	not supplied by the target cohort
C7orf72	1	0	False	absent	not supplied by the target cohort
C8orf38	1	0	False	absent	not supplied by the target cohort
C8orf40	1	0	False	absent	not supplied by the target cohort
C8orf41	1	0	False	absent	not supplied by the target cohort
C8orf42	1	0	False	absent	not supplied by the target cohort
C8orf55	1	0	False	absent	not supplied by the target cohort
C8orf73	1	0	False	absent	not supplied by the target cohort
C8orf83	1	0	False	absent	not supplied by the target cohort
C9orf102	1	0	False	absent	not supplied by the target cohort
C9orf103	1	0	False	absent	not supplied by the target cohort
C9orf119	1	0	False	absent	not supplied by the target cohort
C9orf123	1	0	False	absent	not supplied by the target cohort
C9orf140	1	0	False	absent	not supplied by the target cohort
C9orf167	1	0	False	absent	not supplied by the target cohort
C9orf21	1	0	False	absent	not supplied by the target cohort
C9orf23	1	0	False	absent	not supplied by the target cohort
C9orf30	1	0	False	absent	not supplied by the target cohort
C9orf45	1	0	False	absent	not supplied by the target cohort
C9orf46	1	0	False	absent	not supplied by the target cohort
C9orf5	1	0	False	absent	not supplied by the target cohort
C9orf6	1	0	False	absent	not supplied by the target cohort
C9orf7	1	0	False	absent	not supplied by the target cohort
C9orf80	1	0	False	absent	not supplied by the target cohort
C9orf82	1	0	False	absent	not supplied by the target cohort
C9orf86	1	0	False	absent	not supplied by the target cohort
C9orf95	1	0	False	absent	not supplied by the target cohort
CA13	1	0	False	absent	not supplied by the target cohort
CABC1	1	0	False	absent	not supplied by the target cohort
CACNA1A	1	0	False	absent	not supplied by the target cohort
CALM2	1	0	False	absent	not supplied by the target cohort
CALM3	1	0	False	absent	not supplied by the target cohort
CARD17	1	0	False	absent	not supplied by the target cohort
CARD9	1	0	False	absent	not supplied by the target cohort
CASC3	1	0	False	absent	not supplied by the target cohort
CBARA1	1	0	False	absent	not supplied by the target cohort
CBWD5	1	0	False	absent	not supplied by the target cohort
CCDC109A	1	0	False	absent	not supplied by the target cohort
CCDC111	1	0	False	absent	not supplied by the target cohort
CCDC45	1	0	False	absent	not supplied by the target cohort
CCDC48	1	0	False	absent	not supplied by the target cohort
CCDC55	1	0	False	absent	not supplied by the target cohort
CCDC56	1	0	False	absent	not supplied by the target cohort
CCDC72	1	0	False	absent	not supplied by the target cohort
CCDC74A	1	0	False	absent	not supplied by the target cohort
CCDC75	1	0	False	absent	not supplied by the target cohort
CCDC76	1	0	False	absent	not supplied by the target cohort
CCDC90A	1	0	False	absent	not supplied by the target cohort
CCL3	1	0	False	absent	not supplied by the target cohort
CCT3	1	0	False	absent	not supplied by the target cohort
CD164L2	1	0	False	absent	not supplied by the target cohort
CD300E	1	0	False	absent	not supplied by the target cohort
CD302	1	0	False	absent	not supplied by the target cohort
CD68	1	0	False	absent	not supplied by the target cohort
CDH23	1	0	False	absent	not supplied by the target cohort
CDK11A	1	0	False	absent	not supplied by the target cohort
CDK11B	1	0	False	absent	not supplied by the target cohort
CDK3	1	0	False	absent	not supplied by the target cohort
CDR2	1	0	False	absent	not supplied by the target cohort
CDRT4	1	0	False	absent	not supplied by the target cohort
CENPC1	1	0	False	absent	not supplied by the target cohort
CEP110	1	0	False	absent	not supplied by the target cohort
CEP170	1	0	False	absent	not supplied by the target cohort
CES1	1	0	False	absent	not supplied by the target cohort
CHCHD8	1	0	False	absent	not supplied by the target cohort
CHMP4A	1	0	False	absent	not supplied by the target cohort
CHP	1	0	False	absent	not supplied by the target cohort
CKMT1B	1	0	False	absent	not supplied by the target cohort
CLCNKA	1	0	False	absent	not supplied by the target cohort
CLEC3B	1	0	False	absent	not supplied by the target cohort
CLRN2	1	0	False	absent	not supplied by the target cohort
CMAH	1	0	False	absent	not supplied by the target cohort
CNIH	1	0	False	absent	not supplied by the target cohort
CNO	1	0	False	absent	not supplied by the target cohort

feature	source_coverage	target_coverage	present_in_target	verdict	note
COBRA1	1	0	False	absent	not supplied by the target cohort
COPG	1	0	False	absent	not supplied by the target cohort
COX4NB	1	0	False	absent	not supplied by the target cohort
CP110	1	0	False	absent	not supplied by the target cohort
CSAG1	1	0	False	absent	not supplied by the target cohort
CSDA	1	0	False	absent	not supplied by the target cohort
CSRP2BP	1	0	False	absent	not supplied by the target cohort
CTNND1	1	0	False	absent	not supplied by the target cohort
CTPS	1	0	False	absent	not supplied by the target cohort
CTSL1	1	0	False	absent	not supplied by the target cohort
CXorf26	1	0	False	absent	not supplied by the target cohort
CYBASC3	1	0	False	absent	not supplied by the target cohort
CYP2B7P1	1	0	False	absent	not supplied by the target cohort
CYP3A7	1	0	False	absent	not supplied by the target cohort
CYP51A1	1	0	False	absent	not supplied by the target cohort
CYTSA	1	0	False	absent	not supplied by the target cohort
CYTSB	1	0	False	absent	not supplied by the target cohort
CYorf15A	1	0	False	absent	not supplied by the target cohort
CYorf15B	1	0	False	absent	not supplied by the target cohort
DARC	1	0	False	absent	not supplied by the target cohort
DBNL	1	0	False	absent	not supplied by the target cohort
DCI	1	0	False	absent	not supplied by the target cohort
DDR1	1	0	False	absent	not supplied by the target cohort
DDT	1	0	False	absent	not supplied by the target cohort
DDX39	1	0	False	absent	not supplied by the target cohort
DDX47	1	0	False	absent	not supplied by the target cohort
DEFB113	1	0	False	absent	not supplied by the target cohort
DEFB134	1	0	False	absent	not supplied by the target cohort
DEFB4A	1	0	False	absent	not supplied by the target cohort
DEFT1P	1	0	False	absent	not supplied by the target cohort
DGCR6	1	0	False	absent	not supplied by the target cohort
DGCR8	1	0	False	absent	not supplied by the target cohort
DHRS4	1	0	False	absent	not supplied by the target cohort
DHRS4L2	1	0	False	absent	not supplied by the target cohort
DIMT1L	1	0	False	absent	not supplied by the target cohort
DKC1	1	0	False	absent	not supplied by the target cohort
DKFZp761E198	1	0	False	absent	not supplied by the target cohort
DNAJC25	1	0	False	absent	not supplied by the target cohort
DOM3Z	1	0	False	absent	not supplied by the target cohort
DPH3B	1	0	False	absent	not supplied by the target cohort
DTX2	1	0	False	absent	not supplied by the target cohort
DULLARD	1	0	False	absent	not supplied by the target cohort
DUS2L	1	0	False	absent	not supplied by the target cohort
DUSP8	1	0	False	absent	not supplied by the target cohort
EBNA1BP2	1	0	False	absent	not supplied by the target cohort
EEF1B2	1	0	False	absent	not supplied by the target cohort
EEF1G	1	0	False	absent	not supplied by the target cohort
EEF2K	1	0	False	absent	not supplied by the target cohort
EFHA1	1	0	False	absent	not supplied by the target cohort
EIF2C1	1	0	False	absent	not supplied by the target cohort
EIF2C2	1	0	False	absent	not supplied by the target cohort
EIF2C3	1	0	False	absent	not supplied by the target cohort
EIF2C4	1	0	False	absent	not supplied by the target cohort
EIF4A1	1	0	False	absent	not supplied by the target cohort
ELOVL1	1	0	False	absent	not supplied by the target cohort
ENTPD8	1	0	False	absent	not supplied by the target cohort
EPB49	1	0	False	absent	not supplied by the target cohort
EPN2	1	0	False	absent	not supplied by the target cohort
ERVWE1	1	0	False	absent	not supplied by the target cohort
ETV3L	1	0	False	absent	not supplied by the target cohort
EVX2	1	0	False	absent	not supplied by the target cohort
FADS1	1	0	False	absent	not supplied by the target cohort
FAM100A	1	0	False	absent	not supplied by the target cohort
FAM100B	1	0	False	absent	not supplied by the target cohort
FAM105B	1	0	False	absent	not supplied by the target cohort
FAM108B1	1	0	False	absent	not supplied by the target cohort
FAM108C1	1	0	False	absent	not supplied by the target cohort
FAM113A	1	0	False	absent	not supplied by the target cohort
FAM113B	1	0	False	absent	not supplied by the target cohort
FAM115C	1	0	False	absent	not supplied by the target cohort
FAM116A	1	0	False	absent	not supplied by the target cohort
FAM116B	1	0	False	absent	not supplied by the target cohort
FAM119A	1	0	False	absent	not supplied by the target cohort
FAM125B	1	0	False	absent	not supplied by the target cohort
FAM128A	1	0	False	absent	not supplied by the target cohort
FAM128B	1	0	False	absent	not supplied by the target cohort
FAM136A	1	0	False	absent	not supplied by the target cohort
FAM136B	1	0	False	absent	not supplied by the target cohort
FAM151B	1	0	False	absent	not supplied by the target cohort
FAM153B	1	0	False	absent	not supplied by the target cohort
FAM153C	1	0	False	absent	not supplied by the target cohort
FAM158A	1	0	False	absent	not supplied by the target cohort
FAM164A	1	0	False	absent	not supplied by the target cohort
FAM165B	1	0	False	absent	not supplied by the target cohort
FAM176B	1	0	False	absent	not supplied by the target cohort
FAM177A1	1	0	False	absent	not supplied by the target cohort

feature	source_coverage	target_coverage	present_in_target	verdict	note
FAM177B	1	0	False	absent	not supplied by the target cohort
FAM188B	1	0	False	absent	not supplied by the target cohort
FAM18A	1	0	False	absent	not supplied by the target cohort
FAM18B2	1	0	False	absent	not supplied by the target cohort
FAM190B	1	0	False	absent	not supplied by the target cohort
FAM22F	1	0	False	absent	not supplied by the target cohort
FAM36A	1	0	False	absent	not supplied by the target cohort
FAM38A	1	0	False	absent	not supplied by the target cohort
FAM40A	1	0	False	absent	not supplied by the target cohort
FAM48A	1	0	False	absent	not supplied by the target cohort
FAM54B	1	0	False	absent	not supplied by the target cohort
FAM55C	1	0	False	absent	not supplied by the target cohort
FAM57B	1	0	False	absent	not supplied by the target cohort
FAM58B	1	0	False	absent	not supplied by the target cohort
FAM70B	1	0	False	absent	not supplied by the target cohort
FAM78A	1	0	False	absent	not supplied by the target cohort
FAM82A1	1	0	False	absent	not supplied by the target cohort
FAM82A2	1	0	False	absent	not supplied by the target cohort
FAM82B	1	0	False	absent	not supplied by the target cohort
FAM86C	1	0	False	absent	not supplied by the target cohort
FAM89A	1	0	False	absent	not supplied by the target cohort
FBXO41	1	0	False	absent	not supplied by the target cohort
FCGR3A	1	0	False	absent	not supplied by the target cohort
FCRL6	1	0	False	absent	not supplied by the target cohort
FFAR3	1	0	False	absent	not supplied by the target cohort
FKBP1A	1	0	False	absent	not supplied by the target cohort
FLJ10213	1	0	False	absent	not supplied by the target cohort
FLJ10357	1	0	False	absent	not supplied by the target cohort
FLJ14107	1	0	False	absent	not supplied by the target cohort
FLJ35220	1	0	False	absent	not supplied by the target cohort
FLJ36031	1	0	False	absent	not supplied by the target cohort
FLJ36777	1	0	False	absent	not supplied by the target cohort
FLJ40448	1	0	False	absent	not supplied by the target cohort
FLJ42393	1	0	False	absent	not supplied by the target cohort
FLJ44635	1	0	False	absent	not supplied by the target cohort
FLJ44653	1	0	False	absent	not supplied by the target cohort
FLJ45340	1	0	False	absent	not supplied by the target cohort
FLJ45445	1	0	False	absent	not supplied by the target cohort
FNTB	1	0	False	absent	not supplied by the target cohort
FOXD4	1	0	False	absent	not supplied by the target cohort
FOXI2	1	0	False	absent	not supplied by the target cohort
FTSJD1	1	0	False	absent	not supplied by the target cohort
FTSJD2	1	0	False	absent	not supplied by the target cohort
FXC1	1	0	False	absent	not supplied by the target cohort
FZD8	1	0	False	absent	not supplied by the target cohort
GALNT4	1	0	False	absent	not supplied by the target cohort
GALNTL1	1	0	False	absent	not supplied by the target cohort
GALNTL4	1	0	False	absent	not supplied by the target cohort
GBA	1	0	False	absent	not supplied by the target cohort
GBP7	1	0	False	absent	not supplied by the target cohort
GCET2	1	0	False	absent	not supplied by the target cohort
GCFC1	1	0	False	absent	not supplied by the target cohort
GCOM1	1	0	False	absent	not supplied by the target cohort
GDEP	1	0	False	absent	not supplied by the target cohort
GGTA1	1	0	False	absent	not supplied by the target cohort
GIMAP5	1	0	False	absent	not supplied by the target cohort
GLB1	1	0	False	absent	not supplied by the target cohort
GLT25D1	1	0	False	absent	not supplied by the target cohort
GLT25D2	1	0	False	absent	not supplied by the target cohort
GLTPD1	1	0	False	absent	not supplied by the target cohort
GMPPB	1	0	False	absent	not supplied by the target cohort
GNB2L1	1	0	False	absent	not supplied by the target cohort
GOLGA6L7P	1	0	False	absent	not supplied by the target cohort
GPER	1	0	False	absent	not supplied by the target cohort
GPR108	1	0	False	absent	not supplied by the target cohort
GPR141	1	0	False	absent	not supplied by the target cohort
GPR142	1	0	False	absent	not supplied by the target cohort
GPR172A	1	0	False	absent	not supplied by the target cohort
GPR172B	1	0	False	absent	not supplied by the target cohort
GPR44	1	0	False	absent	not supplied by the target cohort
GPR77	1	0	False	absent	not supplied by the target cohort
GPX6	1	0	False	absent	not supplied by the target cohort
GRLF1	1	0	False	absent	not supplied by the target cohort
GSTM2P1	1	0	False	absent	not supplied by the target cohort
GTPBP5	1	0	False	absent	not supplied by the target cohort
H3F3B	1	0	False	absent	not supplied by the target cohort
HARS	1	0	False	absent	not supplied by the target cohort
HAUS4	1	0	False	absent	not supplied by the target cohort
HBCBP	1	0	False	absent	not supplied by the target cohort
HBG1	1	0	False	absent	not supplied by the target cohort
HBXIP	1	0	False	absent	not supplied by the target cohort
HDHD1A	1	0	False	absent	not supplied by the target cohort
HEATR7A	1	0	False	absent	not supplied by the target cohort
HEPN1	1	0	False	absent	not supplied by the target cohort
HERC3	1	0	False	absent	not supplied by the target cohort
HIST1H2AD	1	0	False	absent	not supplied by the target cohort

feature	source_coverage	target_coverage	present_in_target	verdict	note
HIST1H2AG	1	0	False	absent	not supplied by the target cohort
HIST1H2BF	1	0	False	absent	not supplied by the target cohort
HIST1H2BG	1	0	False	absent	not supplied by the target cohort
HIST1H3D	1	0	False	absent	not supplied by the target cohort
HIST1H3H	1	0	False	absent	not supplied by the target cohort
HIST1H4C	1	0	False	absent	not supplied by the target cohort
HIST1H4K	1	0	False	absent	not supplied by the target cohort
HIST2H2AC	1	0	False	absent	not supplied by the target cohort
HIST2H2BF	1	0	False	absent	not supplied by the target cohort
HIST3H2BB	1	0	False	absent	not supplied by the target cohort
HLA-DRB1	1	0	False	absent	not supplied by the target cohort
HLA-DRB5	1	0	False	absent	not supplied by the target cohort
HLA-H	1	0	False	absent	not supplied by the target cohort
HNRNPA1L2	1	0	False	absent	not supplied by the target cohort
HNRNPH2	1	0	False	absent	not supplied by the target cohort
HNRPDL	1	0	False	absent	not supplied by the target cohort
HNRPLL	1	0	False	absent	not supplied by the target cohort
HOXA9	1	0	False	absent	not supplied by the target cohort
HRNBP3	1	0	False	absent	not supplied by the target cohort
HSN2	1	0	False	absent	not supplied by the target cohort
HSP90AA4P	1	0	False	absent	not supplied by the target cohort
HSPA1A	1	0	False	absent	not supplied by the target cohort
HSPA1B	1	0	False	absent	not supplied by the target cohort
HSPA8	1	0	False	absent	not supplied by the target cohort
HSPC159	1	0	False	absent	not supplied by the target cohort
HTR3D	1	0	False	absent	not supplied by the target cohort
IFI30	1	0	False	absent	not supplied by the target cohort
IFIT1B	1	0	False	absent	not supplied by the target cohort
IGF2AS	1	0	False	absent	not supplied by the target cohort
IGHA1	1	0	False	absent	not supplied by the target cohort
IGL@	1	0	False	absent	not supplied by the target cohort
IGLV8-61	1	0	False	absent	not supplied by the target cohort
IL1F5	1	0	False	absent	not supplied by the target cohort
IL28RA	1	0	False	absent	not supplied by the target cohort
IL8	1	0	False	absent	not supplied by the target cohort
INCA1	1	0	False	absent	not supplied by the target cohort
JHDM1D	1	0	False	absent	not supplied by the target cohort
JPH4	1	0	False	absent	not supplied by the target cohort
KAZ	1	0	False	absent	not supplied by the target cohort
KCNJ12	1	0	False	absent	not supplied by the target cohort
KCTD7	1	0	False	absent	not supplied by the target cohort
KIAA0146	1	0	False	absent	not supplied by the target cohort
KIAA0174	1	0	False	absent	not supplied by the target cohort
KIAA0182	1	0	False	absent	not supplied by the target cohort
KIAA0195	1	0	False	absent	not supplied by the target cohort
KIAA0240	1	0	False	absent	not supplied by the target cohort
KIAA0284	1	0	False	absent	not supplied by the target cohort
KIAA0317	1	0	False	absent	not supplied by the target cohort
KIAA0406	1	0	False	absent	not supplied by the target cohort
KIAA0415	1	0	False	absent	not supplied by the target cohort
KIAA0467	1	0	False	absent	not supplied by the target cohort
KIAA0494	1	0	False	absent	not supplied by the target cohort
KIAA0495	1	0	False	absent	not supplied by the target cohort
KIAA0528	1	0	False	absent	not supplied by the target cohort
KIAA0562	1	0	False	absent	not supplied by the target cohort
KIAA0564	1	0	False	absent	not supplied by the target cohort
KIAA0652	1	0	False	absent	not supplied by the target cohort
KIAA0664	1	0	False	absent	not supplied by the target cohort
KIAA0748	1	0	False	absent	not supplied by the target cohort
KIAA0776	1	0	False	absent	not supplied by the target cohort
KIAA0831	1	0	False	absent	not supplied by the target cohort
KIAA0892	1	0	False	absent	not supplied by the target cohort
KIAA0913	1	0	False	absent	not supplied by the target cohort
KIAA0947	1	0	False	absent	not supplied by the target cohort
KIAA1012	1	0	False	absent	not supplied by the target cohort
KIAA1267	1	0	False	absent	not supplied by the target cohort
KIAA1310	1	0	False	absent	not supplied by the target cohort
KIAA1370	1	0	False	absent	not supplied by the target cohort
KIAA1530	1	0	False	absent	not supplied by the target cohort
KIAA1539	1	0	False	absent	not supplied by the target cohort
KIAA1543	1	0	False	absent	not supplied by the target cohort
KIAA1632	1	0	False	absent	not supplied by the target cohort
KIAA1704	1	0	False	absent	not supplied by the target cohort
KIAA1712	1	0	False	absent	not supplied by the target cohort
KIAA1737	1	0	False	absent	not supplied by the target cohort
KIAA1797	1	0	False	absent	not supplied by the target cohort
KIAA1826	1	0	False	absent	not supplied by the target cohort
KIAA1949	1	0	False	absent	not supplied by the target cohort
KIAA1967	1	0	False	absent	not supplied by the target cohort
KILLIN	1	0	False	absent	not supplied by the target cohort
KLHDC5	1	0	False	absent	not supplied by the target cohort
KLRAQ1	1	0	False	absent	not supplied by the target cohort
KLRC1	1	0	False	absent	not supplied by the target cohort
KLRK1	1	0	False	absent	not supplied by the target cohort
KPNA7	1	0	False	absent	not supplied by the target cohort
KPRP	1	0	False	absent	not supplied by the target cohort

feature	source_coverage	target_coverage	present_in_target	verdict	note
KRT17	1	0	False	absent	not supplied by the target cohort
KRTAP10-1	1	0	False	absent	not supplied by the target cohort
KRTAP10-10	1	0	False	absent	not supplied by the target cohort
KRTAP10-12	1	0	False	absent	not supplied by the target cohort
KRTAP10-3	1	0	False	absent	not supplied by the target cohort
KRTAP10-5	1	0	False	absent	not supplied by the target cohort
KRTAP10-8	1	0	False	absent	not supplied by the target cohort
KRTAP12-1	1	0	False	absent	not supplied by the target cohort
KRTAP12-3	1	0	False	absent	not supplied by the target cohort
KRTAP12-4	1	0	False	absent	not supplied by the target cohort
KRTAP21-2	1	0	False	absent	not supplied by the target cohort
KRTAP23-1	1	0	False	absent	not supplied by the target cohort
KRTAP5-1	1	0	False	absent	not supplied by the target cohort
KRTAP5-11	1	0	False	absent	not supplied by the target cohort
KRTAP6-1	1	0	False	absent	not supplied by the target cohort
KTELC1	1	0	False	absent	not supplied by the target cohort
KTI12	1	0	False	absent	not supplied by the target cohort
LASS2	1	0	False	absent	not supplied by the target cohort
LASS4	1	0	False	absent	not supplied by the target cohort
LASS5	1	0	False	absent	not supplied by the target cohort
LASS6	1	0	False	absent	not supplied by the target cohort
LCE1A	1	0	False	absent	not supplied by the target cohort
LCE1D	1	0	False	absent	not supplied by the target cohort
LCE1F	1	0	False	absent	not supplied by the target cohort
LCE3B	1	0	False	absent	not supplied by the target cohort
LGTN	1	0	False	absent	not supplied by the target cohort
LHB	1	0	False	absent	not supplied by the target cohort
LINS1	1	0	False	absent	not supplied by the target cohort
LIPN	1	0	False	absent	not supplied by the target cohort
LOC100128003	1	0	False	absent	not supplied by the target cohort
LOC100128510	1	0	False	absent	not supplied by the target cohort
LOC100128816	1	0	False	absent	not supplied by the target cohort
LOC100128868	1	0	False	absent	not supplied by the target cohort
LOC100129395	1	0	False	absent	not supplied by the target cohort
LOC100129503	1	0	False	absent	not supplied by the target cohort
LOC100129637	1	0	False	absent	not supplied by the target cohort
LOC100129717	1	0	False	absent	not supplied by the target cohort
LOC100129827	1	0	False	absent	not supplied by the target cohort
LOC100129831	1	0	False	absent	not supplied by the target cohort
LOC100130071	1	0	False	absent	not supplied by the target cohort
LOC100130916	1	0	False	absent	not supplied by the target cohort
LOC100131234	1	0	False	absent	not supplied by the target cohort
LOC100131726	1	0	False	absent	not supplied by the target cohort
LOC100132062	1	0	False	absent	not supplied by the target cohort
LOC100132832	1	0	False	absent	not supplied by the target cohort
LOC100132999	1	0	False	absent	not supplied by the target cohort
LOC100133299	1	0	False	absent	not supplied by the target cohort
LOC100287036	1	0	False	absent	not supplied by the target cohort
LOC100287042	1	0	False	absent	not supplied by the target cohort
LOC100287685	1	0	False	absent	not supplied by the target cohort
LOC100288114	1	0	False	absent	not supplied by the target cohort
LOC100288346	1	0	False	absent	not supplied by the target cohort
LOC100288418	1	0	False	absent	not supplied by the target cohort
LOC100289135	1	0	False	absent	not supplied by the target cohort
LOC100289314	1	0	False	absent	not supplied by the target cohort
LOC100289409	1	0	False	absent	not supplied by the target cohort
LOC100289574	1	0	False	absent	not supplied by the target cohort
LOC100290676	1	0	False	absent	not supplied by the target cohort
LOC100291428	1	0	False	absent	not supplied by the target cohort
LOC100291682	1	0	False	absent	not supplied by the target cohort
LOC100293342	1	0	False	absent	not supplied by the target cohort
LOC145757	1	0	False	absent	not supplied by the target cohort
LOC162632	1	0	False	absent	not supplied by the target cohort
LOC171220	1	0	False	absent	not supplied by the target cohort
LOC219347	1	0	False	absent	not supplied by the target cohort
LOC220930	1	0	False	absent	not supplied by the target cohort
LOC221710	1	0	False	absent	not supplied by the target cohort
LOC284749	1	0	False	absent	not supplied by the target cohort
LOC338797	1	0	False	absent	not supplied by the target cohort
LOC339047	1	0	False	absent	not supplied by the target cohort
LOC339240	1	0	False	absent	not supplied by the target cohort
LOC375190	1	0	False	absent	not supplied by the target cohort
LOC388387	1	0	False	absent	not supplied by the target cohort
LOC389333	1	0	False	absent	not supplied by the target cohort
LOC391322	1	0	False	absent	not supplied by the target cohort
LOC401934	1	0	False	absent	not supplied by the target cohort
LOC402644	1	0	False	absent	not supplied by the target cohort
LOC440104	1	0	False	absent	not supplied by the target cohort
LOC440248	1	0	False	absent	not supplied by the target cohort
LOC440313	1	0	False	absent	not supplied by the target cohort
LOC440337	1	0	False	absent	not supplied by the target cohort
LOC440957	1	0	False	absent	not supplied by the target cohort
LOC441493	1	0	False	absent	not supplied by the target cohort
LOC613206	1	0	False	absent	not supplied by the target cohort
LOC642846	1	0	False	absent	not supplied by the target cohort
LOC643008	1	0	False	absent	not supplied by the target cohort

feature	source_coverage	target_coverage	present_in_target	verdict	note
LOC646938	1	0	False	absent	not supplied by the target cohort
LOC646982	1	0	False	absent	not supplied by the target cohort
LOC647979	1	0	False	absent	not supplied by the target cohort
LOC653712	1	0	False	absent	not supplied by the target cohort
LOC728417	1	0	False	absent	not supplied by the target cohort
LOC728752	1	0	False	absent	not supplied by the target cohort
LOC729678	1	0	False	absent	not supplied by the target cohort
LOC729852	1	0	False	absent	not supplied by the target cohort
LOC729991	1	0	False	absent	not supplied by the target cohort
LOC90110	1	0	False	absent	not supplied by the target cohort
LOC90925	1	0	False	absent	not supplied by the target cohort
LONP2	1	0	False	absent	not supplied by the target cohort
LQK1	1	0	False	absent	not supplied by the target cohort
LRAT	1	0	False	absent	not supplied by the target cohort
LRDD	1	0	False	absent	not supplied by the target cohort
LRRC26	1	0	False	absent	not supplied by the target cohort
LRRC33	1	0	False	absent	not supplied by the target cohort
LRRC37A4	1	0	False	absent	not supplied by the target cohort
LRRC50	1	0	False	absent	not supplied by the target cohort
LRRC8C	1	0	False	absent	not supplied by the target cohort
LRWD1	1	0	False	absent	not supplied by the target cohort
LSMD1	1	0	False	absent	not supplied by the target cohort
LTA4H	1	0	False	absent	not supplied by the target cohort
LY6G6F	1	0	False	absent	not supplied by the target cohort
MAEA	1	0	False	absent	not supplied by the target cohort
MAGOH	1	0	False	absent	not supplied by the target cohort
MAP2K3	1	0	False	absent	not supplied by the target cohort
MAPKSP1	1	0	False	absent	not supplied by the target cohort
MARS	1	0	False	absent	not supplied by the target cohort
MBD3L5	1	0	False	absent	not supplied by the target cohort
MBL1P	1	0	False	absent	not supplied by the target cohort
MCCD1	1	0	False	absent	not supplied by the target cohort
MCM3APAS	1	0	False	absent	not supplied by the target cohort
MDP1	1	0	False	absent	not supplied by the target cohort
MED18	1	0	False	absent	not supplied by the target cohort
MED21	1	0	False	absent	not supplied by the target cohort
METT10D	1	0	False	absent	not supplied by the target cohort
METT5D1	1	0	False	absent	not supplied by the target cohort
METTL11A	1	0	False	absent	not supplied by the target cohort
METTL2A	1	0	False	absent	not supplied by the target cohort
MFAP3L	1	0	False	absent	not supplied by the target cohort
MFSD2B	1	0	False	absent	not supplied by the target cohort
MGC27345	1	0	False	absent	not supplied by the target cohort
MGC29506	1	0	False	absent	not supplied by the target cohort
MGC31957	1	0	False	absent	not supplied by the target cohort
MGC39372	1	0	False	absent	not supplied by the target cohort
MIR21	1	0	False	absent	not supplied by the target cohort
MKI67IP	1	0	False	absent	not supplied by the target cohort
MLF1IP	1	0	False	absent	not supplied by the target cohort
MLL	1	0	False	absent	not supplied by the target cohort
MLL3	1	0	False	absent	not supplied by the target cohort
MLL4	1	0	False	absent	not supplied by the target cohort
MLL5	1	0	False	absent	not supplied by the target cohort
MOBKL1A	1	0	False	absent	not supplied by the target cohort
MOBKL1B	1	0	False	absent	not supplied by the target cohort
MOBKL2A	1	0	False	absent	not supplied by the target cohort
MOBKL2B	1	0	False	absent	not supplied by the target cohort
MOBKL2C	1	0	False	absent	not supplied by the target cohort
MOBKL3	1	0	False	absent	not supplied by the target cohort
MORF4	1	0	False	absent	not supplied by the target cohort
MOSC1	1	0	False	absent	not supplied by the target cohort
MRGPRD	1	0	False	absent	not supplied by the target cohort
MRGPRE	1	0	False	absent	not supplied by the target cohort
MRP63	1	0	False	absent	not supplied by the target cohort
MRPS24	1	0	False	absent	not supplied by the target cohort
MSH5	1	0	False	absent	not supplied by the target cohort
MTERF	1	0	False	absent	not supplied by the target cohort
MTERFD1	1	0	False	absent	not supplied by the target cohort
MTERFD2	1	0	False	absent	not supplied by the target cohort
MTHFS	1	0	False	absent	not supplied by the target cohort
MTMR15	1	0	False	absent	not supplied by the target cohort
MTMR9L	1	0	False	absent	not supplied by the target cohort
MTP18	1	0	False	absent	not supplied by the target cohort
MUC21	1	0	False	absent	not supplied by the target cohort
MUDENG	1	0	False	absent	not supplied by the target cohort
MUSTN1	1	0	False	absent	not supplied by the target cohort
MUTED	1	0	False	absent	not supplied by the target cohort
MXD4	1	0	False	absent	not supplied by the target cohort
MYCBP	1	0	False	absent	not supplied by the target cohort
MYCL1	1	0	False	absent	not supplied by the target cohort
MYST1	1	0	False	absent	not supplied by the target cohort
MYST2	1	0	False	absent	not supplied by the target cohort
MYST3	1	0	False	absent	not supplied by the target cohort
MYST4	1	0	False	absent	not supplied by the target cohort
March 1	1	0	False	absent	not supplied by the target cohort
March 2	1	0	False	absent	not supplied by the target cohort

feature	source_coverage	target_coverage	present_in_target	verdict	note
March 3	1	0	False	absent	not supplied by the target cohort
March 5	1	0	False	absent	not supplied by the target cohort
March 6	1	0	False	absent	not supplied by the target cohort
March 7	1	0	False	absent	not supplied by the target cohort
March 8	1	0	False	absent	not supplied by the target cohort
March 9	1	0	False	absent	not supplied by the target cohort
NAPRT1	1	0	False	absent	not supplied by the target cohort
NARG2	1	0	False	absent	not supplied by the target cohort
NAT15	1	0	False	absent	not supplied by the target cohort
NCRNA00085	1	0	False	absent	not supplied by the target cohort
NCRNA00105	1	0	False	absent	not supplied by the target cohort
NCRNA00110	1	0	False	absent	not supplied by the target cohort
NCRNA00114	1	0	False	absent	not supplied by the target cohort
NCRNA00188	1	0	False	absent	not supplied by the target cohort
NCRNA00201	1	0	False	absent	not supplied by the target cohort
NCRNA00204	1	0	False	absent	not supplied by the target cohort
NCRNA00219	1	0	False	absent	not supplied by the target cohort
NCRNA00235	1	0	False	absent	not supplied by the target cohort
NDUFC2	1	0	False	absent	not supplied by the target cohort
NDUFS5	1	0	False	absent	not supplied by the target cohort
NDUFS8	1	0	False	absent	not supplied by the target cohort
NEIL1	1	0	False	absent	not supplied by the target cohort
NELF	1	0	False	absent	not supplied by the target cohort
NHEDC2	1	0	False	absent	not supplied by the target cohort
NKX1-2	1	0	False	absent	not supplied by the target cohort
NOMO2	1	0	False	absent	not supplied by the target cohort
NOP56	1	0	False	absent	not supplied by the target cohort
NRBP2	1	0	False	absent	not supplied by the target cohort
NSUN5P2	1	0	False	absent	not supplied by the target cohort
NT5C3	1	0	False	absent	not supplied by the target cohort
NT5C3L	1	0	False	absent	not supplied by the target cohort
NUDT8	1	0	False	absent	not supplied by the target cohort
NUTF2	1	0	False	absent	not supplied by the target cohort
OBFC2A	1	0	False	absent	not supplied by the target cohort
OBFC2B	1	0	False	absent	not supplied by the target cohort
ODZ1	1	0	False	absent	not supplied by the target cohort
OMG	1	0	False	absent	not supplied by the target cohort
OR10G7	1	0	False	absent	not supplied by the target cohort
OR13H1	1	0	False	absent	not supplied by the target cohort
OR14I1	1	0	False	absent	not supplied by the target cohort
OR1L1	1	0	False	absent	not supplied by the target cohort
OR1N1	1	0	False	absent	not supplied by the target cohort
OR1N2	1	0	False	absent	not supplied by the target cohort
OR2A14	1	0	False	absent	not supplied by the target cohort
OR2A2	1	0	False	absent	not supplied by the target cohort
OR2T2	1	0	False	absent	not supplied by the target cohort
OR2T34	1	0	False	absent	not supplied by the target cohort
OR2T6	1	0	False	absent	not supplied by the target cohort
OR2W3	1	0	False	absent	not supplied by the target cohort
OR4N2	1	0	False	absent	not supplied by the target cohort
OR51D1	1	0	False	absent	not supplied by the target cohort
OR52R1	1	0	False	absent	not supplied by the target cohort
OR56A4	1	0	False	absent	not supplied by the target cohort
OR5A2	1	0	False	absent	not supplied by the target cohort
OR7D4	1	0	False	absent	not supplied by the target cohort
OR9K2	1	0	False	absent	not supplied by the target cohort
ORC2L	1	0	False	absent	not supplied by the target cohort
ORC3L	1	0	False	absent	not supplied by the target cohort
ORC4L	1	0	False	absent	not supplied by the target cohort
ORC5L	1	0	False	absent	not supplied by the target cohort
ORM2	1	0	False	absent	not supplied by the target cohort
OSTalpha	1	0	False	absent	not supplied by the target cohort
OTUB1	1	0	False	absent	not supplied by the target cohort
P2RY11	1	0	False	absent	not supplied by the target cohort
PABPC4	1	0	False	absent	not supplied by the target cohort
PABPN1L	1	0	False	absent	not supplied by the target cohort
PAH	1	0	False	absent	not supplied by the target cohort
PCDHA11	1	0	False	absent	not supplied by the target cohort
PCDHA13	1	0	False	absent	not supplied by the target cohort
PCDHA4	1	0	False	absent	not supplied by the target cohort
PCDHGB4	1	0	False	absent	not supplied by the target cohort
PDCL3	1	0	False	absent	not supplied by the target cohort
PDIA5	1	0	False	absent	not supplied by the target cohort
PECI	1	0	False	absent	not supplied by the target cohort
PER1	1	0	False	absent	not supplied by the target cohort
PET112L	1	0	False	absent	not supplied by the target cohort
PFDN6	1	0	False	absent	not supplied by the target cohort
PFN3	1	0	False	absent	not supplied by the target cohort
PGCP	1	0	False	absent	not supplied by the target cohort
PHF15	1	0	False	absent	not supplied by the target cohort
PHF17	1	0	False	absent	not supplied by the target cohort
PI4K2B	1	0	False	absent	not supplied by the target cohort
PI4KAP2	1	0	False	absent	not supplied by the target cohort
PIGY	1	0	False	absent	not supplied by the target cohort
PILRB	1	0	False	absent	not supplied by the target cohort
PION	1	0	False	absent	not supplied by the target cohort

feature	source_coverage	target_coverage	present_in_target	verdict	note
PISD	1	0	False	absent	not supplied by the target cohort
PKM2	1	0	False	absent	not supplied by the target cohort
PL-5283	1	0	False	absent	not supplied by the target cohort
PLDN	1	0	False	absent	not supplied by the target cohort
PLEKHJ1	1	0	False	absent	not supplied by the target cohort
PLEKHM1P	1	0	False	absent	not supplied by the target cohort
PLK1S1	1	0	False	absent	not supplied by the target cohort
PLSCR3	1	0	False	absent	not supplied by the target cohort
PMS2CL	1	0	False	absent	not supplied by the target cohort
PMS2L11	1	0	False	absent	not supplied by the target cohort
PMS2L5	1	0	False	absent	not supplied by the target cohort
PNMA5	1	0	False	absent	not supplied by the target cohort
POU2AF1	1	0	False	absent	not supplied by the target cohort
PPAN	1	0	False	absent	not supplied by the target cohort
PPIAL4G	1	0	False	absent	not supplied by the target cohort
PPIL5	1	0	False	absent	not supplied by the target cohort
PPP2R3B	1	0	False	absent	not supplied by the target cohort
PPPDE1	1	0	False	absent	not supplied by the target cohort
PPPDE2	1	0	False	absent	not supplied by the target cohort
PPT2	1	0	False	absent	not supplied by the target cohort
PRH1	1	0	False	absent	not supplied by the target cohort
PRIC285	1	0	False	absent	not supplied by the target cohort
PRKRIP1	1	0	False	absent	not supplied by the target cohort
PRMT10	1	0	False	absent	not supplied by the target cohort
PRODH	1	0	False	absent	not supplied by the target cohort
PRR25	1	0	False	absent	not supplied by the target cohort
PRSS1	1	0	False	absent	not supplied by the target cohort
PRSS41	1	0	False	absent	not supplied by the target cohort
PRSSL1	1	0	False	absent	not supplied by the target cohort
PS1TP4	1	0	False	absent	not supplied by the target cohort
PSMA6	1	0	False	absent	not supplied by the target cohort
PSME2	1	0	False	absent	not supplied by the target cohort
PTCD1	1	0	False	absent	not supplied by the target cohort
PTX4	1	0	False	absent	not supplied by the target cohort
PYCR2	1	0	False	absent	not supplied by the target cohort
ProSAPiP1	1	0	False	absent	not supplied by the target cohort
QARS	1	0	False	absent	not supplied by the target cohort
RAB34	1	0	False	absent	not supplied by the target cohort
RAB4A	1	0	False	absent	not supplied by the target cohort
RAB7L1	1	0	False	absent	not supplied by the target cohort
RABL2A	1	0	False	absent	not supplied by the target cohort
RABL2B	1	0	False	absent	not supplied by the target cohort
RABL5	1	0	False	absent	not supplied by the target cohort
RAG1AP1	1	0	False	absent	not supplied by the target cohort
RAGE	1	0	False	absent	not supplied by the target cohort
RBM16	1	0	False	absent	not supplied by the target cohort
RBM9	1	0	False	absent	not supplied by the target cohort
RBMX	1	0	False	absent	not supplied by the target cohort
RDBP	1	0	False	absent	not supplied by the target cohort
RDH14	1	0	False	absent	not supplied by the target cohort
RG9MTD1	1	0	False	absent	not supplied by the target cohort
RG9MTD3	1	0	False	absent	not supplied by the target cohort
RGNEF	1	0	False	absent	not supplied by the target cohort
RGPD4	1	0	False	absent	not supplied by the target cohort
RHCE	1	0	False	absent	not supplied by the target cohort
RIC8A	1	0	False	absent	not supplied by the target cohort
RIF1	1	0	False	absent	not supplied by the target cohort
RINL	1	0	False	absent	not supplied by the target cohort
RNASEK	1	0	False	absent	not supplied by the target cohort
RNASEN	1	0	False	absent	not supplied by the target cohort
RNF160	1	0	False	absent	not supplied by the target cohort
RNF216L	1	0	False	absent	not supplied by the target cohort
ROBLD3	1	0	False	absent	not supplied by the target cohort
ROCK1P1	1	0	False	absent	not supplied by the target cohort
ROD1	1	0	False	absent	not supplied by the target cohort
RPL10	1	0	False	absent	not supplied by the target cohort
RPL13	1	0	False	absent	not supplied by the target cohort
RPL13A	1	0	False	absent	not supplied by the target cohort
RPL13P5	1	0	False	absent	not supplied by the target cohort
RPL17	1	0	False	absent	not supplied by the target cohort
RPL18A	1	0	False	absent	not supplied by the target cohort
RPL21	1	0	False	absent	not supplied by the target cohort
RPL23	1	0	False	absent	not supplied by the target cohort
RPL23AP82	1	0	False	absent	not supplied by the target cohort
RPL26	1	0	False	absent	not supplied by the target cohort
RPL28	1	0	False	absent	not supplied by the target cohort
RPL4	1	0	False	absent	not supplied by the target cohort
RPL5	1	0	False	absent	not supplied by the target cohort
RPL7A	1	0	False	absent	not supplied by the target cohort
RPS13	1	0	False	absent	not supplied by the target cohort
RPS2	1	0	False	absent	not supplied by the target cohort
RPS20	1	0	False	absent	not supplied by the target cohort
RPS26	1	0	False	absent	not supplied by the target cohort
RPS2P32	1	0	False	absent	not supplied by the target cohort
RPS3A	1	0	False	absent	not supplied by the target cohort
RSC1A1	1	0	False	absent	not supplied by the target cohort

feature	source_coverage	target_coverage	present_in_target	verdict	note
RSPH10B2	1	0	False	absent	not supplied by the target cohort
RTCD1	1	0	False	absent	not supplied by the target cohort
RTL1	1	0	False	absent	not supplied by the target cohort
RUNDC2A	1	0	False	absent	not supplied by the target cohort
RUNX1	1	0	False	absent	not supplied by the target cohort
RWDD4A	1	0	False	absent	not supplied by the target cohort
SAPS1	1	0	False	absent	not supplied by the target cohort
SAPS2	1	0	False	absent	not supplied by the target cohort
SAPS3	1	0	False	absent	not supplied by the target cohort
SBDS	1	0	False	absent	not supplied by the target cohort
SC4MOL	1	0	False	absent	not supplied by the target cohort
SC5DL	1	0	False	absent	not supplied by the target cohort
SCARNA20	1	0	False	absent	not supplied by the target cohort
SCLT1	1	0	False	absent	not supplied by the target cohort
SCNM1	1	0	False	absent	not supplied by the target cohort
SDCCAG1	1	0	False	absent	not supplied by the target cohort
SDHAP2	1	0	False	absent	not supplied by the target cohort
SEC1	1	0	False	absent	not supplied by the target cohort
SELS	1	0	False	absent	not supplied by the target cohort
SEPX1	1	0	False	absent	not supplied by the target cohort
SERF1A	1	0	False	absent	not supplied by the target cohort
SERF2	1	0	False	absent	not supplied by the target cohort
SERINC4	1	0	False	absent	not supplied by the target cohort
SF3B14	1	0	False	absent	not supplied by the target cohort
SF4	1	0	False	absent	not supplied by the target cohort
SFRS1	1	0	False	absent	not supplied by the target cohort
SFRS11	1	0	False	absent	not supplied by the target cohort
SFRS12	1	0	False	absent	not supplied by the target cohort
SFRS12IP1	1	0	False	absent	not supplied by the target cohort
SFRS13A	1	0	False	absent	not supplied by the target cohort
SFRS14	1	0	False	absent	not supplied by the target cohort
SFRS15	1	0	False	absent	not supplied by the target cohort
SFRS16	1	0	False	absent	not supplied by the target cohort
SFRS17A	1	0	False	absent	not supplied by the target cohort
SFRS18	1	0	False	absent	not supplied by the target cohort
SFRS2	1	0	False	absent	not supplied by the target cohort
SFRS2B	1	0	False	absent	not supplied by the target cohort
SFRS2IP	1	0	False	absent	not supplied by the target cohort
SFRS3	1	0	False	absent	not supplied by the target cohort
SFRS4	1	0	False	absent	not supplied by the target cohort
SFRS5	1	0	False	absent	not supplied by the target cohort
SFRS6	1	0	False	absent	not supplied by the target cohort
SFRS7	1	0	False	absent	not supplied by the target cohort
SFRS8	1	0	False	absent	not supplied by the target cohort
SFRS9	1	0	False	absent	not supplied by the target cohort
SGK269	1	0	False	absent	not supplied by the target cohort
SGK3	1	0	False	absent	not supplied by the target cohort
SH2D6	1	0	False	absent	not supplied by the target cohort
SH3D20	1	0	False	absent	not supplied by the target cohort
SIGLEC14	1	0	False	absent	not supplied by the target cohort
SIGLECP3	1	0	False	absent	not supplied by the target cohort
SLC16A3	1	0	False	absent	not supplied by the target cohort
SLC22A10	1	0	False	absent	not supplied by the target cohort
SLC24A6	1	0	False	absent	not supplied by the target cohort
SLC26A5	1	0	False	absent	not supplied by the target cohort
SLC35E2	1	0	False	absent	not supplied by the target cohort
SLC35E4	1	0	False	absent	not supplied by the target cohort
SLC36A2	1	0	False	absent	not supplied by the target cohort
SMCR7L	1	0	False	absent	not supplied by the target cohort
SMG1	1	0	False	absent	not supplied by the target cohort
SMN2	1	0	False	absent	not supplied by the target cohort
SNAP47	1	0	False	absent	not supplied by the target cohort
SNHG1	1	0	False	absent	not supplied by the target cohort
SNHG11	1	0	False	absent	not supplied by the target cohort
SNHG12	1	0	False	absent	not supplied by the target cohort
SNHG7	1	0	False	absent	not supplied by the target cohort
SNHG8	1	0	False	absent	not supplied by the target cohort
SNHG9	1	0	False	absent	not supplied by the target cohort
SNORA10	1	0	False	absent	not supplied by the target cohort
SNORA28	1	0	False	absent	not supplied by the target cohort
SNORA30	1	0	False	absent	not supplied by the target cohort
SNORA47	1	0	False	absent	not supplied by the target cohort
SNORA49	1	0	False	absent	not supplied by the target cohort
SNORA71C	1	0	False	absent	not supplied by the target cohort
SNORA76	1	0	False	absent	not supplied by the target cohort
SNORA77	1	0	False	absent	not supplied by the target cohort
SNORD15A	1	0	False	absent	not supplied by the target cohort
SNORD72	1	0	False	absent	not supplied by the target cohort
SOLH	1	0	False	absent	not supplied by the target cohort
SP140	1	0	False	absent	not supplied by the target cohort
SP9	1	0	False	absent	not supplied by the target cohort
SPEG	1	0	False	absent	not supplied by the target cohort
SPHAR	1	0	False	absent	not supplied by the target cohort
SPINK8	1	0	False	absent	not supplied by the target cohort
SPON2	1	0	False	absent	not supplied by the target cohort
SR140	1	0	False	absent	not supplied by the target cohort

feature	source_coverage	target_coverage	present_in_target	verdict	note
SSX6	1	0	False	absent	not supplied by the target cohort
SSX7	1	0	False	absent	not supplied by the target cohort
ST7	1	0	False	absent	not supplied by the target cohort
STAG3L2	1	0	False	absent	not supplied by the target cohort
SUMO2	1	0	False	absent	not supplied by the target cohort
SYNJ2BP	1	0	False	absent	not supplied by the target cohort
SYVN1	1	0	False	absent	not supplied by the target cohort
Septin 1	1	0	False	absent	not supplied by the target cohort
Septin 11	1	0	False	absent	not supplied by the target cohort
Septin 14	1	0	False	absent	not supplied by the target cohort
Septin 2	1	0	False	absent	not supplied by the target cohort
Septin 5	1	0	False	absent	not supplied by the target cohort
Septin 6	1	0	False	absent	not supplied by the target cohort
Septin 7	1	0	False	absent	not supplied by the target cohort
Septin 8	1	0	False	absent	not supplied by the target cohort
Septin 9	1	0	False	absent	not supplied by the target cohort
TAF9	1	0	False	absent	not supplied by the target cohort
TARM1	1	0	False	absent	not supplied by the target cohort
TAS1R3	1	0	False	absent	not supplied by the target cohort
TAX1BP3	1	0	False	absent	not supplied by the target cohort
TCF23	1	0	False	absent	not supplied by the target cohort
TCP1	1	0	False	absent	not supplied by the target cohort
TDGF3	1	0	False	absent	not supplied by the target cohort
TEKT4	1	0	False	absent	not supplied by the target cohort
TH1L	1	0	False	absent	not supplied by the target cohort
THOC4	1	0	False	absent	not supplied by the target cohort
TIMM16	1	0	False	absent	not supplied by the target cohort
TMBIM1	1	0	False	absent	not supplied by the target cohort
TMED7	1	0	False	absent	not supplied by the target cohort
TMEM111	1	0	False	absent	not supplied by the target cohort
TMEM149	1	0	False	absent	not supplied by the target cohort
TMEM164	1	0	False	absent	not supplied by the target cohort
TMEM179B	1	0	False	absent	not supplied by the target cohort
TMEM183A	1	0	False	absent	not supplied by the target cohort
TMEM188	1	0	False	absent	not supplied by the target cohort
TMEM189	1	0	False	absent	not supplied by the target cohort
TMEM199	1	0	False	absent	not supplied by the target cohort
TMEM211	1	0	False	absent	not supplied by the target cohort
TMEM48	1	0	False	absent	not supplied by the target cohort
TMEM49	1	0	False	absent	not supplied by the target cohort
TMEM66	1	0	False	absent	not supplied by the target cohort
TMEM81	1	0	False	absent	not supplied by the target cohort
TMEM82	1	0	False	absent	not supplied by the target cohort
TMEM85	1	0	False	absent	not supplied by the target cohort
TMEM88B	1	0	False	absent	not supplied by the target cohort
TMEM93	1	0	False	absent	not supplied by the target cohort
TMPRSS9	1	0	False	absent	not supplied by the target cohort
TNFSF12	1	0	False	absent	not supplied by the target cohort
TOMM6	1	0	False	absent	not supplied by the target cohort
TPT1	1	0	False	absent	not supplied by the target cohort
TRA@	1	0	False	absent	not supplied by the target cohort
TRAJ17	1	0	False	absent	not supplied by the target cohort
TRAPPC4	1	0	False	absent	not supplied by the target cohort
TRBV9	1	0	False	absent	not supplied by the target cohort
TRD@	1	0	False	absent	not supplied by the target cohort
TREX1	1	0	False	absent	not supplied by the target cohort
TRIM39	1	0	False	absent	not supplied by the target cohort
TSGA14	1	0	False	absent	not supplied by the target cohort
TTC15	1	0	False	absent	not supplied by the target cohort
TTC21B	1	0	False	absent	not supplied by the target cohort
TTC3	1	0	False	absent	not supplied by the target cohort
TTC35	1	0	False	absent	not supplied by the target cohort
TTTY14	1	0	False	absent	not supplied by the target cohort
TTTY6B	1	0	False	absent	not supplied by the target cohort
TUBB2C	1	0	False	absent	not supplied by the target cohort
TXNDC3	1	0	False	absent	not supplied by the target cohort
TYW1B	1	0	False	absent	not supplied by the target cohort
UBA7	1	0	False	absent	not supplied by the target cohort
UBE2V1	1	0	False	absent	not supplied by the target cohort
UBXN6	1	0	False	absent	not supplied by the target cohort
UCK2	1	0	False	absent	not supplied by the target cohort
UGT2B7	1	0	False	absent	not supplied by the target cohort
UNCX	1	0	False	absent	not supplied by the target cohort
UQCRH	1	0	False	absent	not supplied by the target cohort
UQCRHL	1	0	False	absent	not supplied by the target cohort
USP15	1	0	False	absent	not supplied by the target cohort
UTP14C	1	0	False	absent	not supplied by the target cohort
VIPAR	1	0	False	absent	not supplied by the target cohort
VPS24	1	0	False	absent	not supplied by the target cohort
WASH2P	1	0	False	absent	not supplied by the target cohort
WASH3P	1	0	False	absent	not supplied by the target cohort
WASH7P	1	0	False	absent	not supplied by the target cohort
WBSCR26	1	0	False	absent	not supplied by the target cohort
WDR33	1	0	False	absent	not supplied by the target cohort
WDR45L	1	0	False	absent	not supplied by the target cohort
WDR8	1	0	False	absent	not supplied by the target cohort

feature	source_coverage	target_coverage	present_in_target	verdict	note
WDR85	1	0	False	absent	not supplied by the target cohort
WHAMML1	1	0	False	absent	not supplied by the target cohort
WHSC2	1	0	False	absent	not supplied by the target cohort
WIT1	1	0	False	absent	not supplied by the target cohort
WNT3	1	0	False	absent	not supplied by the target cohort
WSCD1	1	0	False	absent	not supplied by the target cohort
XKR3	1	0	False	absent	not supplied by the target cohort
YIPF7	1	0	False	absent	not supplied by the target cohort
ZC3H11A	1	0	False	absent	not supplied by the target cohort
ZC3H12A	1	0	False	absent	not supplied by the target cohort
ZEB1	1	0	False	absent	not supplied by the target cohort
ZFP106	1	0	False	absent	not supplied by the target cohort
ZFP161	1	0	False	absent	not supplied by the target cohort
ZMYND17	1	0	False	absent	not supplied by the target cohort
ZNF131	1	0	False	absent	not supplied by the target cohort
ZNF137	1	0	False	absent	not supplied by the target cohort
ZNF167	1	0	False	absent	not supplied by the target cohort
ZNF187	1	0	False	absent	not supplied by the target cohort
ZNF193	1	0	False	absent	not supplied by the target cohort
ZNF238	1	0	False	absent	not supplied by the target cohort
ZNF252	1	0	False	absent	not supplied by the target cohort
ZNF259	1	0	False	absent	not supplied by the target cohort
ZNF263	1	0	False	absent	not supplied by the target cohort
ZNF280D	1	0	False	absent	not supplied by the target cohort
ZNF295	1	0	False	absent	not supplied by the target cohort
ZNF321	1	0	False	absent	not supplied by the target cohort
ZNF322A	1	0	False	absent	not supplied by the target cohort
ZNF37A	1	0	False	absent	not supplied by the target cohort
ZNF397OS	1	0	False	absent	not supplied by the target cohort
ZNF410	1	0	False	absent	not supplied by the target cohort
ZNF434	1	0	False	absent	not supplied by the target cohort
ZNF561	1	0	False	absent	not supplied by the target cohort
ZNF643	1	0	False	absent	not supplied by the target cohort
ZNF673	1	0	False	absent	not supplied by the target cohort
ZNF707	1	0	False	absent	not supplied by the target cohort
ZNF720	1	0	False	absent	not supplied by the target cohort
ZNF767	1	0	False	absent	not supplied by the target cohort
ZNF772	1	0	False	absent	not supplied by the target cohort
ZNF773	1	0	False	absent	not supplied by the target cohort
ZNF828	1	0	False	absent	not supplied by the target cohort
ZNF841	1	0	False	absent	not supplied by the target cohort
ZP3	1	0	False	absent	not supplied by the target cohort
ZRSR1	1	0	False	absent	not supplied by the target cohort
ZSCAN5B	1	0	False	absent	not supplied by the target cohort
selenoprotein 15	1	0	False	absent	not supplied by the target cohort

Table S7. Full random-gene-panel benchmark grid (Methods 4.8, Section 2.1, Figure 3). Every cell draws 2000 panels. `pool` is the candidate universe the panels are drawn from; `recipe` is the modeling specification, applied identically to the selected and the random panels; `real_panel_selection_mode` records how the selected panel was resized at that k. `prop_ci_lo` and `prop_ci_hi` are exact Clopper-Pearson bounds on the proportion.

pool	panel_size	recipe	task	real_auroc_refit	real_panel_is_locked_signature	real_panel_selection_C	real_panel_selection_mode	real_panel_genes	locked_model_auroc	null_mean	null_sd	null_p05
transportable	5	l1_modalC	validation	1	False	0.02617	l1_only	FBXL16;NELL2;NMT2;P2RY10;S100A8	nan	0.9033	0.1206	0.6627
transportable	5	l1_modalC	specificity	0.7212	False	0.02617	l1_only	FBXL16;NELL2;NMT2;P2RY10;S100A8	nan	0.6261	0.0662	0.5146
transportable	10	l1_modalC	validation	1	True	0.1	locked_signature	FAM43A;FBXL16;FCER1A;KLRB1;NELL2;NMT2;P2RY10;S100A8;SBK1;SLC7A6	1	0.9598	0.0633	0.8471
transportable	10	l1_modalC	specificity	0.7339	True	0.1	locked_signature	FAM43A;FBXL16;FCER1A;KLRB1;NELL2;NMT2;P2RY10;S100A8;SBK1;SLC7A6	0.7329	0.6593	0.0547	0.5632
transportable	20	l1_modalC	validation	1	False	5.266	l1_only	ANXA3;AQP3;BCL2;CCR3;CD177;CD247;FAM43A;FBXL16;FCER1A;IL10RA;KLRB1;NELL2;P2RY10;S100A12;S100A8;SBK1;SPN;VNN1;WDR82;ZNF395	nan	0.9841	0.0332	0.9399
transportable	20	l1_modalC	specificity	0.7336	False	5.266	l1_only	ANXA3;AQP3;BCL2;CCR3;CD177;CD247;FAM43A;FBXL16;FCER1A;IL10RA;KLRB1;NELL2;P2RY10;S100A12;S100A8;SBK1;SPN;VNN1;WDR82;ZNF395	nan	0.6826	0.0453	0.6051

pool	panel_size	recipe	task	real_auroc_refit	real_panel_is_locked_signature	real_panel_selection_C	real_panel_selection_mode	real_panel_genes	locked_model_auroc	null_mean	null_sd	null_p05
transportable	50	l1_modalC	validation	1	False	40.51	l1_only	ANXA3;APOL3;AQP3;ARG1;ASPH;ASTE1;BCL2;BTN3A3;CCR3;CD177;CD247;CDKN2D;CECR1;FAM162A;FAM43A;FBXL16;FCER1A;GPR18;HMOX2;IKBKB;IL10RA;IL18R1;IRAK3;KLRB1;LPXN;MS4A4A;NCBP2;NELL2;OLAH;P2RY10;PPP1R16B;RCC2;S100A12;S100A8;SAMSN1;SBK1;SET;SIRPG;SLC26A8;SLC38A1;SLC7A6;SPN;TMEM109;TP53I3;VNN1;WDR82;ZDHHC19;ZNF275;ZNF395;ZNF438	nan	0.995	0.0115	0.9786

pool	panel_size	recipe	task	real_auroc_refit	real_panel_is_locked_signature	real_panel_selection_C	real_panel_selection_mode	real_panel_genes	locked_model_auroc	null_mean	null_sd	null_p05
transportable	50	l1_modalC	specificity	0.7328	False	40.51	l1_only	ANXA3;APOL3;AQP3;ARG1;ASPH;ASTE1;BCL2;BTN3A3;CCR3;CD177;CD247;CDKN2D;CECR1;FAM162A;FAM43A;FBXL16;FCER1A;GPR18;HMOX2;IKBKB;IL10RA;IL18R1;IRAK3;KLRB1;LPXN;MS4A4A;NCBP2;NELL2;OLAH;P2RY10;PPP1R16B;RCC2;S100A12;S100A8;SAMSN1;SBK1;SET;SIRPG;SLC26A8;SLC38A1;SLC7A6;SPN;TMEM109;TP53I3;VNN1;WDR82;ZDHHC19;ZNF275;ZNF395;ZNF438	nan	0.7015	0.0365	0.6404
transportable	10	unpenalized	validation	1	True	0.1	locked_signature	FAM43A;FBXL16;FCER1A;KLRB1;NELL2;NMT2;P2RY10;S100A8;SBK1;SLC7A6	1	0.9124	0.1365	0.5
transportable	10	unpenalized	specificity	0.734	True	0.1	locked_signature	FAM43A;FBXL16;FCER1A;KLRB1;NELL2;NMT2;P2RY10;S100A8;SBK1;SLC7A6	0.7329	0.6508	0.0652	0.5397
top300_candidate	10	l1_modalC	validation	1	True	0.1	locked_signature	FAM43A;FBXL16;FCER1A;KLRB1;NELL2;NMT2;P2RY10;S100A8;SBK1;SLC7A6	1	0.9987	0.0029	0.9929
top300_candidate	10	l1_modalC	specificity	0.7339	True	0.1	locked_signature	FAM43A;FBXL16;FCER1A;KLRB1;NELL2;NMT2;P2RY10;S100A8;SBK1;SLC7A6	0.7329	0.705	0.0295	0.656

pool	panel_size	recipe	task	real_auroc_refit	real_panel_is_locked_signature	real_panel_selection_C	real_panel_selection_mode	real_panel_genes	locked_model_auroc	null_mean	null_sd	null_p05
top300_candidate	10	unpenalized	validation	1	True	0.1	locked_signature	FAM43A; FBXL16; FCER1A; KLRB1;NELL2; NMT2;P2RY10; S100A8;SBK1; SLC7A6	1	0.9846	0.0644	0.9585
top300_candidate	10	unpenalized	specificity	0.734	True	0.1	locked_signature	FAM43A; FBXL16; FCER1A; KLRB1;NELL2; NMT2;P2RY10; S100A8;SBK1; SLC7A6	0.7329	0.6642	0.055	0.5677

Table S7 note. No duplicate panel was drawn in any cell (`n_redundant_draws` = 0 throughout). `panel_list_sha256` identifies the exact draw sequence.

Table S8. The 2000 random gene panels drawn for the primary benchmark cell (transportable pool, $k = 10$, L1 at the modal C), one row per draw, genes semicolon-separated and sorted within each panel. Listed here are the first 50 draws; all 2000 are in the data workbook, Supplementary File S3.

draw	genes
1	BET1L;BMP6;BTBD2;CSGALNACT2;KIAA0391;KLC1;PREP;RBM6; SLC16A11;TIAM1
2	CNOT4;HEMK1;IL11;LCK;MGC39584;NDUFAF2;PPAT;SLC30A1;STBD1; UHRF1
3	ATP1B3;CHD3;ESF1;GRB2;KRT33A;PLGLB2;RDH13;SETD8;TRAK1;ZFY
4	CDIPT;GALNT2;HCFC1R1;INTS6;RAB3A;SCAPER;SCO2;TSPAN2;UBL4A; ZDHHC20
5	CENPF;DENND1C;GCC2;KIF21A;NPAT;PLEKHB2;PTDSS1;RDH5;SYAP1; VOPP1
6	ACAT1;CCNF;CRYGS;DNAJC30;FAM13A;PODNL1;RAB33A;SMURF2; SOCS5;SWAP70
7	ANKRD27;C4orf33;CCNF;DPP8;KLF14;LTK;MLKL;NRM;PREX1;PSPH
8	ALKBH3;CYP4F8;ESF1;FCGR1B;GPATCH4;KIF1A;NLRC4;PDE2A; SMARCE1;ZNF395
9	CAPN3;FAM58A;KIAA1143;MLH1;NIPSNAP3A;PRR5L;PSMA5;SLC36A1; UBA1;ZNF791
10	BNIP3L;CEP120;LONRF1;MIA3;RBM38;RNF26;RPL10L;SLA;TRIP6;VEGFB
11	BIRC7;C8orf33;GLUD1;GNG5;GYPE;PEA15;PLEKHF1;RAB3IP;YTHDC1; ZBTB3
12	BAIAP3;BUB3;EIF2B5;EPRS;LACTB;LARP7;RNF219;S100A2;SLC25A37; SLC5A9
13	ADRA2C;CLIC2;KIAA0226;LPCAT3;MED23;NDUFB5;PSEN2;ROGDI; SEZ6L2;THBD
14	AP1M1;CD24;CLTC;COPS4;GCC1;ISOC1;MED8;PRPS1;PYGL;SLC3A2
15	AGPAT5;CDC27;FAM83C;GPA33;ITIH4;KYNU;MAP7D3;PAK1;PGLS; YME1L1
16	CENPT;EMILIN1;GDI1;LYPLAL1;NCOA4;NDUFA7;NTAN1;SPAG9;VAV3; WWP1
17	C1orf174;C9orf16;CCNI;EXOSC8;FBXO5;FOXJ3;NCOR2;NFU1;PSMC5; PTPLAD2
18	ANKRD32;CCR1;DHODH;DMXL1;FIGNL1;HERC2;NDUFB8;NGRN;TXNL4A; ZNF142
19	APLP2;CHCHD5;CMTM2;EED;MRPL28;N4BP2L1;PIK3CB;TSPAN1;VAMP4; ZNF626
20	AGK;C6orf120;DDX23;FOXN2;GINS2;LSM3;MRPS22;SLC11A1;TPCN1; TRAF6
21	ABL2;ALPK1;ATP1B1;CAD;CDCA5;DNM1;SUPV3L1;TRA2A;VANGL1; WDR31
22	CD1E;FIP1L1;FUT1;KIAA0907;MFSD9;MGST1;SLC25A20;UTP23;ZFYVE28; ZNF91
23	CRYBB3;FANCL;ITGAM;MLX;OLFML2B;RBBP6;SHOX;SNX9;TMCC2;ULK1
24	BAZ2B;CBY1;FBXO28;FNBP4;IFIT5;MCM4;MOGS;PSAP;UIMC1;ZMIZ1
25	ACAD8;C19orf33;CCNJL;CD59;ITPKB;PMF1;RPL26L1;SEC23IP;TARSL2; ZCRB1
26	ADI1;C1orf228;DVL1;FAM134B;LPGAT1;PSEN1;SLC37A2;SMURF1;SUDS3; TCEAL8
27	C3orf62;C9orf16;FAM174A;HIF1A;IQCE;KCNH2;PQLC2;TMEM127;UGGT1; ZBTB41
28	ATOH8;C18orf54;CD36;CPA4;GTDC1;MAD2L2;PARP1;SRRT;TMX3;YME1L1
29	AFTPH;ARL4C;C5orf45;CDCA7L;CDK5R1;FAM102B;LILRB1;NCBP1; NUSAP1;RIC8B
30	FRYL;ICAM1;MKRN1;NAP1L4;RWDD1;SMAD1;STX4;TESC;TMEM38B; ZNF438
31	ACAP1;ARFGEF2;C14orf93;EZR;HIATL1;ITFG2;MTCH2;TRAM2;UFD1L; WDR45
32	DNAJC7;FUT7;ODC1;PLCH2;SLC26A2;SNRPA;SNX10;TBC1D8;TRPM2; ZNF274
33	DUSP23;EMB;HOXB9;LPAR6;POLR2F;RAD1;SLC36A1;SYMPK;THOC5; ZCCHC9
34	CNOT8;DLL1;GART;HBZ;HDAC1;HLCS;KIAA0922;MLC1;RAC3;S100A16

draw	genes
35	CSNK2A1;LOC100131541;MCM9;MZF1;NADK;NFKBIB;PRCC;SOAT1; TCL1A;YWHAE
36	BTG3;CBX7;MINPP1;PHF1;PIK3R4;POC5;RBBP9;TIPRL;UBE2C;WNT10B
37	CHRNG;IMMT;KRIT1;PAQR6;SLC34A3;SMCHD1;SNRPB;USP21;WDFY4; ZNF805
38	ACCS;CASP5;CIDEB;LRRC27;MAFB;MST4;NPCDR1;NUBP2;QTRT1; RPS27A
39	BLCAP;C6orf89;C9orf78;ITM2A;MCM10;PRMT1;RPAP3;RPS6KA1;SLC39A13; TBL3
40	CDS2;EPB41;FMN1;FNIP2;HAMP;MYO7B;PMVK;SH2B1;SMC4;ZBTB11
41	CCNG2;CLEC4C;GUF1;HIST1H2BK;MAOB;NFIX;PGM2L1;SLC27A4;VAMP3; WDR5
42	AMACR;CACNA2D4;CACYBP;MAN1C1;NDUFA2;P4HA1;POGK;RAD17; TEAD1;TMEM63B
43	ACTN4;C20orf96;FBXO44;FOXB1;KLHL3;MRPL36;NDUFA6;SACM1L; SLC2A9;UBB
44	AHNAK;BOK;C19orf54;CCDC59;PIP5K1A;PPP2R5B;PSMB8;PSMC1;RAB1A; ZRSR2
45	ABLIM2;ADAM33;ANKLE2;CRLF1;FAM160B2;PRKCA;SPATA13;TTC39C; TTL;ZZZ3
46	ARNTL;CA1;DPM1;PTAR1;SLC11A2;SNRPD2;TCEAL3;TCTA;UNC50; ZDHHC6
47	BEX1;CDK2;LAPTM5;MKNK1;NCAM1;NECAB2;PLEKHB2;RBM43;SCPEP1; TESC
48	ABHD2;AP2S1;ARIH2;ATP5SL;DCAKD;MORF4L2;PGAP2;PHKA2;SEC24B; ZNF276
49	ECHDC1;HIST1H2BC;LMAN2;NCSTN;NUCKS1;PAN2;PODN;SLC36A1; TMEM201;ZNF275
50	CD69;CDV3;EFNB3;KLHL7;LONP1;MOGS;PPP1R8;RAPGEFL1;RCHY1; VPS11

Table S8 note. All 2000 draws are in the workbook. Duplicate panels among them: 0. SHA-256 of the panel list, taken over the `genes` column of all 2000 draws joined by newlines, one panel per line: `9dfc67641741f8916d91dbb4e49af9d2fbead288df1b6f9f63c44de319a1296e`.

Table S9a. Per-fold results of the cross-fitted specificity analysis (Methods 4.6, Section 2.2, Figure 4). In each fold the full selection-and-fit recipe is re-run on the training sepsis cases plus all 42 healthy controls, and only that fold's held-out sepsis cases and held-out non-infected ICU controls are scored by it.

fold	n_train_sepsis	n_train_healthy	n_test_sepsis	n_test_noninfected_icu	best_ C	n_nonzero_ genes	auroc
0	193	42	49	15	0.1	13	0.6966
1	193	42	49	15	0.03	8	0.634
2	194	42	48	15	0.1	9	0.7208
3	194	42	48	15	0.03	4	0.7958
4	194	42	48	15	1	17	0.8028

Table S9b. Out-of-fold predictions for all 317 samples in the specificity comparison, each scored exactly once by a model that never saw it. `p_crossfit_within_fold_rank` is the within-fold percentile rank used for the pooled estimate; `p_locked_in_sample` is the score from the final locked model, which saw every sepsis case in training.

geo_accession	y_true	fold	p_crossfit	p_crossfit_within_fold_rank	p_locked_in_sample
GSM1602802	1	0	0.8587	0.2154	0.9066
GSM1602803	1	0	0.935	0.4615	0.945
GSM1602805	1	3	0.7633	0.3594	0.9457
GSM1602810	1	4	0.9587	0.1094	0.9006
GSM1602811	1	4	0.9998	0.9688	0.9813
GSM1602812	1	4	0.9995	0.7812	0.9708
GSM1602813	1	3	0.8195	0.7812	0.9713
GSM1602815	1	3	0.7807	0.4531	0.949
GSM1602816	1	0	0.9277	0.4154	0.9389
GSM1602818	1	2	0.9668	0.875	0.976
GSM1602819	1	0	0.9721	0.9231	0.9785
GSM1602820	1	4	0.997	0.4688	0.9403
GSM1602823	1	4	0.9993	0.6719	0.9621
GSM1602824	1	3	0.6525	0.1094	0.8437
GSM1602825	1	1	0.74	0.4	0.9351
GSM1602826	1	3	0.8254	0.8438	0.9697

geo_accession	y_true	fold	p_crossfit	p_crossfit_within_fold_rank	p_locked_in_sample
GSM1602827	1	4	0.9868	0.2188	0.9352
GSM1602829	1	0	0.9635	0.7846	0.9677
GSM1602830	1	1	0.7866	0.6615	0.9524
GSM1602831	1	2	0.9561	0.7344	0.9671
GSM1602832	1	0	0.6845	0.06154	0.7611
GSM1602833	1	0	0.9442	0.5846	0.9493
GSM1602834	1	2	0.9406	0.5781	0.9543
GSM1602836	1	0	0.9129	0.3846	0.926
GSM1602837	1	3	0.8146	0.7344	0.9633
GSM1602838	1	3	0.7933	0.5469	0.9607
GSM1602839	1	0	0.9713	0.8923	0.976
GSM1602840	1	1	0.7673	0.5538	0.9529
GSM1602841	1	1	0.7298	0.2769	0.9117
GSM1602842	1	3	0.8214	0.8125	0.9626
GSM1602843	1	2	0.8646	0.2969	0.8915
GSM1602844	1	4	0.9998	0.9531	0.9849
GSM1602845	1	0	0.9742	0.9538	0.9811
GSM1602846	1	1	0.6658	0.1231	0.8439
GSM1602847	1	0	0.909	0.3385	0.916
GSM1602848	1	2	0.8579	0.2656	0.8973
GSM1602849	1	1	0.7204	0.2308	0.9037
GSM1602850	1	4	0.9974	0.4844	0.9458
GSM1602851	1	2	0.9378	0.5312	0.9548
GSM1602852	1	4	0.9953	0.3438	0.9343
GSM1602853	1	1	0.7546	0.4923	0.9444
GSM1602855	1	2	0.9754	0.9688	0.9824
GSM1602856	1	2	0.7998	0.1562	0.8488
GSM1602857	1	0	0.8585	0.2	0.9014
GSM1602858	1	4	0.9998	0.9375	0.9792
GSM1602859	1	1	0.7053	0.1692	0.9229
GSM1602860	1	3	0.807	0.6719	0.9618
GSM1602861	1	0	0.9659	0.8154	0.9762
GSM1602863	1	4	0.9913	0.2656	0.9461
GSM1602866	1	2	0.9624	0.7812	0.9759
GSM1602868	1	4	0.9991	0.6094	0.9716
GSM1602869	1	3	0.8498	0.9688	0.9811
GSM1602873	1	2	0.9635	0.8281	0.9742
GSM1602878	1	1	0.7649	0.5077	0.9503
GSM1602879	1	1	0.8142	0.8462	0.9691
GSM1602880	1	1	0.7502	0.4462	0.9471
GSM1602881	1	3	0.7498	0.2656	0.9296
GSM1602882	1	3	0.7724	0.4219	0.9193
GSM1602883	1	4	0.9995	0.7344	0.9778
GSM1602885	1	4	0.9997	0.875	0.9803
GSM1602886	1	3	0.7505	0.2812	0.9492
GSM1602888	1	1	0.6259	0.07692	0.7919
GSM1602889	1	2	0.9666	0.8594	0.9751
GSM1602902	1	4	0.9995	0.7656	0.9739
GSM1602903	1	1	0.7861	0.6462	0.9647
GSM1602906	1	2	0.8994	0.3438	0.9289
GSM1602907	1	1	0.7391	0.3692	0.9355
GSM1602908	1	3	0.8048	0.6406	0.9633
GSM1602912	1	3	0.7889	0.4844	0.9556
GSM1602921	1	2	0.9012	0.3594	0.9242
GSM1602922	1	2	0.9477	0.6406	0.956
GSM1602923	1	4	0.9998	0.9219	0.9793
GSM1602924	1	1	0.8318	0.9538	0.9815
GSM1602925	1	0	0.9573	0.7231	0.964
GSM1602927	1	2	0.9789	0.9844	0.9846
GSM1602928	1	2	0.9635	0.8125	0.9729
GSM1602929	1	3	0.8119	0.7188	0.9692
GSM1602930	1	0	0.8767	0.2462	0.9075
GSM1602931	1	1	0.7377	0.3538	0.9192
GSM1602932	1	2	0.9748	0.9531	0.98
GSM1602933	1	3	0.805	0.6562	0.9573
GSM1602934	1	4	0.9994	0.7031	0.9732
GSM1602935	1	1	0.7546	0.4769	0.9471
GSM1602936	1	3	0.8207	0.7969	0.9615

geo_accession	y_true	fold	p_crossfit	p_crossfit_within_fold_rank	p_locked_in_sample
GSM1602937	1	4	0.9945	0.3125	0.9181
GSM1602940	1	2	0.9623	0.7656	0.9747
GSM1602942	1	0	0.9734	0.9385	0.9789
GSM1602943	1	0	0.8279	0.1231	0.8831
GSM1602944	1	0	0.9351	0.4769	0.9521
GSM1602945	1	0	0.9442	0.6	0.946
GSM1602946	1	0	0.9674	0.8308	0.9739
GSM1602947	1	2	0.9526	0.7031	0.9594
GSM1602950	1	2	0.715	0.1094	0.7826
GSM1602952	1	3	0.7525	0.2969	0.9336
GSM1602953	1	1	0.7142	0.2	0.9305
GSM1602958	1	1	0.7105	0.1846	0.9185
GSM1602968	1	2	0.7606	0.125	0.822
GSM1602973	1	3	0.5267	0.0625	0.8702
GSM1602974	1	1	0.7852	0.6308	0.9581
GSM1602975	1	0	0.9098	0.3538	0.9261
GSM1602976	1	4	0.9996	0.8438	0.9749
GSM1691861	1	3	0.8322	0.875	0.9786
GSM1691863	1	1	0.789	0.6923	0.9621
GSM1691864	1	2	0.9375	0.5156	0.9543
GSM1691865	1	0	0.9471	0.6308	0.9592
GSM1691866	1	4	0.9975	0.5	0.9397
GSM1691867	1	3	0.7717	0.4062	0.9474
GSM1691868	1	3	0.7176	0.1875	0.9273
GSM1691871	1	4	0.9996	0.7969	0.9759
GSM1691874	1	0	0.9283	0.4308	0.9441
GSM1691877	1	0	0.9466	0.6154	0.9596
GSM1691882	1	1	0.81	0.8308	0.972
GSM1691893	1	0	0.8509	0.1692	0.8816
GSM1691895	1	2	0.8669	0.3125	0.8949
GSM1691896	1	4	0.9995	0.75	0.9631
GSM1691900	1	3	0.7021	0.1719	0.9507
GSM1691901	1	3	0.7822	0.4688	0.9467
GSM1691911	1	1	0.7959	0.7538	0.9565
GSM1691915	1	1	0.7761	0.6	0.958
GSM1691922	1	4	0.9965	0.4219	0.9426
GSM1691923	1	4	0.7369	0.03125	0.8493
GSM1691928	1	2	0.8555	0.25	0.8944
GSM1691935	1	0	0.9544	0.6769	0.969
GSM1691944	1	2	0.8092	0.1719	0.8387
GSM1691954	1	1	0.7757	0.5846	0.9625
GSM1691959	1	0	0.9561	0.7077	0.9652
GSM1691965	1	2	0.9481	0.6562	0.9645
GSM1691968	1	4	0.999	0.5938	0.9723
GSM1691977	1	2	0.9324	0.4844	0.9493
GSM1691980	1	0	0.9598	0.7385	0.9678
GSM1691981	1	4	0.9996	0.8281	0.9757
GSM1691984	1	0	0.908	0.3231	0.9377
GSM1691986	1	4	0.9995	0.7188	0.9702
GSM1691987	1	0	0.9761	0.9846	0.9811
GSM1692005	1	2	0.6677	0.09375	0.7497
GSM1692007	1	0	0.9009	0.3077	0.9129
GSM1692009	1	2	0.9491	0.6719	0.9645
GSM1692016	1	3	0.809	0.6875	0.9779
GSM1692019	1	2	0.9678	0.9062	0.9757
GSM1692022	1	1	0.8209	0.9231	0.9758
GSM1692029	1	2	0.9603	0.75	0.9696
GSM1692030	1	2	0.9528	0.7188	0.9643
GSM1692035	1	4	0.9801	0.1719	0.9058
GSM1692038	1	3	0.8254	0.8594	0.9786
GSM1692040	1	1	0.7664	0.5385	0.9478
GSM1692045	1	3	0.8229	0.8281	0.9747
GSM1692050	1	1	0.82	0.9077	0.9741
GSM1692059	1	0	0.9694	0.8615	0.9779
GSM1692061	1	3	0.7596	0.3438	0.9468
GSM1692063	1	0	0.9371	0.5077	0.9493
GSM1692066	1	2	0.6214	0.0625	0.6892
GSM1692084	1	0	0.9757	0.9692	0.9829

geo_accession	y_true	fold	p_crossfit	p_crossfit_within_fold_rank	p_locked_in_sample
GSM1692086	1	3	0.8427	0.9219	0.9802
GSM1692089	1	3	0.4436	0.04688	0.5533
GSM1692096	1	1	0.7403	0.4154	0.9268
GSM1692101	1	0	0.7625	0.09231	0.8202
GSM1692108	1	4	0.996	0.3594	0.9112
GSM1692114	1	0	0.9516	0.6615	0.9634
GSM1692117	1	1	0.8417	0.9846	0.9804
GSM1692118	1	3	0.7958	0.5625	0.9694
GSM1692119	1	0	0.9705	0.8769	0.977
GSM1692121	1	2	0.9061	0.375	0.9386
GSM1692126	1	3	0.8327	0.9062	0.968
GSM1692132	1	0	0.972	0.9077	0.9818
GSM1692136	1	2	0.9281	0.4688	0.9495
GSM1692137	1	1	0.8177	0.8923	0.9683
GSM1692138	1	2	0.9632	0.7969	0.9697
GSM1692142	1	4	0.9984	0.5625	0.9652
GSM1692157	1	3	0.8187	0.7656	0.969
GSM1692160	1	4	0.9997	0.8594	0.9707
GSM1692165	1	1	0.7153	0.2154	0.9209
GSM1692166	1	3	0.8492	0.9531	0.9737
GSM1692167	1	1	0.7286	0.2462	0.8757
GSM1692169	1	0	0.8965	0.2615	0.9159
GSM1692172	1	0	0.83	0.1385	0.8793
GSM1692177	1	3	0.8094	0.7031	0.9751
GSM1692181	1	2	0.9443	0.5938	0.9543
GSM1692192	1	1	0.8162	0.8769	0.9713
GSM1692198	1	1	0.8054	0.7846	0.968
GSM1692199	1	4	0.995	0.3281	0.924
GSM1692201	1	4	0.9994	0.6875	0.9716
GSM1692203	1	1	0.7945	0.7231	0.9551
GSM1692204	1	0	0.9128	0.3692	0.9346
GSM1692211	1	0	0.939	0.5231	0.9486
GSM1692216	1	1	0.6345	0.1077	0.8121
GSM1692223	1	4	0.9989	0.5781	0.9537
GSM1692238	1	2	0.9727	0.9375	0.9797
GSM1692245	1	0	0.8339	0.1538	0.8824
GSM1692250	1	3	0.2955	0.01562	0.553
GSM1692251	1	1	0.7661	0.5231	0.9597
GSM1692255	1	4	0.9967	0.4531	0.94
GSM1692258	1	2	0.8152	0.1875	0.8627
GSM1692259	1	4	0.9838	0.2031	0.9177
GSM1692261	1	4	0.9981	0.5469	0.9623
GSM1692266	1	1	0.7316	0.3077	0.923
GSM1692268	1	1	0.7518	0.4615	0.9239
GSM1692272	1	2	0.8239	0.2188	0.8745
GSM1692278	1	1	0.7893	0.7077	0.9599
GSM1692302	1	4	0.9999	0.9844	0.9805
GSM1692311	1	3	0.7559	0.3281	0.9537
GSM1692326	1	4	0.9965	0.4062	0.925
GSM1692329	1	3	0.8509	0.9844	0.9827
GSM1692330	1	4	0.9967	0.4375	0.9305
GSM1692334	1	4	0.8338	0.04688	0.7337
GSM1692335	1	3	0.7975	0.5938	0.9664
GSM1692337	1	1	0.8249	0.9385	0.9808
GSM1692352	1	0	0.8217	0.1077	0.8769
GSM1692362	1	1	0.806	0.8	0.9646
GSM1692364	1	0	0.9684	0.8462	0.9762
GSM1692366	1	1	0.8099	0.8154	0.9748
GSM1692372	1	4	0.9978	0.5312	0.9391
GSM1692379	1	3	0.8437	0.9375	0.9802
GSM1692383	1	4	0.9996	0.8125	0.9743
GSM1692384	1	3	0.7967	0.5781	0.9362
GSM1692386	1	3	0.8156	0.75	0.968
GSM1692387	1	2	0.9674	0.8906	0.9747
GSM1692388	1	0	0.9544	0.6923	0.9662
GSM1692392	1	3	0.7697	0.3906	0.9516
GSM1692397	1	4	0.9976	0.5156	0.9652
GSM1692399	1	0	0.9368	0.4923	0.9454

geo_accession	y_true	fold	p_crossfit	p_crossfit_within_fold_rank	p_locked_in_sample
GSM1692410	1	1	0.7314	0.2923	0.9383
GSM1692412	1	3	0.8326	0.8906	0.9749
GSM1692413	1	2	0.8947	0.3281	0.9386
GSM1692416	1	3	0.8035	0.625	0.9676
GSM1692420	1	2	0.95	0.6875	0.9601
GSM1692424	1	2	0.9684	0.9219	0.976
GSM1692427	1	2	0.9448	0.625	0.9583
GSM1692429	1	1	0.7326	0.3385	0.9308
GSM1692430	1	4	0.9718	0.1562	0.9104
GSM1692443	1	1	0.7412	0.4308	0.9187
GSM1692452	1	4	0.9992	0.625	0.9517
GSM1692453	1	4	0.9992	0.6406	0.9477
GSM1692455	1	2	0.916	0.4062	0.9314
GSM1692459	1	4	0.9997	0.8906	0.976
GSM1692477	1	0	0.9637	0.8	0.9723
GSM1692482	1	3	0.8021	0.6094	0.9688
GSM1692485	1	1	0.7887	0.6769	0.959
GSM1692487	1	2	0.858	0.2812	0.8956
GSM1692489	1	0	0.9405	0.5538	0.9545
GSM1692492	1	1	0.5675	0.04615	0.6931
GSM1692498	1	3	0.4303	0.03125	0.6671
GSM1692501	1	2	0.9337	0.5	0.9532
GSM1602801	0	0	0.8992	0.2769	0.9228
GSM1602804	0	4	0.4783	0.01562	0.634
GSM1602806	0	0	0.8547	0.1846	0.8871
GSM1602808	0	0	0.7268	0.07692	0.7949
GSM1602814	0	2	0.9227	0.4531	0.9393
GSM1602817	0	4	0.9926	0.2969	0.937
GSM1602821	0	1	0.8151	0.8615	0.9743
GSM1602822	0	0	0.6193	0.03077	0.6589
GSM1602828	0	4	0.9918	0.2812	0.8905
GSM1602835	0	4	0.9543	0.09375	0.9065
GSM1602854	0	2	0.8531	0.2344	0.8913
GSM1602865	0	2	0.9086	0.3906	0.9393
GSM1602867	0	3	0.7472	0.25	0.9494
GSM1602877	0	1	0.5869	0.06154	0.7337
GSM1602887	0	0	0.9006	0.2923	0.9296
GSM1602890	0	1	0.8365	0.9692	0.9789
GSM1602904	0	0	0.8671	0.2308	0.891
GSM1602905	0	2	0.5339	0.04688	0.6096
GSM1602918	0	3	0.6699	0.125	0.8137
GSM1602919	0	4	0.9808	0.1875	0.8709
GSM1602926	0	2	0.8202	0.2031	0.8185
GSM1602938	0	2	0.938	0.5469	0.9514
GSM1602939	0	1	0.7316	0.3231	0.9063
GSM1602941	0	2	0.4286	0.03125	0.4989
GSM1602949	0	3	0.7433	0.2188	0.8817
GSM1602951	0	2	0.9638	0.8438	0.9745
GSM1602955	0	0	0.9391	0.5385	0.9523
GSM1602959	0	3	0.6774	0.1406	0.8195
GSM1602965	0	3	0.5929	0.07812	0.5844
GSM1602967	0	2	0.4219	0.01562	0.4923
GSM1602970	0	3	0.7456	0.2344	0.9307
GSM1602971	0	3	0.7364	0.2031	0.9226
GSM1602972	0	1	0.7727	0.5692	0.9623
GSM1691902	0	4	0.9083	0.0625	0.7722
GSM1691913	0	1	0.4409	0.03077	0.4145
GSM1691917	0	4	0.9892	0.2344	0.9363
GSM1691920	0	0	0.9178	0.4	0.9324
GSM1691926	0	3	0.7001	0.1562	0.8706
GSM1691936	0	0	0.9487	0.6462	0.9659
GSM1691952	0	1	0.7394	0.3846	0.9007
GSM1692053	0	2	0.9204	0.4375	0.9343
GSM1692054	0	0	0.9429	0.5692	0.9551
GSM1692075	0	4	0.991	0.25	0.9112
GSM1692078	0	0	0.9292	0.4462	0.933
GSM1692080	0	2	0.6417	0.07812	0.7329
GSM1692110	0	4	0.9962	0.3906	0.9323

geo_accession	y_true	fold	p_crossfit	p_crossfit_within_fold_rank	p_locked_in_sample
GSM1692147	0	3	0.7896	0.5156	0.9673
GSM1692150	0	3	0.7527	0.3125	0.8758
GSM1692155	0	4	0.9993	0.6562	0.9592
GSM1692176	0	1	0.3729	0.01538	0.2174
GSM1692195	0	1	0.7959	0.7385	0.9567
GSM1692219	0	2	0.9394	0.5625	0.9519
GSM1692231	0	1	0.6945	0.1385	0.899
GSM1692239	0	3	0.7639	0.375	0.9469
GSM1692248	0	1	0.7978	0.7692	0.9634
GSM1692249	0	2	0.9444	0.6094	0.9587
GSM1692270	0	4	0.9687	0.1406	0.8533
GSM1692275	0	3	0.6127	0.09375	0.7666
GSM1692281	0	0	0.5812	0.01538	0.6073
GSM1692314	0	2	0.9192	0.4219	0.9393
GSM1692321	0	4	0.9962	0.375	0.9448
GSM1692328	0	3	0.7906	0.5312	0.9267
GSM1692411	0	4	0.9523	0.07812	0.9083
GSM1692419	0	2	0.7614	0.1406	0.7907
GSM1692442	0	1	0.7029	0.1538	0.8823
GSM1692457	0	0	0.9601	0.7538	0.9695
GSM1692463	0	3	0.7892	0.5	0.9565
GSM1692468	0	1	0.7297	0.2615	0.9105
GSM1692474	0	0	0.9627	0.7692	0.9709
GSM1692475	0	1	0.6287	0.09231	0.8036
GSM1692476	0	1	0.7851	0.6154	0.961
GSM1692483	0	4	0.9659	0.125	0.893
GSM1692490	0	4	0.9997	0.9062	0.9753
GSM1692496	0	0	0.6346	0.04615	0.6973
GSM1692502	0	3	0.7745	0.4375	0.9582

replicate	oob_auroc	validation_auroc	specificity_auroc	n_ oob	n_nonzero_genes	chosen_ C

Table S10a. Full-pipeline bootstrap replicates (Methods 4.7, Section 2.3). Each row is one of 200 replicates in which the top-300 filter was re-derived, C was re-tuned by inner cross-validation, and the L1 model was refitted, then evaluated out-of-bag and scored on both external cohorts.

replicate	oob_auroc	validation_auroc	specificity_auroc	n_ oob	n_nonzero_genes	chosen_ C
0	0.9993	1	0.7706	109	6	0.1
1	1	1	0.7415	104	10	0.1
2	1	1	0.7123	113	6	0.03
3	0.9952	1	0.698	98	4	0.03
4	1	1	0.7234	101	4	0.03
5	1	1	0.7397	113	5	0.03
6	0.9979	1	0.6868	106	7	0.03
7	1	1	0.7263	99	6	0.03
8	1	1	0.6875	104	6	0.03
9	1	1	0.7105	105	6	0.03
10	1	1	0.7222	104	11	0.3
11	1	1	0.7072	106	7	0.03
12	1	1	0.7425	101	12	0.1
13	0.9889	1	0.7136	102	5	0.03
14	0.9987	1	0.7101	106	3	0.03
15	0.9971	1	0.6981	107	6	0.03
16	1	1	0.7705	100	6	0.03
17	1	1	0.7496	104	10	0.1
18	0.9992	1	0.6702	94	5	0.03
19	0.9918	1	0.7636	104	15	0.3
20	0.9993	1	0.709	110	4	0.03
21	1	1	0.7245	103	13	0.3
22	1	1	0.7563	104	6	0.03
23	0.9993	1	0.6764	103	4	0.03
24	1	1	0.6704	100	4	0.03
25	1	1	0.7048	101	5	0.03
26	1	1	0.7403	103	10	0.1
27	1	1	0.6975	99	6	0.03
28	0.9961	1	0.6919	107	12	0.1
29	0.9968	1	0.7423	104	6	0.03
30	1	1	0.7144	105	6	0.03
31	1	1	0.7545	107	13	1
32	1	1	0.6391	114	6	0.03
33	1	1	0.7224	110	6	0.1
34	1	1	0.6905	108	14	1
35	0.9989	1	0.6717	113	7	0.03
36	1	1	0.7112	101	16	0.1
37	1	1	0.6783	104	5	0.03
38	0.997	1	0.688	104	8	0.03
39	0.9938	1	0.7031	100	7	0.03
40	0.995	1	0.6969	114	12	0.1
41	0.9992	1	0.7447	110	9	0.1
42	1	1	0.7163	96	4	0.03
43	1	1	0.7449	102	7	0.03
44	1	1	0.7077	103	10	0.1
45	0.9986	1	0.7187	103	6	0.03
46	0.9889	1	0.6802	98	3	0.03
47	0.9984	1	0.6993	104	3	0.03
48	1	1	0.7255	106	9	0.1
49	1	1	0.743	101	13	0.3
50	1	1	0.716	108	10	0.1
51	0.9933	1	0.7001	117	5	0.03
52	0.9941	1	0.6986	106	9	0.1
53	1	1	0.6744	100	6	0.1
54	0.9991	1	0.6615	107	5	0.03
55	0.9985	1	0.6492	102	5	0.03
56	0.9989	1	0.707	103	7	0.03
57	1	1	0.7495	114	6	0.03
58	1	1	0.6968	92	4	0.03
59	0.9992	1	0.7631	101	4	0.03

replicate	oob_auroc	validation_auroc	specificity_auroc	n_ oob	n_nonzero_genes	chosen_ C
60	0.9906	1	0.6928	105	5	0.03
61	1	1	0.7896	108	8	0.03
62	0.9992	1	0.6634	109	7	0.03
63	1	1	0.7212	102	7	0.03
64	1	1	0.7189	111	8	0.1
65	1	1	0.6813	100	7	0.03
66	1	1	0.6975	100	8	0.03
67	0.9985	1	0.6857	100	4	0.03
68	1	1	0.6856	100	7	0.1
69	0.9983	1	0.6697	99	6	0.03
70	1	1	0.7394	102	10	0.1
71	1	1	0.7274	116	9	0.03
72	1	1	0.7298	108	8	0.03
73	1	1	0.7147	104	13	1
74	1	1	0.7947	107	4	0.03
75	0.9981	1	0.692	106	9	0.1
76	1	1	0.743	113	9	0.1
77	1	1	0.7174	105	18	1
78	0.9941	1	0.6845	97	4	0.03
79	1	1	0.7543	99	9	0.1
80	1	1	0.6806	103	7	0.03
81	1	1	0.6659	107	4	0.03
82	0.9986	1	0.6512	108	4	0.03
83	1	1	0.7268	94	7	0.03
84	1	1	0.7223	102	6	0.03
85	1	1	0.7385	106	10	0.1
86	1	1	0.6812	112	4	0.03
87	1	1	0.7349	101	13	0.1
88	1	1	0.6824	103	4	0.03
89	1	1	0.7196	105	8	0.1
90	1	1	0.7642	105	10	0.3
91	1	1	0.7673	109	5	0.03
92	0.9985	1	0.6746	100	5	0.03
93	1	1	0.7207	99	17	0.3
94	1	1	0.7285	98	5	0.03
95	1	1	0.7628	101	16	0.3
96	1	1	0.6948	104	4	0.03
97	0.9994	1	0.7172	108	5	0.03
98	1	1	0.7219	105	10	0.1
99	0.9964	0.9986	0.6917	104	3	0.03
100	1	1	0.7229	108	7	0.03
101	0.9916	1	0.6873	105	5	0.03
102	1	1	0.7417	97	10	0.1
103	0.9987	1	0.7259	110	5	0.03
104	0.9992	1	0.7104	102	6	0.03
105	1	1	0.736	108	5	0.03
106	0.9944	1	0.6837	95	10	0.1
107	0.998	1	0.6942	102	4	0.03
108	0.9963	1	0.7361	106	5	0.03
109	0.9948	1	0.7048	113	10	0.1
110	0.9987	1	0.6733	108	3	0.03
111	0.9954	1	0.6895	106	4	0.03
112	0.9885	1	0.7175	108	16	0.3
113	0.9948	1	0.7219	105	8	0.03
114	1	1	0.7307	108	12	0.1
115	1	1	0.7342	112	14	0.3
116	1	1	0.6904	109	6	0.03
117	1	1	0.7429	104	6	0.03
118	1	1	0.7119	104	6	0.03
119	0.9899	1	0.6852	99	7	0.03
120	1	1	0.7193	104	19	1
121	0.9974	1	0.7245	98	11	0.1
122	0.9992	1	0.7055	102	5	0.03
123	0.9993	1	0.7845	109	5	0.03
124	1	1	0.7493	97	9	0.1
125	1	1	0.7337	102	18	1
126	0.9978	1	0.7497	106	12	0.1

replicate	oob_auroc	validation_auroc	specificity_auroc	n_ oob	n_nonzero_genes	chosen_ C
127	1	1	0.731	100	13	0.1
128	1	1	0.7162	106	12	0.1
129	0.9959	1	0.7251	104	7	0.03
130	0.9994	1	0.7321	114	5	0.03
131	1	1	0.7285	103	4	0.03
132	1	1	0.7355	107	5	0.03
133	0.9993	1	0.7398	104	7	0.03
134	1	1	0.6725	109	5	0.03
135	1	1	0.7425	98	7	0.03
136	0.9991	1	0.6785	102	3	0.03
137	1	1	0.7177	104	15	0.3
138	0.9987	1	0.7295	102	3	0.03
139	1	1	0.7646	102	5	0.03
140	1	1	0.6654	116	5	0.03
141	1	1	0.7241	114	7	0.03
142	1	1	0.6676	107	6	0.03
143	1	1	0.7115	109	3	0.03
144	1	1	0.726	102	14	0.1
145	1	1	0.6745	105	3	0.03
146	1	1	0.7344	110	14	0.3
147	0.9986	1	0.7218	109	5	0.03
148	1	1	0.6934	107	6	0.1
149	0.9985	1	0.644	105	5	0.03
150	0.9992	1	0.7689	103	5	0.03
151	0.9986	1	0.7056	104	8	0.03
152	1	1	0.7436	105	9	0.03
153	1	1	0.7312	97	12	0.3
154	1	1	0.7717	111	12	0.3
155	1	1	0.739	104	19	1
156	0.9991	1	0.7237	104	6	0.03
157	0.9992	1	0.6682	102	5	0.03
158	0.9941	1	0.6975	106	6	0.03
159	1	1	0.77	106	8	0.1
160	1	1	0.7329	102	11	0.1
161	1	1	0.7423	98	6	0.03
162	1	1	0.7389	92	10	0.1
163	0.9978	1	0.6836	108	5	0.03
164	0.9993	1	0.6789	109	5	0.03
165	0.9935	1	0.7057	107	11	0.1
166	1	1	0.7324	103	11	0.1
167	0.9994	1	0.7392	111	4	0.03
168	1	1	0.7428	104	9	0.1
169	1	1	0.7223	111	5	0.03
170	0.9992	1	0.7126	104	10	0.03
171	0.9938	1	0.7582	103	12	0.3
172	1	1	0.681	103	6	0.03
173	1	1	0.7034	107	5	0.03
174	0.9911	1	0.6734	102	5	0.03
175	1	1	0.7062	109	8	0.03
176	1	1	0.748	102	6	0.1
177	0.9992	1	0.6613	95	5	0.03
178	1	1	0.7323	113	11	0.1
179	1	1	0.725	98	7	0.03
180	0.9991	1	0.7723	98	18	1
181	1	1	0.6752	103	8	0.03
182	0.9931	1	0.6975	107	3	0.03
183	1	1	0.7212	106	4	0.03
184	0.9978	1	0.6932	105	7	0.03
185	1	1	0.7243	106	11	0.1
186	1	1	0.7278	106	6	0.03
187	1	1	0.6867	109	6	0.03
188	0.9958	1	0.6988	106	14	1
189	0.9958	1	0.7102	101	6	0.03
190	1	1	0.7457	105	7	0.1
191	1	1	0.7357	95	13	0.3
192	1	1	0.7162	102	5	0.03
193	1	1	0.7219	112	8	0.03

replicate	oob_auroc	validation_auroc	specificity_auroc	n_<br>oob	n_nonzero_genes	chosen_<br>C
194	1	1	0.6937	107	5	0.03
195	1	1	0.7075	101	6	0.03
196	0.9988	1	0.7567	107	6	0.03
197	0.9946	1	0.7004	109	3	0.03
198	0.9944	1	0.7161	103	5	0.03
199	0.9904	1	0.6758	111	8	0.03

Table S10b. Gene re-selection frequency across the same 200 replicates. 109 distinct genes were selected at least once; the 10 genes of the locked signature are flagged.

gene_symbol	n_selected	selection_frequency	in_locked_signature
P2RY10	153	0.765	True
NELL2	103	0.515	True
S100A12	87	0.435	False
S100A8	83	0.415	True
NMT2	82	0.41	True
ANXA3	80	0.4	False
FCER1A	78	0.39	True
FBXL16	71	0.355	True
FAM43A	66	0.33	True
EPHX2	57	0.285	False
SBK1	53	0.265	True
ARG1	46	0.23	False
SLC7A6	36	0.18	True
PATZ1	33	0.165	False
PID1	31	0.155	False
WDR82	25	0.125	False
IL11RA	24	0.12	False
CD96	23	0.115	False
CCR3	22	0.11	False
CD177	22	0.11	False
PAFAH2	18	0.09	False
UBASH3A	18	0.09	False
AQP3	18	0.09	False
KLRB1	16	0.08	True
CD247	15	0.075	False
TCTN1	14	0.07	False
SIRPG	13	0.065	False
RNF125	10	0.05	False
GTPBP3	10	0.05	False
GATA3	9	0.045	False
DHRS3	9	0.045	False
CLIC3	8	0.04	False
DYRK2	8	0.04	False
CACNA2D3	7	0.035	False
PRKCQ	7	0.035	False
VNN1	7	0.035	False
FCRL3	7	0.035	False
TIAM1	6	0.03	False
PHTF1	6	0.03	False
NDUFB3	6	0.03	False
CLK2	6	0.03	False
MBOAT2	5	0.025	False
QRICH1	5	0.025	False
TBC1D4	5	0.025	False
STMN3	4	0.02	False
PASK	4	0.02	False
OXNAD1	4	0.02	False
BCL2	4	0.02	False
TNFRSF25	4	0.02	False
WHAMM	4	0.02	False
GLOD4	4	0.02	False
NLRC3	3	0.015	False
LRRN3	3	0.015	False
MAFG	3	0.015	False
SIDT1	3	0.015	False
IL10RA	3	0.015	False

gene_symbol	n_selected	selection_frequency	in_locked_signature
RAB13	3	0.015	False
SLC26A8	3	0.015	False
RAB10	2	0.01	False
DDX28	2	0.01	False
C16orf80	2	0.01	False
NACA	2	0.01	False
CCR6	2	0.01	False
S1PR5	2	0.01	False
SH2B3	2	0.01	False
IK	2	0.01	False
SPN	2	0.01	False
C12orf65	2	0.01	False
TMEM109	2	0.01	False
ARL4A	2	0.01	False
MRPS27	1	0.005	False
DPP4	1	0.005	False
LILRA5	1	0.005	False
TLR5	1	0.005	False
PYHIN1	1	0.005	False
CD4	1	0.005	False
NOV	1	0.005	False
KLF2	1	0.005	False
TNIK	1	0.005	False
PTP4A2	1	0.005	False
UBE2F	1	0.005	False
CKAP4	1	0.005	False
FAM162A	1	0.005	False
SLC9A8	1	0.005	False
NOL11	1	0.005	False
MAP4K1	1	0.005	False
TTC27	1	0.005	False
UTRN	1	0.005	False
UFSP2	1	0.005	False
GATA2	1	0.005	False
SAMD3	1	0.005	False
PABPC1	1	0.005	False
HNRNPA0	1	0.005	False
EOMES	1	0.005	False
PWP2	1	0.005	False
BLCAP	1	0.005	False
MDN1	1	0.005	False
ZNF512	1	0.005	False
HMGB2	1	0.005	False
FAM102A	1	0.005	False
OSGEPL1	1	0.005	False
SOCS3	1	0.005	False
FKBP5	1	0.005	False
GZMM	1	0.005	False
BCL9L	1	0.005	False
CDKN2D	1	0.005	False
NCBP2	1	0.005	False
PEA15	1	0.005	False
SLC22A4	1	0.005	False

Table S10b note. All 109 genes are listed.

Table S11a. Threshold-free performance for every evaluation cohort (Methods 4.10, Section 2.2, Table 3).

cohort	n	n_case	n_control	auroc	auroc_ci_method	auroc_ci_lo	auroc_ci_hi	pr_auc	pr_auc_baseline_prevalence	brier	calibration_intercept	calibration_slope	calibration_note	note
validation_locked	53	28	25	1	one-sided conservative lower bound (rule of three)	0.8871	nan	1	0.5283	0.054	nan	nan	not estimable (LinAlgError); logistic recalibration diverges under complete separation	locked model, no refit, external cohort GSE57065, sepsis vs healthy
specificity_crossfit	317	242	75	0.6315	DeLong (subject-level)	0.5536	0.703	0.8379	0.7634	0.1818	-0.6889	0.2399	nan	cross-fitted: every sample scored by a model that never saw it; sepsis vs non-infected ICU. This is the clinically relevant row.
specificity_crossfit_rank	317	242	75	0.7295	DeLong (subject-level)	0.6604	0.789	0.8865	0.7634	0.249	1.706	0.5518	nan	cross-fitted, within-fold percentile ranks; use the AUROC and PR-AUC from this row, not its threshold metrics (ranks have no natural operating point)
specificity_locked_in_sample	317	242	75	0.7329	DeLong (subject-level)	0.6643	0.792	0.8942	0.7634	0.1857	-1.455	0.9675	nan	final locked model: all 242 sepsis cases were in the training set; retained only to quantify the optimism of that estimate

Table S11b. Threshold-based performance at a fixed 0.5 cut-off and at the Youden-optimal cut-off. Predictive values are given at cohort prevalence and at a 10% reference prevalence.

threshold	tp	fp	tn	fn	sensitivity	specificity	accuracy	balanced__ accuracy	f1	cohort__ prevalence	ppv__at__cohort__ prevalence	npv__at__cohort__ prevalence	ppv__at__prevalence__ 0.1	npv__at__prevalence__ 0.1	cohort	thres[illegible]
0.5	26	0	25	2	0.9286	1	0.9623	0.9643	0.963	0.5283	1	0.9259	1	0.9921	validation__ locked	fixe[illegible]
0.4188	28	0	25	0	1	1	1	1	1	0.5283	1	1	1	1	validation__ locked	you[illegible] sam[illegible]
0.5	239	70	5	3	0.9876	0.0667	0.7697	0.5271	0.8675	0.7634	0.7735	0.625	0.1052	0.9798	specificity__ crossfit	fixe[illegible]
0.7498	211	48	27	31	0.8719	0.36	0.7508	0.616	0.8423	0.7634	0.8147	0.4655	0.1315	0.962	specificity__ crossfit	you[illegible] sam[illegible]
0.5	140	20	55	102	0.5785	0.7333	0.6151	0.6559	0.6965	0.7634	0.875	0.3503	0.1942	0.94	specificity__ crossfit__ rank	fixe[illegible]
0.4615	152	20	55	90	0.6281	0.7333	0.653	0.6807	0.7343	0.7634	0.8837	0.3793	0.2074	0.9467	specificity__ crossfit__ rank	you[illegible] sam[illegible]
0.5	242	71	4	0	1	0.0533	0.776	0.5267	0.8721	0.7634	0.7732	1	0.105	1	specificity__ locked__in__ sample	fixe[illegible]
0.9397	162	23	52	80	0.6694	0.6933	0.6751	0.6814	0.7588	0.7634	0.8757	0.3939	0.1952	0.9497	specificity__ locked__in__ sample	you[illegible] sam[illegible]

Table S12a. Demographic balance and between-group tests for both evaluation contrasts (Methods 4.11, Section 2.2).

contrast	group	n	age_ mean	age_ sd	pct_female	age_welch_ t	age_p	sex_chi2	sex_p
sepsis_vs_noninfected_icu_crossfit	case	242	61.3	15.2	40.5	nan	nan	nan	nan
sepsis_vs_noninfected_icu_crossfit	control	75	59.1	15.5	34.7	nan	nan	nan	nan
sepsis_vs_noninfected_icu_crossfit	TEST	317	nan	nan	nan	1.085	0.28	0.59	0.442
sepsis_vs_healthy_validation_locked	case	28	62.7	15.8	32.1	nan	nan	nan	nan
sepsis_vs_healthy_validation_locked	control	25	44.5	11.2	80	nan	nan	nan	nan
sepsis_vs_healthy_validation_locked	TEST	53	nan	nan	nan	4.893	1.13e-05	10.35	0.00129

Table S12b. AUROC within sex strata and age tertiles for both contrasts.

contrast	stratum	n	n_case	auroc	age_range
sepsis_vs_noninfected_icu_crossfit	sex=female	124	98	0.7329	nan
sepsis_vs_noninfected_icu_crossfit	sex=male	193	144	0.7317	nan
sepsis_vs_noninfected_icu_crossfit	age_T1_ young	112	81	0.7899	18-57
sepsis_vs_noninfected_icu_crossfit	age_T2_mid	100	77	0.7326	58-68
sepsis_vs_noninfected_icu_crossfit	age_T3_old	105	84	0.6182	69-88
sepsis_vs_healthy_validation_locked	sex=female	29	9	1	nan
sepsis_vs_healthy_validation_locked	sex=male	24	19	1	nan
sepsis_vs_healthy_validation_locked	age_T1_ young	18	6	1	19-47
sepsis_vs_healthy_validation_locked	age_T2_mid	17	5	1	48-58
sepsis_vs_healthy_validation_locked	age_T3_old	18	17	1	59-84

Table S13. Label-permutation test (Methods 4.9, Section 2.1, Figure 2). 1000 permutations of the discovery class labels, with the top-300 candidate filter re-derived inside every permutation.

task	observed_auroc	n_ permutations	null_ mean	null_sd	null_p95	null_ max	n_perm_ge_ observed	empirical_p
validation	1	1000	0.5141	0.2475	0.9001	0.9914	0	0.001
specificity	0.7329	1000	0.5154	0.0804	0.6449	0.746	3	0.004

Table S14a. Inventory of the GSE65682 series (Methods 4.1). The 443 excluded samples belong to neither labeled sub-study; every sample title carries a distinct code.

item	n
GSM samples in the series	802
distinct sample-title codes in the series	802
analyzed (labeled) samples	359
excluded samples	443
excluded: sepsis patients in the MARS endotype cohorts	248
excluded: draws taken at an ICU-acquired infection event	3
excluded: no diagnostic or cohort label	192

Table S14b. Scan-batch and sub-study checks inside the discovery series (Sections 2.2 and 3.5). Scan date is parsed from each sample title. Cross-validated AUROCs use an L2 logistic model standardized inside 5 folds. Cross-fitted AUROCs use the within-fold percentile ranks of Table S9b. `value_pct` repeats proportions and AUROCs as percentages.

check	n	n_cases	n_controls	value	value_pct	note
scan date vs group, sepsis/healthy: chi-square	284	nan	nan	77.62	nan	p = 3.1e-11; healthy scanned on 7 of 14 dates
scan date vs group, sepsis/non-infected: chi-square	317	nan	nan	31.27	nan	p = 0.0031
2000 highest-variance genes predict scan date among sepsis patients: mean one-vs-rest CV AUROC	242	nan	nan	0.3782	37.8	range 0.212 to 0.469 over 13 dates
10 signature genes predict scan date among sepsis patients: mean one-vs-rest CV AUROC	242	nan	nan	0.4399	44	range 0.318 to 0.599 over 13 dates
locked signature, sepsis vs healthy, all scan dates (in-sample AUROC)	284	nan	nan	1	100	nan
locked signature, sepsis vs healthy, healthy-scan dates only (in-sample AUROC)	145	103	42	1	100	7 scan dates
sepsis prevalence, pneumonia sub-study	225	192	33	0.8533	85.3	nan
sepsis prevalence, abdominal sub-study	92	50	42	0.5435	54.3	nan
2000 highest-variance genes predict sub-study among sepsis patients: CV AUROC	242	nan	nan	0.8857	88.6	nan
2000 highest-variance genes predict sub-study among non-infected patients: CV AUROC	75	nan	nan	0.8925	89.2	nan
10 signature genes predict sub-study among sepsis patients: CV AUROC	242	nan	nan	0.7259	72.6	nan
10 signature genes predict sub-study among non-infected patients: CV AUROC	75	nan	nan	0.6227	62.3	nan
sub-study label alone as a predictor of sepsis: AUROC	317	nan	nan	0.6767	67.7	0.5 would mean the sub-study carries no outcome information
cross-fitted AUROC, both sub-studies pooled (as reported)	317	nan	nan	0.7295	72.9	nan
cross-fitted AUROC, pneumonia sub-study only	225	192	33	0.7374	73.7	nan
cross-fitted AUROC, abdominal sub-study only	92	50	42	0.8817	88.2	nan
cross-fitted AUROC, sub-study-stratified (weighted by case-control pairs)	317	nan	nan	0.7733	77.3	within-sub-study comparisons only
